# Ru-NHC-Catalyzed Asymmetric, Complete Hydrogenation of Indoles and Benzofurans: One Catalyst with Dual Function

**DOI:** 10.1021/jacs.3c04983

**Published:** 2023-07-12

**Authors:** Fuhao Zhang, Himadri Sekhar Sasmal, Constantin G. Daniliuc, Frank Glorius

**Affiliations:** Westfälische Wilhelms-Universität Münster Organisch-Chemisches Institut, Corrensstrasse 36, 48149 Münster, Germany

## Abstract

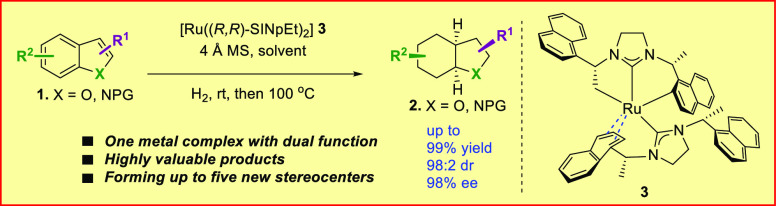

The highly enantioselective and complete hydrogenation of protected indoles and benzofurans has been developed, affording facile access to a range of chiral three-dimensional octahydroindoles and octahydrobenzofurans, which are prevalent in many bioactive molecules and organocatalysts. Remarkably, we are in control of the nature of the ruthenium N-heterocyclic carbene complex and employed the complex as both homogeneous and heterogeneous catalysts, providing new avenues for its potential applications in the asymmetric hydrogenation of more challenging aromatic compounds.

The transformation and utilization of inexpensive and readily available arenes have become a principal area of research.^1−4^ The complete hydrogenation of arenes^5−10^ is one of the most effective methods for converting planar molecules into saturated three-dimensional structures, which are critical building blocks in many aspects of life.^4,11−22^ However, asymmetric hydrogenation of arenes has historically been a major challenge due to the lack of enantioselective catalysts, and the successful cases have largely been confined to the rings with weak aromaticity, such as heteroaromatic rings^5,23−30^ and fused arenes.^31−35^ The combination of heterogeneous and homogeneous catalysis has proven to be a promising method for hydrogenation of aromatic compounds.^36−39^ Recently, Andersson’s group utilized an excessive amount of ligands compared to the Rh precursor to generate both homo- and heterogeneous catalysts in the reaction. Subsequently, they took advantage of the high reaction rate of homogeneous hydrogenation and the induction period of heterogeneous hydrogenation to achieve an asymmetric and complete reduction of arenes (Figure 1a).^38^ Additionally, we realized an unprecedented protocol for asymmetric and complete reduction of the benzofuran through partial hydrogenation of the benzofuran ring using a homogeneous Ru catalyst, followed by the hydrogenation of the benzene ring catalyzed by an *in situ* generated Rh heterogeneous catalyst (Figure 1b).^39^

**Figure 1:**
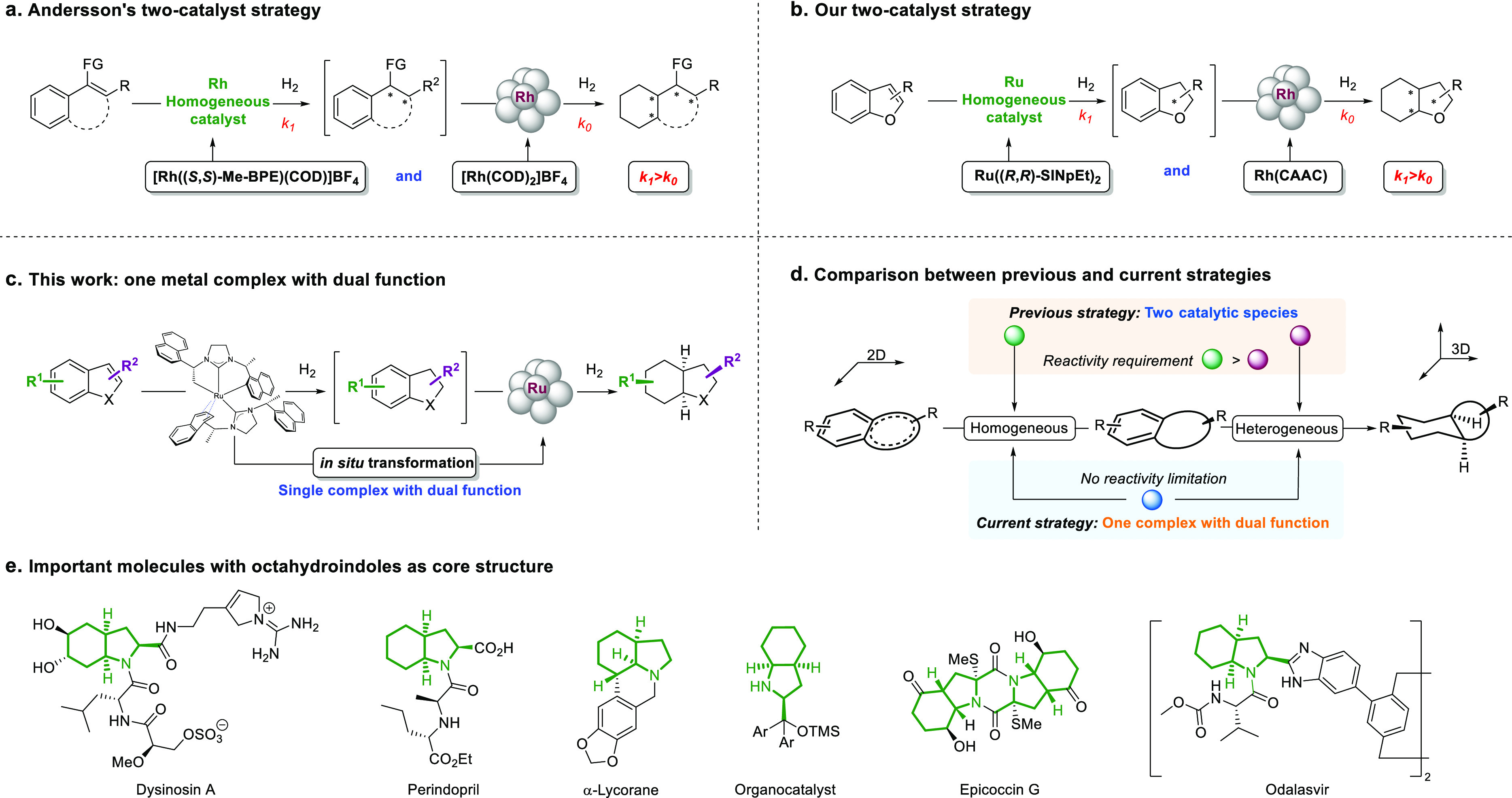
(a–d) Development of a new synergistic catalytic system using one single molecular complex to access saturated octahydroindoles and octahydrobenzofurans. (e) Representative marketed pharmaceuticals, natural products, and catalysts containing octahydroindole moieties.

Due to the presence of two catalysts, the success of the aforementioned two examples (Figure 1a, 1b) is primarily attributed to the compatibility of homogeneous and heterogeneous catalysts. For instance, the reactivity of the homogeneous catalyst to the substrate has to be higher than that of the heterogeneous catalyst to avoid the racemic background reaction and achieve high enantioselectivity. Therefore, we propose a new approach that utilizes both homogeneous and heterogeneous catalysts in succession in one pot with only one species of catalyst in each stage. This would allow us to completely avoid the racemic background reduction independent of the reactivity of the substrate, thus expanding the potential substrate scope. To achieve this, the catalyst must be able to play different roles in different hydrogenation steps. For instance, in the first step, the catalyst should perform as an active homogeneous catalyst, catalyzing the partially enantioselective reduction and then transforming into a heterogeneous catalyst to hydrogenate the more challenging aromatic rings (Figure 1d).

Octahydroindoles are important structural motifs, prevalent in many marketed drugs,^40,41^ natural products,^42,43^ bioactive molecules,^44^ and organocatalysts (Figure 1e).^45,46^ Therefore the development of synthetic methods for chiral octahydroindoles is of high importance.^47−55^ Although there are some reports on the asymmetric partial hydrogenation of indoles,^56−70^ however, to the best of our knowledge, chiral octahydroindoles have not been accessed via complete, enantioselective hydrogenation of indoles despite the straightforwardness of the transformation.^71−77^ Therefore, we proposed a novel enantioselective hydrogenation utilizing dual catalysis driven by a single metal complex to access chiral octahydroindoles. Our group previously developed a Ru-chiral carbene catalyst (Ru((*R*,*R*)-SINpEt)_2_) with excellent reactivity and enantioselectivity in the hydrogenation of heterocyclic aromatic compounds.^31,78−82^ Drawing on the reported literature and our research on carbene chemistry, we envisioned that metal–carbene complexes could be promising to fulfill the requirements for an *in situ* transformable catalyst since these complexes can be converted into nanosized heterogeneous particles.^7,83−86^ We herein report highly enantioselective and diastereoselective complete hydrogenation of indoles, using Ru((*R*,*R*)-SINpEt)_2_ as a dual functional catalyst, to synthesize a wide range of chiral octahydroindoles (Figure 1c). In addition, this strategy was found to be generalized for the complete hydrogenation of benzofurans. The architecturally complex octahydrobenzofurans were thus obtained readily as well.

To help probe the one-pot dual-catalysis strategy using the Ru-NHC catalyst, we opted for *N*-Boc-protected 3-methyl-indole **1a** as our model substrate. We initially conducted the experiment under 100 bar of H_2_ in *n*-hexane at room temperature (Table 1). After 48 h, the reaction temperature was increased to 100 °C to promote the aggregation of the Ru-NHC complex into Ru nanoparticles (Figure S1, SI). After 48 h, the desired octahydroindole **2a** was obtained with 10% yield, 87:13 dr, and 95:5 er (entry 1). It was noted that some partially reduced intermediate generated from the first step had not been completely consumed, indicating that the *in situ* generated heterogeneous catalyst was not active enough. Previous reports have shown that metal nanoparticles are more likely to form and are more active when a porous solid support is present in the reaction system.^87−90^ This is because the porous solid acts as a heterogeneous support for the nucleation of the metal nanoparticles and restricts them from their subsequent agglomeration. In addition, the porous support also helps to facilitate the growth of the metal nanoparticles by acting as a carrier and enhancing the efficient substrate-active site interaction at the solid–liquid interfaces.^90^ Therefore, we tested some solid additives as heterogeneous support (entries 2–5) and found that 4 Å MS resulted in the best outcome, leading to 94% isolated yield, 80:20 dr, and 95:5 er (entry 2). Upon further evaluation of the solvents (entries 6–8), we determined hexane to be the optimal choice.

**Table 1:**
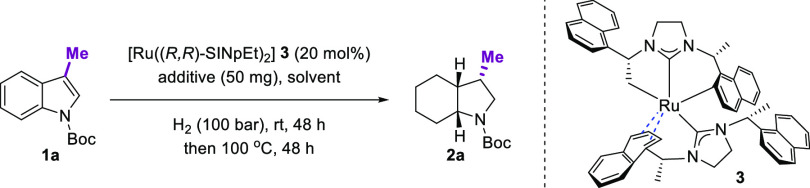
Investigations on Solvents and Additives of Ru-NHC-Catalyzed Asymmetric, Complete Hydrogenation of **1a**^a^

entry	solvent	additive	yield (%)^b^	dr^b^	er^c^
1	*n*-hexane	no	10	87:13	95:5
2	*n*-hexane	4 Å MS	99 [94]^d^	80:20	95:5
3	*n*-hexane	NaCl	46	87:13	94:6
4	*n*-hexane	Celite	15	87:13	94:6
5	*n*-hexane	silica gel	86	78:22	95:5
6	Et_2_O	4 Å MS	98	78:22	82:18
7	THF	4 Å MS	99	80:20	65:35
8	DME	4 Å MS	99	80:20	61:39

a General conditions: **1a** (0.1 mmol), additive (50 mg), and **3** (0.8 mL, 0.025 mmol/mL) in solvent (0.2 mL), and the hydrogenation was performed at 25 °C under 100 bar of H_2_ for 48 h, then at 100 °C under 100 bar of H_2_ for 48 h.

b Determined by GC-FID.

c Determined by HPLC on a chiral stationary phase.

d Isolated yield including all diastereomers.

With the optimized condition established, we investigated the effect of the substituents’ position in the benzene ring (Figure 2). To our delight, substitution across different positions in the benzene ring was efficiently hydrogenated with high yields (**2b**–**2e**). Additionally, the enantioselectivity was preserved when the substituent was remotely situated from the heterocyclic ring in indole (6- or 7-substituted indole, **2c**, **2d**). Nevertheless, in the case of proximal substituents to the heterocyclic ring in indole (5- or 8-position, **2b**, **2e**), the er decreased slightly. Furthermore, when the substituents were at the 5- or 6-position (**2b**, **2c**), diastereoselectivity was adversely affected. Subsequently, we investigated the effects of different substituents at the 6-position on the reaction output. To our delight, the functional group variation of the substituents at the 6-position did not have a noticeable impact on the reactions and resulted in complete hydrogenated products with similar yield, er, and dr. Remarkably, the reaction tolerated both the carbon–oxygen (**2f**) and carbon–silicon (**2g**) bonds. This provides a significant impact in synthetic chemistry, as silyl groups are widely used as synthetic handles for further downstream modifications^91−96^ Furthermore, a phenyl group as a substituent of arene was also reduced under the reaction conditions, giving the fully saturated product (**2h**). Next, we attempted to vary the substituent at position 3 of indoles. We observed that different groups had a slight effect on the yield and diastereoselectivity (**2i**–**2l**). However, a remarkable decline in enantioselectivity was observed in the case of bulky substituents (**2i**, **2j**). Finally, the protecting group of indoles also influenced the reaction, since the er decreased when the Boc protecting group was replaced by the methyl ester (**2m**).

**Figure 2:**
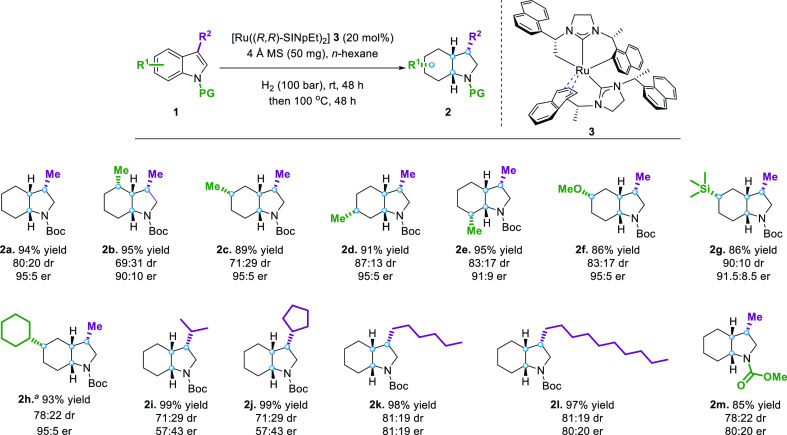
Substrate scope of 3-alkyl-protected indoles. General conditions: **1** (0.1 mmol), 4 Å MS (50 mg), and **3** (0.8 mL, 0.025 mmol/mL) in *n*-hexane (0.2 mL), and the hydrogenation was performed at 25 °C under 100 bar of H_2_ for 48 h and then at 100 °C under 100 bar of H_2_ for 48 h. Yields of isolated products including all diastereomers are given. The major diastereomer is separable. er was determined by chiral HPLC or chiral GC-FID. dr was determined by GC-FID of the crude product mixture. ^*a*^Phenyl-substituent-containing substrate was used.

Subsequently, we examined 2-substituted protected indoles. However, we found the er of the desired product unsatisfactory under the already optimized conditions. Herein, we reoptimized the conditions and found that Et_2_O was the best solvent (Table S2, SI). First of all, we altered the position of the methyl group in the benzene ring (Figure 3, **5b**–**5e**) and found that, except for the 8-substituted indole, which provided a relatively low er (**5e**), the substituents at other positions had a minimal effect on the results of the reaction’s yield and selectivity. Next, we conducted our hydrogenation with different substrates by varying substituents at the 6-position. We observed that the less bulky methoxyl group does not affect the reaction (**5f**). However, when *tert*-butyl (**5g**) and *n*-butyl (**5h**) were present at the 6-position, the enantiomeric ratios of the corresponding products decreased slightly. The bulky *tert*-butyl group also reduced the yield as some of the partially hydrogenated product was not completely consumed. To our delight, the 6,8-disubstituted indole was also suitable for our reaction (**5i**) and yielded the corresponding product with high enantioselectivity and yield. In contrast to 3-substituted indole (**2m**), altering the protecting group had no significant effect on the reaction (**5j**). Finally, the dr improved significantly with increasing bulk at the 2-position; however, the corresponding er dropped dramatically (**5k**).

**Figure 3:**
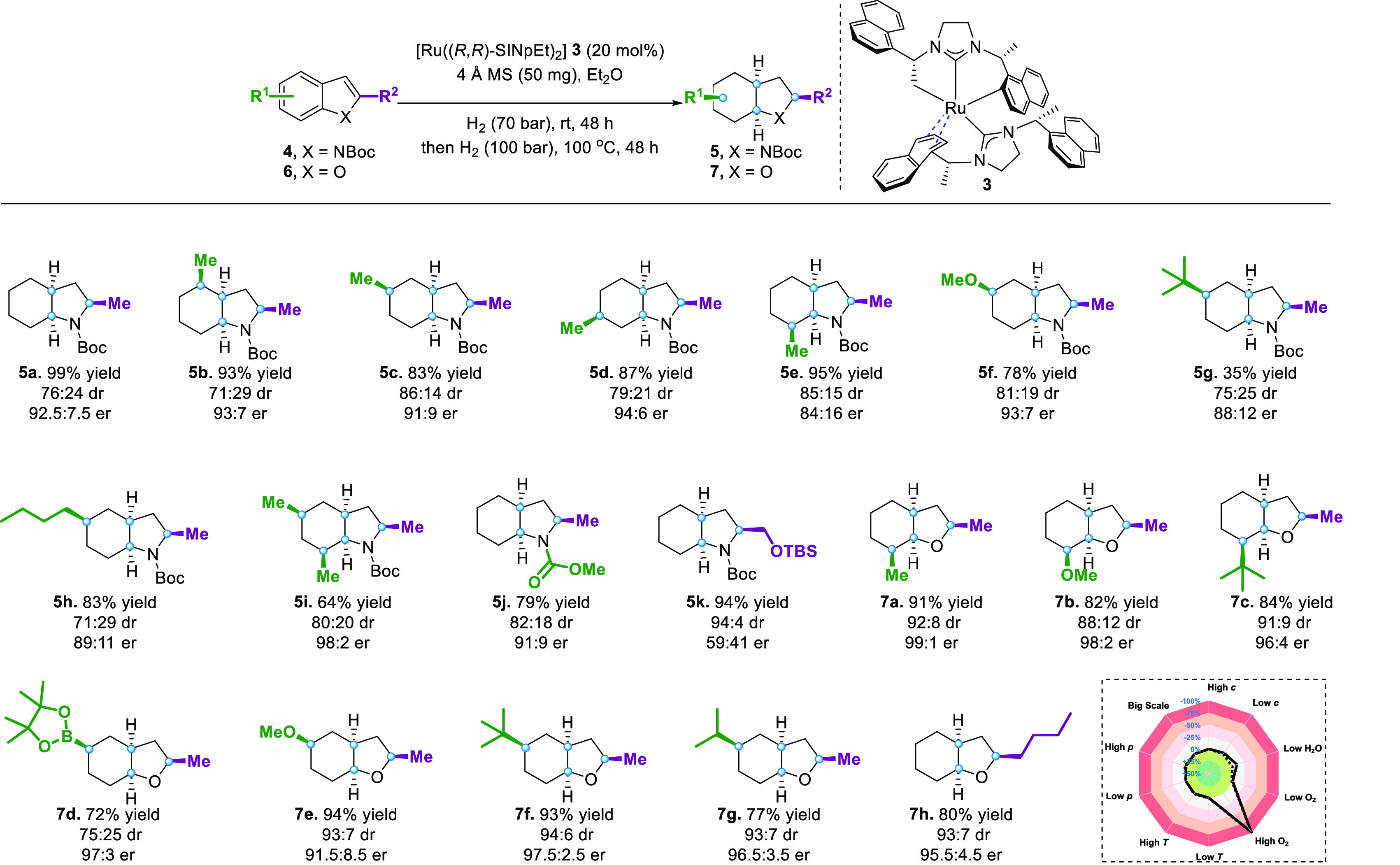
Substrate scope of 2-methyl-protected indoles and 2-alkyl-benzofurans. General conditions: **4** (0.1 mmol), 4 Å MS (50 mg), and **3** (0.8 mL, 0.025 mmol/mL) in Et_2_O (2.0 mL), and the hydrogenation was performed at 25 °C under 70 bar of H_2_ for 48 h and then at 100 °C under 100 bar of H_2_ for 48 h. Yields of isolated products including all diastereomers are given. The major diastereomer is inseparable. er was determined by chiral HPLC or chiral GC-FID. dr was determined by GC-FID of the crude product mixture. Reaction condition-based sensitivity assessment (within box).

We then applied our protocol to the complete hydrogenation of benzofuran to investigate the generality of the proposed dual-catalytic strategy with the successive combination of homo- and heterogeneous reaction conditions. We found that all substrates gave excellent results comparable to our previous report, which used two different catalysts (Figure 3, **7a**–**7h**).^39^ Additionally, the current method also tolerates the carbon–boron bond well (**7d**), thus expanding its application in organic synthesis. To our surprise, the substituents with large steric hindrances, such as *tert*-butyl and isopropyl groups, did not affect the reaction, providing complete conversion of substrates with high yields and excellent dr and er (**7c**, **7f**, **7g**). Variation of the 2-substituent on the furan ring was well tolerated (**7h**). A further comparison between the current protocol with our previous method can be seen in the Supporting Information (Scheme S4, SI). The evaluation of the reaction-condition-based sensitivity screen of the developed protocol revealed a robust reaction (Figure S4, SI).^97^

To demonstrate the utility of our protocol in organic synthesis, we successfully scaled up the reaction to the gram scale and obtained excellent results (Figure 4a). Next, we removed the protecting group of **5a**, performed a coupling reaction with compound **8b**, and successfully obtained the marketed drug perindopril analogue with 50% yield. In addition, compound **2h** could undergo a one-pot reaction that facilitates the switching of protecting groups, leading to **9a**. The single crystal of **9a** (CCDC: 2256901) was also used to determine the absolute conformation of the products. It is well known that secondary amines are excellent organocatalysts. Hence, we applied compound **10c**, obtained from the deprotection of compound **2a**, to catalyze the intermolecular asymmetric 1,4-addition. We found that the secondary amine (**10c**) exhibited good catalytic activity, completing the reaction in only 30 min, with a high yield and enantioselectivity, as well as moderate diastereoselectivity.

**Figure 4:**
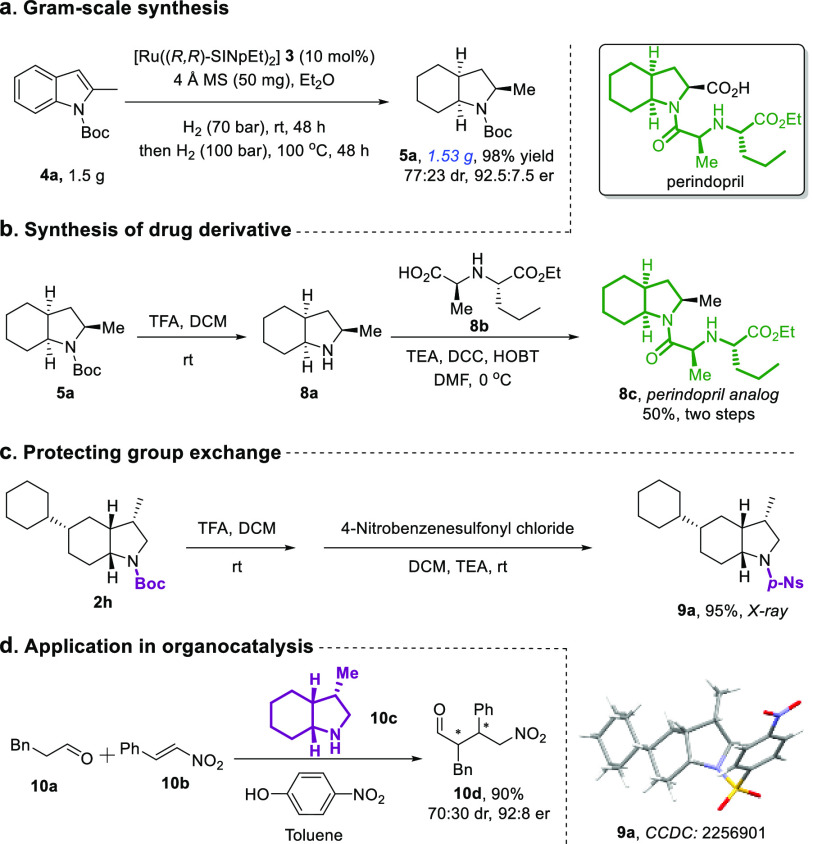
(a) Gram-scale synthesis of **5a**. (b) Diversification of product **5a**, synthesizing drug derivative **8c**. (c) Protecting group exchange. (d) Organocatalytic application.

In conclusion, we have developed a ruthenium-NHC-catalyzed asymmetric, complete hydrogenation of protected indoles and benzofurans, which enabled the installation of up to five newly defined stereocenters. This protocol yielded a broad range of highly valuable octahydroindoles and octahydrobenzofurans in high yields and with good enantioselectivities and diastereoselectivities. Additionally, we rationally designed and implemented a new strategy that one metal complex can play two functions in succession by an *in situ* transformation. Our findings suggest the great potential of the new strategy to be expanded to further substrate classes. We are currently exploring this exciting direction and will disclose our discoveries in due course.

## Supporting Information Available

Experimental details including the synthetic procedures and analytical data for all new compounds, optimization data, gram-scale synthesis, sensitivity screen, mechanistic analysis data including the investigation of the heterogeneous catalyst, and copies of NMR spectra of the compounds (PDF)
