# Supplementary material for: Ru-NHC-Catalyzed Asymmetric, Complete Hydrogenation of Indoles and Benzofurans: One Catalyst with Dual Function
Source: J Am Chem Soc. 2023 Jul 12;145(29):15695–701. doi: 10.1021/jacs.3c04983 (PMC10375535; doi:10.1021/jacs.3c04983)
Supplement: Supplementary file 1 — ja3c04983_si_001.pdf [file ja3c04983_si_001.pdf]

Supporting Information

Ru-NHC Catalyzed Asymmetric, Complete Hydrogenation of Indoles and Benzofurans: One Catalyst with Dual Function

Fuhao Zhang, Himadri Sekhar Sasmal, Constantin G. Daniliuc, Frank Glorius*
AUTHOR ADDRESS: Westfälische Wilhelms-Universität Münster Organisch-Chemisches Institut Corrensstrasse 36, 48149 Münster (Germany)
E-mail: glorius@uni-muenster.de

1. General remarks ... 3
2. Synthesis of Ru-((*R*,*R*)SINpEt)$_2$ catalyst 3. ... 4
3. Preparation of substrates ... 4
3.1. General procedure A (GPA) for preparing *N*-protected indoles: ... 4
3.2. General procedure B (GPB) for preparing *N*-protected indoles: ... 4
3.3. The individual synthetic procedures for the respective compounds as listed below ... 5
4. Investigations of reaction conditions. ... 11
5. General procedure for the asymmetric, complete hydrogenation of protected indoles and benzofurans. ... 12
5.1. General hydrogenation procedure C (GPC) ... 12
5.2. General hydrogenation procedure D (GPD) ... 13
6. Mechanistic considerations and analysis of the stereochemical outcome. ... 45
6.1. Control experiments to investigate the dual role of the catalyst: ... 45
6.2. Procedure for partial hydrogenation of compound 1a. ... 46
6.3. Procedure for partial hydrogenation of compound 4a. ... 47
6.4. Procedure for hydrogenation of compound 2aa ... 48
6.5. Procedure for hydrogenation of compound 5aa ... 49
6.6. Hydrogenation of 5aa with recovered catalyst ... 49
6.7. Mercury poisoning experiments ... 49
6.8. Analysis ... 50
7. Analysis of heterogenous catalyst after catalytical cycle using TEM, HAADF-STEM, and EDX analysis ... 50
8. Low catalyst loading experiments ... 52
9. Preliminary comparison between the current protocol and the previous method with two catalysts. ... 53
9.1. Analysis ... 53
10. Gram scale synthesis of 5a ... 54
11. Elaboration and synthetic application of the products ... 54
12. Sensitivity screen ... 57
13. X-Ray analysis 9a ... 58
14. Spectral data ... 60
15. References ... 123

1. General remarks

Unless otherwise noted, all reactions were carried out under an atmosphere of argon in oven-dried glassware. The employed solvents were either dried by distillation over standard drying agents and stored under argon over molecular sieves (diethyl ether (Na-benzophenone), toluene (CaH_2), THF (Na-benzophenone)) or directly used from a solvent purification system (HPLC grade, dried via an alumina/molecular sieves column under positive argon pressure; n-hexane, dichloromethane). Methanol (3 Å) and dimethylformamide (3 Å) were purchased as dry solvents from commercial suppliers and stored over molecular sieves.

Catalytic hydrogenation reactions were prepared under argon and carried out in Berghof High-Pressure Reactors using hydrogen gas. Reaction temperatures are reported as the temperature of the bath surrounding the vessel unless otherwise stated.

Commercially available chemicals were obtained from Acros Organics, Aldrich Chemical Co., Strem Chemicals, Alfa Aesar, ABCR, Combi-Blocks, Chempur, and TCI Europe and used as received. (*R*)- and (*S*)-1-(1-naphthyl) ethylamine were obtained from BASF (ChiPros®). Unless otherwise noted, no optimizations of the yields were performed for substrate syntheses.

Analytical thin layer chromatography (TLC) was performed on silica gel 60 F254 aluminum plates (Merck). TLC plates were visualized by exposure to short-wave ultraviolet light (254 nm, 366 nm) and were dipped into a solution of $KMnO_4$. Flash chromatography was performed on Acros Organics silica gel (35-70 mesh) under a positive pressure of argon, eluting with the specified solvent system.

GC-MS spectra were recorded on an Agilent Technologies 7890A GC-system with an Agilent 5975C VL MSD or an Agilent 5975 inert Mass Selective Detector (EI) and an HP-5MS column (0.25 mm x 30 m, film: 0.25 µm). Enantiomeric ratios of isolated products were determined with an Agilent Technologies 7890B GC-system and a Supelco *β*-Dex column or on an Astec Chiraldex G-TA column using methods starting at 50 °C, holding this temperature for 5 min and heating with a defined gradient, e.g. 5 °C/min up to a defined temperature, e.g. 100 °C, followed by continued gradient heating, e.g. 0.2 °C/min up to a defined temperature, e.g. 140 °C, heating with a defined gradient, e.g. 5 °C/min up to final temperature, e.g. 200 °C. A standardized notation is used for all samples, which would result in 50_5_5_100_0.2_140_5_200 for this mentioned example. Alternatively, enantiomeric ratios were determined using an Agilent Technologies 1200 Series HPLC with a Daicel Chemical Industries LTD Chiralpak IC3, IA3, or OD-H columns. The signals were detected by UV-absorption spectroscopy (at 210 or 254 nm). Exact ESI mass spectra were recorded on a Bruker Daltonics MicroTof spectrometer. Exact GC-EI mass spectra were recorded on a Thermo Fisher Scientific Trace 1310 GC Exactive Orbitrap, equipped with a Thermo Gold TG-5SILMS (30 m, ID 0.25 mm, 0.25 µm) column. ^{1}H, ^{13}C, and ^{11}B NMR spectra were recorded on a Bruker Avance II300 or Avance II400, AgilentDD2 500, or AgilentDD2 600 in the indicated solvents. Chemical shifts (δ) are given in ppm relative to TMS. The residual solvent signals were used as references and the chemical shifts were converted to the TMS scale ($CDCl_3$: δH = 7.26 ppm, δC = 77.0 ppm).

2. Synthesis of Ru-((*R*,*R*)SINpEt)$_2$ catalyst 3.

The synthesis was performed according to a literature procedure.[1]

Solution of catalyst 3: In an argon-filled glovebox, [Ru(COD)(2-methylally)$_2$] (319 mg, 1 mmol), KO*t*-Bu (225 mg, 2 mmol), (*R*,*R*)-SINpEt·HBF_4 (933 mg, 2 mmol) were added in a 100 ml Schlenk tube. Then under an argon atmosphere, *n*-hexane (40 ml) was added to the Schlenk tube. After stirring at 70 ℃ for 16 h, the reaction was cool down to rt. Catalyst **3** was obtained as a suspension (0.025 mmol/mL).

Solid catalyst 3: In an argon-filled glovebox, [Ru(COD)(2-methylally)$_2$] (319 mg, 1 mmol), KO*t*-Bu (225 mg, 2 mmol), (*R*,*R*)-SINpEt·HBF_4 (933 mg, 2 mmol) were added in a 100 ml Schlenk tube. Then under an argon atmosphere, *n*-hexane (40 ml) was added to the Schlenk tube. After stirring at 70 ℃ for 16 h, the reaction was cool down to rt. The solvent was removed by using an oil pump with a cold trap, then filled the Schlenk tube with argon and stored in an argon-filled glovebox. Catalyst **3** was obtained as a brown solid.

3. Preparation of substrates

3.1. General procedure A (GPA) for preparing *N*-protected indoles:

To a solution of indoles (1 mmol) in MeCN (5 mL) was added Boc_2O (1 mmol) and 4-dimethylamino pyridine (DMAP, 0.01 mmol). The mixture was stirred at 50 ºC for 1 h. The reaction was cooled to room temperature (RT) and the solvent was removed under reduced pressure to afford a crude residue. The crude product was purified by silica gel column chromatography (pentene:ethyl acetate) to give *N*-Boc indoles.

3.2. General procedure B (GPB) for preparing *N*-protected indoles:

To a solution of indoles (1 mmol) in DMF (5 mL) was added NaH (1.2 mmol) at 0 ºC. The mixture was stirred at 0 ºC for 30 min. Then methyl chloroformate (2 mmol) was added into the reaction mixture dropwise and stirred for 2 h. The reaction was then quenched with saturated NH_4Cl aqueous (10 mL) and extracted with ethyl acetate (3 × 10 mL). The combined organic layer was washed with brine, dried over anhydrous Na_2SO_4, and filtered. The solvent was removed under reduced pressure to afford a crude residue. The crude product was purified by silica gel column chromatography (pentene:ethyl acetate) to give *N*-protected indoles.

The following compounds are commercially available.

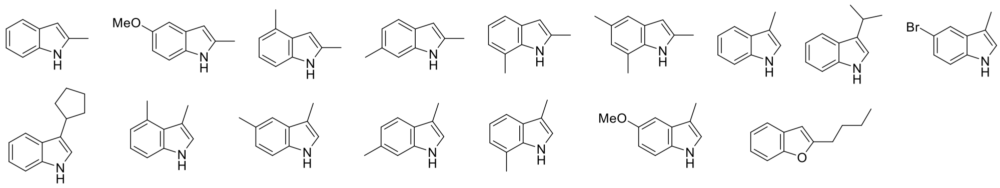

The following compounds have been reported and synthesized according to the literatures.[2]

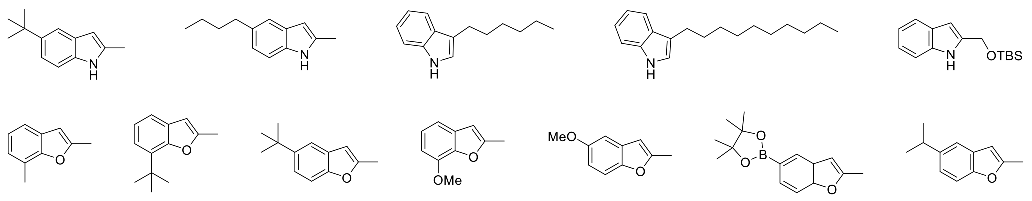

3.3. The individual synthetic procedures for the respective compounds as listed below

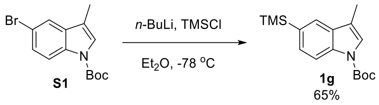

To a solution of **S1** (100 mg, 0.32 mmol, prepared following **GPA**) in anhydrous Et_2O (10 mL) was added *n*-BuLi (0.22 ml, 1.6 M, 0.35 mmol) at –78 °C. The mixture was stirred for 30 min. TMSCl (0.062 mL, 0.48 mmol) was added to the reaction mixture via a syringe, and the reaction was gradually warmed to room temperature (rt) and stirred for 1 h. The reaction was then quenched with saturated NH_4Cl aqueous (10 mL) and extracted with ethyl acetate (3 × 10 mL). The combined organic layer was washed with brine, dried over anhydrous Na_2SO_4, and filtered. The solvent was removed under reduced pressure to afford a crude residue. The crude product was purified by silica gel column chromatography (50:1 = pentene:ethyl acetate) to give **1g** (63 mg, 0.21 mmol, 65%) as a colorless oil.

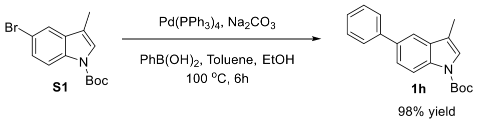

To a solution of **S1** (400 mg, 1.3 mmol) in EtOH-toluene (1:1, 5 mL) was added $Pd(PPh_3)_4$ (75 mg, 0.065 mmol), Na_2CO_3 (3.2 mL, 1.0 M, 3.25 mmol) and $PhB(OH)_2$ (315 mg, 2.6 mmol). The mixture was stirred at 100 °C under argon for 6 h. The reaction was then quenched with saturated NH_4Cl aqueous (10 mL) and extracted with ethyl acetate (3 × 10 mL). The combined organic layer was washed with brine, dried over anhydrous Na_2SO_4, and filtered. The solvent was removed under reduced pressure to afford a crude residue. The crude product was purified by silica gel column chromatography (50:1 = pentene:ethyl acetate) to give **1h** (390 mg, 1.27 mmol, 98%) as a colorless oil.

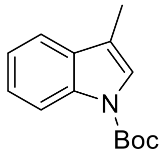

***tert*-butyl 3-methyl-1*H*-indole-1-carboxylate** (**1a**). The title compound was synthesized according to **GPA**. 98% yield. ^{1}H NMR (400 MHz, Chloroform-*d*) δ 8.17 (d, *J* = 6.5 Hz, 1H), 7.56 – 7.51 (m, 1H), 7.40 (s, 1H), 7.38 – 7.32 (m, 1H), 7.31 – 7.25 (m, 1H), 2.31 (d, *J* = 1.3 Hz, 3H), 1.71 (s, 9H). ^{13}C NMR (76 MHz, $CDCl_3$) δ 149.8, 135.4, 131.4, 124.1, 122.7, 122.2, 118.8, 116.3, 115.1, 83.1, 28.2, 9.6.

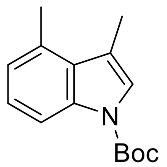

tert-butyl 3,4-dimethyl-1*H*-indole-1-carboxylate (**1b**). The title compound was synthesized according to **GPA**. 96% yield. ^{1}H NMR (400 MHz, Chloroform-*d*) δ 8.00 (d, *J* = 7.7 Hz, 1H), 7.30 (s, 1H), 7.16 (t, *J* = 7.8 Hz, 1H), 6.95 (d, *J* = 7.3 Hz, 1H), 2.67 (s, 3H), 2.44 (d, *J* = 1.2 Hz, 3H), 1.65 (s, 9H). ^{13}C NMR (101 MHz, $CDCl_3$) δ 149.7, 136.1, 131.2, 129.4, 124.1, 124.1, 123.1, 117.2, 113.0, 83.1, 28.2, 19.8, 13.3.

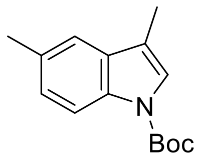

tert-butyl 3,5-dimethyl-1*H*-indole-1-carboxylate (**1c**). The title compound was synthesized according to **GPA**. 96% yield. ^{1}H NMR (400 MHz, Chloroform-*d*) δ 7.98 (s, 1H), 7.31 (s, 1H), 7.28 (s, 1H), 7.13 (d, *J* = 8.4 Hz, 1H), 2.46 (s, 3H), 2.24 (d, *J* = 1.2 Hz, 3H), 1.66 (s, 9H). ^{13}C NMR (101 MHz, $CDCl_3$) δ 149.8, 133.6, 131.7, 131.6, 125.5, 122.8, 118.8, 116.1, 114.7, 82.9, 28.2, 21.3, 9.6.

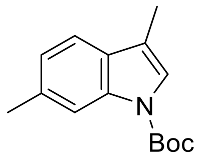

tert-butyl 3,6-dimethyl-1*H*-indole-1-carboxylate (**1d**). The title compound was synthesized according to **GPA**. 90% yield.^{1}H NMR (400 MHz, Chloroform-*d*) δ 8.01 (s, 1H), 7.39 (d, *J* = 7.9 Hz, 1H), 7.13 – 7.08 (m, 1H), 2.51 (s, 3H), 2.27 (d, *J* = 1.3 Hz, 3H), 1.68 (s, 9H). ^{13}C NMR (101 MHz, $CDCl_3$) δ 149.9, 135.7, 134.2, 129.2, 123.7, 122.1, 118.5, 116.3, 115.4, 83.0, 28.2, 21.9, 9.7.

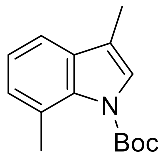

tert-butyl 3,7-dimethyl-1*H*-indole-1-carboxylate (**1e**). The title compound was synthesized according to **GPA**. 89% yield. ^{1}H NMR (400 MHz, Chloroform-*d*) δ 7.34 – 7.30 (m, 1H), 7.29 (t, *J* = 1.3 Hz, 1H), 7.17 (t, *J* = 7.5 Hz, 1H), 7.11 (d, *J* = 7.3 Hz, 1H), 2.64 (s, 3H), 2.24 (d, *J* = 1.2 Hz, 3H), 1.63 (s, 9H). ^{13}C NMR (101 MHz, $CDCl_3$) δ 149.7, 135.1, 132.8, 127.6, 125.5, 125.1, 122.8, 116.4, 116.1, 82.8, 28.1, 22.2, 9.6.

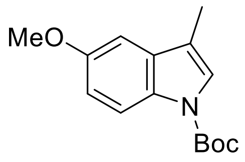

***tert*-butyl 5-methoxy-3-methyl-1*H*-indole-1-carboxylate** (**1f**). The title compound was synthesized according to **GPA**. 99% yield. ^{1}H NMR (400 MHz, Chloroform-*d*) δ 7.99 (s, 1H), 7.33 (s, 1H), 6.95 – 6.89 (m, 2H), 3.87 (s, 3H), 2.24 (d, *J* = 1.2 Hz, 3H), 1.65 (s, 9H). ^{13}C NMR (101 MHz, $CDCl_3$) δ 155.8, 149.8, 132.3, 129.9, 123.5, 116.1, 115.9, 112.7, 101.8, 83.0, 55.8, 28.3, 9.7.

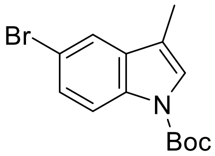

***tert*-butyl 5-bromo-3-methyl-1*H*-indole-1-carboxylate** (**S1**). The title compound was synthesized according to **GPA**. 97% yield. ^{1}H NMR (400 MHz, Chloroform-*d*) δ 7.98 (s, 1H), 7.61 (d, *J* = 1.8 Hz, 1H), 7.43 – 7.30 (m, 2H), 2.23 (d, *J* = 1.2 Hz, 3H), 1.65 (s, 9H). ^{13}C NMR (101 MHz, $CDCl_3$) δ 149.4, 134.2, 133.2, 126.9, 123.9, 121.7, 116.6, 115.7, 115.6, 83.6, 28.2, 9.5.

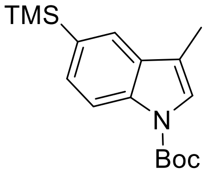

***tert*-butyl 3-methyl-5-(trimethylsilyl)-1*H*-indole-1-carboxylate** (**1g**). 65% yield. ^{1}H NMR (400 MHz, Chloroform-*d*) δ 8.09 (s, 1H), 7.64 (s, 1H), 7.49 – 7.44 (m, 1H), 7.34 (s, 1H), 2.29 (d, *J* = 1.1 Hz, 3H), 1.66 (s, 9H), 0.32 (s, 9H).

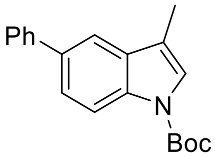

***tert*-butyl 2-methyl-5-phenyl-1*H*-indole-1-carboxylate** (**1h**). 98% yield. ^{1}H NMR (400 MHz, Chloroform-*d*) δ 8.08 (s, 1H), 7.66 – 7.55 (m, 3H), 7.48 (dd, *J* = 8.6, 1.7 Hz, 1H), 7.38 (t, *J* = 7.6 Hz, 2H), 7.33 – 7.21 (m, 2H), 2.23 (d, *J* = 1.2 Hz, 3H), 1.60 (s, 9H). ^{13}C NMR (101 MHz, $CDCl_3$) δ 149.7, 141.8, 135.7, 134.9, 131.9, 128.7, 127.4, 126.8, 123.7, 123.4, 117.4, 116.6, 115.3, 83.3, 28.2, 9.6.

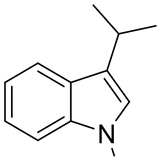

***tert*-butyl 3-isopropyl-1*H*-indole-1-carboxylate** (**1i**). The title compound was synthesized according to **GPA**. 92% yield. ^{1}H NMR (400 MHz, Chloroform-*d*) δ 8.12 (s, 1H), 7.62 – 7.54 (m, 1H), 7.37 – 7.27 (m, 2H), 7.23 (td, *J* = 7.6, 1.1 Hz, 1H), 3.13 (pd, *J* = 6.8, 0.9 Hz, 1H), 1.67 (s, 9H), 1.35 (d, *J* = 6.9 Hz, 6H). ^{13}C NMR (101 MHz, $CDCl_3$) δ 151.0, 135.7, 130.1, 128.1, 124.1, 122.1, 120.5, 119.4, 115.3, 83.3, 28.2, 25.2, 22.6.

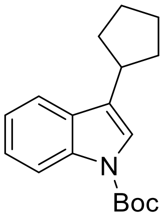

<u>*tert*-butyl 3-cyclopentyl-1*H*-indole-1-carboxylate</u> (**1j**). The title compound was synthesized according to **GPA**. 87% yield. ^{1}H NMR (400 MHz, Chloroform-*d*) δ 8.11 (s, 1H), 7.61 – 7.54 (m, 1H), 7.38 – 7.27 (m, 2H), 7.22 (td, *J* = 7.7, 1.1 Hz, 1H), 3.19 (p, *J* = 7.7 Hz, 1H), 2.21 – 2.10 (m, 2H), 1.87 – 1.68 (m, 6H), 1.67 (s, 9H). ^{13}C NMR (101 MHz, $CDCl_3$) δ 150.0, 135.9, 130.7, 125.6, 124.1, 122.1, 120.7, 119.6, 115.2, 83.2, 36.5, 32.6, 28.2, 25.2.

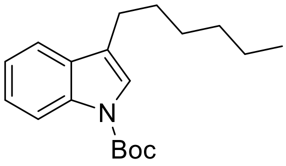

<u>*tert*-butyl 3-hexyl-1*H*-indole-1-carboxylate</u> (**1k**). The title compound was synthesized according to **GPA**. 88% yield. ^{1}H NMR (400 MHz, Chloroform-*d*) δ 8.11 (d, *J* = 6.6 Hz, 1H), 7.56 – 7.45 (m, 1H), 7.40 – 7.27 (m, 2H), 7.27 – 7.18 (m, 1H), 2.76 – 2.55 (m, 2H), 1.67 (s, 11H), 1.46 – 1.28 (m, 6H), 0.97 – 0.87 (m, 3H). ^{13}C NMR (101 MHz, $CDCl_3$) δ 149.9, 135.5, 130.9, 124.1, 122.2, 121.5, 119.0, 115.2, 83.2, 31.7, 29.3, 29.2, 28.2, 24.9, 22.6, 14.1.

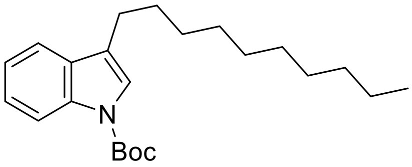

<u>*tert*-butyl 3-decyl-1*H*-indole-1-carboxylate</u> (**1l**). The title compound was synthesized according to **GPA**. 94% yield. ^{1}H NMR (400 MHz, Chloroform-*d*) δ 8.11 (d, *J* = 6.3 Hz, 1H), 7.52 (d, *J* = 7.3 Hz, 1H), 7.40 – 7.27 (m, 2H), 7.27 – 7.18 (m, 1H), 2.72 – 2.63 (m, 2H), 1.72 – 1.67 (m, 11H), 1.43 – 1.24 (m, 14H), 0.93 – 0.86 (m, 3H). ^{13}C NMR (101 MHz, $CDCl_3$) δ 149.4, 135.3, 134.2, 130.9, 124.1, 122.2, 121.5, 119.0, 115.2, 83.2, 31.9, 29.62, 29.6, 29.59, 29.5, 29.32, 29.3, 28.2, 24.9, 22.7, 14.1.

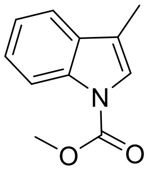

<u>methyl 3-methyl-1*H*-indole-1-carboxylate</u> (**1m**). The title compound was synthesized according to **GPB**. 83% yield. ^{1}H NMR (400 MHz, Chloroform-*d*) δ 8.07 (s, 1H), 7.43 (d, *J* = 7.7 Hz, 1H), 7.31 – 7.23 (m, 2H), 7.21 – 7.17 (m, 1H), 3.94 (s, 3H), 2.20 (d, *J* = 1.2 Hz, 3H). ^{13}C NMR (101 MHz, $CDCl_3$) δ 151.4, 135.5, 131.4, 124.5, 122.6, 122.3, 118.9, 117.2, 115.0, 53.6, 9.6.

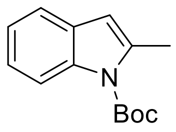

***tert*-butyl 2-methyl-1*H*-indole-1-carboxylate** (4**a**). The title compound was synthesized according to **GPA**. 98% yield. ^{1}H NMR (400 MHz, Chloroform-*d*) δ 8.22 – 8.13 (m, 1H), 7.53 – 7.45 (m, 1H), 7.33 – 7.20 (m, 2H), 6.42 – 6.29 (m, 1H), 2.65 (d, *J* = 1.1 Hz, 3H), 1.74 (s, 9H). ^{13}C NMR (76 MHz, $CDCl_3$) δ 150.6, 137.7, 136.4, 129.3, 123.0, 122.5, 119.4, 115.4, 107.9, 83.5, 28.2, 17.1.

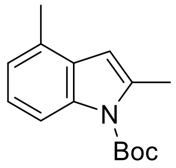

***tert*-butyl 2,4-dimethyl-1*H*-indole-1-carboxylate** (**4b**). The title compound was synthesized according to **GPA**. 90% yield. ^{1}H NMR (400 MHz, Chloroform-*d*) δ 7.93 (d, *J* = 8.3 Hz, 1H), 7.17 – 7.05 (m, 1H), 6.99 (d, *J* = 7.3 Hz, 1H), 6.35 (s, 1H), 2.60 (d, *J* = 0.9 Hz, 3H), 2.47 (s, 3H), 1.68 (s, 9H). ^{13}C NMR (101 MHz, Chloroform-*d*) δ 150.7, 137.2, 136.2, 128.8, 128.8, 123.1, 123.0, 113.0, 106.3, 83.5, 28.3, 18.4, 17.2.

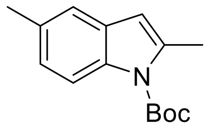

***tert*-butyl 2,5-dimethyl-1*H*-indole-1-carboxylate** (**4c**). The title compound was synthesized according to **GPA**. 95% yield. ^{1}H NMR (400 MHz, Chloroform-*d*) δ 8.15 (d, *J* = 7.9 Hz, 1H), 7.57 (s, 1H), 7.55 – 7.52 (m, 1H), 7.35 – 7.31 (m, 1H), 7.27 – 7.23 (m, 1H), 3.85 – 3.60 (m, 6H), 1.67 (s, 9H). ^{13}C NMR (101 MHz, $CDCl_3$) δ 171.5, 149.6, 135.4, 130.0, 124.5, 124.4, 122.6, 119.0, 115.3, 113.1, 83.6, 52.1, 30.9, 28.2.

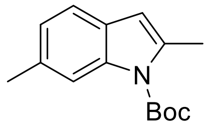

***tert*-butyl 2,6-dimethyl-1*H*-indole-1-carboxylate** (**4d**). The title compound was synthesized according to **GPA**. 95% yield.^{1}H NMR (400 MHz, Chloroform-*d*) δ 8.02 – 7.95 (m, 1H), 7.30 (d, *J* = 7.9 Hz, 1H), 7.07 – 6.95 (m, 1H), 6.25 (s, 1H), 2.60 – 2.53 (m, 3H), 2.46 (s, 3H), 1.68 (s, 9H). ^{13}C NMR (101 MHz, $CDCl_3$) δ 150.8, 136.9, 136.9, 132.9, 127.0, 123.9, 119.0, 115.8, 107.8, 83.4, 28.3, 22.0, 17.2.

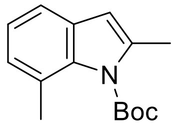

***tert*-butyl 2,7-dimethyl-1*H*-indole-1-carboxylate** (**4e**). The title compound was synthesized according to **GPA**. 54% yield. ^{1}H NMR (400 MHz, Chloroform-*d*) δ 7.29 (d, *J* = 7.5 Hz, 1H), 7.09 (t, *J* = 7.5 Hz, 1H), 7.00 (d, *J* = 7.3 Hz, 1H), 2.50 (d, *J* = 1.0 Hz, 3H), 2.46 (s, 3H), 1.65 (s, 9H). ^{13}C NMR (101 MHz, $CDCl_3$) δ 150.6, 137.0, 135.7, 130.1, 125.9, 123.9, 122.5, 117.3, 106.4, 83.6, 27.9, 20.8, 15.9.

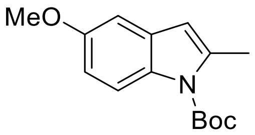

***tert*-butyl 5-methoxy-2-methyl-1*H*-indole-1-carboxylate** (**4f**). The title compound was synthesized according to **GPA**. 92% yield. ^{1}H NMR (400 MHz, Chloroform-*d*) δ 7.98 (d, *J* = 9.0 Hz, 1H), 6.91 (d, *J* = 2.5 Hz, 1H), 6.83 (dd, *J* = 9.0, 2.6 Hz, 1H), 6.24 (s, 1H), 3.84 (s, 3H), 2.57 (s, 3H), 1.67 (s, 9H). ^{13}C NMR (101 MHz, Chloroform-*d*) δ 155.7, 150.6, 138.5, 131.1, 130.2, 116.2, 111.4, 107.8, 102.4, 83.4, 55.6, 28.3, 17.2.

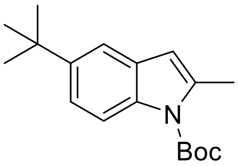

***tert*-butyl 5-(tert-butyl)-2-methyl-1*H*-indole-1-carboxylate** (**4g**). The title compound was synthesized according to **GPA**. 88% yield. ^{1}H NMR (400 MHz, Chloroform-*d*) δ 7.99 (d, *J* = 8.8 Hz, 1H), 7.43 (d, *J* = 1.9 Hz, 1H), 7.29 (dd, *J* = 8.8, 2.0 Hz, 1H), 6.28 (s, 1H), 2.59 (d, *J* = 0.9 Hz, 3H), 1.68 (s, 9H), 1.37 (s, 9H). ^{13}C NMR (101 MHz, $CDCl_3$) δ 150.7, 145.6, 137.9, 134.4, 129.2, 120.9, 115.7, 114.9, 108.1, 83.3, 34.5, 31.7, 28.3, 17.0.

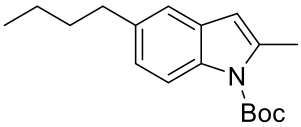

***tert*-butyl 5-butyl-2-methyl-1*H*-indole-1-carboxylate** (**4h**). The title compound was synthesized according to **GPA**. 95% yield. ^{1}H NMR (400 MHz, Chloroform-*d*) δ 7.98 (d, *J* = 8.5 Hz, 1H), 7.25 – 7.20 (m, 1H), 7.05 (dd, *J* = 8.5, 1.6 Hz, 1H), 6.25 (s, 1H), 2.73 – 2.63 (m, 2H), 2.58 (d, *J* = 0.9 Hz, 3H), 1.68 (s, 9H), 1.66 – 1.58 (m, 2H), 1.36 (dq, *J* = 14.6, 7.3 Hz, 2H), 0.93 (t, *J* = 7.3 Hz, 3H). ^{13}C NMR (101 MHz, $CDCl_3$) δ 150.7, 137.8, 137.2, 134.8, 129.5, 123.8, 118.9, 115.1, 107.8, 83.4, 35.4, 34.1, 28.3, 22.3, 17.1, 14.0.

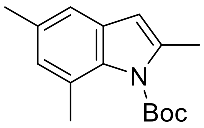

***tert*-butyl 2,5,7-trimethyl-1*H*-indole-1-carboxylate** (**4i**). The title compound was synthesized according to **GPA**. 65% yield. ^{1}H NMR (400 MHz, Chloroform-*d*) δ 7.07 (s, 1H), 6.83 (s, 1H), 6.24 – 6.16 (m, 1H), 2.48 (d, *J* = 1.0 Hz, 3H), 2.42 (s, 3H), 2.37 (s, 3H), 1.64 (s, 9H). ^{13}C NMR (101 MHz, $CDCl_3$) δ 150.7, 137.2, 134.0, 131.9, 130.5, 127.4, 123.6, 117.3, 106.3, 83.4, 28.0, 21.0, 20.7, 16.0.

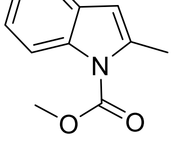

methyl 2-methyl-1*H*-indole-1-carboxylate (**4j**). The title compound was synthesized according to **GPB**. 79% yield. ^{1}H NMR (400 MHz, Chloroform-*d*) δ 8.12 (d, *J* = 8.1 Hz, 1H), 7.47 (d, *J* = 7.3 Hz, 1H), 7.30 – 7.21 (m,

2H), 6.37 (s, 1H), 4.07 (s, 3H), 2.64 (s, 3H). ^{13}C NMR (101 MHz, $CDCl_3$) δ 152.6, 137.7, 136.3, 129.5, 123.3, 122.9, 119.6, 115.4, 108.5, 53.3, 16.7.

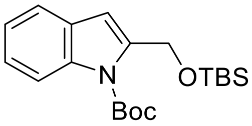

***tert*-butyl 2-(((*tert*-butyldimethylsilyl)oxy)methyl)-1*H*-indole-1-carboxylate** (**4k**). The title compound was synthesized according to **GPA**. 93% yield. ^{1}H NMR (400 MHz, Chloroform-*d*) δ 8.10 (d, *J* = 8.0 Hz, 1H), 7.59 – 7.46 (m, 1H), 7.26 – 7.16 (m, 2H), 6.66 (s, 1H), 5.04 (d, *J* = 1.3 Hz, 2H), 1.68 (s, 9H), 0.98 (s, 9H), 0.14 (s, 6H). ^{13}C NMR (101 MHz, $CDCl_3$) δ 150.4, 141.8, 136.6, 129.4, 123.4, 122.7, 120.2, 115.4, 106.5, 83.9, 61.1, 28.2, 25.9, 18.4, -5.3.

4. Investigations of reaction conditions.

Table **S1** Investigations on solvents and additives of Ru-NHC catalyzed asymmetric, complete hydrogenation of **1a**.[a]

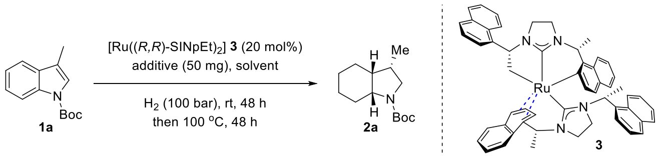

entry	solvent	additive	yield (%)[b]	dr[b]	er[c]
1	*n*-hexane	no	10	87:13	95:5
2	*n*-hexane	4 Å MS	99 [94][d]	80:20	95:5
3	*n*-hexane	NaCl	46	87:13	94:6
4	*n*-hexane	Celite	15	87:13	94:6
5	*n*-hexane	Silica gel	86	78:22	95:5
6	Et_2O	4 Å MS	98	78:22	82:18
7	THF	4 Å MS	99	80:20	65:35
8	DME	4 Å MS	99	80:20	61:39

[a] General conditions: **1a** (0.1 mmol), additive (50 mg), and **3** (0.8 ml, 0.025 mmol/mL) in solvent (0.2 ml), and the hydrogenation was performed at 25 °C under 100 bar H_2 for 48 h, then at 100 °C under 100 bar H_2 for 48 h. [b] Determined by GC-FID. [c] Determined by HPLC on a chiral stationary phase. [d] Isolated yield including all diastereomers.

To a 4 mL glass vial (screwcap with septum) equipped with a stir bar, the substrate (0.1 mmol, 1.00 equiv.), pulverized 4 Å molecular sieve (50 mg), and the vial carefully evacuated. Under an argon atmosphere, indicated solvent (0.2 mL) and the preformed Ru((*R*,*R*)-SINpEt)$_2$ catalyst **3** (0.025 mmol/mL) as stock suspension (0.8 mL, 20 mol%) in *n*-hexane was added. The glass vial was placed in a 150 mL stainless steel autoclave under an argon atmosphere. The autoclave was pressurized and depressurized with hydrogen gas three times before the pressure was set to 100 bar. The reaction mixture was stirred at rt for 48 h. After this period, the reaction temperature was increased to 100 °C and the reaction mixture continued to stir for 48 h. Then the autoclave was cooled down to rt. It was carefully depressurized. Yield and d.r. were determined by GC-FID analysis of the crude mixture. e.r. was determined by HPLC on a chiral stationary phase.

Table **S2**. Investigations on solvents and additives of Ru-NHC catalyzed asymmetric, complete hydrogenation of **4a**.[a]

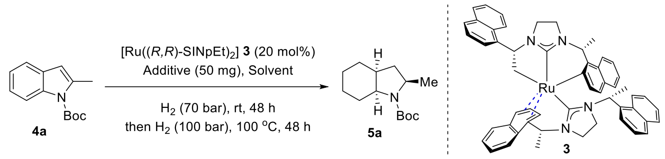

entry	solvent	additive	yield (%)[b]	dr[b]	er[c]
1	*n*-hexane	no	86	78:22	90:10
2	*n*-hexane	4 Å MS	95	76:24	89:11
3	Et_2O	4 Å MS	99 [98][d]	76:24	92.5:7.5
4	THF	4 Å MS	93	78:22	92:8
5	toluene	4 Å MS	90	77:23	91:9
6	DME	4 Å MS	78	76:24	89:11
7	Et_2O	NaCl	89	80:20	92:8
8	Et_2O	Na_2SO_4	99	79:21	92:8
9	Et_2O	$MgSO_4$	trace	76:24	-----
10	Et_2O	Na_2CO_3	28	80:20	91:9

[a] General conditions: **4a** (0.1 mmol), additive (50 mg), and **3** (0.8 ml, 0.025 mmol/mL) in solvent (2.0 ml), and the hydrogenation was performed at 25 °C under 70 bar H_2 for 48 h, then at 100 °C under 100 bar H_2 for 48 h. [b] Determined by GC-FID. [c] Determined by GC-FID on a chiral stationary phase. [d] Isolated yield including all diastereomers.

To an 8 mL glass vial (screwcap with septum) equipped with a stir bar, the substrate (0.1 mmol, 1.00 equiv.), pulverized 4 Å molecular sieve (50 mg), and the vial carefully evacuated. Under an argon atmosphere, indicated solvent (2 mL) and the preformed Ru((*R*,*R*)-SINpEt)$_2$ catalyst **3** (0.025 mmol/mL) as stock suspension (0.8 mL, 20 mol%) in *n*-hexane was added. The glass vial was placed in a 150 mL stainless steel autoclave under an argon atmosphere. The autoclave was pressurized and depressurized with hydrogen gas three times before the pressure was set to 70 bar. The reaction mixture was stirred at 25 °C for 48 h. After this period, the hydrogen pressure was increased to 100 bar and the temperature increased to 100 °C and the reaction mixture continued to stir for 48 h. Then the autoclave was cooled down to rt, it was carefully depressurized. Yield and d.r. were determined by GC-FID analysis of the crude mixture. e.r. was determined by GC-FID on a chiral stationary phase.

5. General procedure for the asymmetric, complete hydrogenation of protected indoles and benzofurans.

5.1. General hydrogenation procedure C (GPC)

To a 4 mL glass vial (screwcap with septum) equipped with a stir bar, the substrate (0.1 mmol, 1.00 equiv.), pulverized 4 Å molecular sieve (50 mg), and the vial carefully evacuated. Under an argon atmosphere, *n*-hexane (0.2 mL) and the preformed Ru((*R*,*R*)-SINpEt)$_2$ catalyst **3** (0.025 mmol/mL) as stock suspension (0.8 mL, 20 mol%) in *n*-hexane were added. The glass vial was placed in a 150 mL stainless steel autoclave under an argon atmosphere. The autoclave was pressurized and depressurized with hydrogen gas three times before the pressure was set to 100 bar. The reaction mixture was stirred at rt for 48 h. After this period, the reaction temperature was increased to 100 °C and the reaction mixture continued to stir for 48 h. Then the autoclave was cooled down to rt, it was carefully depressurized, and the crude product was purified by silica gel column chromatography (pentene:ethyl acetate) to give the pure product. d.r. was determined by GC-FID analysis of the crude mixture.

5.2. General hydrogenation procedure D (GPD)

To an 8 mL glass vial (screwcap with septum) equipped with a stir bar, the substrate (0.1 mmol, 1.00 equiv.), pulverized 4 Å molecular sieve (50 mg), and the vial carefully evacuated. Under an argon atmosphere, Et_2O (2 mL) and the preformed Ru((*R*,*R*)-SINpEt)$_2$ catalyst **3** (0.025 mmol/mL) as stock suspension (0.8 mL, 20 mol%) in *n*-hexane was added. The glass vial was placed in a 150 mL stainless steel autoclave under an argon atmosphere. The autoclave was pressurized and depressurized with hydrogen gas three times before the pressure was set to 70 bar. The reaction mixture was stirred at 25 °C for 48 h. After this period, the hydrogen pressure was increased to 100 bar and the temperature was increased to 100 °C and the reaction mixture continued to stir for 48 h. Then the autoclave was cooled down to rt, it was carefully depressurized, and the crude product was purified by silica gel column chromatography (pentene:ethyl acetate) to give the pure product. d.r. was determined by GC-FID analysis of the crude mixture.

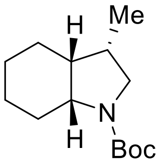

***tert*-butyl (3*S*,3a*R*,7a*R*)-3-methyloctahydro-1*H*-indole-1-carboxylate** (**2a**). The title compound was synthesized according to **GPC**. The product was isolated by column chromatography using a gradient of pentene:ethyl acetate (50:1) and obtained as a colorless oil (22.5 mg, 0.094 mmol, 94%, 80:20 dr, 95:5 er). The major diastereomer could be separated by flash chromatography. Data for the major diastereomer: ^{1}H NMR (400 MHz, Chloroform-*d*) δ 3.68 – 3.54 (m, 2H), 3.00 (t, *J* = 10.5 Hz, 1H), 2.65 – 2.37 (m, 1H), 2.18 – 2.05 (m, 1H), 2.00 – 1.84 (m, 1H), 1.70 – 1.62 (m, 1H), 1.57 – 1.49 (m, 1H), 1.45 (s, 11H), 1.28 – 1.04 (m, 3H), 0.92 (d, *J* = 6.9 Hz, 3H). ^{13}C NMR (101 MHz, $CDCl_3$) δ 155.7, 78.8, 58.3, 52.8, 41.6, 35.0, 28.6, 27.0, 24.4, 22.3, 20.7, 12.5. $[\alpha]_D^{20}$: – 68.3 (*c* = 1.0, CH_2Cl_2). HRMS (ESI) Calcd for $C_{14}H_{25}NNaO_2$ $[M+Na]^+$: 262.1778, found: 262.1777. HPLC on Chiralpak IC-3 column; hexane:isopropanol = 99:1; flow rate = 0.5 mL/min; UV detection at 210 nm; t_R = 17.30 min (minor), t_R = 17.94 min (major).

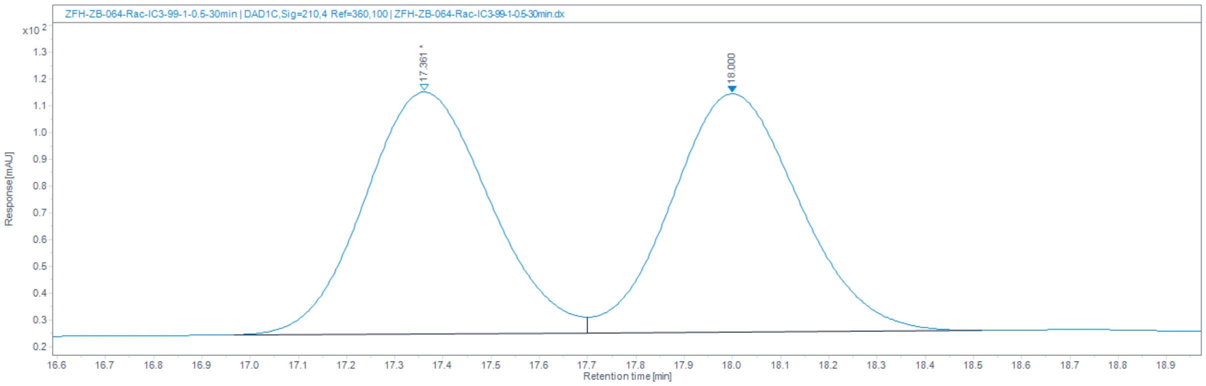

#	Name	Signal description	RT (min)	Area (mAU·s)	Area%	Height (mAU)	Height%	Amount	Concentration	Start time (min)	End time (min)
1		DAD1C,Sig=210,4 Ref=360,100	17.361	1625.530	49.816	90.396	50.37			16.967	17.699
2		DAD1C,Sig=210,4 Ref=360,100	18.000	1637.518	50.184	89.057	49.63			17.699	18.518

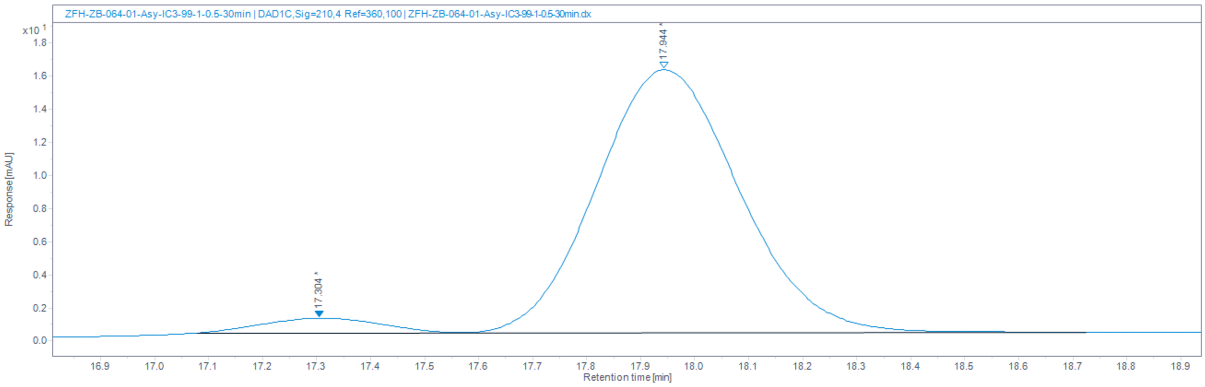

#	Name	Signal description	RT (min)	Area (mAU·s)	Area%	Height (mAU)	Height%	Amount	Concentration	Start time (min)	End time (min)
1		DAD1C,Sig=210,4 Ref=360,100	17.304	13.527	4.434	0.898	5.35			17.079	17.561
2		DAD1C,Sig=210,4 Ref=360,100	17.944	291.575	95.566	15.882	94.65			17.561	18.725

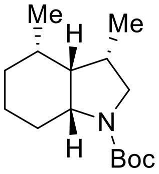

<u>*tert*-butyl (3*S*,3a*R*,4*S*,7a*R*)-3,4-dimethyloctahydro-1*H*-indole-1-carboxylate</u> (**2b**). The title compound was synthesized according to **GPC**. The product was isolated by column chromatography using a gradient of pentene:ethyl acetate (50:1) and obtained as a colorless oil (24 mg, 0.095 mmol, 95%, 69:31 dr, 90:10 er). The major diastereomer could be separated by flash chromatography. Data for the major diastereomer: ^{1}H NMR (400 MHz, Chloroform-*d*) δ 3.75 – 3.34 (m, 2H), 3.21 – 2.80 (m, 1H), 2.40 – 2.26 (m, 1H), 2.24 – 2.10 (m, 1H), 1.98 – 1.53 (m, 3H), 1.49 – 1.38 (m, 11H), 1.28 – 1.14 (m, 5H), 1.09 – 0.94 (m, 4H). ^{13}C NMR (101 MHz, $CDCl_3$) δ 155.1, 155.0, 78.7, 78.6, 78.6, 57.7, 55.0, 54.5, 45.1, 44.4, 33.9, 33.0, 32.6, 32.5, 30.5, 30.4, 30.3, 29.9, 28.6, 23.8, 23.6, 21.1, 21.0, 20.4, 20.3, 18.9. $[\alpha]_D^{20}$: –26.0 (c = 0.8, CH_2Cl_2). HRMS (ESI) Calcd for $C_{15}H_{27}NNaO_2$ $[M+Na]^+$: 276.1934, found: 276.1933. Chiral GC-FID (50_5_5_100_0.2_140_5_200, *β*-Dex-120): t_1 = 223.10 min (minor enantiomer), t_2 = 223.55 min (major enantiomer).

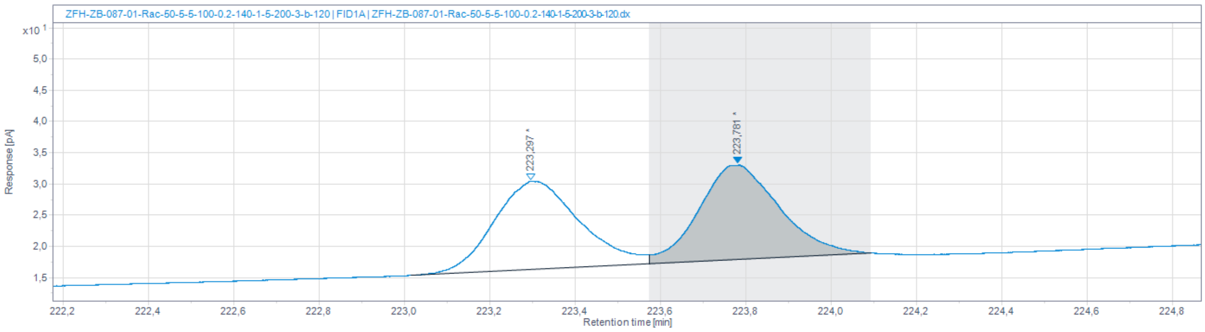

#	Name	Signal description	RT (min)	Area (pA·s)	Area%	Height (pA)	Height%	Amount	Concentration	Start time (min)	End time (min)
1		FID1A	223,297	196,758	49,706	14,154	48,32			223,017	223,575
2		FID1A	223,781	199,084	50,294	15,137	51,68			223,575	224,094

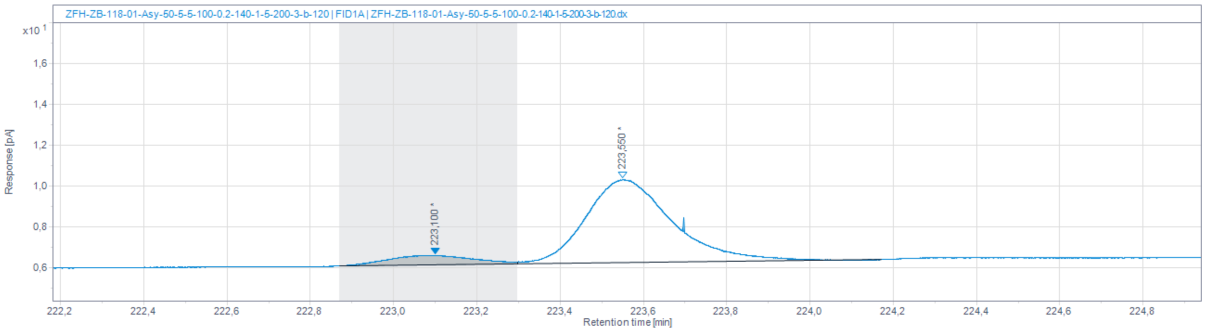

#	Name	Signal description	RT (min)	Area (pA·s)	Area%	Height (pA)	Height%	Amount	Concentration	Start time (min)	End time (min)
1		FID1A	223,100	6,564	10,048	0,462	10,21			222,870	223,298
2		FID1A	223,550	58,761	89,952	4,059	89,79			223,298	224,170

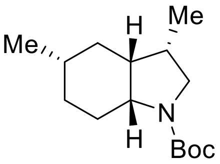

***tert*-butyl (3*S*,3a*R*,5*S*,7a*R*)-3,5-dimethyloctahydro-1*H*-indole-1-carboxylate** (**2c**). The title compound was synthesized according to **GPC**. The product was isolated by column chromatography using a gradient of pentene:ethyl acetate (50:1) and obtained as a colorless oil (22.5 mg, 0.089 mmol, 89%, 71:29 dr, 95:5 er). The major diastereomer could be separated by flash chromatography. Data for the major diastereomer: ^{1}H NMR (400 MHz, Chloroform-*d*) δ 3.73 – 3.47 (m, 2H), 2.98 (t, J = 10.9 Hz, 1H), 2.77 – 2.58 (m, 1H), 2.20 – 2.03 (m, 1H), 2.00 – 1.88 (m, 1H), 1.54 – 1.25 (m, 15H), 0.90 (t, J = 7.1 Hz, 6H). ^{13}C NMR (101 MHz, $CDCl_3$) δ 155.8, 78.8, 57.9, 54.9, 52.7, 42.2, 34.8, 31.1, 31.0, 29.2, 28.6, 22.9, 11.9. $[\alpha]_D^{20}$: –58.5 (c = 1.0, CH_2Cl_2). HRMS (ESI) Calcd for $C_{15}H_{27}NNaO_2$ $[M+Na]^+$: 276.1934, found: 276.1934. HPLC on Chiralpak IC-3 column; hexane:isopropanol = 99:1; flow rate = 0.5 mL/min; UV detection at 210 nm; t_R = 15.92 min (minor), t_R = 16.75 min (major).

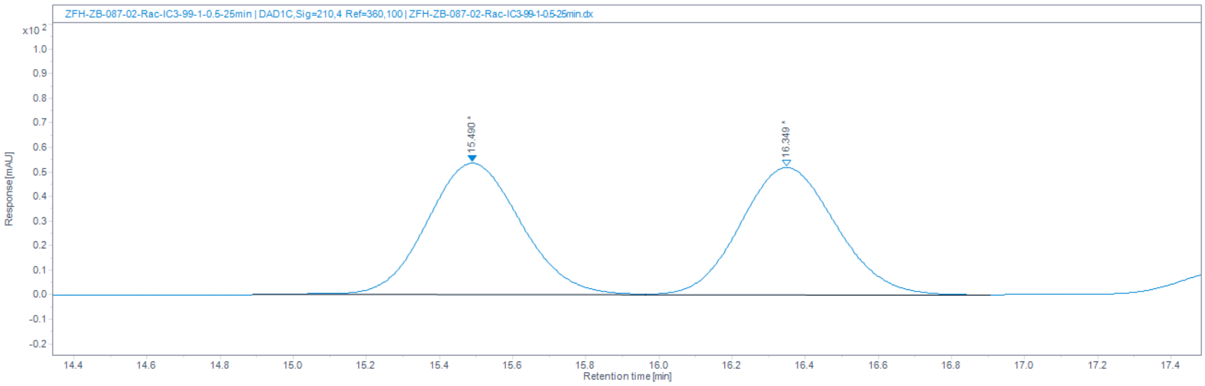

#	Name	Signal description	RT (min)	Area (mAU·s)	Area%	Height (mAU)	Height%	Amount	Concentration	Start time (min)	End time (min)
1		DAD1C,Sig=210,4 Ref=360,100	15.490	959.976	50.535	53.590	50.85			14.889	15.963
2		DAD1C,Sig=210,4 Ref=360,100	16.349	939.665	49.465	51.794	49.15			15.963	16.905

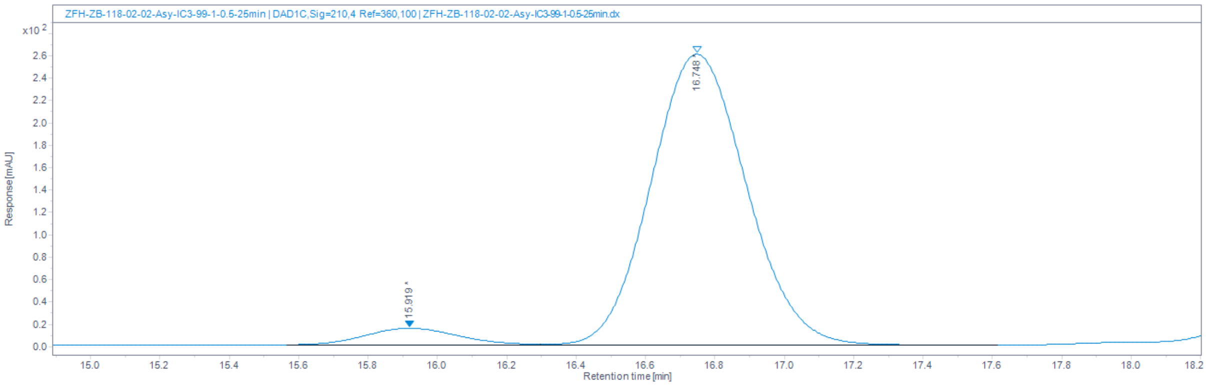

#	Name	Signal description	RT (min)	Area (mAU·s)	Area%	Height (mAU)	Height%	Amount	Concentration	Start time (min)	End time (min)
1		DAD1C,Sig=210,4 Ref=360,100	15.919	274.839	5.237	14.815	5.39			15.566	16.295
2		DAD1C,Sig=210,4 Ref=360,100	16.748	4973.109	94.763	259.876	94.61			16.295	17.616

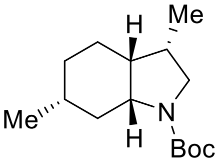

***tert*-butyl (3*S*,3a*R*,6*R*,7a*R*)-3,6-dimethyloctahydro-1*H*-indole-1-carboxylate** (**2d**). The title compound was synthesized according to **GPC**. The product was isolated by column chromatography using a gradient of pentene:ethyl acetate (50:1) and obtained as a colorless oil (23 mg, 0.091 mmol, 91%, 87:13 dr, 95:5 er). The major diastereomer could be separated by flash chromatography. Data for the major diastereomer: ^{1}H NMR (400 MHz, Chloroform-*d*) δ 3.86 – 3.43 (m, 2H), 3.07 – 2.75 (m, 1H), 2.33 – 1.91 (m, 3H), 1.62 – 1.41 (m, 13H), 1.16 – 1.00 (m, 4H), 0.98 – 0.85 (m, 4H). ^{13}C NMR (101 MHz, $CDCl_3$) δ 154.9, 154.7, 78.8, 78.6, 57.4, 52.9, 52.6, 44.0, 43.3, 39.2, 38.5, 37.3, 36.2, 35.9, 35.2, 30.8, 28.6, 27.9, 27.7, 22.0, 21.5, 21.3, 16.7, 16.5. $[\alpha]_D^{20}$: –42.6 (*c* = 0.5, CH_2Cl_2). HRMS (ESI) Calcd for $C_{15}H_{27}NNaO_2$ $[M+Na]^+$: 276.1934, found: 276.1933. Chiral GC-FID (50_5_5_100_0.2_140_5_200, *β*-Dex-120): t_1 = 200.76 min (minor enantiomer), t_2 = 203.09 min (major enantiomer).

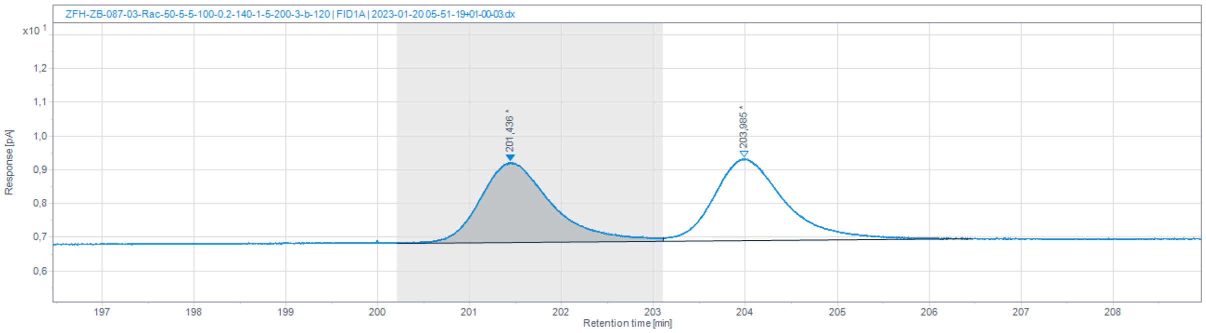

#	Name	Signal description	RT (min)	Area (pA·s)	Area%	Height (pA)	Height%	Amount	Concentration	Start time (min)	End time (min)
1		FID1A	201,436	126,390	49,517	2,359	49,40			200,208	203,109
2		FID1A	203,985	128,857	50,483	2,416	50,60			203,109	206,464

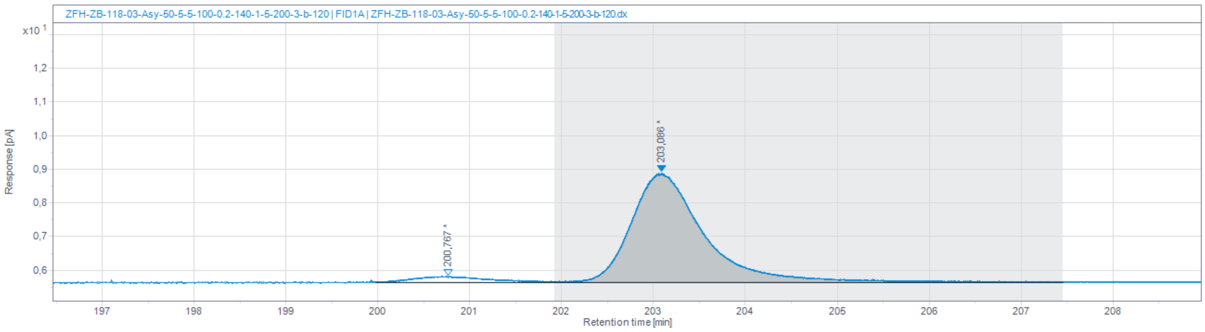

#	Name	Signal description	RT (min)	Area (pA·s)	Area%	Height (pA)	Height%	Amount	Concentration	Start time (min)	End time (min)
1		FID1A	200,767	9,007	4,860	0,164	4,82			199,979	201,928
2		FID1A	203,086	176,323	95,140	3,230	95,18			201,928	207,462

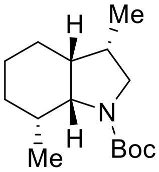

***tert*-butyl (3*S*,3a*R*,7*R*,7a*R*)-3,7-dimethyloctahydro-1*H*-indole-1-carboxylate** (**2e**). The title compound was synthesized according to **GPC**. The product was isolated by column chromatography using a gradient of pentene:ethyl acetate (50:1) and obtained as a colorless oil (24mg, 0.095 mmol, 95%, 83:17 dr, 91:9 er). The major diastereomer could be separated by flash chromatography. Data for the major diastereomer: ^{1}H NMR (400 MHz, Chloroform-*d*) δ 3.93 (t, *J* = 6.6 Hz, 1H), 3.72 (dd, *J* = 10.8, 7.2 Hz, 1H), 2.85 (t, *J* = 10.9 Hz, 1H), 2.22 – 2.04 (m, 3H), 1.69 – 1.61 (m, 1H), 1.48 – 1.17 (m, 14H), 0.96 – 0.85 (m, 6H). ^{13}C NMR (101 MHz, $CDCl_3$) δ 156.2, 78.9, 60.7, 53.3, 40.3, 35.5, 31.9, 28.5, 26.3, 19.3, 17.5, 12.0. HRMS (ESI) Calcd for $C_{15}H_{27}NNaO_2$ $[M+Na]^+$: 276.1934, found: 276.1933. The e.r. was determined through the corresponding benzoyl-protected product. $[\alpha]_D^{20}$: –73.2 (*c* = 1.0, CH_2Cl_2). HPLC on Chiralpak IA-3 column; hexane:isopropanol = 90:10; flow rate = 0.5 mL/min; UV detection at 254 nm; t_R = 14.21 min (minor), t_R = 17.69 min (major).

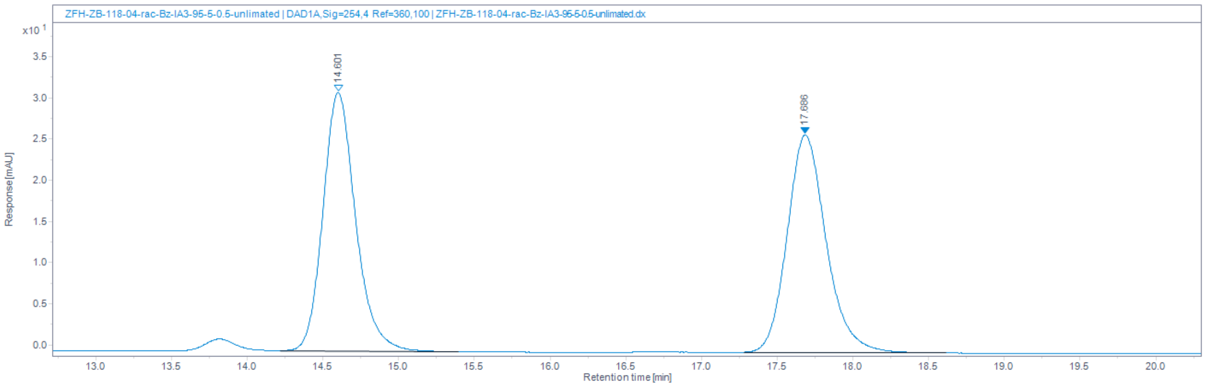

#	Name	Signal description	RT (min)	Area (mAU·s)	Area%	Height (mAU)	Height%	Amount	Concentration	Start time (min)	End time (min)
1		DAD1A,Sig=254,4 Ref=360,100	14.601	471.474	49.909	31.376	54.31			14.217	15.397
2		DAD1A,Sig=254,4 Ref=360,100	17.686	473.203	50.091	26.391	45.69			17.280	18.617

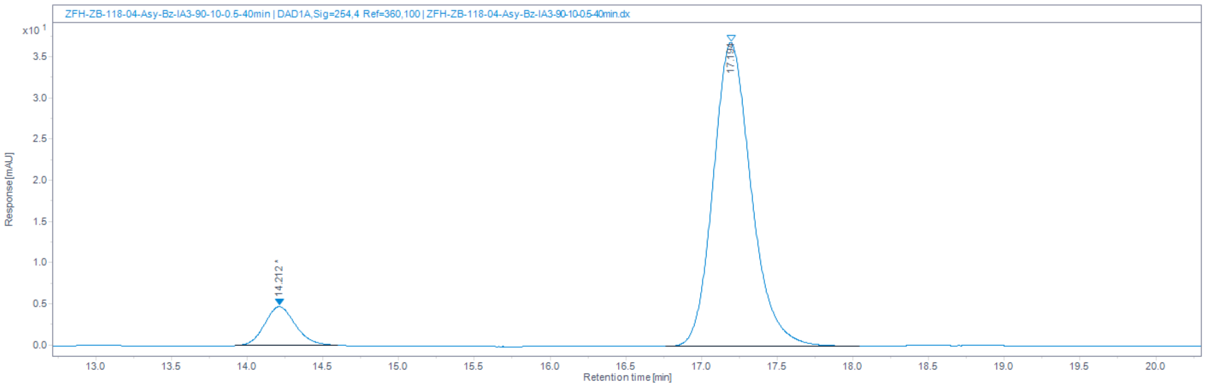

#	Name	Signal description	RT (min)	Area (mAU·s)	Area%	Height (mAU)	Height%	Amount	Concentration	Start time (min)	End time (min)
1		DAD1A,Sig=254,4 Ref=360,100	14.212	66.468	9.336	4.680	11.26			13.921	14.594
2		DAD1A,Sig=254,4 Ref=360,100	17.194	645.517	90.664	36.888	88.74			16.763	18.043

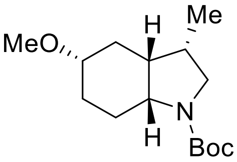

***tert*-butyl (3*S*,3a*R*,5*S*,7a*R*)-5-methoxy-3-methyloctahydro-1*H*-indole-1-carboxylate** (**2f**). The title compound was synthesized according to **GPC**. The product was isolated by column chromatography using a gradient of pentene:ethyl acetate (30:1) and obtained as a colorless oil (23 mg, 0.086 mmol, 86%, 83:17 dr, 95:5 er). The major diastereomer could be separated by flash chromatography. Data for the major diastereomer: ^{1}H NMR (400 MHz, Chloroform-*d*) δ 3.58 (d, *J* = 40.3 Hz, 2H), 3.36 (s, 3H), 3.18 – 2.95 (m, 2H), 2.74 (d, *J* = 99.6 Hz, 1H), 2.27 – 2.12 (m, 1H), 2.12 – 1.98 (m, 1H), 1.89 – 1.72 (m, 2H), 1.44 (s, 10H), 1.22 – 1.10 (m, 1H), 0.92 (d, *J* = 6.8 Hz, 4H). ^{13}C NMR (101 MHz, $CDCl_3$) δ 155.3, 78.7, 61.9, 57.5, 55.6, 52.4, 41.8, 34.6, 28.5, 28.2, 25.9, 24.7, 11.9. $[\alpha]_D^{20}$: –61.4 (*c* = 0.9, CH_2Cl_2). HRMS (ESI) Calcd for $C_{15}H_{27}NNaO_3$ $[M+Na]^+$: 292.1883, found: 292.1881. Chiral GC-FID (50_5_5_100_0.2_140_5_200, Hydrodex *β*-6TBDM): t_1 = 223.19 min (major enantiomer), t_2 = 224.39 min (minor enantiomer).

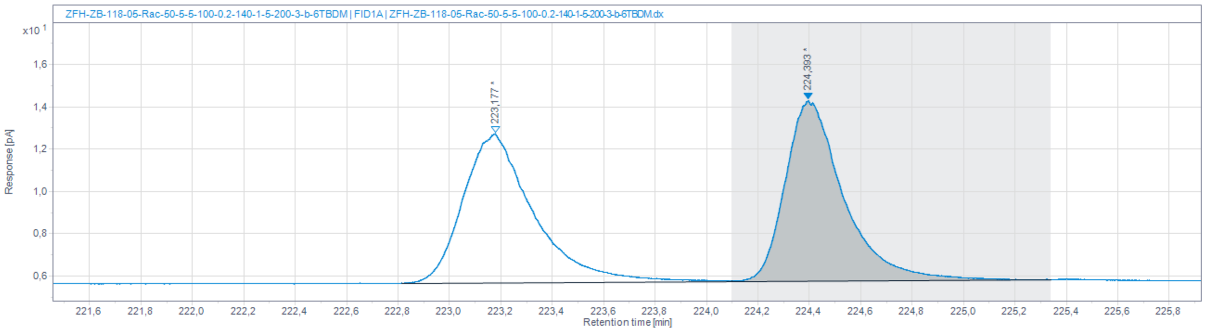

#	Name	Signal description	RT (min)	Area (pA·s)	Area%	Height (pA)	Height%	Amount	Concentration	Start time (min)	End time (min)
1		FID1A	223,177	133,668	49,985	7,031	45,26			222,815	224,099
2		FID1A	224,393	133,749	50,015	8,503	54,74			224,099	225,342

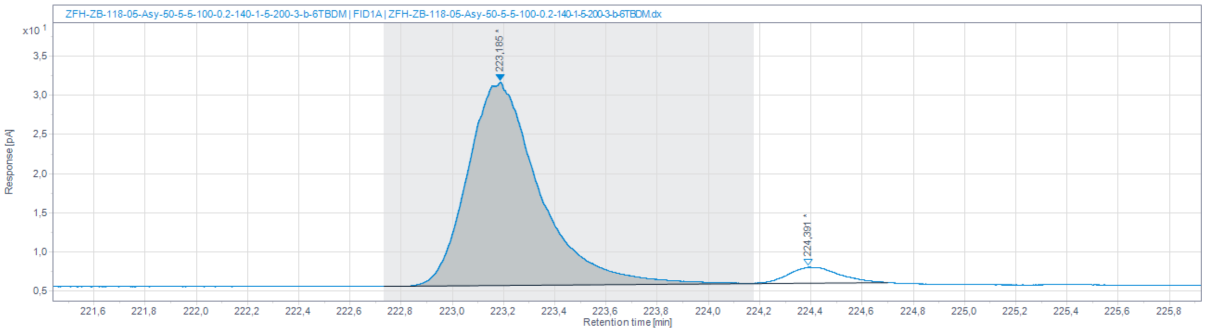

#	Name	Signal description	RT (min)	Area (pA·s)	Area%	Height (pA)	Height%	Amount	Concentration	Start time (min)	End time (min)
1		FID1A	223,185	509,206	94,732	26,057	92,71			222,735	224,177
2		FID1A	224,391	28,318	5,268	2,050	7,29			224,177	224,701

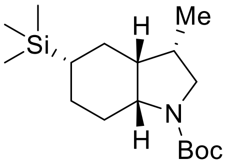

***tert*-butyl (3*S*,3a*R*,5*S*,7a*R*)-3-methyl-5-(trimethylsilyl)octahydro-1*H*-indole-1-carboxylate** (**2g**). The title compound was synthesized according to **GPC**. The product was isolated by column chromatography using a gradient of pentene:ethyl acetate (50:1) and obtained as a colorless oil (27 mg, 0.086 mmol, 86%, 90:10 dr, 91.5:8.5 er). The major diastereomer could be separated by flash chromatography. Data for the major diastereomer: ^{1}H NMR (400 MHz, Chloroform-*d*) δ 3.71 (s, 1H), 3.63 – 3.47 (m, 1H), 2.98 (t, J = 11.1 Hz, 1H), 2.68 (s, 1H), 2.20 – 2.04 (m, 1H), 1.93 – 1.79 (m, 1H), 1.45 (s, 13H), 1.05 – 0.83 (m, 5H), 0.55 – 0.32 (m, 1H), -0.06 (s, 9H). ^{13}C NMR (101 MHz, $CDCl_3$) δ 155.9, 78.9, 58.4, 52.7, 42.8, 35.2, 28.6, 23.4, 22.7, 21.3, 11.9, -3.6. $[\alpha]_D^{20}$: –35.5 (c = 1.0, CH_2Cl_2). HRMS (ESI) Calcd for $C_{17}H_{33}NNaO_2Si$ $[M+Na]^+$: 334.2173, found: 334.2174. Chiral GC-FID (50_5_5_100_0.2_200, Hydrodex β-6TBDM): t_1 = 231.96 min (major enantiomer), t_2 = 237.19 min (minor enantiomer).

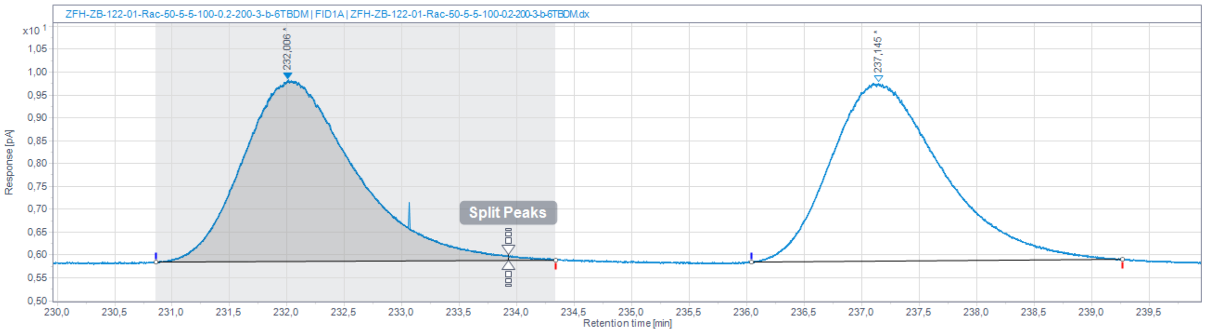

#	Name	Signal description	RT (min)	Area (pA·s)	Area%	Height (pA)	Height%	Amount	Concentration	Start time (min)	End time (min)
1		FID1A	232,006	268,814	50,539	3,960	50,50			230,859	234,339
2		FID1A	237,145	263,080	49,461	3,881	49,50			236,040	239,268

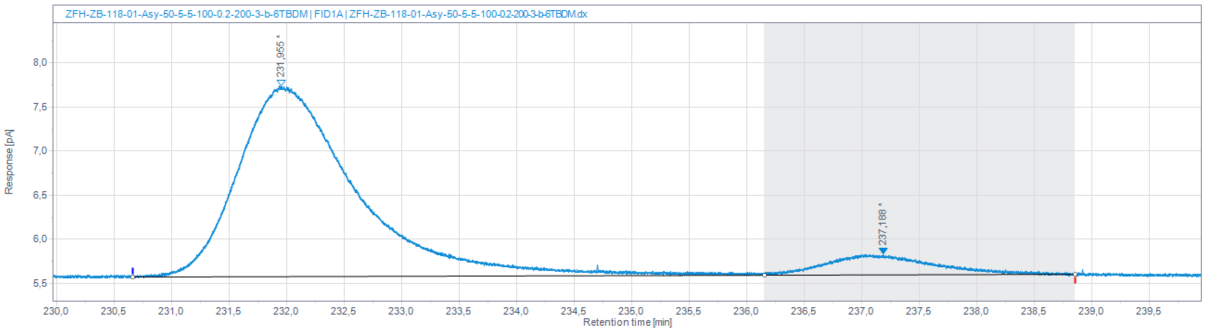

#	Name	Signal description	RT (min)	Area (pA·s)	Area%	Height (pA)	Height%	Amount	Concentration	Start time (min)	End time (min)
1		FID1A	231,955	153,141	91,571	2,142	90,72			230,664	236,158
2		FID1A	237,188	14,096	8,429	0,219	9,28			236,158	238,858

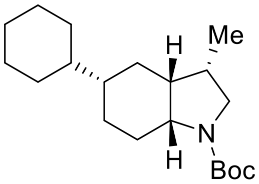

***tert*-butyl (3*S*,3a*R*,5*S*,7a*R*)-5-cyclohexyl-3-methyloctahydro-1*H*-indole-1-carboxylate** (**2h**). The title compound was synthesized according to **GPC**. The product was isolated by column chromatography using a gradient of pentene:ethyl acetate (50:1) and obtained as a colorless oil (30 mg, 0.093 mmol, 93%, 78:22 dr, 95:5 er). The major diastereomer could be separated by flash chromatography. Data for the major diastereomer: ^{1}H NMR (400 MHz, Chloroform-*d*) δ 3.65 (s, 1H), 3.60 – 3.47 (m, 1H), 2.97 (t, *J* = 11.0 Hz, 1H), 2.68 (s, 1H), 2.19 – 2.06 (m, 1H), 1.99 – 1.80 (m, 1H), 1.74 – 1.62 (m, 5H), 1.45 (s, 12H), 1.21 – 0.88 (m, 11H), 0.77 (q, *J* = 12.3 Hz, 1H). ^{13}C NMR (101 MHz, $CDCl_3$) δ 155.8, 78.8, 58.4, 52.7, 43.4, 41.6, 34.9, 30.3, 30.0, 28.6, 26.8, 26.79, 26.77, 25.9, 23.9, 12.0. $[\alpha]_D^{20}$: –39.9 (*c* = 1.0, CH_2Cl_2). HRMS (ESI) Calcd for $C_{20}H_{35}NNaO_2$ $[M+Na]^+$: 344.2560, found: 344.2561. HPLC on Chiralpak IA-3 column; hexane:isopropanol = 99:1; flow rate = 0.5 mL/min; UV detection at 210 nm; t_R = 8.36 min (major), t_R = 8.69 min (minor).

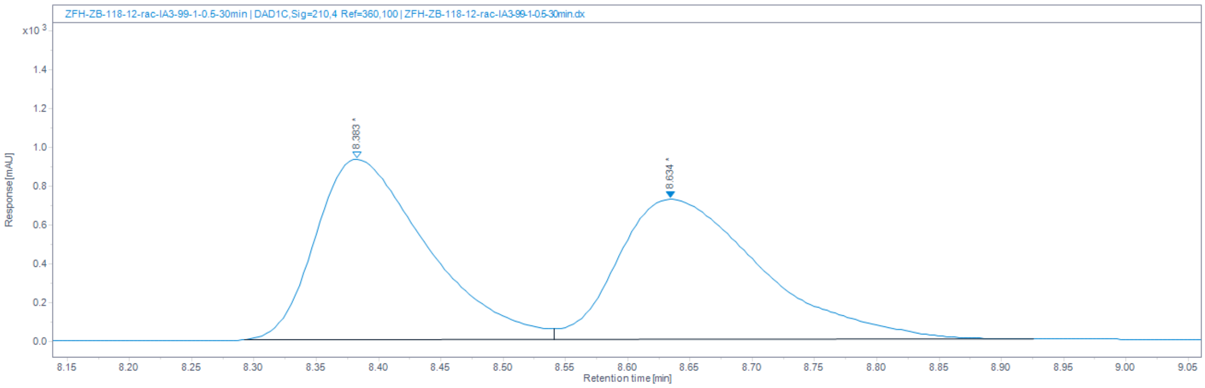

#	Name	Signal description	RT (min)	Area (mAU·s)	Area%	Height (mAU)	Height%	Amount	Concentration	Start time (min)	End time (min)
1		DAD1C,Sig=210,4 Ref=360,100	8.383	5799.901	50.090	931.007	56.35			8.292	8.541
2		DAD1C,Sig=210,4 Ref=360,100	8.634	5779.133	49.910	721.301	43.65			8.541	8.926

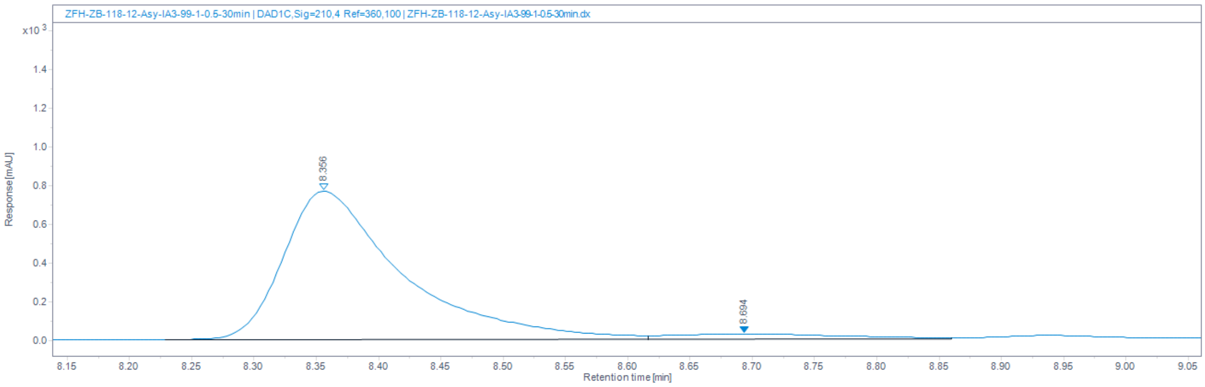

#	Name	Signal description	RT (min)	Area (mAU·s)	Area%	Height (mAU)	Height%	Amount	Concentration	Start time (min)	End time (min)
1		DAD1C,Sig=210,4 Ref=360,100	8.356	5088.835	94.845	765.732	96.46			8.228	8.617
2		DAD1C,Sig=210,4 Ref=360,100	8.694	276.582	5.155	28.135	3.54			8.617	8.860

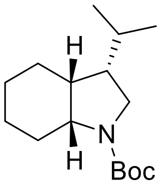

***tert*-butyl (3*S*,3a*R*,7a*R*)-3-isopropyloctahydro-1*H*-indole-1-carboxylate** (**2i**). The title compound was synthesized according to **GPC**. The product was isolated by column chromatography using a gradient of pentene:ethyl acetate (50:1) and obtained as a colorless oil (26.5mg, 0.099 mmol, 99%, 71:29 dr, 57:43 er). The major diastereomer could be separated by flash chromatography. Data for the major diastereomer: ^{1}H NMR (400 MHz, Chloroform-*d*) δ 3.64 – 3.47 (d, *J* = 68.4 Hz, 2H), 3.05 (t, *J* = 10.3 Hz, 1H), 2.87 – 2.45 (m, 1H), 2.04 – 1.92 (m, 1H), 1.72 – 1.64 (m, 1H), 1.61 – 1.47 (m, 3H), 1.44 (s, 9H), 1.40 – 1.27 (m, 2H), 1.23 – 0.98 (m, 3H), 0.88 (d, *J* = 6.0 Hz, 3H), 0.83 (d, *J* = 6.1 Hz, 3H). ^{13}C NMR (101 MHz, $CDCl_3$) δ 156.2, 78.9, 58.6, 50.7, 48.6, 40.3, 28.5, 26.4, 24.7, 21.8, 21.6, 21.1, 20.5. $[\alpha]_D^{20}$: –2.0 (*c* = 1.0, CH_2Cl_2). HRMS (ESI) Calcd for $C_{16}H_{29}NNaO_2$ $[M+Na]^+$: 290.2091, found: 290.2091. Chiral GC-FID (50_5_5_100_0.2_140_5_200, *β*-Dex-120): t_1 = 219.70 min (minor enantiomer), t_2 = 220.72 min (major enantiomer).

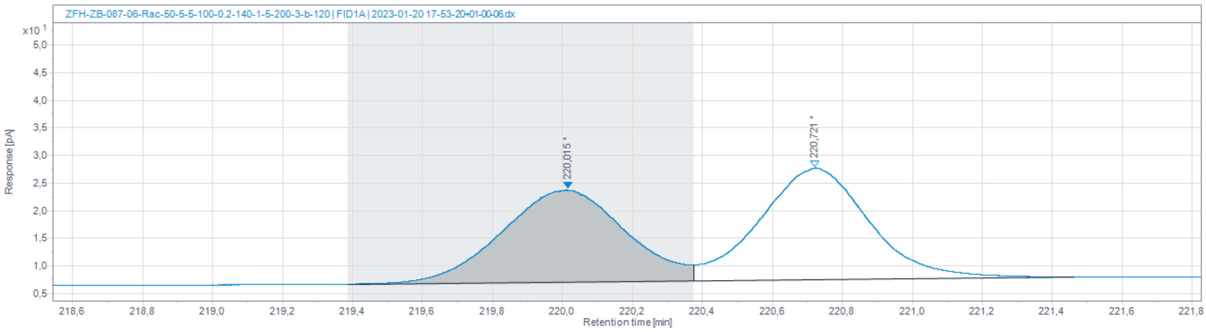

#	Name	Signal description	RT (min)	Area (pA·s)	Area%	Height (pA)	Height%	Amount	Concentration	Start time (min)	End time (min)
1		FID1A	220,015	407,847	48,169	16,622	45,15			219,387	220,375
2		FID1A	220,721	438,859	51,831	20,190	54,85			220,375	221,463

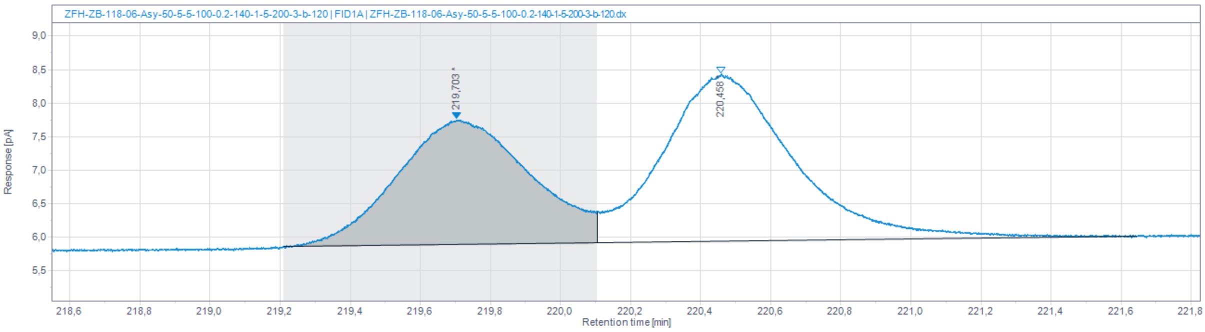

#	Name	Signal description	RT (min)	Area (pA·s)	Area%	Height (pA)	Height%	Amount	Concentration	Start time (min)	End time (min)
1		FID1A	219,703	49,015	43,073	1,855	42,76			219,211	220,105
2		FID1A	220,458	64,779	56,927	2,483	57,24			220,105	221,643

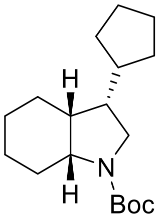

***tert*-butyl (3*S*,3a*R*,7a*R*)-3-cyclopentyloctahydro-1*H*-indole-1-carboxylate** (**2j**). The title compound was synthesized according to **GPC**. The product was isolated by column chromatography using a gradient of pentene:ethyl acetate (50:1) and obtained as a colorless oil (29 mg, 0.099 mmol, 99%, 71:29 dr, 57:43 er). The major diastereomer could be separated by flash chromatography. Data for the major diastereomer: ^{1}H NMR (400 MHz, Chloroform-*d*) δ 3.65 – 3.55 (m, 2H), 3.08 (t, *J* = 10.1 Hz, 1H), 2.77 – 2.54 (m, 1H), 2.04 – 1.91 (m, 1H), 1.79 – 1.37 (m, 21H), 1.27 – 1.02 (m, 5H). ^{13}C NMR (101 MHz, $CDCl_3$) δ 155.9, 78.6, 58.4, 51.2, 47.3, 38.5, 31.9, 30.9, 28.6, 25.3, 24.9, 24.7, 22.4, 20.5. $[\alpha]_D^{20}$: –7.3 (*c* = 1.0, CH_2Cl_2). HRMS (ESI) Calcd for $C_{18}H_{31}NNaO_2$ $[M+Na]^+$: 316.2247, found: 316.2245. HPLC on Chiralpak IC-3 column; hexane:isopropanol = 99:1; flow rate = 0.5 mL/min; UV detection at 210 nm; t_R = 17.35 min (minor), t_R = 18.44 min (major).

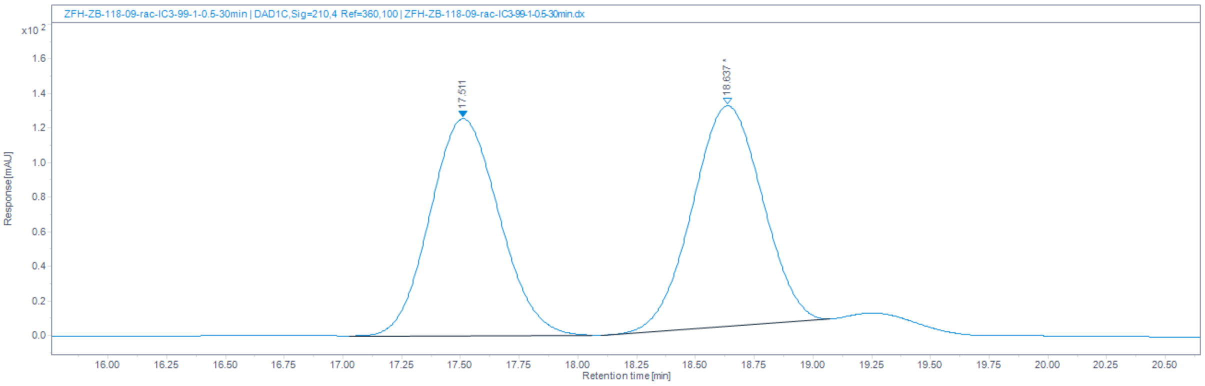

#	Name	Signal description	RT (min)	Area (mAU·s)	Area%	Height (mAU)	Height%	Amount	Concentration	Start time (min)	End time (min)
1		DAD1C,Sig=210,4 Ref=360,100	17.511	2566.539	49.299	125.454	49.60			17.028	18.059
2		DAD1C,Sig=210,4 Ref=360,100	18.637	2639.537	50.701	127.467	50.40			18.100	19.070

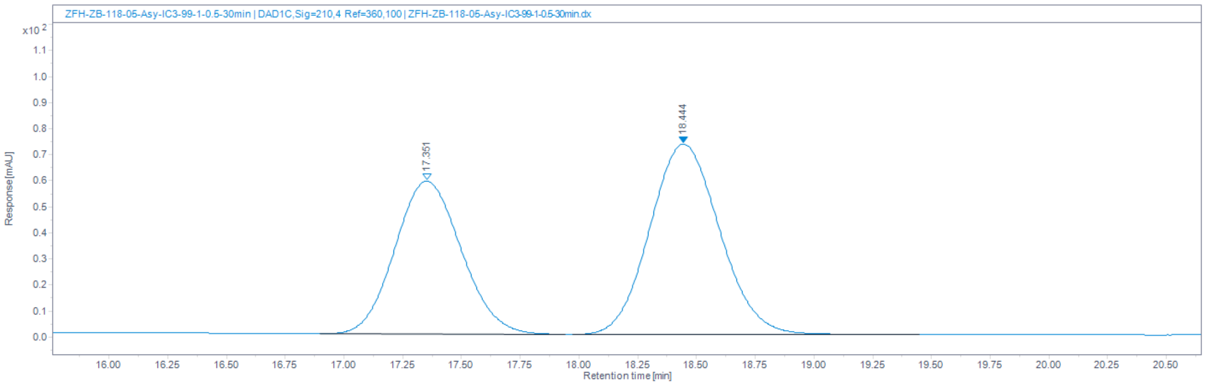

#	Name	Signal description	RT (min)	Area (mAU·s)	Area%	Height (mAU)	Height%	Amount	Concentration	Start time (min)	End time (min)
1		DAD1C,Sig=210,4 Ref=360,100	17.351	1157.378	43.291	58.718	44.55			16.896	17.940
2		DAD1C,Sig=210,4 Ref=360,100	18.444	1516.106	56.709	73.098	55.45			17.973	19.446

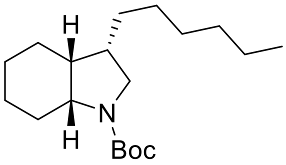

***tert*-butyl (3*S*,3a*R*,7a*R*)-3-hexyloctahydro-1*H*-indole-1-carboxylate** (**2k**). The title compound was synthesized according to **GPC**. The product was isolated by column chromatography using a gradient of pentene:ethyl acetate (50:1) and obtained as a colorless oil (30 mg, 0.098 mmol, 98%, 81:19 dr, 81:19 er). The major diastereomer could be separated by flash chromatography. Data for the major diastereomer: ^{1}H NMR (400 MHz, Chloroform-*d*) δ 3.66 – 3.51 (d, *J* = 60.4 Hz, 2H), 3.01 (t, *J* = 9.7 Hz, 1H), 2.80 – 2.42 (m, 1H), 2.04 – 1.88 (m, 2H), 1.69 – 1.66 (m, 1H), 1.45 (m, 12H), 1.32 – 1.07 (m, 13H), 0.87 (t, *J* = 6.7 Hz, 3H). ^{13}C NMR (101 MHz, $CDCl_3$) δ 155.9, 78.8, 58.3, 51.6, 40.8, 31.8, 29.5, 28.6, 28.1, 27.6, 26.9, 24.6, 22.6, 22.1, 20.6, 14.1. $[\alpha]_D^{20}$: –30.3 (*c* = 1.0, CH_2Cl_2). HRMS (ESI) Calcd for $C_{19}H_{35}NNaO_2$ $[M+Na]^+$: 332.2559, found: 332.2560. HPLC on Chiralpak IA-3 column; hexane:isopropanol = 99:1; flow rate = 0.5 mL/min; UV detection at 210 nm; t_R = 8.22 min (major), t_R = 8.68 min (minor).

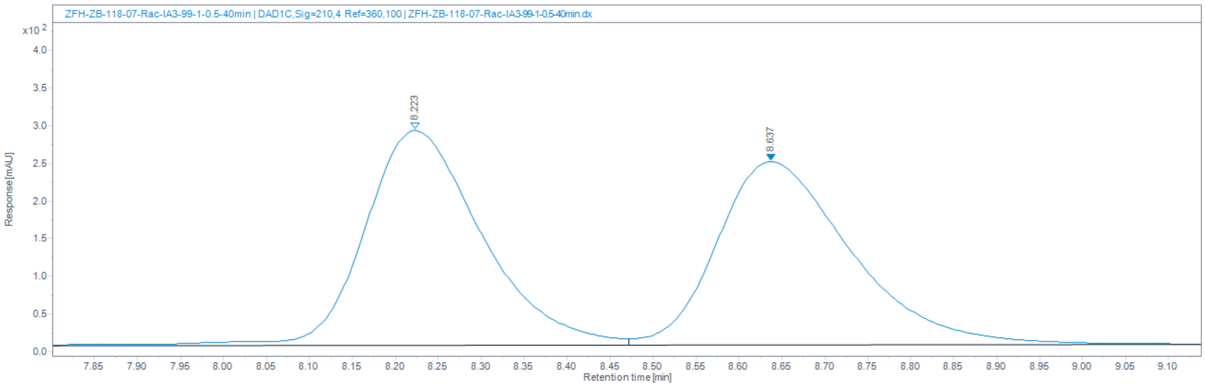

#	Name	Signal description	RT (min)	Area (mAU·s)	Area%	Height (mAU)	Height%	Amount	Concentration	Start time (min)	End time (min)
1		DAD1C,Sig=210,4 Ref=360,100	8.223	2719.395	50.701	284.828	53.97			7.337	8.472
2		DAD1C,Sig=210,4 Ref=360,100	8.637	2644.200	49.299	242.935	46.03			8.472	9.224

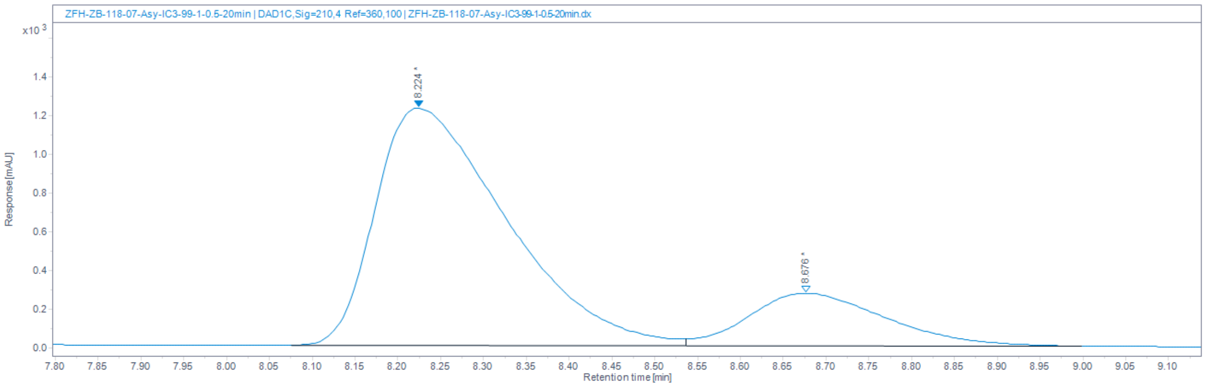

#	Name	Signal description	RT (min)	Area (mAU·s)	Area%	Height (mAU)	Height%	Amount	Concentration	Start time (min)	End time (min)
1		DAD1C,Sig=210,4 Ref=360,100	8.224	13056.390	81.221	1225.375	81.87			8.076	8.536
2		DAD1C,Sig=210,4 Ref=360,100	8.676	3018.666	18.779	271.330	18.13			8.536	8.999

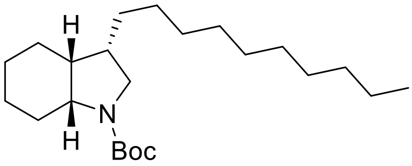

***tert*-butyl (3*S*,3a*R*,7a*R*)-3-decyloctahydro-1*H*-indole-1-carboxylate** (**2l**). The title compound was synthesized according to **GPC**. The product was isolated by column chromatography using a gradient of pentene:ethyl acetate (50:1) and obtained as a colorless oil (35.5mg, 0.097 mmol, 97%, 81:19 dr, 80:20 er). The major diastereomer could be separated by flash chromatography. Data for the major diastereomer: ^{1}H NMR (400 MHz, Chloroform-*d*) δ 3.66 – 3.51 (m, 2H), 3.01 (t, *J* = 9.7 Hz, 1H), 2.72 – 2.46 (m, 1H), 2.03 – 1.90 (m, 2H), 1.70 – 1.64 (m, 1H), 1.45 (m, 12H), 1.25 (m, 21H), 0.87 (t, *J* = 6.8 Hz, 3H). ^{13}C NMR (101 MHz, $CDCl_3$) δ 155.9, 78.8, 58.3, 51.6, 40.8, 31.9, 29.9, 29.65, 29.63, 29.6, 29.4, 28.6, 28.2, 27.6, 24.6, 22.7, 22.2, 20.7, 14.1. $[\alpha]_D^{20}$: –22.5 (*c* = 1.0, CH_2Cl_2). HRMS (ESI) Calcd for $C_{23}H_{43}NNaO_2$ $[M+Na]^+$: 388.3186, found: 388.3186. HPLC on Chiralpak IA-3 column; hexane:isopropanol = 99:1; flow rate = 0.5 mL/min; UV detection at 210 nm; t_R = 9.73 min (major), t_R = 10.31 min (minor).

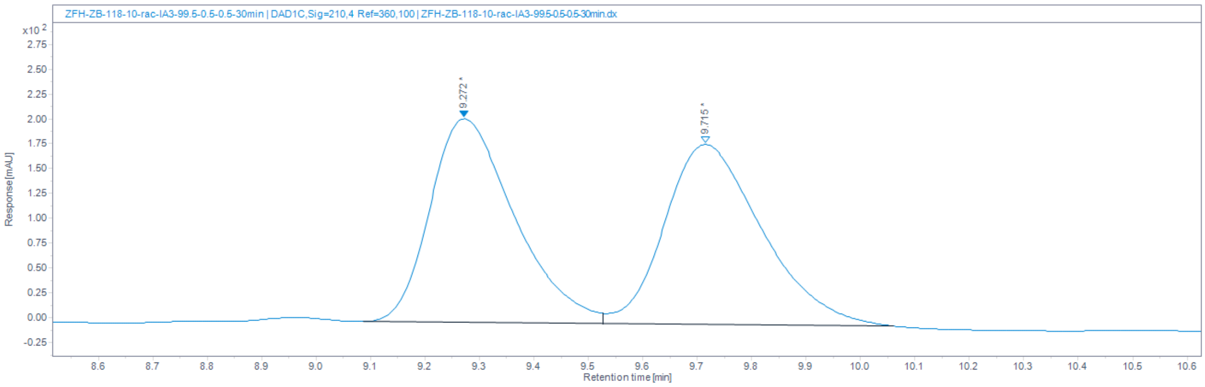

#	Name	Signal description	RT (min)	Area (mAU·s)	Area%	Height (mAU)	Height%	Amount	Concentration	Start time (min)	End time (min)
1		DAD1C,Sig=210,4 Ref=360,100	9.272	2212.809	49.472	204.926	53.08			9.086	9.526
2		DAD1C,Sig=210,4 Ref=360,100	9.715	2260.070	50.528	181.160	46.92			9.526	10.061

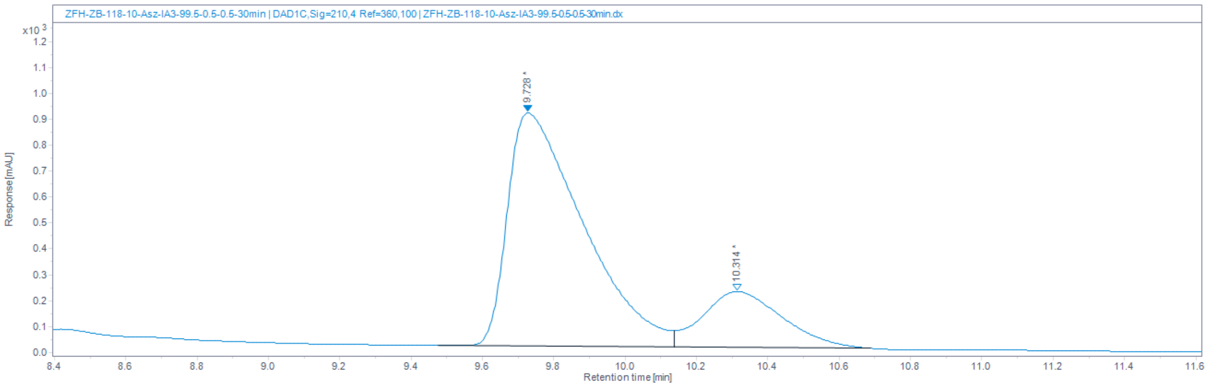

#	Name	Signal description	RT (min)	Area (mAU·s)	Area%	Height (mAU)	Height%	Amount	Concentration	Start time (min)	End time (min)
1		DAD1C,Sig=210,4 Ref=360,100	9.728	13214.368	79.791	897.892	80.66			9.475	10.138
2		DAD1C,Sig=210,4 Ref=360,100	10.314	3346.934	20.209	215.226	19.34			10.138	10.690

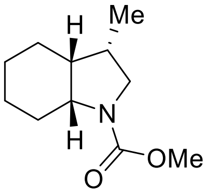

methyl (3*S*,3a*R*,7a*R*)-3-methyloctahydro-1*H*-indole-1-carboxylate (**2m**). The title compound was synthesized according to **GPC**. The product was isolated by column chromatography using a gradient of pentene:ethyl acetate (30:1) and obtained as a colorless oil (17 mg, 0.085 mmol, 85%, 78:22 dr, 80:20 er). The major diastereomer could be separated by flash chromatography. Data for the major diastereomer: ^{1}H NMR (400 MHz, Chloroform-*d*) δ 3.72 – 3.54 (m, 5H), 3.17 – 2.95 (m, 1H), 2.67 – 2.32 (m, 1H), 2.20 – 2.04 (m, 1H), 1.99 – 1.83 (m, 1H), 1.73 – 1.61 (m, 1H), 1.58 – 1.31 (m, 3H), 1.24 – 1.01 (m, 3H), 0.92 (d, J = 6.9 Hz, 3H). ^{13}C NMR (101 MHz, $CDCl_3$) δ 156.3, 58.7, 52.3, 51.8, 41.4, 35.2, 26.7, 24.3, 22.3, 20.7, 12.3. $[\alpha]_D^{20}$: –29.6 (c = 0.5, CH_2Cl_2). HRMS (ESI) Calcd for $C_{11}H_{19}NNaO_2$ $[M+Na]^+$: 220.1308, found: 220.1307. HPLC on Chiralpak IA-3 column; hexane:isopropanol = 99:1; flow rate = 0.5 mL/min; UV detection at 210 nm; t_R = 16.47 min (major), t_R = 17.63 min (minor).

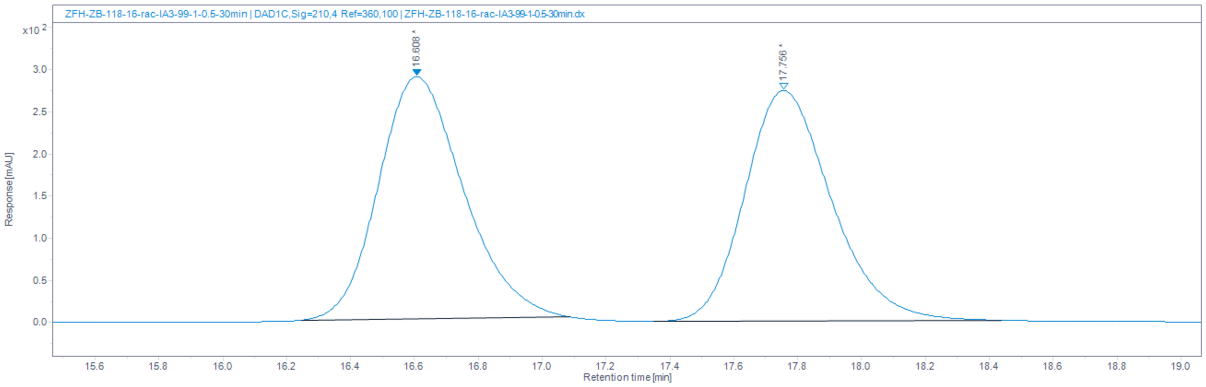

#	Name	Signal description	RT (min)	Area (mAU·s)	Area%	Height (mAU)	Height%	Amount	Concentration	Start time (min)	End time (min)
1		DAD1C,Sig=210,4 Ref=360,100	16.608	5319.859	50.721	287.310	51.24			16.246	17.088
2		DAD1C,Sig=210,4 Ref=360,100	17.756	5168.699	49.279	273.451	48.76			17.351	18.439

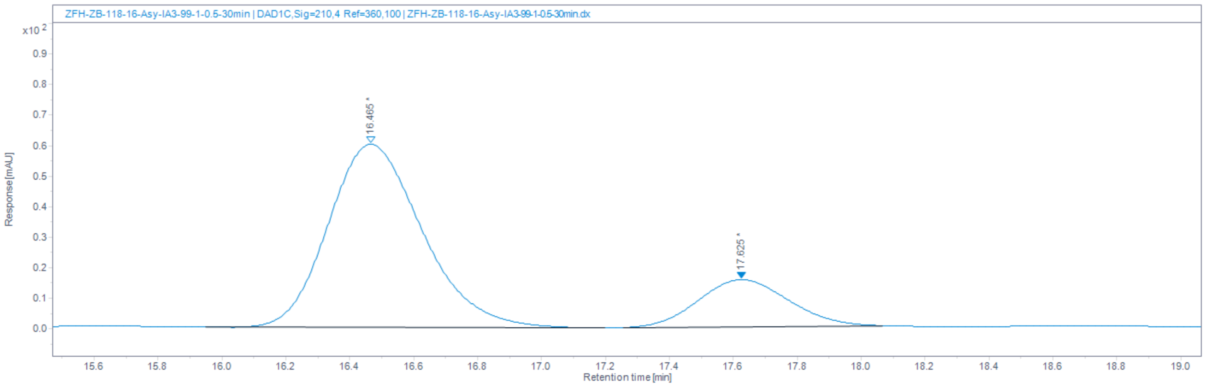

#	Name	Signal description	RT (min)	Area (mAU·s)	Area%	Height (mAU)	Height%	Amount	Concentration	Start time (min)	End time (min)
1		DAD1C,Sig=210,4 Ref=360,100	16.465	1231.747	80.218	60.074	79.59			15.949	17.198
2		DAD1C,Sig=210,4 Ref=360,100	17.625	303.744	19.782	15.405	20.41			17.254	18.066

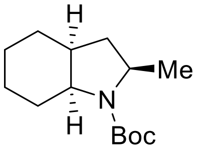

<u>***tert*-butyl (2*R*,3a*S*,7a*S*)-2-methyloctahydro-1*H*-indole-1-carboxylate**</u> (**5a**): The title compound was synthesized according to **GPD**. The product was isolated by column chromatography using a gradient of pentene:ethyl acetate (50:1) and obtained as a colorless oil (23.6 mg, 0.099 mmol, 99%, 76:24 dr, 92.5:7.5 er). The major diastereomer could not be separated by flash chromatography. Data for the mixture of all diastereomers: ^{1}H NMR (400 MHz, Chloroform-*d*) δ 3.98 – 3.56 (m, 2H), 2.28 – 2.08 (m, 1H), 2.08 – 1.84 (m, 2H), 1.76 – 1.53 (m, 4H), 1.45 (s, 9H), 1.38 – 1.22 (m, 4H), 1.22 – 1.02 (m, 3H). ^{13}C NMR (101 MHz, $CDCl_3$) δ 154.9, 78.5, 57.8, 53.2, 36.0, 34.6, 33.4, 29.6, 28.6, 26.1, 24.2, 20.6. HRMS (ESI) Calcd for $C_{14}H_{25}NNaO_2$ [M+Na]$^+$: 262.1778, found: 262.1776. Chiral GC-FID (50_5_5_200, *β*-Dex-325): t_1 = 34.84 min (minor enantiomer), t_2 = 34.98 min (major enantiomer).

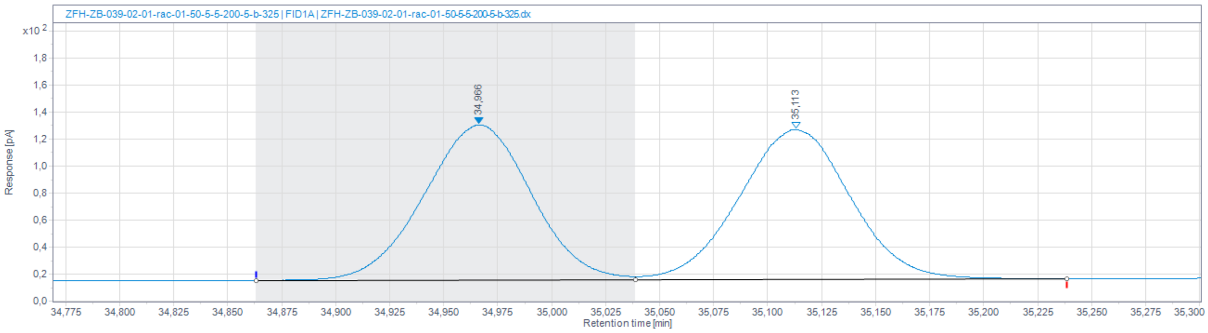

#	Name	Signal description	RT (min)	Area (pA·s)	Area%	Height (pA)	Height%	Amount	Concentration	Start time (min)	End time (min)
1		FID1A	34,966	396,514	49,912	114,438	50,82			34,863	35,039
2		FID1A	35,113	397,907	50,088	110,723	49,18			35,039	35,239

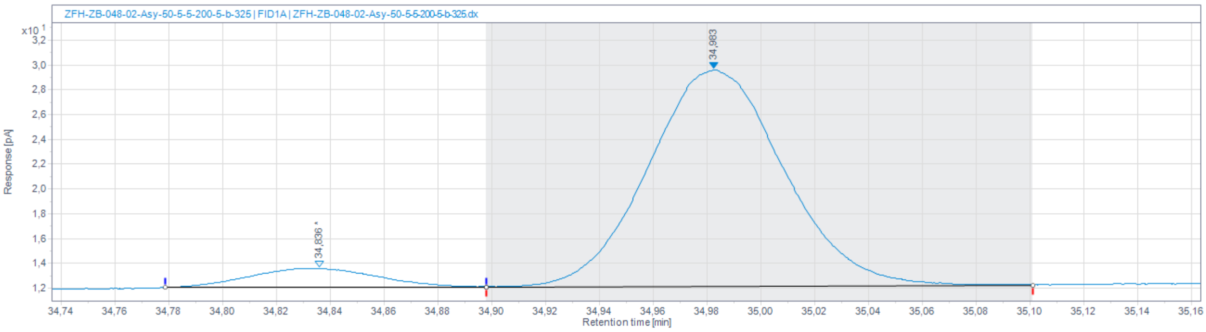

#	Name	Signal description	RT (min)	Area (pA·s)	Area%	Height (pA)	Height%	Amount	Concentration	Start time (min)	End time (min)
1		FID1A	34,836	4,953	7,510	1,517	8,02			34,779	34,898
2		FID1A	34,983	60,997	92,490	17,403	91,98			34,898	35,101

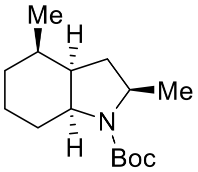

tert-butyl (2*R*,3a*R*,4*R*,7a*S*)-2,4-dimethyloctahydro-1*H*-indole-1-carboxylate (**5b**). The title compound was synthesized according to **GPD**. The product was isolated by column chromatography using a gradient of pentene:ethyl acetate (50:1) and obtained as a colorless oil (23.6 mg, 0.093 mmol, 93%, 71:29 dr, 93:7 er). The major diastereomer could not be separated by flash chromatography. Data for the mixture of all diastereomers: ^{1}H NMR (400 MHz, Chloroform-*d*) δ 4.01 – 3.57 (m, 2H), 2.39 – 2.03 (m, 1H), 2.02 – 1.83 (m, 2H), 1.81 – 1.59 (m, 2H), 1.51 – 1.35 (m, 11H), 1.34 – 1.09 (m, 4H), 1.09 – 0.95 (m, 2H), 0.92 – 0.81 (m, 3H). ^{13}C NMR (101 MHz, $CDCl_3$) δ 154.6, 154.4, 78.7, 78.6, 78.5, 78.4, 58.7, 58.3, 58.2, 57.9, 55.2, 53.04, 53.0, 52.9, 51.6, 42.9, 42.4, 42.1, 40.8, 39.8, 32.1, 31.75, 31.7, 31.6, 31.5, 29.9, 29.6, 29.3, 29.04, 29.0, 28.9, 28.8, 28.6, 27.4, 27.1, 26.5, 24.1, 24.0, 23.9, 23.6, 23.1, 22.2, 21.8, 20.9, 20.8, 20.03, 20.0, 18.5, 18.4. HRMS (ESI) Calcd for $C_{15}H_{27}NNaO_2$ $[M+Na]^+$: 276.1934, found: 276.1934. Chiral GC-FID (50_5_5_100_0.2_140_5_200, *β*-Dex-325): t_1 = 171.23 min (minor enantiomer), t_2 = 174.59 min (major enantiomer).

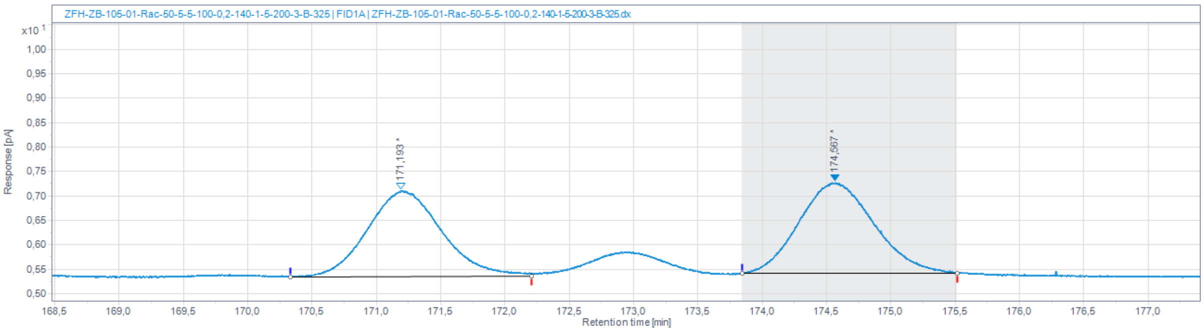

#	Name	Signal description	RT (min)	Area (pA·s)	Area%	Height (pA)	Height%	Amount	Concentration	Start time (min)	End time (min)
1		FID1A	171,193	70,186	48,410	1,755	48,67			170,332	172,210
2		FID1A	174,567	74,797	51,590	1,851	51,33			173,845	175,519

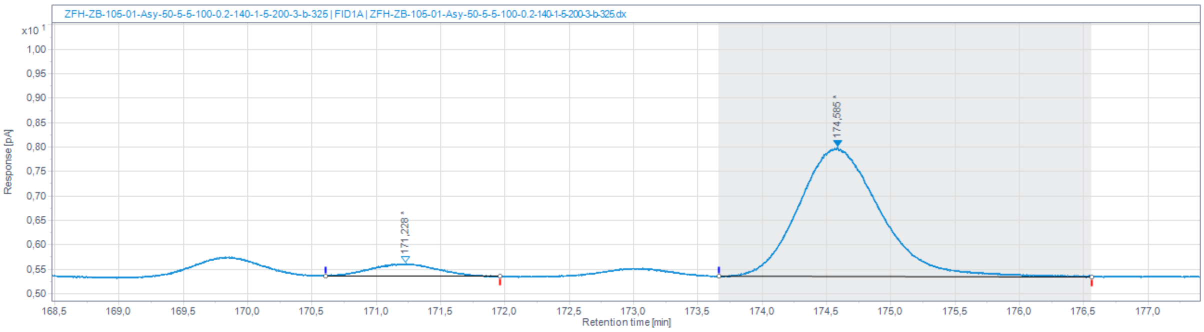

#	Name	Signal description	RT (min)	Area (pA·s)	Area%	Height (pA)	Height%	Amount	Concentration	Start time (min)	End time (min)
1		FID1A	171,228	8,943	7,213	0,248	8,62			170,603	171,965
2		FID1A	174,585	115,048	92,787	2,633	91,38			173,666	176,563

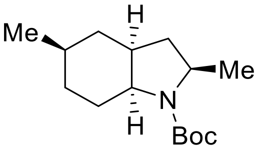

***tert*-butyl (2*R*,3a*S*,5*R*,7a*S*)-2,5-dimethyloctahydro-1*H*-indole-1-carboxylate** (**5c**). The title compound was synthesized according to **GPD**. The product was isolated by column chromatography using a gradient of pentene:ethyl acetate (50:1) and obtained as a colorless oil (21 mg, 0.083 mmol, 83%, 86:14 dr, 91:9 er). The major diastereomer could not be separated by flash chromatography. Data for the mixture of all diastereomers: ^{1}H NMR (400 MHz, Chloroform-*d*) δ 3.80 – 3.58 (m, 2H), 2.23 – 2.08 (m, 2H), 1.77 – 1.49 (m, 6H), 1.45 (s, 9H), 1.31 (d, *J* = 6.3 Hz, 3H), 1.23 – 1.13 (m, 3H), 0.93 (d, *J* = 6.6 Hz, 3H). ^{13}C NMR (101 MHz, $CDCl_3$) δ 155.6, 78.7, 58.2, 54.1, 39.5, 36.6, 35.5, 29.0, 28.5, 27.8, 25.3, 23.1, 22.3. HRMS (ESI) Calcd for $C_{15}H_{27}NNaO_2$ $[M+Na]^+$: 276.1934, found: 276.1934. Chiral GC-FID (50_5_5_100_0.2_140_5_200, *β*-Dex-325): t_1 = 160.62 min (minor enantiomer), t_2 = 162.87 min (major enantiomer).

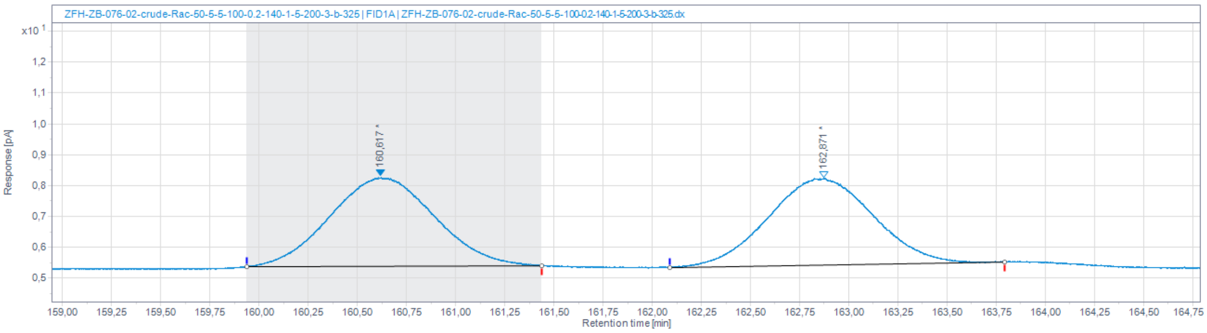

#	Name	Signal description	RT (min)	Area (pA·s)	Area%	Height (pA)	Height%	Amount	Concentration	Start time (min)	End time (min)
1		FID1A	160,617	105,629	50,493	2,862	50,65			159,938	161,440
2		FID1A	162,871	103,565	49,507	2,789	49,35			162,090	163,795

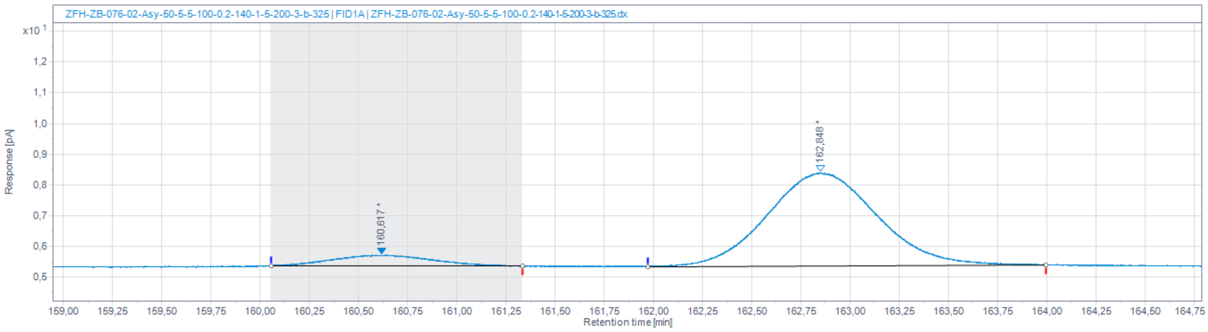

#	Name	Signal description	RT (min)	Area (pA·s)	Area%	Height (pA)	Height%	Amount	Concentration	Start time (min)	End time (min)
1		FID1A	160,617	11,837	9,208	0,340	10,14			160,056	161,335
2		FID1A	162,848	116,717	90,792	3,012	89,86			161,973	163,996

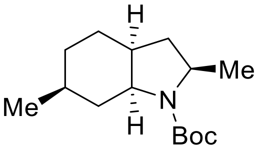

***tert*-butyl (2*R*,3a*S*,6*S*,7a*S*)-2,6-dimethyloctahydro-1*H*-indole-1-carboxylate** (**5d**). The title compound was synthesized according to **GPD**. The product was isolated by column chromatography using a gradient of pentene:ethyl acetate (50:1) and obtained as a colorless oil (22 mg, 0.087 mmol, 87%, 79:21 dr, 94:6 er). The major diastereomer could not be separated by flash chromatography. Data for the mixture of all diastereomers: ^{1}H NMR (400 MHz, Chloroform-*d*) δ 4.04 – 3.56 (m, 2H), 2.24 – 2.08 (m, 1H), 2.05 – 1.79 (m, 2H), 1.71 – 1.56 (m, 3H), 1.45 (s, 10H), 1.34 – 1.15 (m, 4H), 1.06 – 0.68 (m, 5H). ^{13}C NMR (101 MHz, $CDCl_3$) δ 154.6, 154.4, 78.7, 78.5, 58.2, 58.0, 57.6, 57.4, 53.3, 52.0, 38.5, 38.2, 36.5, 36.4, 35.7, 35.6, 35.3, 34.3, 34.0, 33.3, 33.1, 30.8, 29.3, 28.6, 25.8, 23.0, 22.5, 22.3, 22.1, 21.6, 20.7. HRMS (ESI) Calcd for $C_{15}H_{27}NNaO_2$ $[M+Na]^+$: 276.1934, found: 276.1933. Chiral GC-FID (50_5_5_100_0.2_140_5_200, *β*-Dex-325): t_1 = 156.79 min (minor enantiomer), t_2 = 158.74 min (major enantiomer).

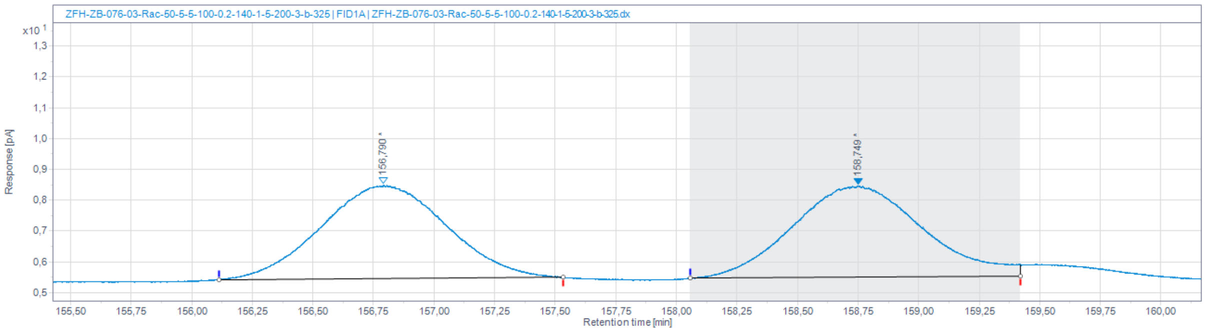

#	Name	Signal description	RT (min)	Area (pA·s)	Area%	Height (pA)	Height%	Amount	Concentration	Start time (min)	End time (min)
1		FID1A	156,790	108,340	49,706	3,023	50,62			156,111	157,532
2		FID1A	158,749	109,623	50,294	2,949	49,38			158,058	159,419

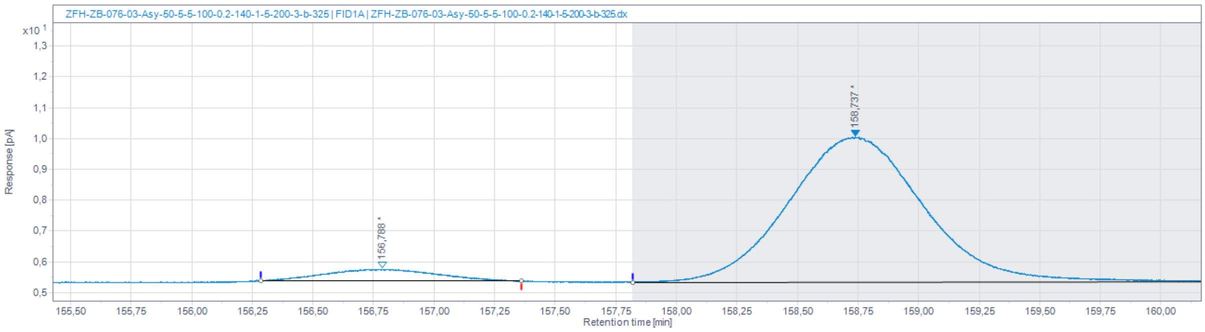

#	Name	Signal description	RT (min)	Area (pA·s)	Area%	Height (pA)	Height%	Amount	Concentration	Start time (min)	End time (min)
1		FID1A	156,788	11,937	6,114	0,372	7,33			156,285	157,362
2		FID1A	158,737	183,307	93,886	4,699	92,67			157,822	160,376

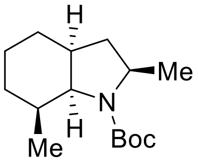

***tert*-butyl (2*R*,3a*S*,7*S*,7a*S*)-2,7-dimethyloctahydro-1*H*-indole-1-carboxylate** (**5e**). The title compound was synthesized according to **GPD**. The product was isolated by column chromatography using a gradient of pentene:ethyl acetate (50:1) and obtained as a colorless oil (24 mg, 0.095 mmol, 95%, 85:15 dr, 84:16 er). The major diastereomer could not be separated by flash chromatography. Data for the mixture of all diastereomers: ^{1}H NMR (400 MHz, Chloroform-*d*) δ 3.84 (d, J = 6.6 Hz, 1H), 3.79 – 3.66 (m, 1H), 2.27 (s, 1H), 2.23 – 2.10 (m, 1H), 1.90 – 1.77 (m, 1H), 1.67 – 1.53 (m, 3H), 1.45 (s, 11H), 1.32 (d, J = 6.1 Hz, 3H), 1.30 – 1.16 (m, 2H), 0.92 (d, J = 7.6 Hz, 3H). ^{13}C NMR (101 MHz, $CDCl_3$) δ 155.2, 78.6, 60.7, 53.8, 39.3, 35.6, 30.1, 29.1, 28.6, 26.4, 19.6, 17.5, 14.8. HRMS (ESI) Calcd for $C_{15}H_{27}NNaO_2$ $[M+Na]^+$: 276.1934, found: 276.1934. Chiral GC-FID (50_5_5_100_0.2_140_5_200, *β*-Dex-325): t_1 = 171.43 min (minor enantiomer), t_2 = 174.21 min (major enantiomer).

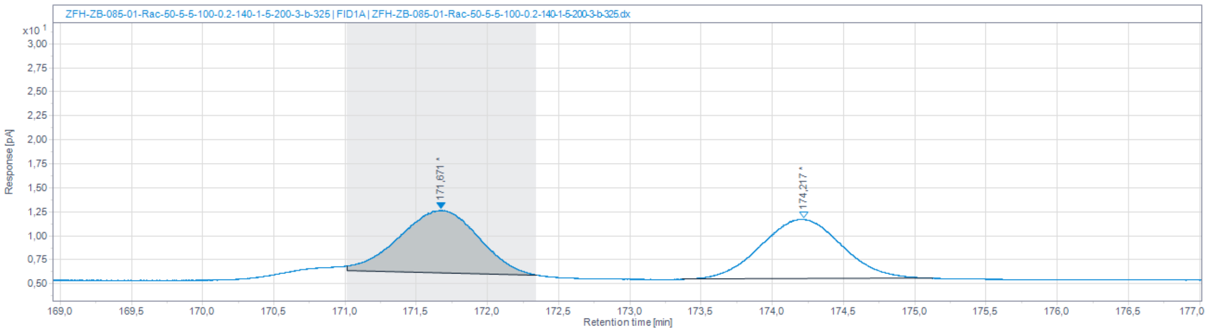

#	Name	Signal description	RT (min)	Area (pA·s)	Area%	Height (pA)	Height%	Amount	Concentration	Start time (min)	End time (min)
1		FID1A	171,671	251,994	50,672	6,515	51,16			171,015	172,346
2		FID1A	174,217	245,309	49,328	6,219	48,84			173,365	175,123

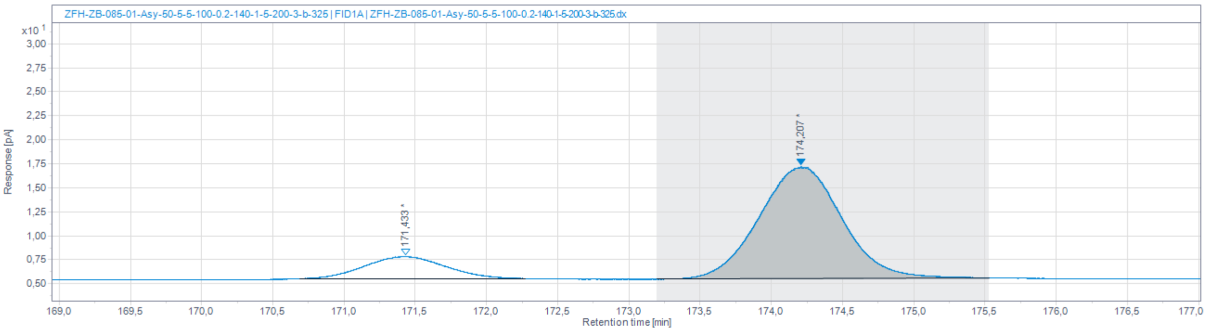

#	Name	Signal description	RT (min)	Area (pA·s)	Area%	Height (pA)	Height%	Amount	Concentration	Start time (min)	End time (min)
1		FID1A	171,433	90,510	16,137	2,287	16,40			170,688	172,270
2		FID1A	174,207	470,369	83,863	11,653	83,60			173,199	175,532

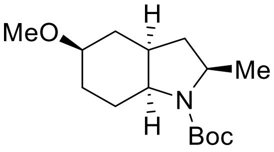

***tert*-butyl (2*R*,3a*R*,5*R*,7a*S*)-5-methoxy-2-methyloctahydro-1*H*-indole-1-carboxylate** (**5f**): The title compound was synthesized according to **GPD**. The product was isolated by column chromatography using a gradient of pentene:ethyl acetate (50:1) and obtained as a colorless oil (21 mg, 0.078 mmol, 78%, 81:19 dr, 93:7 er). The major diastereomer could not be separated by flash chromatography. Data for the mixture of all diastereomers: ^{1}H NMR (400 MHz, Chloroform-*d*) δ 3.93 – 3.53 (m, 2H), 3.43 (p, J = 3.3 Hz, 1H), 3.27 (s, 3H), 2.17 – 1.70 (m, 6H), 1.65 – 1.48 (m, 2H), 1.45 (s, 9H), 1.38 – 1.31 (m, 1H), 1.29 (d, J = 6.0 Hz, 3H). ^{13}C NMR (76 MHz, $CDCl_3$) δ 154.8, 78.6, 74.5, 57.7, 55.8, 53.5, 52.1, 38.3, 35.0, 29.0, 28.6, 26.7, 22.8. HRMS (ESI) Calcd for $C_{15}H_{27}NNaO_3$ $[M+Na]^+$: 292.1883, found: 292.1882. HPLC on Chiralpak IC-3 column; hexane:isopropanol = 99:1; flow rate = 0.5 mL/min; UV detection at 210 nm; t_R = 25.87 min (minor), t_R = 27.09 min (major).

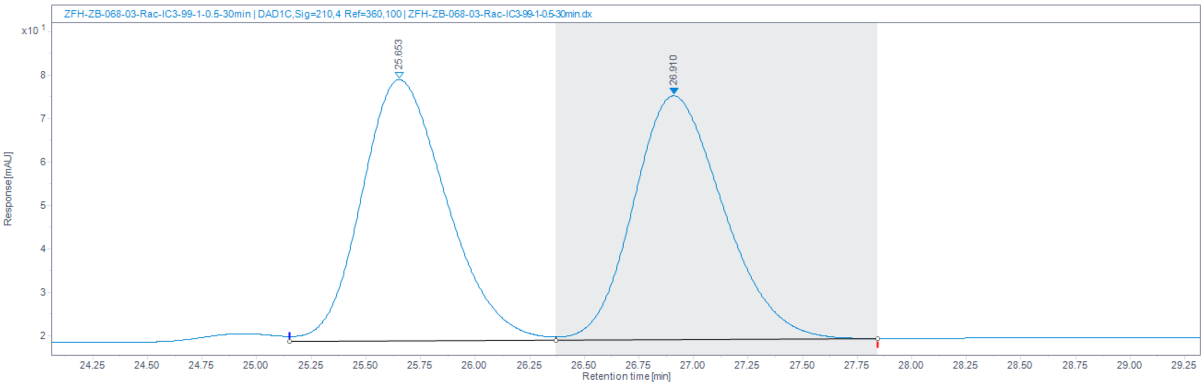

#	Name	Signal description	RT (min)	Area (mAU·s)	Area%	Height (mAU)	Height%	Amount	Concentration	Start time (min)	End time (min)
1		DAD1C,Sig=210,4 Ref=360,100	25.653	1630.476	50.287	60.180	51.74			25.150	26.369
2		DAD1C,Sig=210,4 Ref=360,100	26.910	1611.855	49.713	56.132	48.26			26.369	27.845

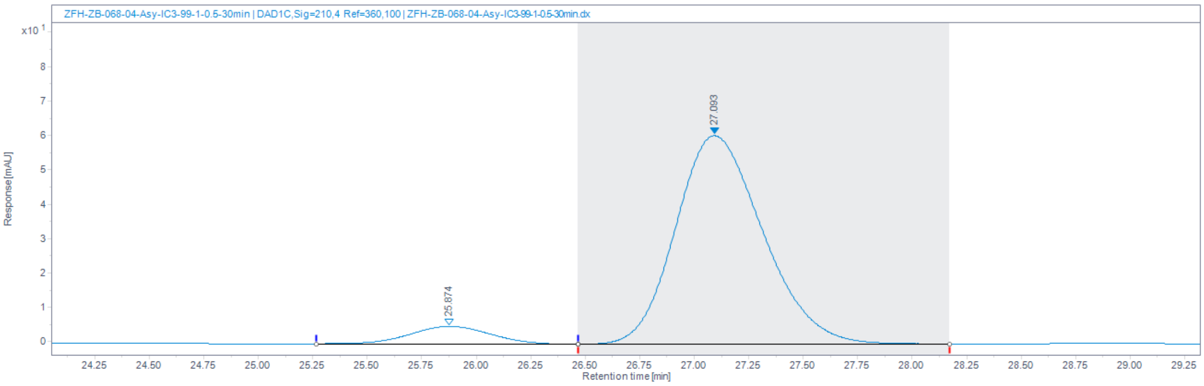

#	Name	Signal description	RT (min)	Area (mAU·s)	Area%	Height (mAU)	Height%	Amount	Concentration	Start time (min)	End time (min)
1		DAD1C,Sig=210,4 Ref=360,100	25.874	133.012	7.292	5.151	7.83			25.265	26.465
2		DAD1C,Sig=210,4 Ref=360,100	27.093	1691.092	92.708	60.621	92.17			26.466	28.172

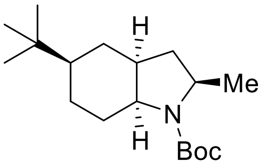

***tert*-butyl (2*R*,3a*S*,5*R*,7a*S*)-5-(*tert*-butyl)-2-methyloctahydro-1*H*-indole-1-carboxylate** (**5g**). The title compound was synthesized according to **GPD**. The product was isolated by column chromatography using a gradient of pentene:ethyl acetate (50:1) and obtained as a colorless oil (9 mg, 0.031 mmol, 31%, 75:25 dr, 88:12 er). The major diastereomer could not be separated by flash chromatography. Data for the mixture of all diastereomers: ^{1}H NMR (400 MHz, Chloroform-*d*) δ 3.75 (dt, *J* = 13.9, 6.7 Hz, 1H), 3.71 – 3.62 (m, 1H), 2.34 – 2.21 (m, 1H), 2.21 – 2.09 (m, 1H), 1.84 – 1.76 (m, 1H), 1.74 – 1.68 (m, 1H), 1.61 – 1.48 (m, 2H), 1.45 (s, 9H), 1.29 (d, *J* = 6.2 Hz, 5H), 1.19 – 1.13 (m, 1H), 1.04 – 0.92 (m, 1H), 0.81 (s, 9H). ^{13}C NMR (101 MHz, $CDCl_3$) δ 155.6, 78.7, 58.0, 54.2, 43.3, 40.2, 37.2, 32.6, 29.3, 28.5, 27.0, 26.2, 23.1, 21.9. HRMS (ESI) Calcd for $C_{18}H_{33}NNaO_2$ $[M+Na]^+$: 318.2404, found: 318.2402. HPLC on Chiralpak IC-3 column; hexane:isopropanol = 99:1; flow rate = 0.5 mL/min; UV detection at 210 nm; t_R = 12.80 min (minor), t_R = 15.90 min (major).

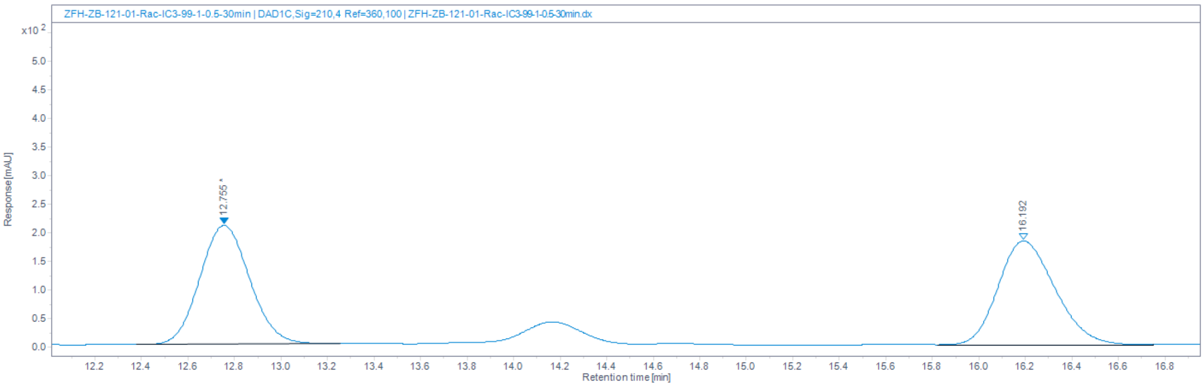

#	Name	Signal description	RT (min)	Area (mAU·s)	Area%	Height (mAU)	Height%	Amount	Concentration	Start time (min)	End time (min)
1		DAD1C,Sig=210,4 Ref=360,100	12.755	3090.287	49.903	207.644	53.30			12.377	13.256
2		DAD1C,Sig=210,4 Ref=360,100	16.192	3102.280	50.097	181.943	46.70			15.823	16.751

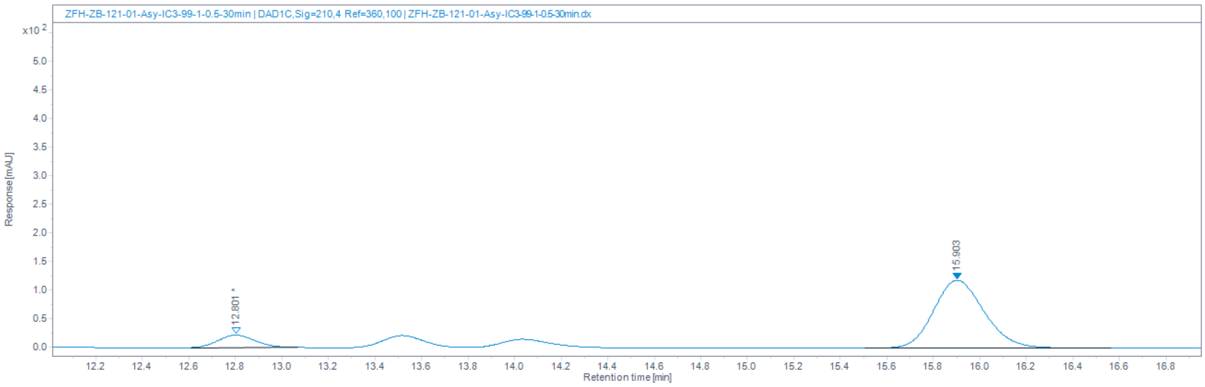

#	Name	Signal description	RT (min)	Area (mAU·s)	Area%	Height (mAU)	Height%	Amount	Concentration	Start time (min)	End time (min)
1		DAD1C,Sig=210,4 Ref=360,100	12.801	239.574	11.857	21.550	15.41			12.609	13.070
2		DAD1C,Sig=210,4 Ref=360,100	15.903	1781.029	88.143	118.327	84.59			15.504	16.564

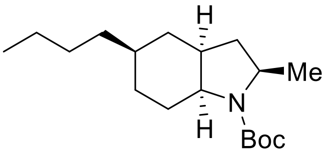

***tert*-butyl (2*R*,3a*S*,5*R*,7a*S*)-5-butyl-2-methyloctahydro-1*H*-indole-1-carboxylate** (**5h**). The title compound was synthesized according to **GPD**. The product was isolated by column chromatography using a gradient of pentene:ethyl acetate (50:1) and obtained as a colorless oil (24.5 mg, 0.083 mmol, 83%, 71:29 dr, 89:11 er). The major diastereomer could not be separated by flash chromatography. Data for the mixture of all diastereomers: ^{1}H NMR (400 MHz, Chloroform-*d*) δ 4.07 – 3.49 (m, 2H), 2.24 – 2.07 (m, 2H), 1.83 – 1.48 (m, 5H), 1.45 (s, 11H), 1.33 – 1.16 (m, 11H), 0.90 – 0.84 (m, 3H). ^{13}C NMR (101 MHz, $CDCl_3$) δ 155.5, 78.7, 58.4, 54.0, 39.5, 36.5, 36.3, 33.5, 32.9, 29.7, 28.5, 27.1, 25.4, 23.0, 22.9, 14.1. HRMS (ESI) Calcd for $C_{18}H_{33}NNaO_2$ $[M+Na]^+$: 318.2404, found: 318.2403. HPLC on Chiralpak IC-3 column; hexane:isopropanol = 99:1; flow rate = 0.5 mL/min; UV detection at 210 nm; t_R = 12.93 min (minor), t_R = 14.07 min (major).

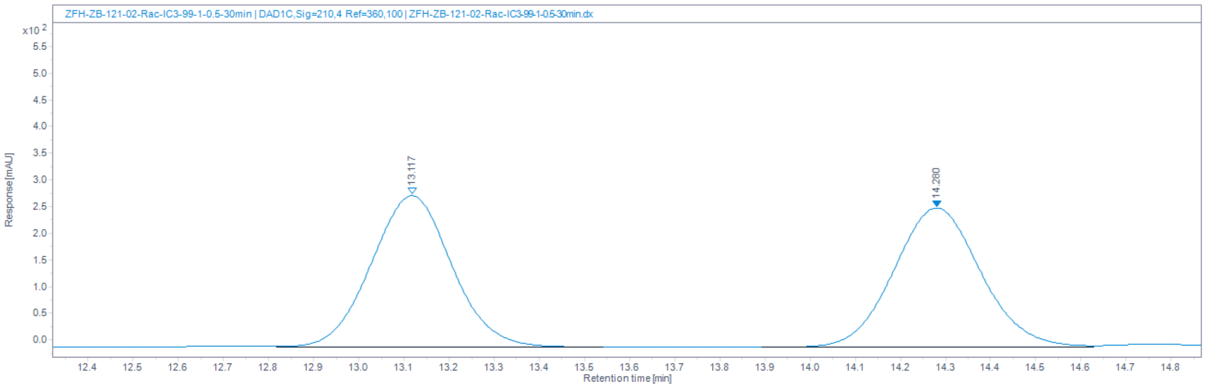

#	Name	Signal description	RT (min)	Area (mAU·s)	Area%	Height (mAU)	Height%	Amount	Concentration	Start time (min)	End time (min)
1		DAD1C,Sig=210,4 Ref=360,100	13.117	3548.312	50.230	284.492	52.12			12.817	13.541
2		DAD1C,Sig=210,4 Ref=360,100	14.280	3515.760	49.770	261.308	47.88			13.891	14.629

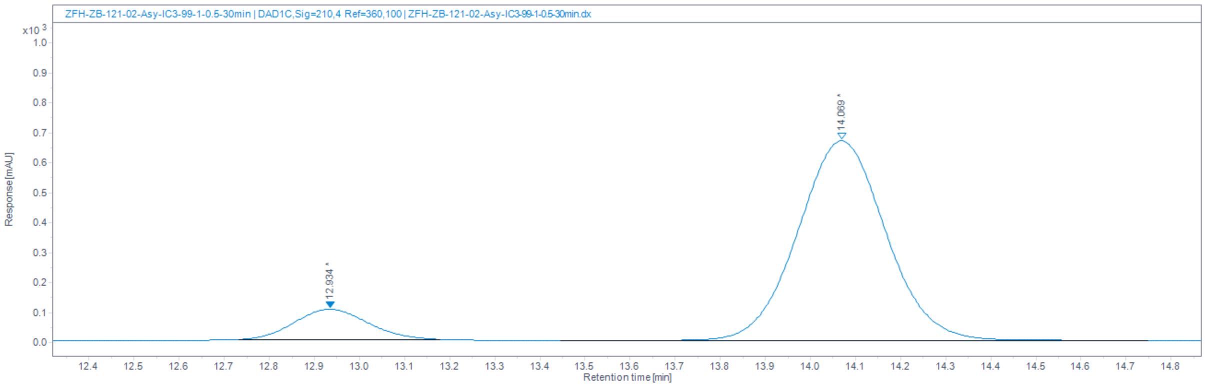

#	Name	Signal description	RT (min)	Area (mAU·s)	Area%	Height (mAU)	Height%	Amount	Concentration	Start time (min)	End time (min)
1		DAD1C,Sig=210,4 Ref=360,100	12.934	1137.071	11.147	100.458	13.11			12.733	13.178
2		DAD1C,Sig=210,4 Ref=360,100	14.069	9063.754	88.853	665.993	86.89			13.445	14.749

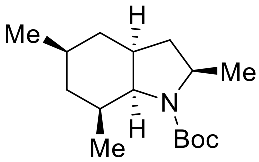

tert-butyl (2*R*,3a*S*,5*R*,7*S*,7a*S*)-2,5,7-trimethyloctahydro-1H-indole-1-carboxylate (**5i**). The title compound was synthesized according to **GPD**. The product was isolated by column chromatography using a gradient of pentene:ethyl acetate (50:1) and obtained as a colorless oil (17 mg, 0.064 mmol, 64%, 80:20 dr, 98:2 er). The major diastereomer could not be separated by flash chromatography. Data for the mixture of all diastereomers: ^{1}H NMR (400 MHz, Chloroform-*d*) δ 3.87 (dd, *J* = 9.4, 6.2 Hz, 1H), 3.79 – 3.65 (m, 1H), 2.38 – 2.24 (m, 2H), 2.25 – 2.13 (m, 1H), 2.02 – 1.91 (m, 1H), 1.78 – 1.70 (m, 1H), 1.68 – 1.53 (m, 2H), 1.45 (s, 9H), 1.35 – 1.24 (m, 4H), 1.10 – 0.99 (m, 1H), 0.93 (d, *J* = 7.6 Hz, 3H), 0.91 – 0.88 (m, 3H). ^{13}C NMR (101 MHz, $CDCl_3$) δ 155.8, 78.8, 60.6, 54.2, 42.2, 37.0, 36.6, 35.2, 29.3, 28.5, 27.3, 23.5, 21.1, 21.0. HRMS (ESI) Calcd for $C_{16}H_{29}NNaO_2$ $[M+Na]^+$: 290.2091, found: 290.2090. Chiral GC-FID (50_5_5_100_0.2_140_5_200, *β*-Dex-325): t_1 = 247.11 min (minor enantiomer), t_2 = 249.17 min (major enantiomer).

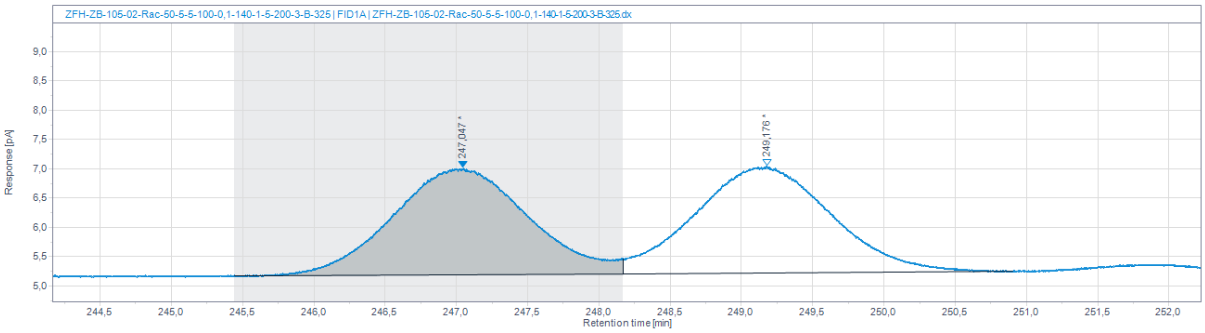

#	Name	Signal description	RT (min)	Area (pA·s)	Area%	Height (pA)	Height%	Amount	Concentration	Start time (min)	End time (min)
1		FID1A	247,047	120,270	50,027	1,809	49,96			245,446	248,173
2		FID1A	249,176	120,138	49,973	1,812	50,04			248,173	250,902

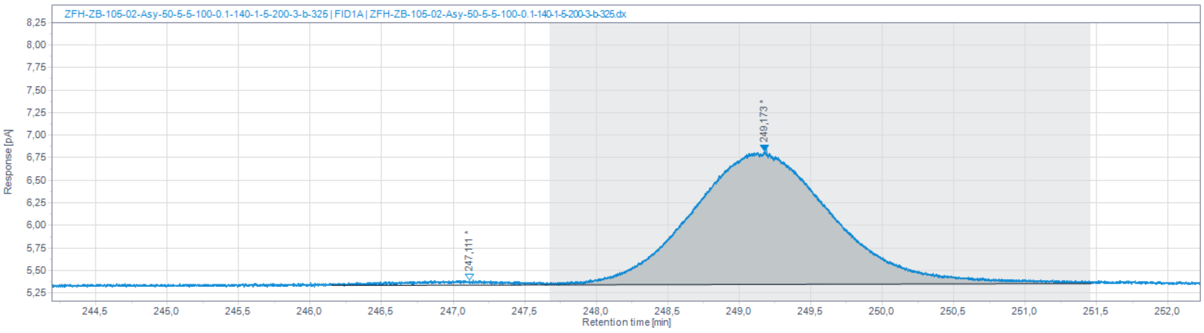

#	Name	Signal description	RT (min)	Area (pA·s)	Area%	Height (pA)	Height%	Amount	Concentration	Start time (min)	End time (min)
1		FID1A	247,111	2,239	2,201	0,043	2,83			246,153	247,678
2		FID1A	249,173	99,519	97,799	1,462	97,17			247,678	251,457

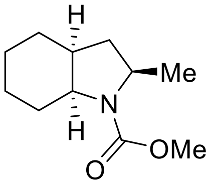

methyl (2*R*,3a*S*,7a*S*)-2-methyloctahydro-1*H*-indole-1-carboxylate (**5j**). The title compound was synthesized according to **GPD**. The product was isolated by column chromatography using a gradient of pentene:ethyl acetate (50:1) and obtained as a colorless oil (15.6 mg, 0.079 mmol, 79%, 82:18 dr, 91:9 er). The major diastereomer could not be separated by flash chromatography. Data for the mixture of all diastereomers: ^{1}H NMR (400 MHz, Chloroform-*d*) δ 4.00 – 3.58 (m, 3H), 2.49 – 2.10 (m, 1H), 2.09 – 1.85 (m, 1H), 1.75 – 1.43 (m, 3H), 1.40 – 0.95 (m, 4H). ^{13}C NMR (101 MHz, $CDCl_3$) δ 155.2, 58.1, 57.8, 57.5, 57.3, 53.7, 53.1, 52.4, 52.0, 51.8, 51.7, 36.3, 35.5, 34.6, 34.3, 33.7, 33.3, 29.7, 29.2, 27.8, 27.0, 26.0, 25.9, 25.89, 24.1, 23.8, 23.6, 22.9, 21.9, 21.6, 20.9, 20.64, 20.6, 20.5. HRMS (ESI) Calcd for $C_{11}H_{19}NNaO_2$ $[M+Na]^+$: 220.1308, found: 220.1306. Chiral GC-FID (50_5_5_100_0.2_140_5_200, *β*-Dex-325): t_1 = 126.65 min (minor enantiomer), t_2 = 136.10 min (major enantiomer).

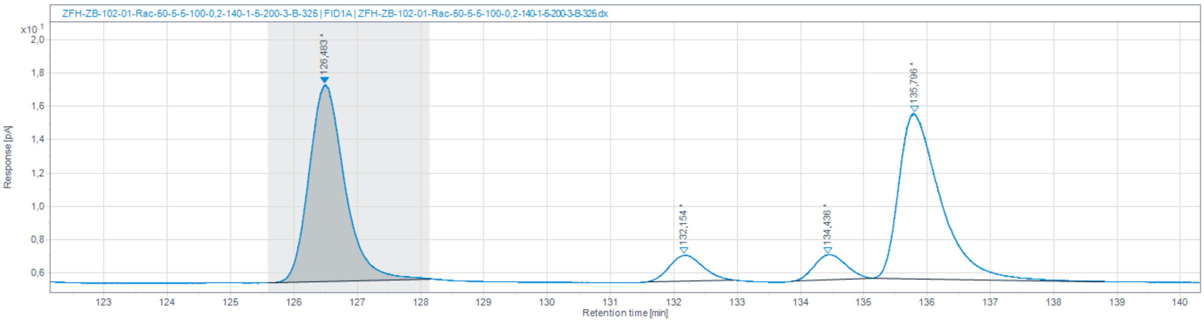

#	Name	Signal description	RT (min)	Area (pA·s)	Area%	Height (pA)	Height%	Amount	Concentration	Start time (min)	End time (min)
1		FID1A	126,483	461,095	45,141	11,830	47,70			125,588	128,150
2		FID1A	132,154	56,410	5,522	1,545	6,23			131,585	132,946
3		FID1A	134,436	51,647	5,056	1,509	6,08			133,884	135,141
4		FID1A	135,796	452,310	44,281	9,917	39,99			135,141	138,799

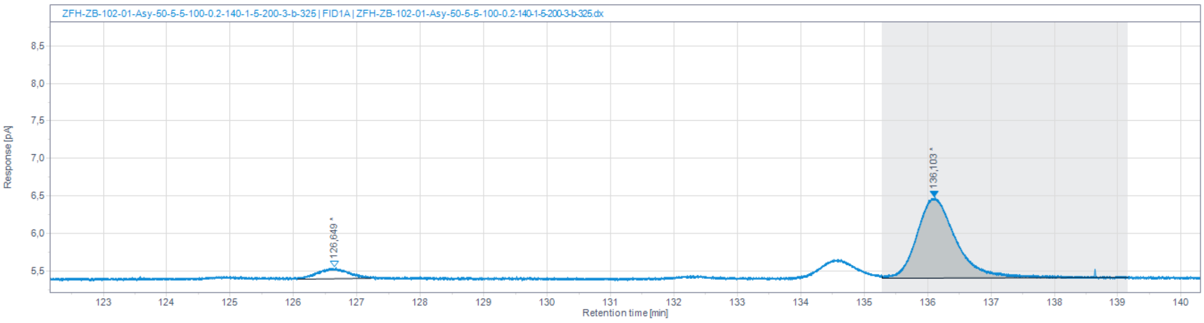

#	Name	Signal description	RT (min)	Area (pA·s)	Area%	Height (pA)	Height%	Amount	Concentration	Start time (min)	End time (min)
1		FID1A	126,649	4,303	8,615	0,132	11,04			126,068	127,220
2		FID1A	136,103	45,640	91,385	1,064	88,96			135,283	139,159

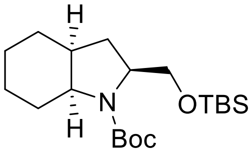

***tert*-butyl (2*S*,3a*S*,7a*S*)-2-(((*tert*-butyldimethylsilyl)oxy)methyl)octahydro-1*H*-indole-1-carboxylate** (**5k**). The title compound was synthesized according to **GPD**. The product was isolated by column chromatography using a gradient of pentene:ethyl acetate (50:1) and obtained as a colorless oil (34.5 mg, 0.094 mmol, 94%, 94:6 dr, 59:41 er). The major diastereomer could not be separated by flash chromatography. Data for the mixture of all diastereomers: ^{1}H NMR (400 MHz, Chloroform-*d*) δ 4.12 – 3.43 (m, 4H), 2.30 – 1.96 (m, 2H), 1.87 – 1.56 (m, 5H), 1.44 (s, 10H), 1.37 – 1.20 (m, 2H), 1.18 – 1.02 (m, 1H), 0.89 (s, 9H), 0.04 (d, J = 3.8 Hz, 6H). ^{13}C NMR (101 MHz, $CDCl_3$) δ 154.5, 78.9, 78.5, 64.9, 62.6, 58.9, 57.8, 57.7, 36.0, 35.7, 31.2, 29.4, 28.6, 28.5, 26.2, 25.9, 24.1, 20.6, 18.3, -5.3, -5.4.HRMS (ESI) Calcd for $C_{20}H_{39}NNaO_3Si$ $[M+Na]^+$: 392.2591, found: 392.2585. HPLC on Chiralpak IG-3 column; hexane:isopropanol = 99:1; flow rate = 0.5 mL/min; UV detection at 215 nm; t_R = 8.10 min (minor), t_R = 8.44 min (major).

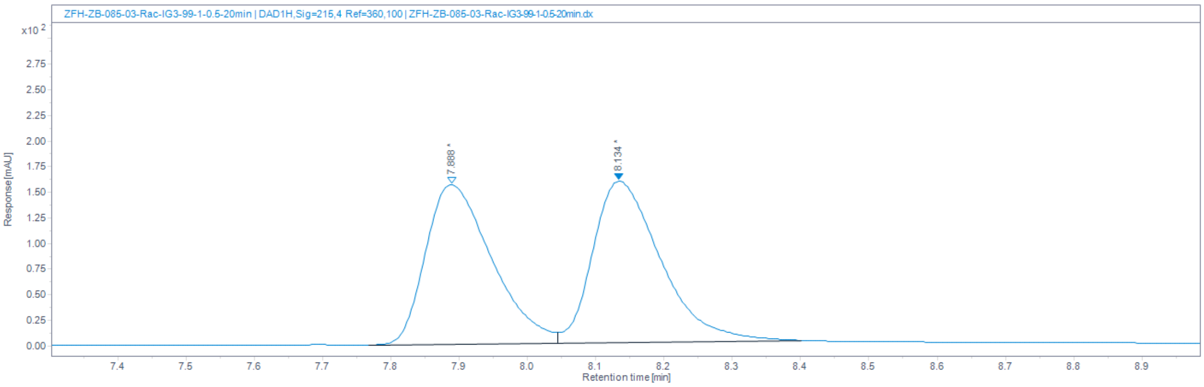

#	Name	Signal description	RT (min)	Area (mAU·s)	Area%	Height (mAU)	Height%	Amount	Concentration	Start time (min)	End time (min)
1		DAD1H,Sig=215,4 Ref=360,100	7.888	1049.097	49.099	155.809	49.74			7.767	8.044
2		DAD1H,Sig=215,4 Ref=360,100	8.134	1087.594	50.901	157.434	50.26			8.044	8.401

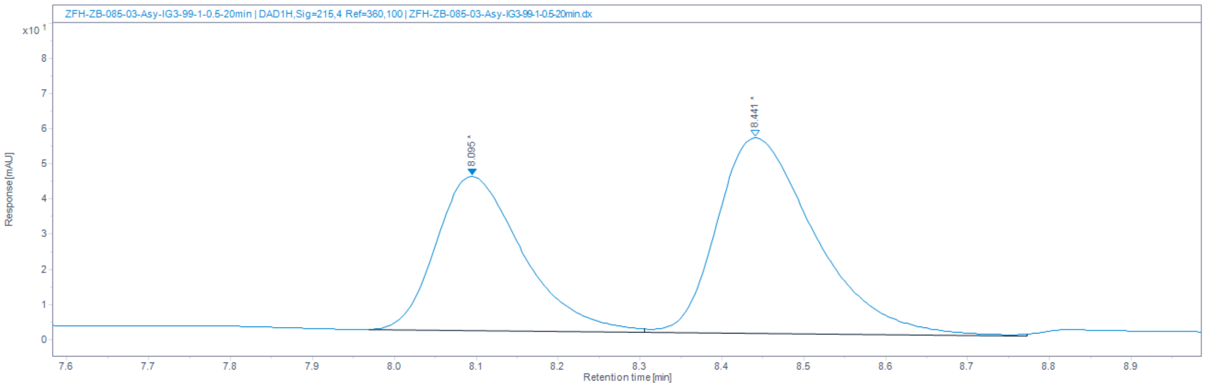

#	Name	Signal description	RT (min)	Area (mAU·s)	Area%	Height (mAU)	Height%	Amount	Concentration	Start time (min)	End time (min)
1		DAD1H,Sig=215,4 Ref=360,100	8.095	322.811	40.973	43.894	44.08			7.968	8.306
2		DAD1H,Sig=215,4 Ref=360,100	8.441	465.054	59.027	55.686	55.92			8.306	8.773

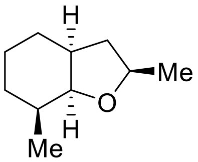

(2*R*,3a*S*,7*S*,7a*S*)-2,7-dimethyloctahydrobenzofuran (**7a**). The title compound was synthesized according to **GPD**. The product was isolated by column chromatography using a gradient of pentene:ethyl acetate (100:1) and obtained as a colorless volatile oil (14 mg, 0.091 mmol, 91%, 92:8 dr, 99:1 er). The major diastereomer could not be separated by flash chromatography. Data for the mixture of all diastereomers: ^{1}H NMR (400 MHz, Chloroform-*d*) δ 4.02 – 3.89 (m, 1H), 3.58 (t, *J* = 3.5 Hz, 1H), 2.25 – 2.13 (m, 1H), 1.96 – 1.85 (m, 1H), 1.68 – 1.61 (m, 2H), 1.54 – 1.45 (m, 2H), 1.44 – 1.36 (m, 2H), 1.31 (d, *J* = 6.3 Hz, 3H), 1.30 – 1.22 (m, 2H), 1.23 – 1.16 (m, 2H), 1.15 – 1.10 (m, 1H), 1.05 (d, *J* = 7.0 Hz, 3H). ^{13}C NMR (101 MHz, $CDCl_3$) δ 82.1, 73.5, 39.7, 38.8, 33.9, 29.9, 28.6, 25.5, 23.0, 19.2. ^{1}H NMR, ^{13}C NMR data were consistent with those reported in literatures[2g]. Chiral GC-FID (50_5_5_100_0.2_140_5_200, *β*-Dex-325): t_1 = 24.61 min (minor enantiomer), t_2 = 25.48 min (major enantiomer).

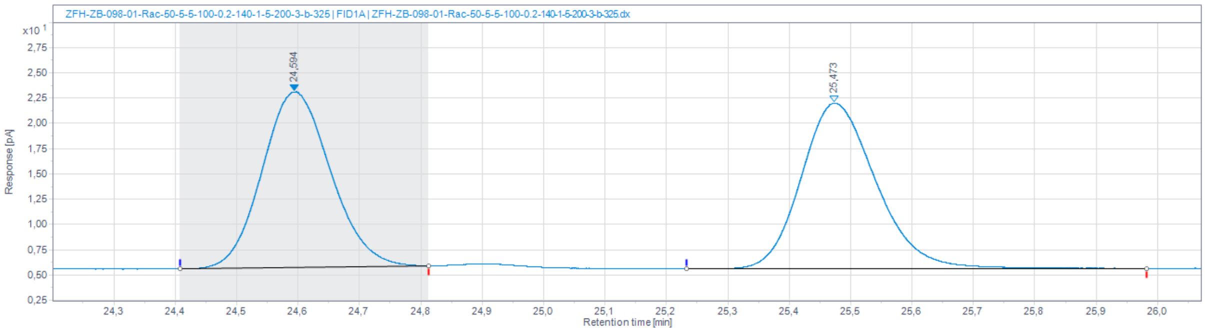

#	Name	Signal description	RT (min)	Area (pA·s)	Area%	Height (pA)	Height%	Amount	Concentration	Start time (min)	End time (min)
1		FID1A	24,594	135,079	49,204	17,369	51,50			24,408	24,813
2		FID1A	25,473	139,450	50,796	16,357	48,50			25,233	25,983

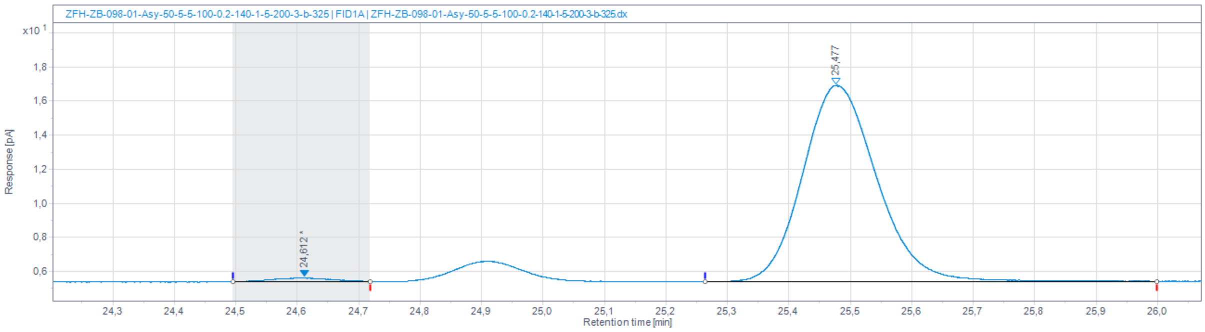

#	Name	Signal description	RT (min)	Area (pA·s)	Area%	Height (pA)	Height%	Amount	Concentration	Start time (min)	End time (min)
1		FID1A	24,612	1,286	1,305	0,196	1,67			24,495	24,719
2		FID1A	25,477	97,269	98,695	11,518	98,33			25,263	25,999

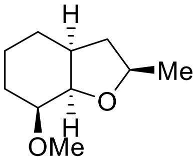

(2*R*,3a*S*,7*S*,7a*R*)-7-methoxy-2-methyloctahydrobenzofuran (**7b**). The title compound was synthesized according to **GPD**. The product was isolated by column chromatography using a gradient of pentene:ethyl acetate (50:1) and obtained as a volatile colorless oil (14 mg, 0.082 mmol, 82%, 88:12 dr, 98:2 er). The major diastereomer could not be separated by flash chromatography. Data for the mixture of all diastereomers: ^{1}H NMR (400 MHz, Chloroform-*d*) δ 4.11 – 3.98 (m, 1H), 3.93 (t, *J* = 3.6 Hz, 1H), 3.41 (s, 3H), 3.37 – 3.27 (m, 1H), 2.24 – 2.12 (m, 1H), 2.08 – 1.99 (m, 1H), 1.86 – 1.79 (m, 1H), 1.77 – 1.66 (m, 1H), 1.59 – 1.47 (m, 2H), 1.34 (d, *J* = 6.3 Hz, 3H), 1.26 – 1.18 (m, 3H). ^{13}C NMR (101 MHz, $CDCl_3$) δ 79.5, 78.3, 74.3, 56.3, 39.3, 39.1, 29.3, 25.1, 23.3, 22.8. ^{1}H NMR, ^{13}C NMR data were consistent with those reported in literatures[2g]. Chiral GC-FID (50_5_5_100_0.2_140_5_200, *β*-Dex-325): t_1 = 53.72 min (minor enantiomer), t_2 = 54.89 min (major enantiomer).

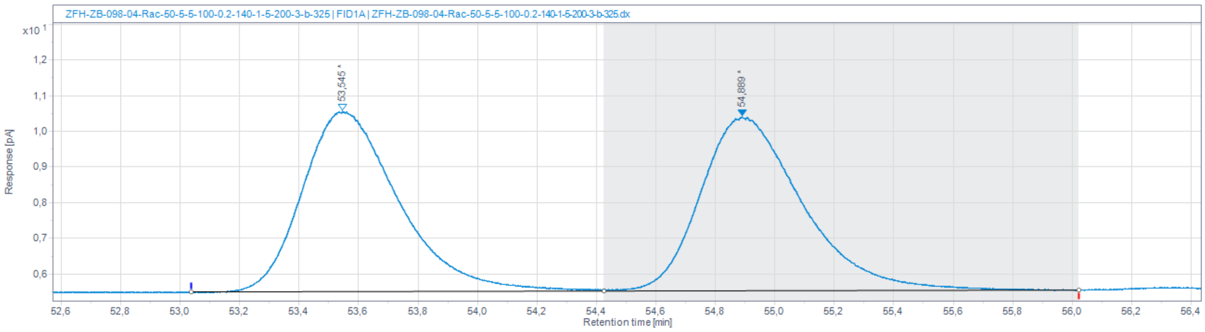

#	Name	Signal description	RT (min)	Area (pA·s)	Area%	Height (pA)	Height%	Amount	Concentration	Start time (min)	End time (min)
1		FID1A	53,545	114,101	49,623	5,050	50,93			53,038	54,425
2		FID1A	54,889	115,835	50,377	4,865	49,07			54,425	56,021

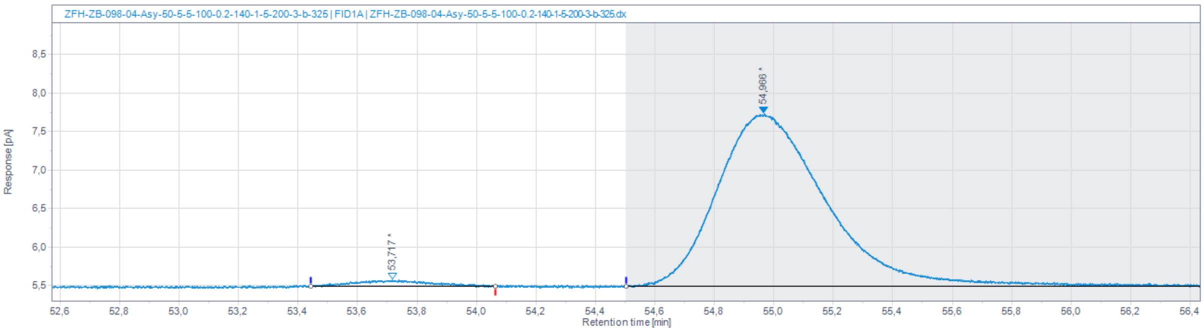

#	Name	Signal description	RT (min)	Area (pA·s)	Area%	Height (pA)	Height%	Amount	Concentration	Start time (min)	End time (min)
1		FID1A	53,717	1,264	2,165	0,073	3,14			53,442	54,062
2		FID1A	54,966	57,101	97,835	2,236	96,86			54,505	56,525

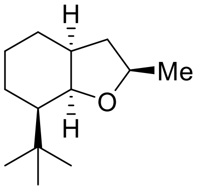

(2*R*,3a*S*,7*R*,7a*R*)-7-(*tert*-butyl)-2-methyloctahydrobenzofuran (**7c**). The title compound was synthesized according to **GPD**. The product was isolated by column chromatography using a gradient of pentene:ethyl acetate (100:1) and obtained as a volatile colorless oil (16.5 mg, 0.084 mmol, 84%, 91:9 dr, 96:4 er). The major diastereomer could not be separated by flash chromatography. Data for the mixture of all diastereomers: ^{1}H NMR (400 MHz, Chloroform-*d*) δ 3.96 – 3.84 (m, 1H), 3.78 (t, *J* = 3.2 Hz, 1H), 2.15 – 2.03 (m, 1H), 1.92 – 1.81 (m, 1H), 1.76 – 1.65 (m, 1H), 1.56 – 1.43 (m, 2H), 1.44 – 1.29 (m, 1H), 1.27 (d, *J* = 6.3 Hz, 3H), 1.25 – 1.11 (m, 3H), 1.03 (dd, *J* = 12.4, 5.5 Hz, 1H), 0.95 (s, 9H). ^{13}C NMR (101 MHz, $CDCl_3$) δ 79.0, 73.8, 48.8, 40.1, 39.2, 33.0, 30.4, 28.5, 26.3, 23.2, 21.7. ^{1}H NMR, ^{13}C NMR data were consistent with those reported in literatures[2g]. Chiral GC-FID (50_5_1_100_0.05_200, Hydrodex *β*-6TBDM): t_1 = 71.36 min (major enantiomer), t_2 = 72.11 min (minor enantiomer).

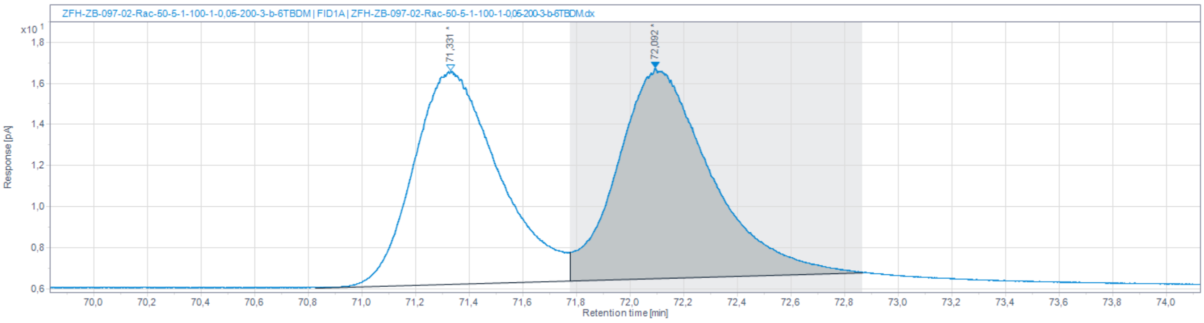

#	Name	Signal description	RT (min)	Area (pA·s)	Area%	Height (pA)	Height%	Amount	Concentration	Start time (min)	End time (min)
1		FID1A	71,331	229,099	48,164	10,330	50,36			70,827	71,776
2		FID1A	72,092	246,570	51,836	10,183	49,64			71,776	72,865

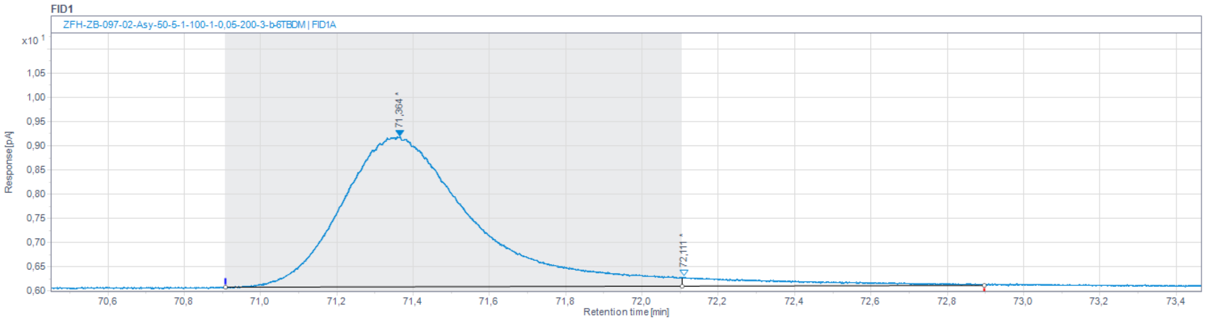

#	Name	Signal description	RT (min)	Area (pA·s)	Area%	Height (pA)	Height%	Amount	Concentration	Start time (min)	End time (min)
1		FID1A	71,364	73,740	95,679	3,088	94,65			70,908	72,106
2		FID1A	72,111	3,330	4,321	0,175	5,35			72,106	72,896

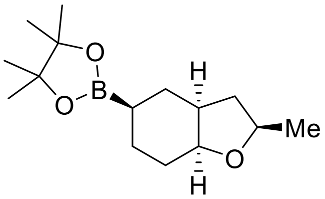

<u>4,4,5,5-tetramethyl-2-((2*R*,3a*S*,5*R*,7a*S*)-2-methyloctahydrobenzofuran-5-yl)-1,3,2-dioxaborolane</u> (**7d**). The title compound was synthesized according to **GPD**. The product was isolated by column chromatography using a gradient of pentene:ethyl acetate (100:1) and obtained as a colorless oil (19 mg, 0.072 mmol, 72%, 75:25 dr, 97:3 er). The major diastereomer could not be separated by flash chromatography. Data for the mixture of all diastereomers: ^{1}H NMR (400 MHz, Chloroform-*d*) δ 4.02 – 3.92 (m, 1H), 3.74 (q, J = 3.5 Hz, 1H), 2.22 – 2.10 (m, 1H), 2.08 – 1.97 (m, 1H), 1.97 – 1.85 (m, 1H), 1.70 – 1.46 (m, 5H), 1.30 (d, J = 6.3 Hz, 3H), 1.22 (d, J = 2.3 Hz, 15H), 1.18 – 1.10 (m, 2H), 0.89 – 0.78 (m, 1H). ^{13}C NMR (101 MHz, $CDCl_3$) δ 82.9, 82.8, 77.373.8, 40.0, 38.6, 31.3, 28.8, 24.7, 24.7, 22.7, 22.1. ^{11}B NMR (128 MHz, Chloroform-*d*) δ 33.67. ^{1}H NMR, ^{13}C NMR, and ^{11}B NMR data were consistent with those reported in the literatures[2g]. Chiral GC-FID (50_5_5_80_1.0_200, GTA-0701-37): t_1 = 97.63 min (major enantiomer), t_2 = 99.08 min (minor enantiomer).

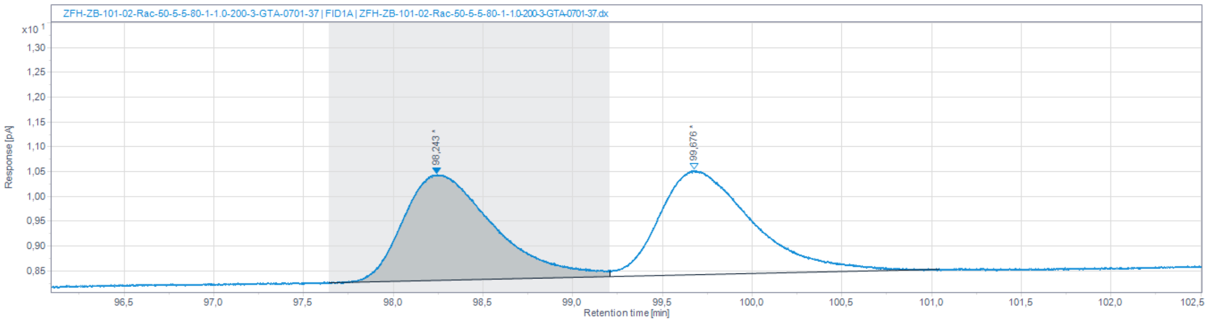

#	Name	Signal description	RT (min)	Area (pA·s)	Area%	Height (pA)	Height%	Amount	Concentration	Start time (min)	End time (min)
1		FID1A	98,243	74,581	49,751	2,119	50,25			97,645	99,208
2		FID1A	99,676	75,328	50,249	2,098	49,75			99,208	101,043

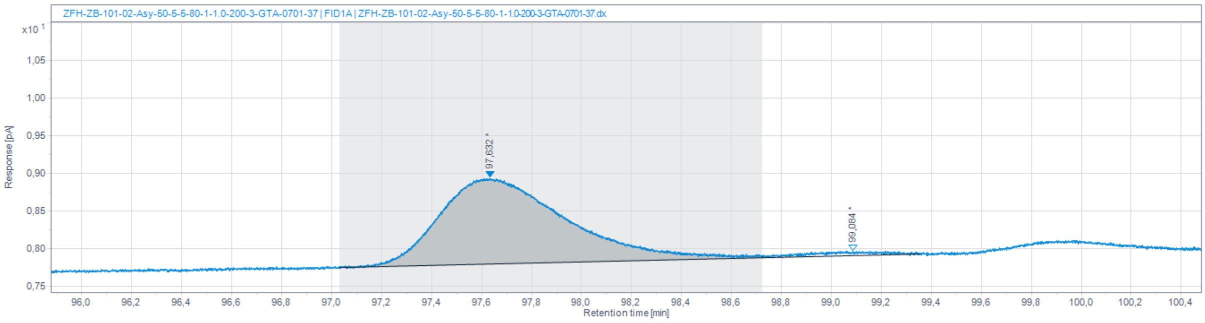

#	Name	Signal description	RT (min)	Area (pA·s)	Area%	Height (pA)	Height%	Amount	Concentration	Start time (min)	End time (min)
1		FID1A	97,632	39,675	97,265	1,125	95,72			97,033	98,724
2		FID1A	99,084	1,115	2,735	0,050	4,28			98,724	99,353

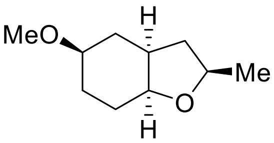

<u>(2*R*,3a*R*,5*R*,7a*S*)-5-methoxy-2-methyloctahydrobenzofuran</u> (**7e**). The title compound was synthesized according to **GPD**. The product was isolated by column chromatography using a gradient of pentene:ethyl acetate (50:1) and obtained as a colorless volatile oil (16 mg, 0.094 mmol, 94%, 93:7 dr, 91.5:8.5 er). The major diastereomer could not be separated by flash chromatography. Data for the mixture of all diastereomers: ^{1}H NMR (400 MHz, Chloroform-*d*) δ 4.07 – 3.94 (m, 1H), 3.76 – 3.64 (m, 1H), 3.34 (s, 3H), 3.16 – 3.04 (m, 1H), 2.31 – 2.19 (m, 1H), 2.15 – 2.00 (m, 2H), 1.92 – 1.81 (m, 1H), 1.85 – 1.74 (m, 1H), 1.67 – 1.42 (m, 3H), 1.31 (d, J = 6.3 Hz, 3H), 1.30 – 1.21 (m, 2H). ^{13}C NMR (101 MHz, $CDCl_3$) δ 78.3, 76.5, 74.0, 55.5, 40.0, 37.8, 35.4, 26.2, 26.0, 22.7. ^{1}H NMR, ^{13}C NMR data were consistent with those reported in literatures[2g]. Chiral GC-FID (50_5_5_100_0.2_140_5_200, *β*-Dex-325): t_1 = 126.65 min (minor enantiomer), t_2 = 136.10 min (major enantiomer).

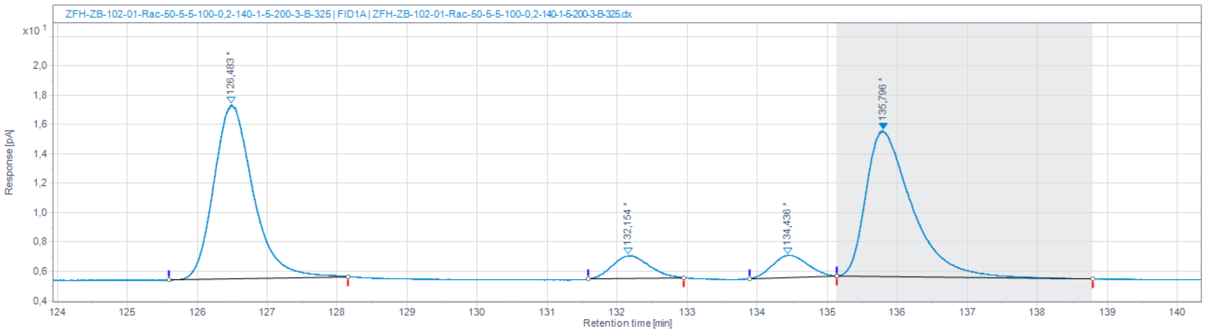

#	Name	Signal description	RT (min)	Area (pA·s)	Area%	Height (pA)	Height%	Amount	Concentration	Start time (min)	End time (min)
1		FID1A	126,483	461,095	45,141	11,830	47,70			125,588	128,150
2		FID1A	132,154	56,410	5,522	1,545	6,23			131,585	132,946
3		FID1A	134,436	51,647	5,056	1,509	6,08			133,884	135,141
4		FID1A	135,796	452,310	44,281	9,917	39,99			135,141	138,799

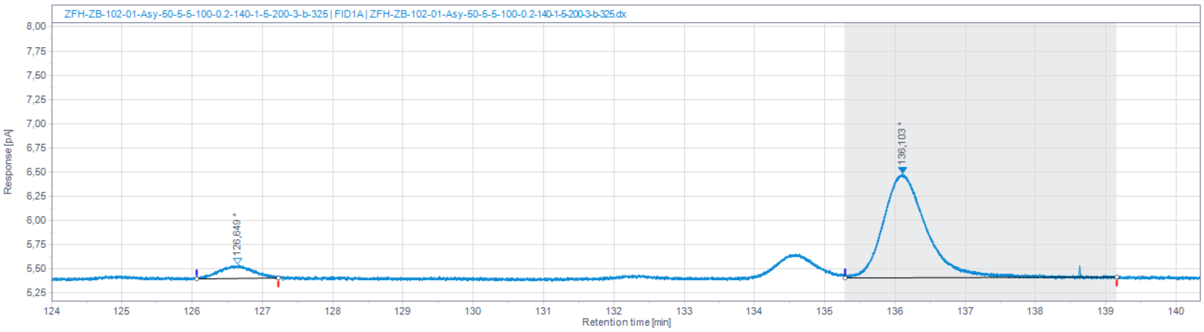

#	Name	Signal description	RT (min)	Area (pA·s)	Area%	Height (pA)	Height%	Amount	Concentration	Start time (min)	End time (min)
1		FID1A	126,649	4,303	8,615	0,132	11,04			126,068	127,220
2		FID1A	136,103	45,640	91,385	1,064	88,96			135,283	139,159

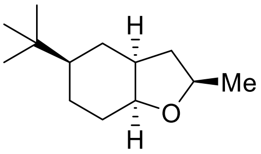

(2*R*,3a*S*,5*R*,7a*S*)-5-(*tert*-butyl)-2-methyloctahydrobenzofuran (**7f**). The title compound was synthesized according to **GPD**. The product was isolated by column chromatography using a gradient of pentene:ethyl acetate (100:1) and obtained as a colorless volatile oil (18 mg, 0.091 mmol, 91%, 94:6 dr, 97.5:2.5 er). The major diastereomer could not be separated by flash chromatography. Data for the mixture of all diastereomers: ^{1}H NMR (400 MHz, Chloroform-*d*) δ 4.04 – 3.92 (m, 1H), 3.71 (q, J = 3.7 Hz, 1H), 2.31 – 2.18 (m, 1H), 2.13 – 2.03 (m, 1H), 2.00 – 1.88 (m, 1H), 1.59 – 1.47 (m, 3H), 1.31 (d, J = 6.2 Hz, 3H), 1.27 – 1.21 (m, 1H), 1.18 – 1.12 (m, 1H), 1.00 – 0.91 (m, 2H), 0.84 (s, 9H). ^{13}C NMR (101 MHz, $CDCl_3$) δ 77.2, 73.8, 47.0, 40.5, 39.2, 32.3, 31.5, 28.6, 27.4, 22.7, 21.7. ^{1}H NMR, ^{13}C NMR data were consistent with those reported in literatures[2g]. Chiral GC-FID (50_5_5_100_0.2_140_5_200, β-Dex-325): t_1 = 62.22 min (minor enantiomer), t_2 = 65.28 min (major enantiomer).

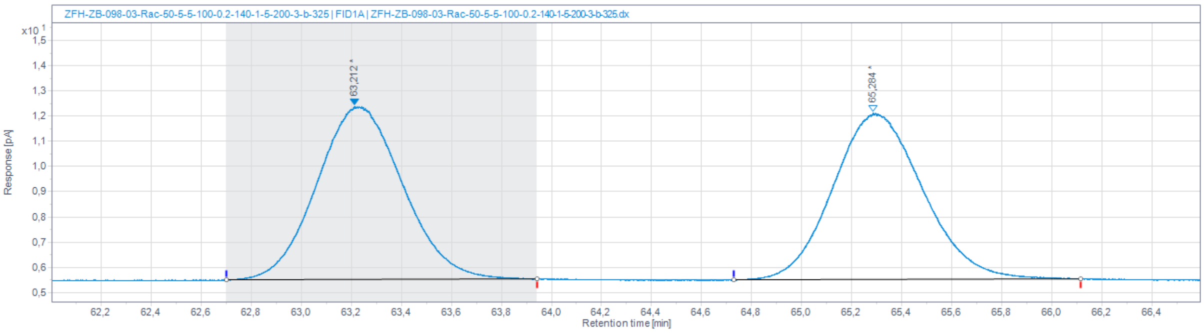

#	Name	Signal description	RT (min)	Area (pA·s)	Area%	Height (pA)	Height%	Amount	Concentration	Start time (min)	End time (min)
1		FID1A	63,212	163,907	49,770	6,853	50,94			62,699	63,942
2		FID1A	65,284	165,422	50,230	6,599	49,06			64,729	66,114

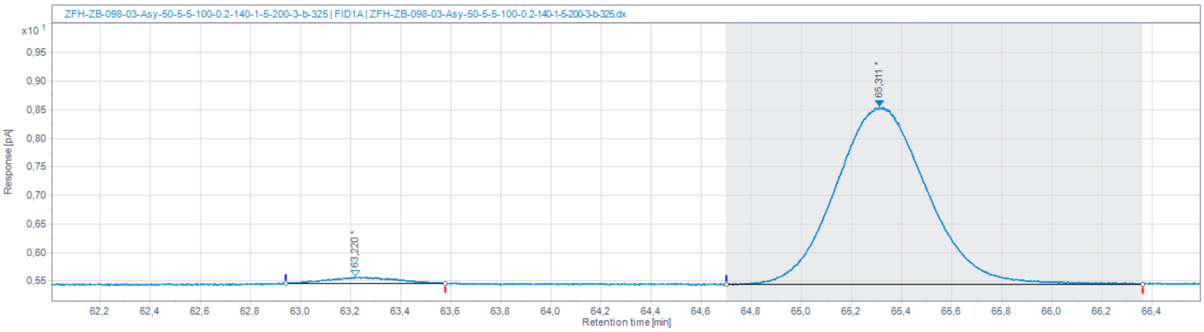

#	Name	Signal description	RT (min)	Area (pA·s)	Area%	Height (pA)	Height%	Amount	Concentration	Start time (min)	End time (min)
1		FID1A	63,220	1,964	2,428	0,104	3,25			62,941	63,576
2		FID1A	65,311	78,916	97,572	3,095	96,75			64,702	66,363

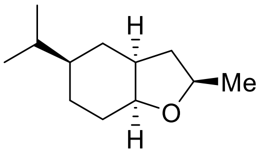

(2*R*,3a*S*,5*R*,7a*S*)-5-isopropyl-2-methyloctahydrobenzofuran (**7g**). The title compound was synthesized according to **GPD**. The product was isolated by column chromatography using a gradient of pentene:ethyl acetate (100:1) and obtained as a colorless volatile oil (14 mg, 0.077 mmol, 77%, 93:7 dr, 96.5:3.5 er). The major diastereomer could not be separated by flash chromatography. Data for the mixture of all diastereomers: ^{1}H NMR (400 MHz, Chloroform-*d*) δ 4.05 – 3.92 (m, 1H), 3.72 (q, J = 3.7 Hz, 1H), 2.32 – 2.17 (m, 1H), 2.14 – 2.03 (m, 1H), 2.00 – 1.90 (m, 1H), 1.58 – 1.38 (m, 4H), 1.31 (d, J = 6.2 Hz, 3H), 1.27 – 1.21 (m, 2H), 1.14 (dd, J = 12.5, 5.6 Hz, 1H), 1.07 – 0.93 (m, 2H), 0.85 (d, J = 6.8 Hz, 6H). ^{13}C NMR (101 MHz, $CDCl_3$) δ 77.5, 73.8, 42.9, 40.4, 38.8, 33.8, 32.7, 28.2, 23.8, 22.8, 19.6. ^{1}H NMR, ^{13}C NMR data were consistent with those reported in literatures[2g]. Chiral GC-FID (50_5_5_100_0.2_140_5_200, β-Dex-325): t_1 = 48.54 min (minor enantiomer), t_2 = 49.22 min (major enantiomer).

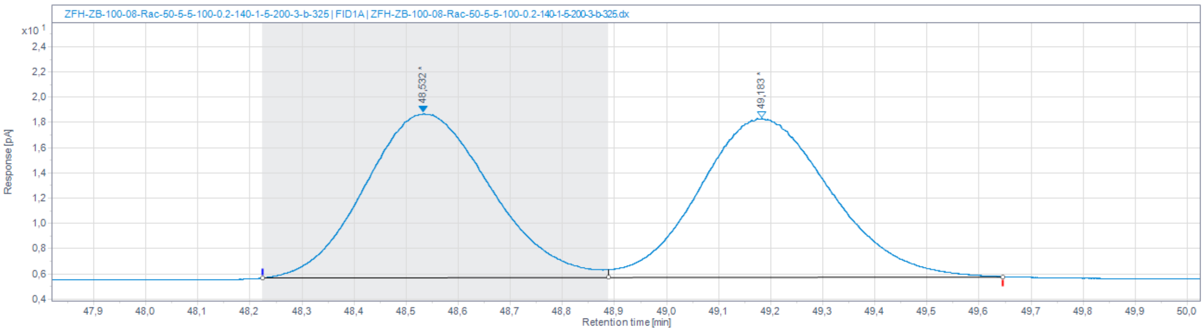

#	Name	Signal description	RT (min)	Area (pA·s)	Area%	Height (pA)	Height%	Amount	Concentration	Start time (min)	End time (min)
1		FID1A	48,532	218,169	49,718	12,999	50,87			48,224	48,888
2		FID1A	49,183	220,647	50,282	12,557	49,13			48,888	49,645

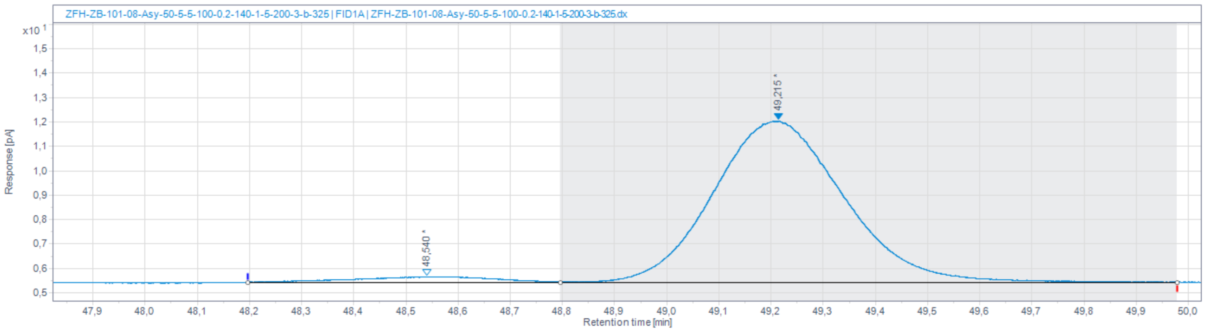

#	Name	Signal description	RT (min)	Area (pA·s)	Area%	Height (pA)	Height%	Amount	Concentration	Start time (min)	End time (min)
1		FID1A	48,540	4,153	3,495	0,227	3,33			48,197	48,795
2		FID1A	49,215	114,679	96,505	6,591	96,67			48,795	49,979

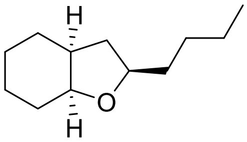

(2*R*,3a*S*,7a*S*)-2-butyloctahydrobenzofuran (**7h**). The title compound was synthesized according to **GPD**. The product was isolated by column chromatography using a gradient of pentene:ethyl acetate (100:1) and obtained as a volatile colorless oil (14.5 mg, 0.080 mmol, 80%, 93:7 dr, 95.5:4.5 er). The major diastereomer could not be separated by flash chromatography. Data for the mixture of all diastereomers: ^{1}H NMR (400 MHz, Chloroform-*d*) δ 3.87 – 3.80 (m, 1H), 3.77 (q, J = 4.6 Hz, 1H), 2.09 – 2.01 (m, 2H), 1.85 – 1.76 (m, 1H), 1.73 – 1.62 (m, 2H), 1.61 – 1.45 (m, 6H), 1.43 – 1.18 (m, 11H), 0.90 (t, J = 7.0 Hz, 3H). ^{13}C NMR (101 MHz, $CDCl_3$) δ 78.5, 77.1, 37.7, 37.5, 37.2, 29.0, 28.9, 28.6, 23.9, 22.8, 21.4, 14.1. HRMS (EI) Calcd for $C_{12}H_{22}O$ $[M]^+$: 182.1665, found: 182.1666. Chiral GC-FID (50_5_5_100_0.2_140_5_200, *β*-Dex-120): t_1 = 66.66 min (minor enantiomer), t_2 = 67.51 min (major enantiomer).

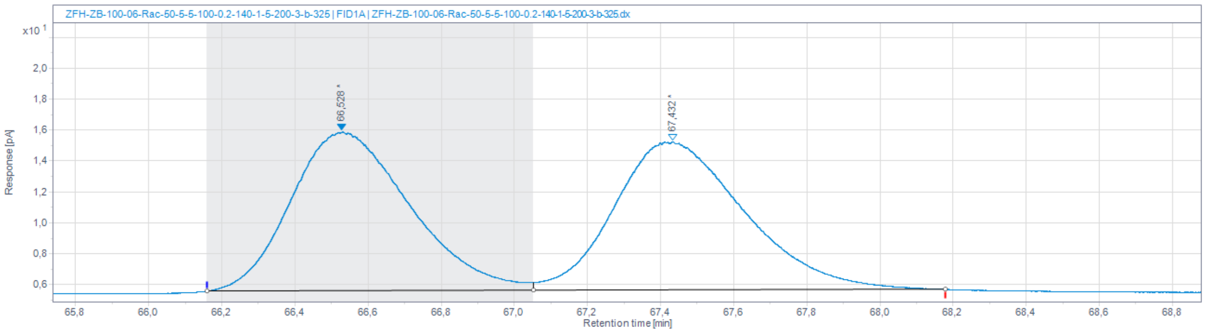

#	Name	Signal description	RT (min)	Area (pA·s)	Area%	Height (pA)	Height%	Amount	Concentration	Start time (min)	End time (min)
1		FID1A	66,528	232,704	49,428	10,265	51,73			66,160	67,052
2		FID1A	67,432	238,090	50,572	9,577	48,27			67,052	68,179

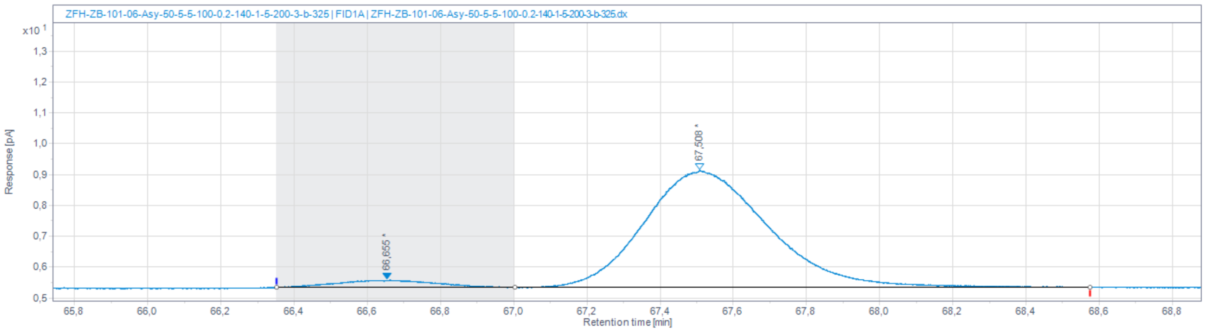

#	Name	Signal description	RT (min)	Area (pA·s)	Area%	Height (pA)	Height%	Amount	Concentration	Start time (min)	End time (min)
1		FID1A	66,655	4,161	4,505	0,222	5,55			66,353	67,004
2		FID1A	67,508	88,209	95,495	3,775	94,45			67,004	68,575

6. Mechanistic considerations and analysis of the stereochemical outcome.

To gain deep insight into the reaction process, mechanism, and stereochemical outcome, we performed a series of controlled experiments, hydrogenation with a recovered catalyst, and a mercury poisoning experiment.

6.1. Control experiments to investigate the dual role of the catalyst:

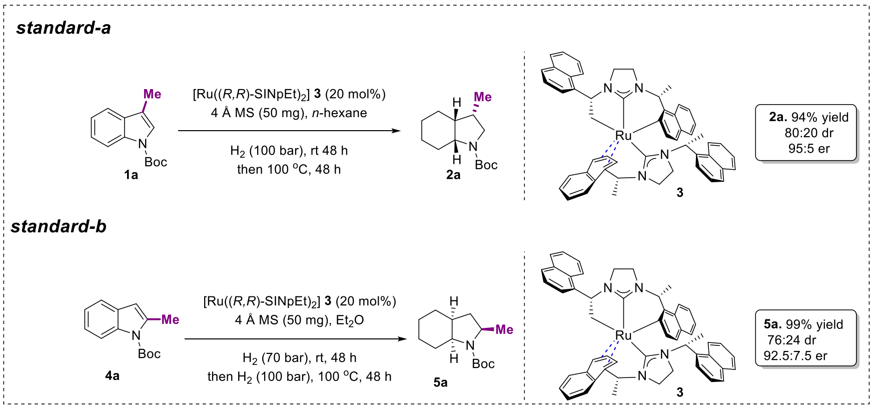

Scheme S1. Standard reactions

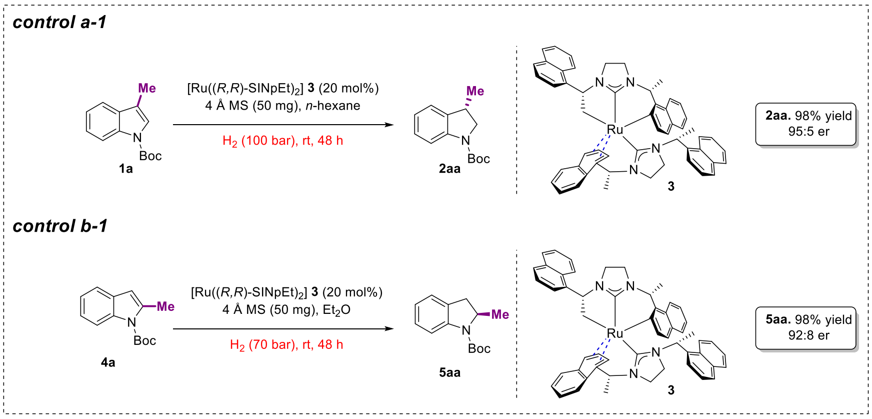

Scheme S2. Control experiment 1: partial hydrogenation of compounds **1a** and **4a.**

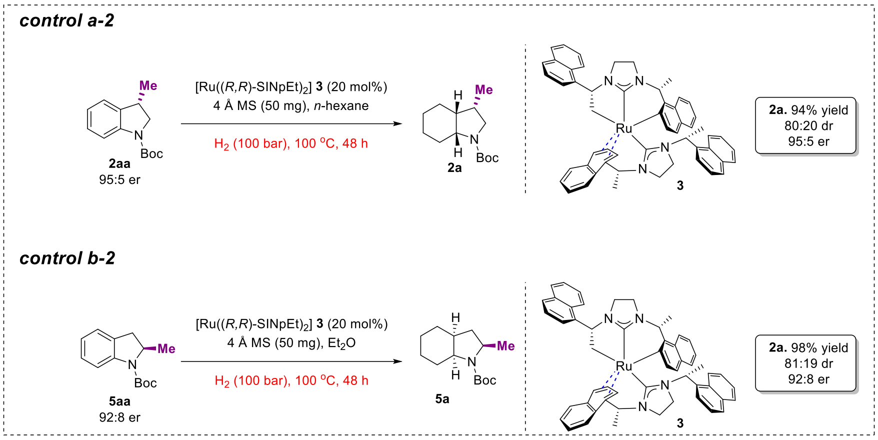

Scheme S3. Control experiment 2: hydrogenation of compounds **2aa** and **5aa**.

6.2. Procedure for partial hydrogenation of compound 1a.

To a 4 mL glass vial (screwcap with septum) equipped with a stir bar, **1a** (0.1 mmol, 1.00 equiv.), pulverized 4 Å molecular sieve (50 mg), and the vial carefully evacuated. Under an argon atmosphere, *n*-hexane (0.2 mL) and the preformed Ru((*R*,*R*)-SINpEt)$_2$ catalyst **3** (0.025 mmol/mL) as stock suspension (0.8 mL, 20 mol%) in *n*-hexane were added. The glass vial was placed in a 150 mL stainless steel autoclave under an argon atmosphere. The autoclave was pressurized and depressurized with hydrogen gas three times before the pressure was set to 100 bar. The reaction mixture was stirred at 25 °C for 48 h. Then the autoclave was carefully depressurized, and the crude product was purified by silica gel column chromatography with pentene:ethyl acetate (50:1) to give the pure product as a colorless oil (23 mg, 0.098 mmol, 98%, 95:5 er).

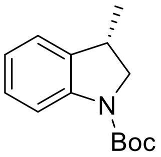

<u>*tert*-butyl (*S*)-3-methylindoline-1-carboxylate</u> (**2aa**). ^{1}H NMR (400 MHz, Chloroform-*d*) δ 7.84 – 7.46 (d, *J* = 153.3 Hz, 1H), 7.15 (dd, *J* = 18.9, 7.5 Hz, 2H), 7.02 – 6.91 (m, 1H), 4.15 (t, *J* = 10.0 Hz, 1H), 3.59 – 3.33 (m, 2H), 1.57 (s, 9H), 1.32 (d, *J* = 6.8 Hz, 3H). ^{13}C NMR (101 MHz, $CDCl_3$) δ 152.6, 141.7, 135.7, 127.5, 123.5, 122.2, 114.6, 80.4, 55.6, 34.0, 28.4, 20.2. $[\alpha]_D^{20}$: +29.4 (*c* = 1.0, CH_2Cl_2). ^{1}H NMR, ^{13}C NMR data, and optical rotation were consistent with those reported in literatures[3] (lit.[3] $[\alpha]_D^{25}$: +25.1, *c* = 1.0, $CHCl_3$). HPLC on Chiralpak IC-3 column; hexane:isopropanol = 99:1; flow rate = 0.5 mL/min; UV detection at 254 nm; t_R = 12.96 min (major), t_R = 14.03 min (minor).

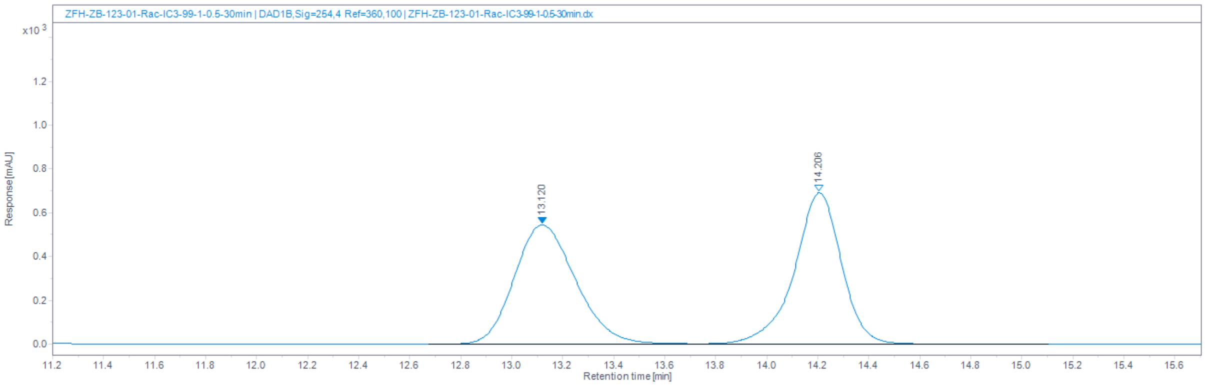

#	Name	Signal description	RT (min)	Area (mAU·s)	Area%	Height (mAU)	Height%	Amount	Concentration	Start time (min)	End time (min)
1		DAD1B,Sig=254,4 Ref=360,100	13.120	9096.102	50.557	543.704	44.03			12.675	13.737
2		DAD1B,Sig=254,4 Ref=360,100	14.206	8895.667	49.443	691.032	55.97			13.737	15.105

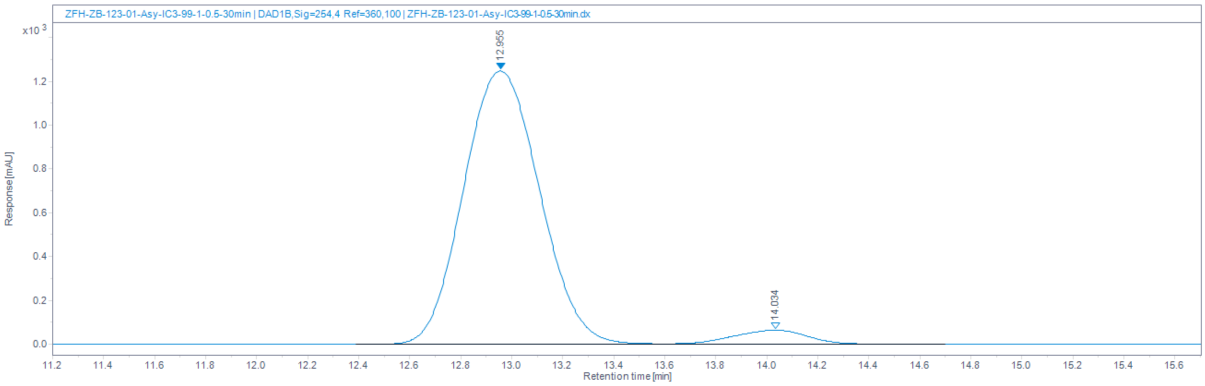

#	Name	Signal description	RT (min)	Area (mAU·s)	Area%	Height (mAU)	Height%	Amount	Concentration	Start time (min)	End time (min)
1		DAD1B,Sig=254,4 Ref=360,100	12.955	25850.056	95.283	1247.350	95.14			12.388	13.602
2		DAD1B,Sig=254,4 Ref=360,100	14.034	1279.663	4.717	63.724	4.86			13.602	14.698

6.3. Procedure for partial hydrogenation of compound 4a.

To an 8 mL glass vial (screwcap with septum) equipped with a stir bar, **4a** (0.1 mmol, 1.00 equiv.), pulverized 4 Å molecular sieve (50 mg), and the vial carefully evacuated. Under an argon atmosphere, Et_2O (2 mL) and the preformed Ru((*R*,*R*)-SINpEt)$_2$ catalyst **3** (0.025 mmol/mL) as stock suspension (0.8 mL, 20 mol%) in *n*-hexane was added. The glass vial was placed in a 150 mL stainless steel autoclave under an argon atmosphere. The autoclave was pressurized and depressurized with hydrogen gas three times before the pressure was set to 70 bar. The reaction mixture was stirred at 25 °C for 48 h. Then the autoclave was carefully depressurized, and the crude product was purified by silica gel column chromatography with pentene:ethyl acetate (50:1) to give the pure product as a colorless oil (23 mg, 0.098 mmol, 98%, 92:8 er).

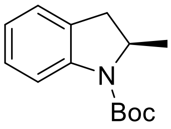

***tert*-butyl 2-methyl-1*H*-indole-1-carboxylate** (**5aa**). ^{1}H NMR (400 MHz, Chloroform-*d*) δ 7.75 (s, 1H), 7.21 – 7.05 (m, 2H), 6.94 (t, *J* = 7.4 Hz, 1H), 4.51 (s, 1H), 3.33 (d, *J* = 9.6 Hz, 1H), 2.71 – 2.50 (m, 1H), 1.57 (s, 9H),

1.29 (d, J = 6.3 Hz, 3H). ^{13}C NMR (101 MHz, $CDCl_3$) δ 152.3, 141.7, 130.0, 127.3, 124.9, 122.2, 115.2, 80.5, 55.2, 35.7, 28.4, 21.1. $[\alpha]_D^{20}$: –29.7 (c = 1.0, $CDCl_3$). ^{1}H NMR, ^{13}C NMR data, and optical rotation were consistent with those reported in literatures[3] (lit.[3] $[\alpha]_D^{25}$: –44.6, c = 0.52, $CHCl_3$). HPLC on Chiralpak IC-3 column; hexane:isopropanol = 99:1; flow rate = 0.5 mL/min; UV detection at 254 nm; t_R = 10.98 min (minor), t_R = 13.14 min (major).

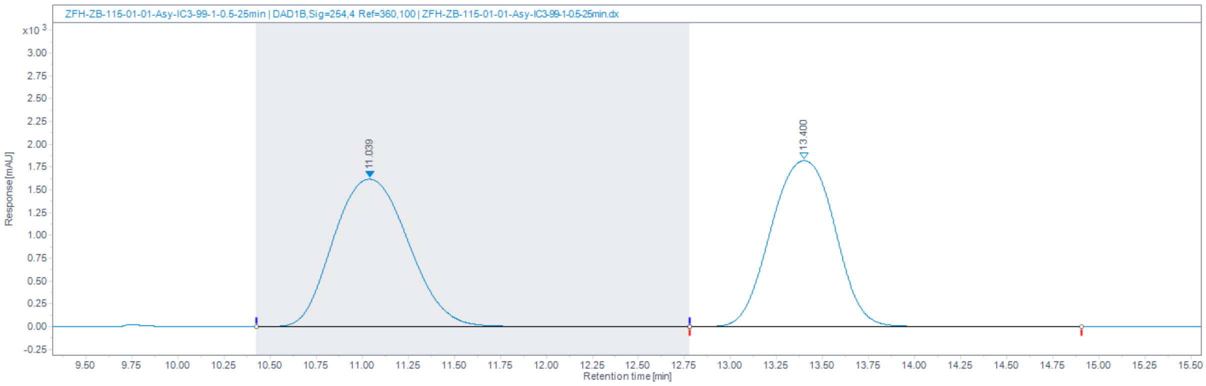

#	Name	Signal description	RT (min)	Area (mAU·s)	Area%	Height (mAU)	Height%	Amount	Concentration	Start time (min)	End time (min)
1		DAD1B,Sig=254,4 Ref=360,100	11.039	45681.278	50.931	1615.941	47.04			10.425	12.777
2		DAD1B,Sig=254,4 Ref=360,100	13.400	44011.889	49.069	1819.254	52.96			12.780	14.907

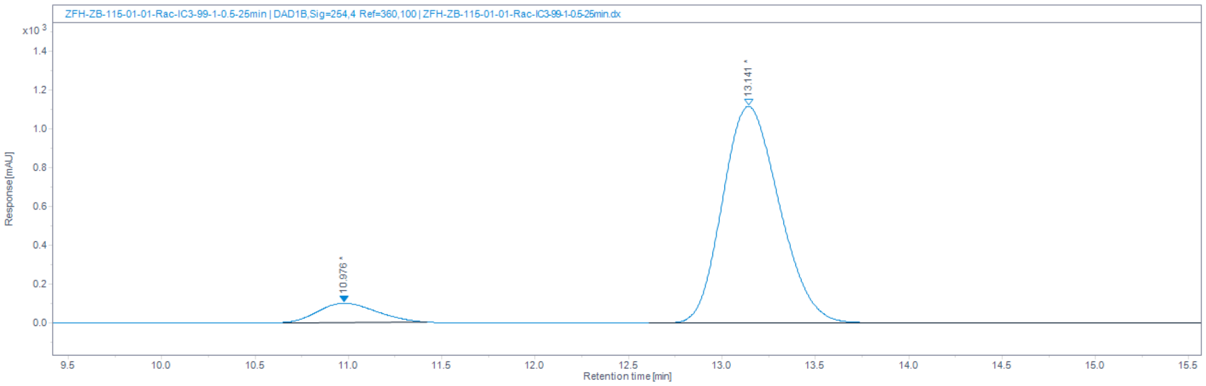

#	Name	Signal description	RT (min)	Area (mAU·s)	Area%	Height (mAU)	Height%	Amount	Concentration	Start time (min)	End time (min)
1		DAD1B,Sig=254,4 Ref=360,100	10.976	2122.096	8.245	97.988	8.06			10.651	11.420
2		DAD1B,Sig=254,4 Ref=360,100	13.141	23615.576	91.755	1118.185	91.94			12.609	16.045

6.4. Procedure for hydrogenation of compound 2aa

To a 4 mL glass vial (screwcap with septum) equipped with a stir bar, **2aa** (0.1 mmol, 1.00 equiv.), pulverized 4 Å molecular sieve (50 mg), and the vial carefully evacuated. Under an argon atmosphere, *n*-hexane (0.2 mL) and the preformed Ru((*R*,*R*)-SINpEt)$_2$ catalyst **3** (0.025 mmol/mL) as stock suspension (0.8 mL, 20 mol%) in *n*-hexane were added. The glass vial was placed in a 150 mL stainless steel autoclave under an argon atmosphere. The autoclave was pressurized and depressurized with hydrogen gas three times before the pressure was set to 100 bar. The reaction mixture was stirred at 100 °C for 48 h. Then the autoclave was cooled down to rt, it was carefully depressurized. Yields and diastereomeric and enantiomeric ratios were determined by GC-FID analysis.

6.5. Procedure for hydrogenation of compound 5aa

To an 8 mL glass vial (screwcap with septum) equipped with a stir bar, **5aa** (0.1 mmol, 1.00 equiv.), pulverized 4 Å molecular sieve (50 mg), and the vial carefully evacuated. Under an argon atmosphere, Et_2O (2 mL) and the preformed Ru((*R*,*R*)-SINpEt)$_2$ catalyst **3** (0.025 mmol/mL) as stock suspension (0.8 mL, 20 mol%) in *n*-hexane was added. The glass vial was placed in a 150 mL stainless steel autoclave under an argon atmosphere. The autoclave was pressurized and depressurized with hydrogen gas three times before the pressure was set to 100 bar. The reaction mixture was stirred at 100 °C for 48 h. Then the autoclave was cooled down to rt, it was carefully depressurized. Yields and diastereomeric and enantiomeric ratios were determined by GC-FID analysis.

6.6. Hydrogenation of 5aa with recovered catalyst

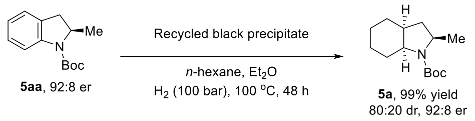

To an 8 mL glass vial (screwcap with septum) equipped with a stir bar, **5aa** (0.1 mmol, 1.00 equiv.), and the vial carefully evacuated. Under an argon atmosphere, Et_2O (2 mL), *n*-hexane (0.8 mL), and the isolated black precipitate recovered from the standard reaction (20 mol%) were added. The glass vial was placed in a 150 mL stainless steel autoclave under an argon atmosphere. The autoclave was pressurized and depressurized with hydrogen gas three times before the pressure was set to 100 bar. The reaction mixture was stirred at 100 °C for 48 h. Then the autoclave was cooled down to rt, it was carefully depressurized. Yields and diastereomeric and enantiomeric ratios were determined by GC-FID analysis.

6.7. Mercury poisoning experiments

Furthermore, to prove that the nature of the active catalytic species in the hydrogenation of six-membered ring is heterogeneous, the hydrogenation of **5aa** was performed in the presence of mercury.[4]

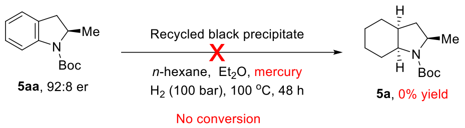

To an 8 mL glass vial (screwcap with septum) equipped with a stir bar, **5aa** (0.1 mmol, 1.00 equiv.), and 20 drops mercury, the vial was carefully evacuated. Under an argon atmosphere, Et_2O (2 mL), *n*-hexane (0.8 mL), and the isolated black precipitate recovered from the standard reaction (20 mol%) were added. The glass vial was placed in a 150 mL stainless steel autoclave under an argon atmosphere. The autoclave was pressurized and depressurized with hydrogen gas three times before the pressure was set to 100 bar. The reaction mixture was stirred at 100 °C for 48 h. Then the autoclave was cooled down to rt, it was carefully depressurized. Yields and diastereomeric and enantiomeric ratios were determined by GC-FID analysis.

6.8. Analysis

For control 1: We first allowed the standard substrate to react at rt and indicated H_2 pressure for 48 hours, and then stopped the reaction without heating up, finding that the partially hydrogenated product was able to be obtained in high yields and good e.r. (Scheme S2). Then enantiomeric ratios were identical to the results for the product under standard reaction conditions (Scheme S1). Additionally, complete hydrogenation products were not observed. This indicates that the first partial hydrogenation of the pyrrole ring is completed before increasing reaction temperature and high reaction temperature were needed to form Ru-nanoparticles.

For control 2: Reactions, using the partially hydrogenated products **2aa** and **5aa** as substrates, Ru((*R*,*R*)-SINpEt)$_2$ **3** as catalyst, reacted at 100 ºC and 100 atm for 48 h. We can see that the results were comparable with those of the one-pot reactions (Scheme S1, S3). This indicates that no enantioinformation is lost during the hydrogenation of dihydro-intermediates to fully saturated products. Furthermore, the obtained major diastereomer of stepwise hydrogenation was identical to the major diastereomer obtained in the one-pot procedure (Scheme S1, S3). The diastereoselectivity in the six-membered-ring was controlled by the stereo centers installed in the five-membered-ring hydrogenation.

Hydrogenation with recovered catalyst and mercury poisoning experiments: From the comparison of these two experiments, a complete poison for the heterogeneous catalysts was observed when mercury was presented. These results indicated that the active species in the second hydrogenation is heterogeneous.

7. Analysis of heterogenous catalyst after catalytical cycle using TEM, HAADF-STEM, and EDX analysis

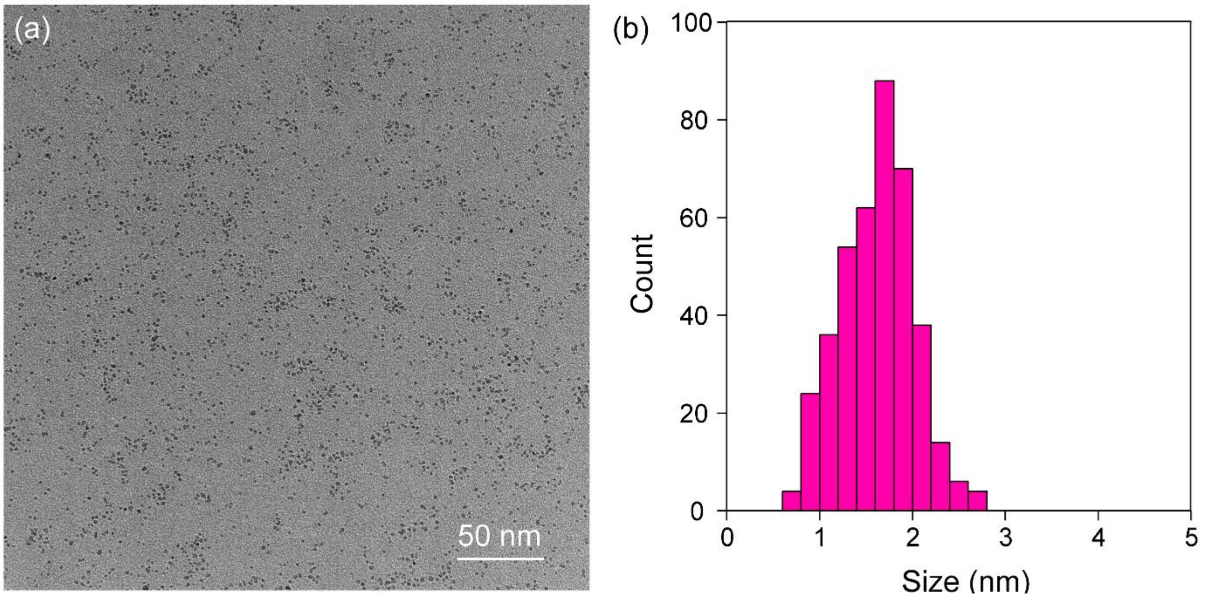

Figure S1: (a) TEM image of the isolated nanoparticles after catalysis. (b) Histogram showing the particle size distribution of nanoparticles with an average size of 1.7 ± 0.9 nm.

Procedure for the preparation of the TEM sample: After the standard reaction, the reaction mixture showed the formation of a black precipitate in the reaction mixture. The reaction mixture was centrifuged and the colourless

supernatant from the reaction mixture was removed to isolate the residual part. The black residue was washed with *i*-PrOH. The black residue was again redispersed in *i*-PrOH and the TEM sample was prepared by direct drop casting, 5 µL of dispersed black particles in *i*-PrOH onto a copper grid. The sample was measured on a carbon-coated copper grid. For the determination of nanopartcle size distributions, the size of 400 Nanoparticles were measured using the ImageJ software.

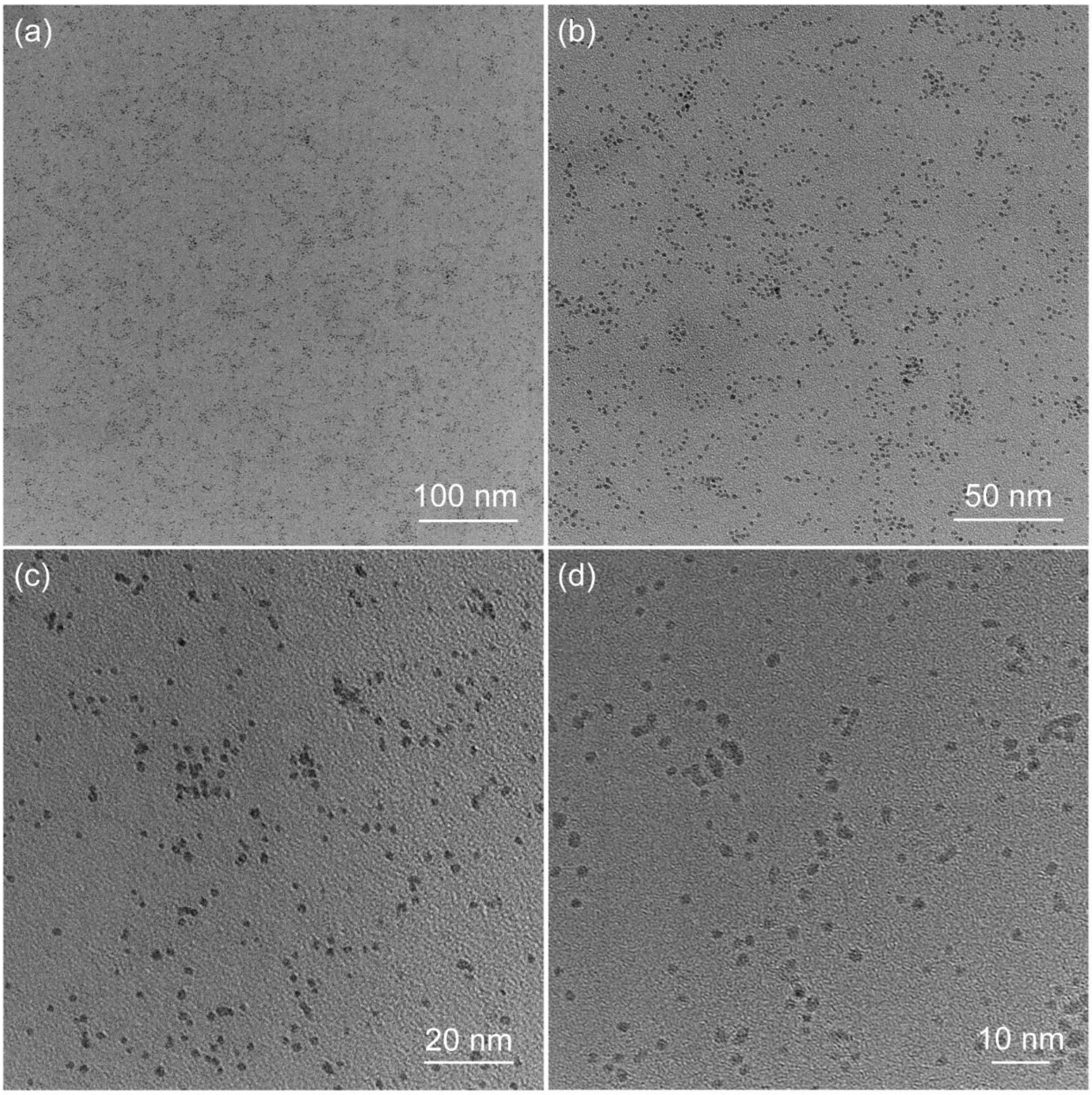

Figure S2: (a)-(d) TEM images of the isolated catalysts after completion of the catalytic hydrogenation.

TEM images of the isolated catalysts after completion of the catalytic hydrogenation revealed the formation of nanoparticles during the reaction. The size distribution histograms of nanoparticles obtained from TEM micrographs revealed that the average size of the nanoparticles is 1.7 ± 0.9 nm.

High-angle annular dark field scanning transmission electron microscopy (HAADF-STEM), and STEM-energy-dispersive X-ray spectrometry (EDX) have effectively identified and characterized nanoparticles. The image contrast in HAADF-STEM has strongly correlated to the atomic mass: heavier elements contribute to brighter contrast. The element of the nanoparticles has been identified as Ru which also appears as a very bright spot in the HAADF-STEM imaging.

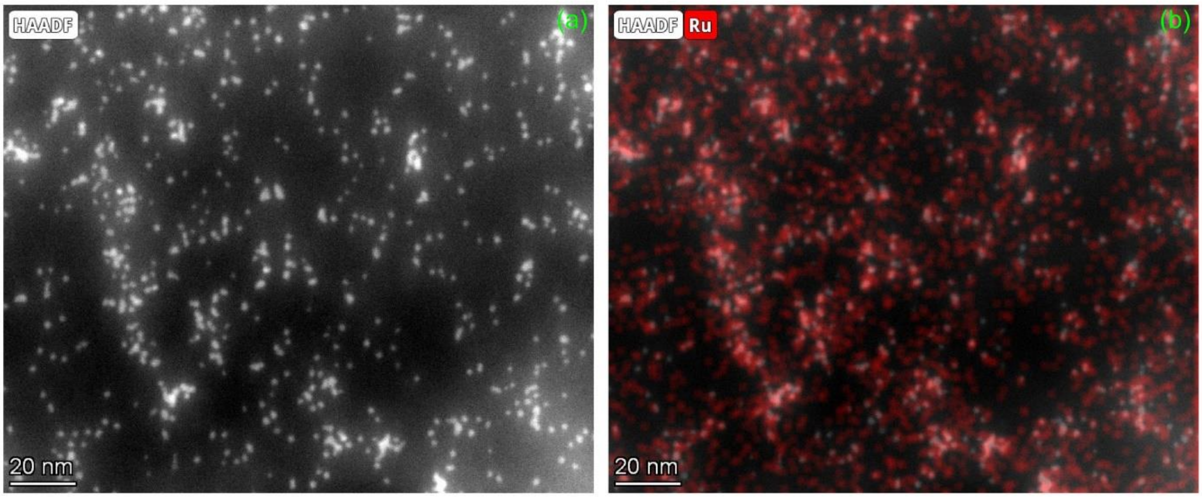

Figure S3: (a) High-angle annular dark field - scanning transmission electron microscopy (HAADF-STEM) image and (b) energy dispersive X-ray (EDX) mapping of the catalyst nanoparticles after the completion of the catalytic hydrogenation reaction.

8. Low catalyst loading experiments

Table **S3**. Investigations on catalyst loading of Ru-NHC catalyzed asymmetric, complete hydrogenation of **4a**.[a]

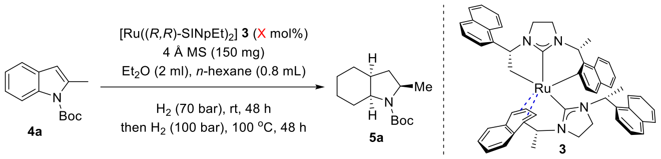

entry	Catalyst loading (X)	Conversion of **4a** (%)	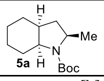			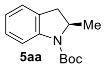	
			yield (%)[b]	dr[b]	er[c]	yield (%)[b]	er[d]
1	5 mol%	100	100	76:24	92.5:7.5	0	-----
2	2.5 mol%	100	98	76:24	92.5:7.5	0	-----
3	1 mol%	100	63	76:24	92.5:7.5	37	92:8
4	5 mol%	100	57	76:24	92.5:7.5	43	92:8

[a] General conditions: **4a** (0.3 mmol), additive (150 mg), and **3** indicated amount in Et_2O (2 ml), *n*-hexane (0.8 ml), and the hydrogenation was performed at 25 °C under 70 bar H_2 for 48 h, then at 100 °C under 100 bar H_2 for 48 h. [b] Determined by GC-FID. [c] Determined by GC-FID on a chiral stationary phase. [d] Determined by HPLC on a chiral stationary phase.

To an 8 mL glass vial (screwcap with septum) equipped with a stir bar, **4a** (0.3 mmol, 1.00 equiv.), pulverized 4 Å molecular sieve (150 mg), and the vial carefully evacuated. Under an argon atmosphere Et_2O (2 mL), n-hexane (0.8 ml), and the solid catalyst **3** (indicated amount) was added. The glass vial was placed in a 150 mL stainless steel autoclave under an argon atmosphere. The autoclave was pressurized and depressurized with hydrogen gas three times before the pressure was set to 70 bar. The reaction mixture was stirred at 25 °C for 48 h. After this period, the hydrogen pressure was increased to 100 bar and the temperature increased to 100 °C and the reaction mixture continued to stir for 48 h. Then the autoclave was cooled down to rt, it was carefully depressurized. Yield

and d.r. were determined by GC-FID analysis of the crude mixture. e.r. was determined by GC-FID on a chiral stationary phase or HPLC on a chiral stationary phase.

Analysis: When we used 2.5 mol% of catalyst, the results were good and consistent with the standard condition (entry 2, table S3). However, when we used less than 1 mol% of catalyst (entry 3 and 4, table S3), we found that **4a** could be completely consumed, but only a moderate yield could be achieved. The reason for this was that the second hydrogenation was not completed, leaving some partially hydrogenated product **5aa**. Furthermore, when we reduced the catalyst amount to 0.5 mol%, we found that the enantiomeric ratio of **5a** and **5aa** were comparable and the e.r. value were same as the standard condition (entry 4, table S3). This demonstrates that catalyst **3** has a high catalytic activity for substrate **4a** and could completely catalyze the hydrogenation of **4a** to **5aa** before transforming **3** to Ru nanoparticles. Therefore, the main limitation for the catalyst loading in the reaction was the heterogeneous hydrogenation step.

9. Preliminary comparison between the current protocol and the previous method with two catalysts.

To investigate the advantages of the current single-catalyst protocol and our previous two-catalyst method,[2g] we conducted some comparative experiments.

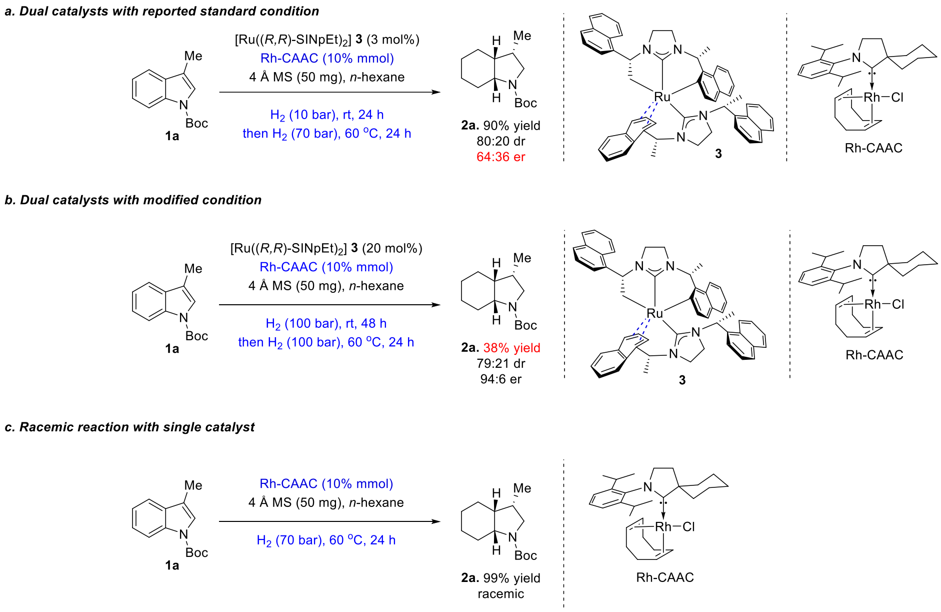

Scheme S4. Comparative experiment between current protocol and reported methods.

9.1. Analysis

First, we attempted to hydrogenate compound **1a** using a dual-catalyst method.[2g] The results showed that the reaction could proceed, but the e.r. of the product was less satisfactory (Scheme S4, equation a). This could be because **1a** was not completely converted to the dihydro-product before the heterogeneous catalyst was activated, which led to the occurrence of a racemic background reaction. In order to get full conversion in the first step, we adjusted the reaction conditions by increasing the reaction time, hydrogen pressure, and catalyst loading. The results showed that the e.r. of the product was slightly lower than the currently reported reaction, but the yield

was significantly reduced (Scheme S4, equation b). By contrast, in the racemic reaction with Rh-CAAC as a single catalyst, **1a** can be completely hydrogenated within 24 h (Scheme S4, equation c). This demonstrated that Rh-CAAC was inhibited in the high-pressure reaction condition. Therefore, from the preliminary comparative experiments, it appears that the single catalyst has some advantages over the two-catalyst system in certain aspects.

10. Gram scale synthesis of 5a

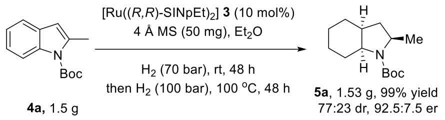

In an argon-filled glovebox, [Ru(COD)(2-methylally)$_2$] (319 mg, 1 mmol), KO*t*-Bu (225 mg, 2 mmol), (*R*, *R*)-SINpEt·HBF_4 (933 mg, 2 mmol) were added in a 100 ml Schlenk tube. Then under an argon atmosphere, *n*-hexane (40 ml) was added to the Schlenk tube. After stirring at 70 °C for 16 h, the reaction was cool down to rt. The solvent was removed under vacuum and a solid catalyst **3** was obtained. The solid catalyst **3** is not stable to oxygen and should be stored in an argon atmosphere. In an argon-filled glovebox, to a 100 mL round-bottom flask equipped with a stir bar, the substrate (1.5g, 6.5 mmol, 1.00 equiv.), pulverized 4 Å molecular sieve (3.2 g), and performed solid Ru((*R*,*R*)-SINpEt)$_2$ catalyst **3** (630mg, 10 mol%) were added. Under an argon atmosphere, Et_2O (26 mL), and *n*-hexane (6 mL) were added to the round-bottom flask. Then, the round-bottom flask was placed in a 150 mL stainless steel autoclave under an argon atmosphere. The autoclave was pressurized and depressurized with hydrogen gas three times before the pressure was set to 70 bar. The reaction mixture was stirred at 25 °C for 48 h. After this period, the hydrogen pressure was increased to 100 bar and the temperature increased to 100 °C and the reaction mixture continued to stir for 48 h. Then the autoclave was cooled down to rt, it was carefully depressurized, and the crude product was purified by silica gel column chromatography (pentene:ethyl acetate = 50:1) to give the pure product 1.53 g (6.4 mmol, 98% yield). d.r. was determined by GC-FID analysis of the crude mixture. E.r. was determined by GC-FID on a chiral stationary phase.

11. Elaboration and synthetic application of the products

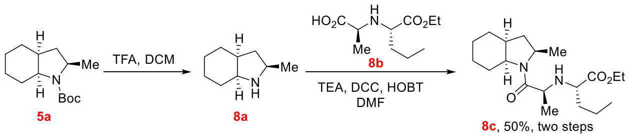

To a solution of **5a** (120 mg, 0.5 mmol) in DCM (2 mL) was added TFA (2 mL). The mixture was stirred at room temperature for 30 min and then the solvent was removed under reduced pressure. The crude product was used for the next step without purification. To a solution of **8b** (109 mg, 0.5 mmol) in DMF (2 mL) was added DCC (113 mg, 0.55 mmol), and HOBT (85 mg, 80% with water, 0.5 mmol). The mixture was stirred at 0 °C for 30 min. Then the solution was added TEA (0.15 mL, 1.1 mmol), and **5a** from the last step at 0 °C. The mixture was stirred at rt for 6 h. The reaction was quenched with H_2O (10 mL) and extracted with EtOAc (3 × 10 mL). The combined organic layer was washed with brine and dried over anhydrous Na_2SO_4. The solvent was removed under reduced

to afford the crude product which was purified by silica gel column chromatography (1:1 = pentene:ethyl acetate) to give **8c** (85 mg, 0.25 mmol, 50%, 84:16 dr) as a solid.

Ethyl (*S*)-2-(((*S*)-1-((2*R*,3a*S*,7a*S*)-2-methyloctahydro-1*H*-indol-1-yl)-1-oxopropan-2-yl)amino)pentanoate (**8c**). ^{1}H NMR (400 MHz, Chloroform-*d*) δ 4.18 (q, *J* = 7.1 Hz, 2H), 4.10 – 3.97 (m, 1H), 3.68 (dt, *J* = 12.1, 6.2 Hz, 1H), 3.50 (q, *J* = 6.6 Hz, 1H), 3.07 (t, *J* = 6.8 Hz, 1H), 2.29 – 2.09 (m, 2H), 2.02 – 1.90 (m, 1H), 1.79 – 1.46 (m, 8H), 1.38 – 1.10 (m, 14H), 0.88 (t, *J* = 7.3 Hz, 3H). ^{13}C NMR (101 MHz, $CDCl_3$) δ 174.8, 173.6, 60.5, 60.0, 58.4, 53.1, 53.07, 37.0, 35.6, 34.5, 30.1, 25.9, 24.2, 24.1, 21.6, 20.7, 19.0, 14.3, 13.8. HRMS (ESI) Calcd for $C_{19}H_{34}N_2NaO_3$ $[M+Na]^+$: 361.2462, found: 361.2458.

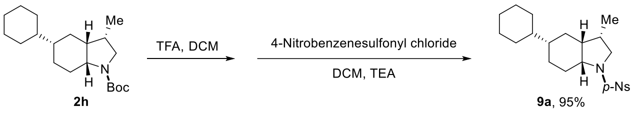

To a solution of **2h** (30 mg, 0.093 mmol) in DCM (1 mL) was added trifluoroacetic acid (1 mL). The mixture was stirred at room temperature for 1 h and then the solvent was removed under reduced pressure. The crude product was used for the next step directly. To a solution of crude product obtained from the last step in DCM (2 mL) was added TEA (29 mg, 0.28 mmol), 4-nitrobenzene sulfonyl chloride (25 mg, 0.11 mmol). The mixture was stirred at room temperature for 2 h and then quenched with water (10 mL) and extracted with DCM (3 × 10 mL). The combined organic layer was washed with brine, dried over anhydrous Na_2SO_4, and filtered. The solvent was removed under reduced pressure. The crude product was purified by silica gel column chromatography (100:1 = pentene:ethyl acetate) to give **9a** (36 mg, 0.89 mmol, 95%) as a white solid. The compound was used for single crystal cultivation in DCM and Et_2O.

(3*S*,3a*R*,5*S*,7a*R*)-5-cyclohexyl-3-methyl-1-((4-nitrophenyl)sulfonyl)octahydro-1*H*-indole (9a) ^{1}H NMR (400 MHz, Chloroform-*d*) δ 8.38 (d, *J* = 8.8 Hz, 2H), 8.00 (d, *J* = 8.8 Hz, 2H), 3.49 (dd, *J* = 10.8, 8.5 Hz, 1H), 3.34 (q, *J* = 3.6 Hz, 1H), 3.16 (t, *J* = 11.1 Hz, 1H), 2.62 (dq, *J* = 14.4, 3.2 Hz, 1H), 1.94 – 1.83 (m, 1H), 1.76 – 1.63 (m, 5H), 1.57 (s, 2H), 1.55 – 1.43 (m, 3H), 1.24 – 1.07 (m, 5H), 1.04 – 0.93 (m, 3H), 0.86 (d, *J* = 6.8 Hz, 3H). ^{13}C NMR (101 MHz, $CDCl_3$) δ 149.9, 143.2, 128.6, 124.2, 61.8, 54.4, 43.2, 43.2, 41.4, 35.1, 30.2, 30.0, 28.6, 26.7, 25.7, 23.5, 11.8. HRMS (ESI) Calcd for $C_{21}H_{30}N_2NaO_4S$ $[M+Na]^+$: 429.1818, found: 429.1818.

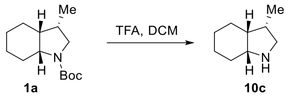

To a solution of **1a** (100 mg, 0.42 mmol) in DCM (3 mL) was added trifluoroacetic acid (3 mL). The mixture was stirred at room temperature for 1 h and then the solvent was removed under reduced pressure. The crude was dissolved in DCM and saturated sodium bicarbonate solution. The solution was extracted with DCM (3 × 10 mL). The combined organic layer was dried over anhydrous Na_2SO_4 and filtered. The solvent was removed under reduced pressure under low temperature as the product **10c** is a volatile liquid. The crude product **10c** was used for the next step without purification.

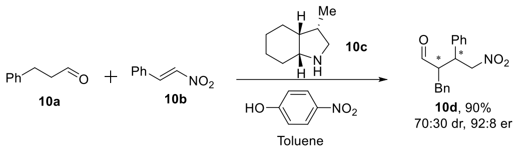

To a solution of **10a** (27 mg, 0.2 mmol) in toluene (0.2 mL) was added **10b** (30 mg, 0.2 mmol), 4-nitrophenol (1.4 mg, 0.01 mmol), and the freshly prepared catalyst **10c** (5.5 mg, 0.04 mmol). The mixture was stirred at room temperature for 24 h and then quenched with water (10 mL) and extracted with ethyl acetate (3 × 10 mL). The combined organic layer was washed with brine, dried over anhydrous Na_2SO_4, and filtered. The solvent was removed under reduced pressure. The crude product was purified by silica gel column chromatography (100:1 = pentene:ethyl acetate) to give **10d** (51 mg, 0.18 mmol, 90%, 70:30 dr, 92:8 er) as a colorless oil.

2-benzyl-4-nitro-3-phenylbutanal (**10d**). ^{1}H NMR (400 MHz, Chloroform-*d*) δ 9.61 (d, *J* = 2.3 Hz, 1H), 7.33 – 7.00 (m, 14H), 6.94 (d, *J* = 7.3 Hz, 2H), 4.84 – 4.66 (m, 1H), 4.66 – 4.56 (m, 2H), 3.81 – 3.68 (m, 2H), 3.08 – 2.87 (m, 2H), 2.83 – 2.74 (m, 1H), 2.74 – 2.62 (m, 2H). ^{13}C NMR (101 MHz, $CDCl_3$) δ 203.1, 203.0, 137.2, 137.1, 136.6, 135.8, 129.2, 129.1, 128.9, 128.8, 128.74, 128.7, 128.4, 128.3, 128.0, 127.0, 126.9, 78.0, 77.6, 55.2, 54.3, 44.4, 43.4, 34.2, 33.6. ^{1}H NMR, and ^{13}C NMR data were consistent with those reported in the literatures.[5] HPLC on Chiralpak IC-3 column; hexane:isopropanol = 95:5; flow rate = 0.5 mL/min; UV detection at 254 nm; : t_1 = 47.71 min (minor diastereomer, major enantiomer), t_2 = 64.41 min (major diastereomer, minor enantiomer), t_3 = 67.67 min (minor diastereomer, minor enantiomer), t_4 = 85.65 min (major diastereomer, major enantiomer).

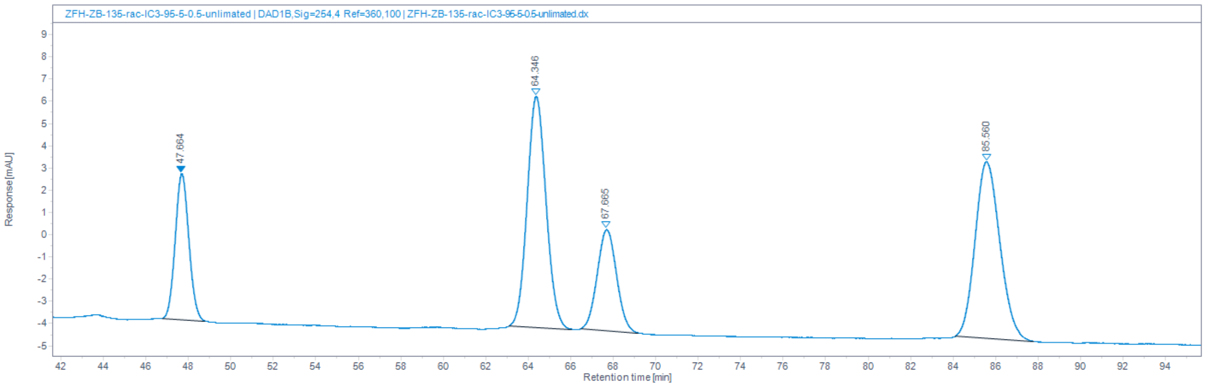

#	Name	Signal description	RT (min)	Area (mAU·s)	Area%	Height (mAU)	Height%	Amount	Concentration	Start time (min)	End time (min)
1		DAD1B,Sig=254,4 Ref=360,100	47.664	293.027	15.694	6.573	22.30			46.774	48.774
2		DAD1B,Sig=254,4 Ref=360,100	64.346	641.710	34.368	10.399	35.28			63.101	66.053
3		DAD1B,Sig=254,4 Ref=360,100	67.665	286.896	15.365	4.551	15.44			66.474	69.187
4		DAD1B,Sig=254,4 Ref=360,100	85.560	645.551	34.574	7.950	26.97			84.101	87.801

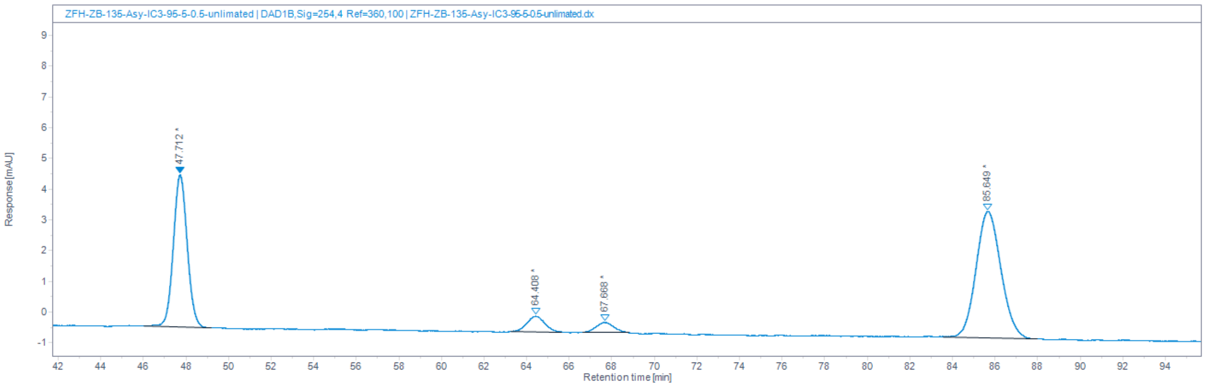

#	Name	Signal description	RT (min)	Area (mAU·s)	Area%	Height (mAU)	Height%	Amount	Concentration	Start time (min)	End time (min)
1		DAD1B,Sig=254,4 Ref=360,100	47.712	224.291	37.066	4.957	49.98			46.026	49.194
2		DAD1B,Sig=254,4 Ref=360,100	64.408	29.778	4.921	0.510	5.15			63.256	65.621
3		DAD1B,Sig=254,4 Ref=360,100	67.668	19.217	3.176	0.332	3.35			66.643	68.788
4		DAD1B,Sig=254,4 Ref=360,100	85.649	331.828	54.837	4.118	41.52			83.584	87.995

12. Sensitivity screen

Recently, our group has established a reaction-condition-based assignment to evaluate the sensitivity of a chemical reaction.[6] The sensitivity screen conducted in this study is based on a modification of the literature procedure.

Standard conditions:

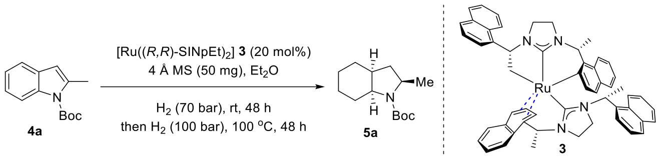

To an 8 mL glass vial (screwcap with septum) equipped with a stir bar, the substrate (0.1 mmol, 1.00 equiv.), pulverized 4 Å molecular sieve (50 mg), and the vial carefully evacuated. Under an argon atmosphere, Et_2O (2 mL) and the preformed Ru((*R*,*R*)-SINpEt)$_2$ catalyst **3** (0.025 mmol/mL) as stock suspension (0.8 mL, 20 mol%) in *n*-hexane was added. The glass vial was placed in a 150 mL stainless steel autoclave under an argon atmosphere. The autoclave was pressurized and depressurized with hydrogen gas three times before the pressure was set to 70 bar. The reaction mixture was stirred at 25 °C for 48 h. After this period, the hydrogen pressure was increased to 100 bar and the temperature increased to 100 °C and the reaction mixture continued to stir for 48 h. Then the autoclave was cooled down to rt, it was carefully depressurized. Dodecane (20 µl) was added. Yield and d.r. were determined by GC-FID analysis of the crude mixture. E.r. was determined by GC-FID on a chiral stationary phase.

Table S4: Reaction-condition-based assignment for the evaluation of the developed hydrogenation.

Entry	Modification	Deviation from standard condition	Yield	Deviation	ee	Deviation of ee
**1**	High *c*	– 1.0 mL Et_2O	98%	– 1%	84%	– 1%
**2**	Low *c*	+ 1.0 mL Et_2O	96%	– 3%	85%	0%
**3**	H_2O	+ 5 µl H_2O	88%	– 11%	84%	– 1%

**4**	Low O_2	degassed solvent	97%	– 2%	85%	0%
**5**	High O_2	+ 5 mL air, autoclave not purged	0%	– 99%	0%	– 100%
**6**	Low *T*	15 °C and 80 °C	99%	0%	84%	– 1%
**7**	High *T*	40 °C and 120 °C	98%	– 1%	83%	– 2%
**8**	Low *p*	50 bar and 80 bar H_2	99%	0%	85%	0%
**9**	High *p*	90 bar and 120 bar H_2	99%	0%	83%	– 2%
**10**	Big Scale	Standard scale x 65	99%	0%	85%	0%

Analysis

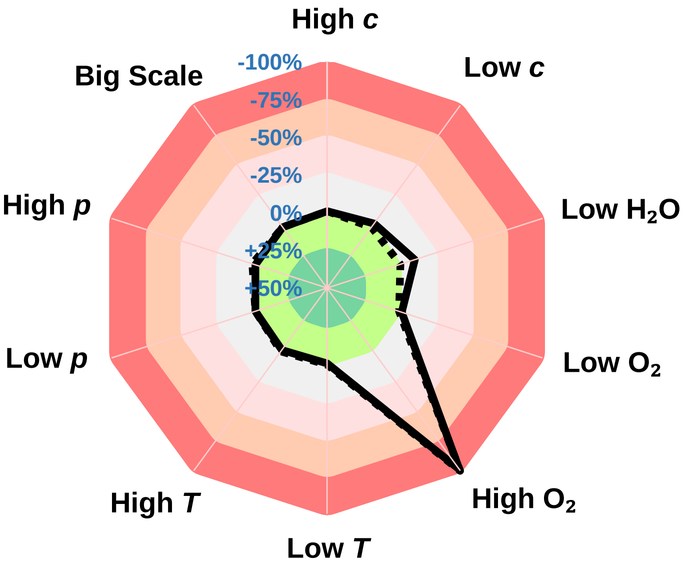

Figure S4: Radar diagram for the reaction-condition-based sensitivity assessment. *c* = concentration. *T* = temperature. *p* = pressure. The solid line indicates the yield, and the dashed line indicates the enantiomeric excess.

Small deviations in the reaction concentration did not have a significant impact on the reaction outcome. While the addition of H_2O led to a slight decrease in the reaction yield, reducing oxygen level had no impact. Furthermore, small deviations in temperature and pressure almost have significant impact on the reaction results. The enantiomeric excess of the obtained product was not sensitive to deviations except to the increased oxygen level, which decomposes the chiral ruthenium catalyst. Scaling up the reaction did not impact yield or enantiomeric excess, which underlines the usefulness of our developed protocol.

13. X-Ray analysis 9a

X-Ray diffraction: Data sets for compound **9a** were collected with a Bruker D8 Venture Photon III Diffractometer. Programs used: data collection: *APEX4* Version 2021.4-0[7] (Bruker AXS Inc., **2021**); cell refinement: *SAINT* Version 8.40B (Bruker AXS Inc., **2021**); data reduction: *SAINT* Version 8.40B (Bruker AXS Inc., **2021**); absorption correction, *SADABS* Version 2016/2 (Bruker AXS Inc., **2021**); structure solution *SHELXT*-Version 2018-3[8] (Sheldrick, G. M. *Acta Cryst.*, **2015**, *A71*, 3-8); structure refinement *SHELXL*- Version 2018-3 [9] (Sheldrick, G. M. *Acta Cryst.*, **2015**, *C71* (1), 3-8) and graphics, *XP* [10] (Version 5.1, Bruker AXS Inc.,

Madison, Wisconsin, USA, **1998**). *R*-values are given for observed reflections, and wR^2 values are given for all reflections.

X-ray crystal structure analysis of 9a (glo10446): A colorless, plate-like specimen of $C_{21}H_{30}N_2O_4S$, approximate dimensions 0.046 mm x 0.233 mm x 0.233 mm, was used for the X-ray crystallographic analysis. The X-ray intensity data were measured on a single crystal diffractometer Bruker D8 Venture Photon III system equipped with a micro focus tube Cu ImS (CuKα, λ = 1.54178 Å) and a MX mirror monochromator. A total of 2326 frames were collected. The total exposure time was 21.44 hours. The frames were integrated with the Bruker SAINT software package using a wide-frame algorithm. The integration of the data using a monoclinic unit cell yielded a total of 18486 reflections to a maximum θ angle of 66.59° (0.84 Å resolution), of which 3526 were independent (average redundancy 5.243, completeness = 97.9%, R_{int} = 3.84%, R_{sig} = 2.89%) and 3471 (98.44%) were greater than $2\sigma(F^2)$. The final cell constants of a = 6.1573(2) Å, b = 10.8653(3) Å, c = 15.5385(4) Å, β = 94.7290(10)°, volume = 1036.00(5) $Å^3$, are based upon the refinement of the XYZ-centroids of 9931 reflections above 20 σ(I) with 9.944° < 2θ < 136.5°. Data were corrected for absorption effects using the Multi-Scan method (SADABS). The ratio of minimum to maximum apparent transmission was 0.795. The calculated minimum and maximum transmission coefficients (based on crystal size) are 0.7030 and 0.9290. The structure was solved and refined using the Bruker SHELXTL Software Package, using the space group $P2_1$, with Z = 2 for the formula unit, $C_{21}H_{30}N_2O_4S$. The final anisotropic full-matrix least-squares refinement on F^2 with 254 variables converged at R1 = 2.42%, for the observed data and wR2 = 6.29% for all data. The goodness-of-fit was 1.049. The largest peak in the final difference electron density synthesis was 0.259 $e^-/Å^3$ and the largest hole was -0.259 $e^-/Å^3$ with an RMS deviation of 0.034 $e^-/Å^3$. On the basis of the final model, the calculated density was 1.303 g/cm^3 and F(000), 436 e^-. Flack parameter was refined to 0.088(6). CCDC Nr.: 2256901.

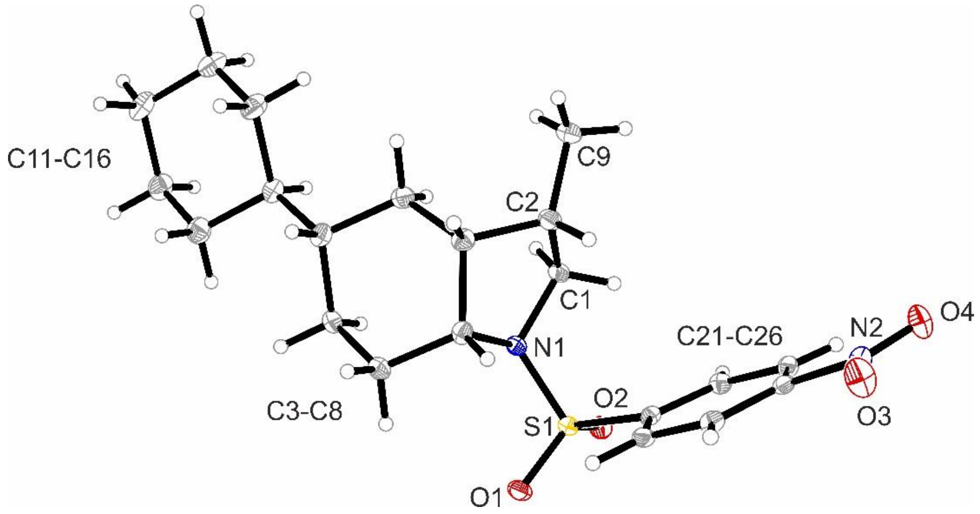

Figure S5. Crystal structure of compound 9a. Thermal ellipsoids are shown at 50% probability.

14. Spectral data

^{1}H NMR (400 MHz, Chloroform-*d*) of compound **1a**

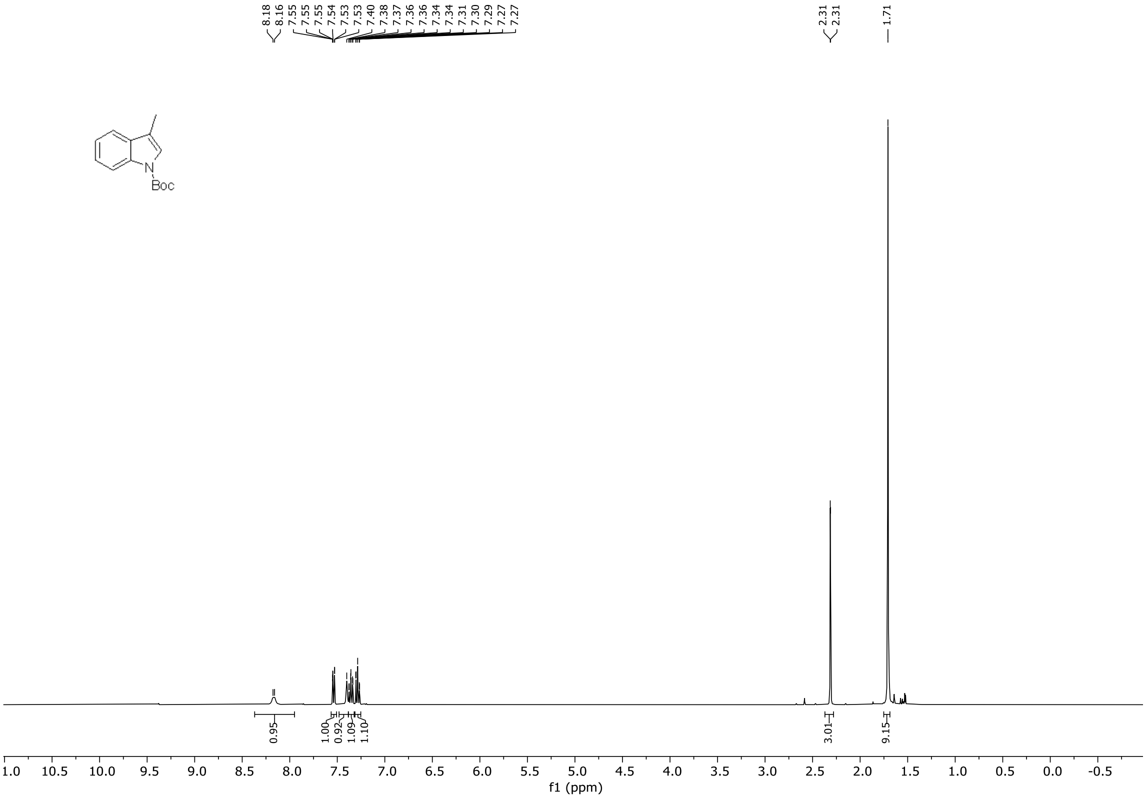

^{13}C NMR (101 MHz, Chloroform-*d*) of compound **1a**

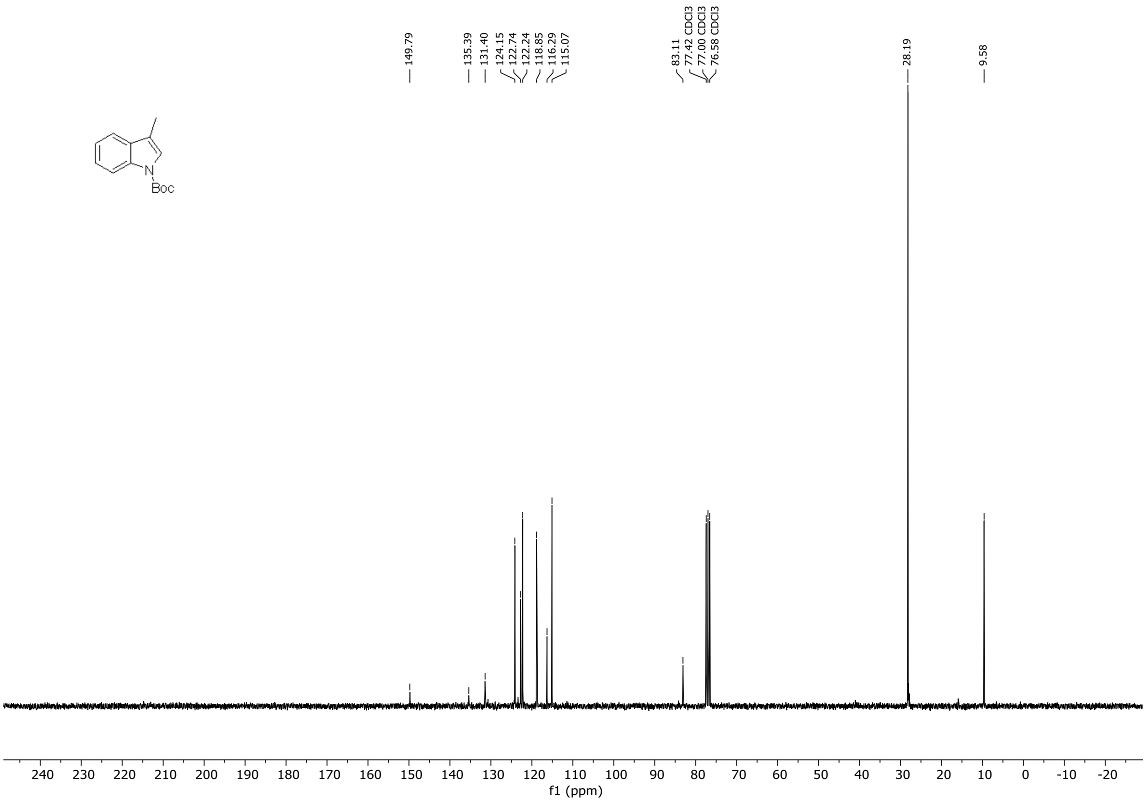

^{1}H NMR (400 MHz, Chloroform-*d*) of compound **1b**

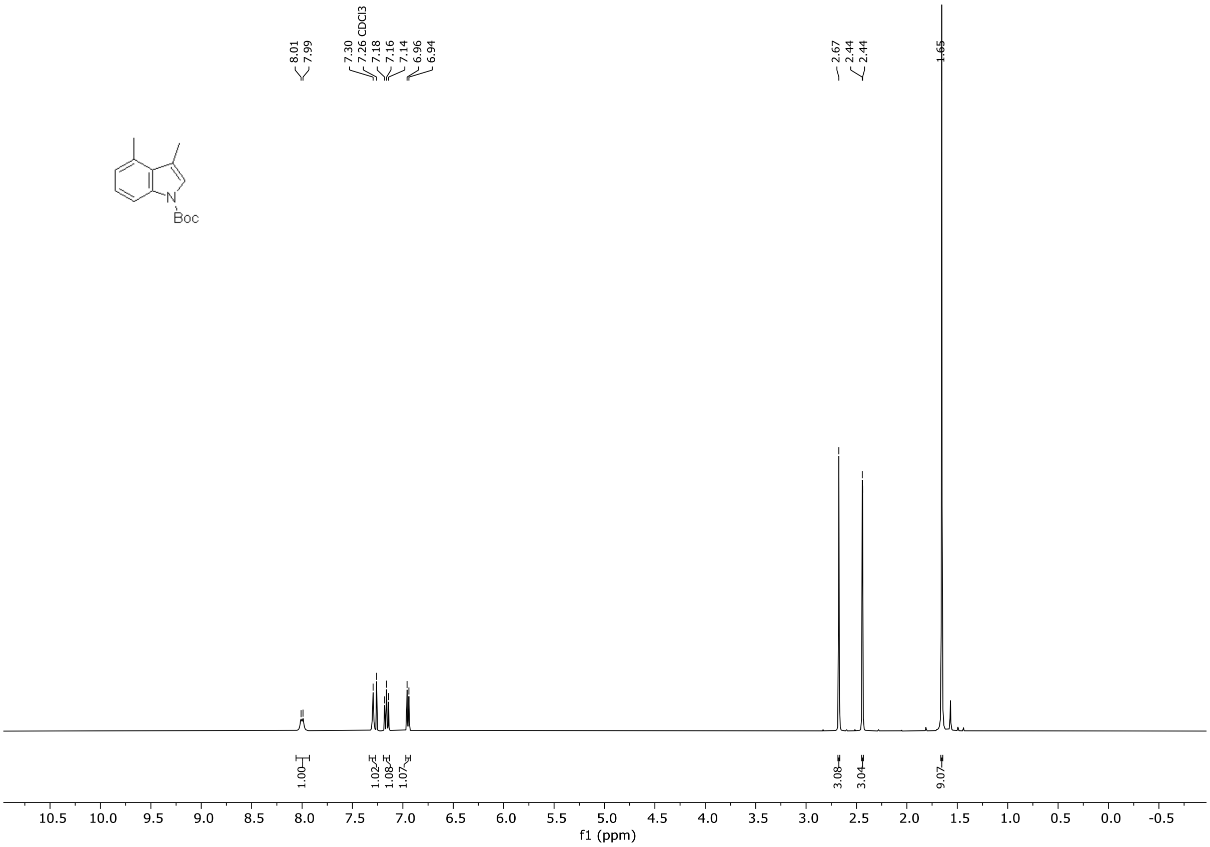

^{13}C NMR (101 MHz, Chloroform-*d*) of compound **1b**

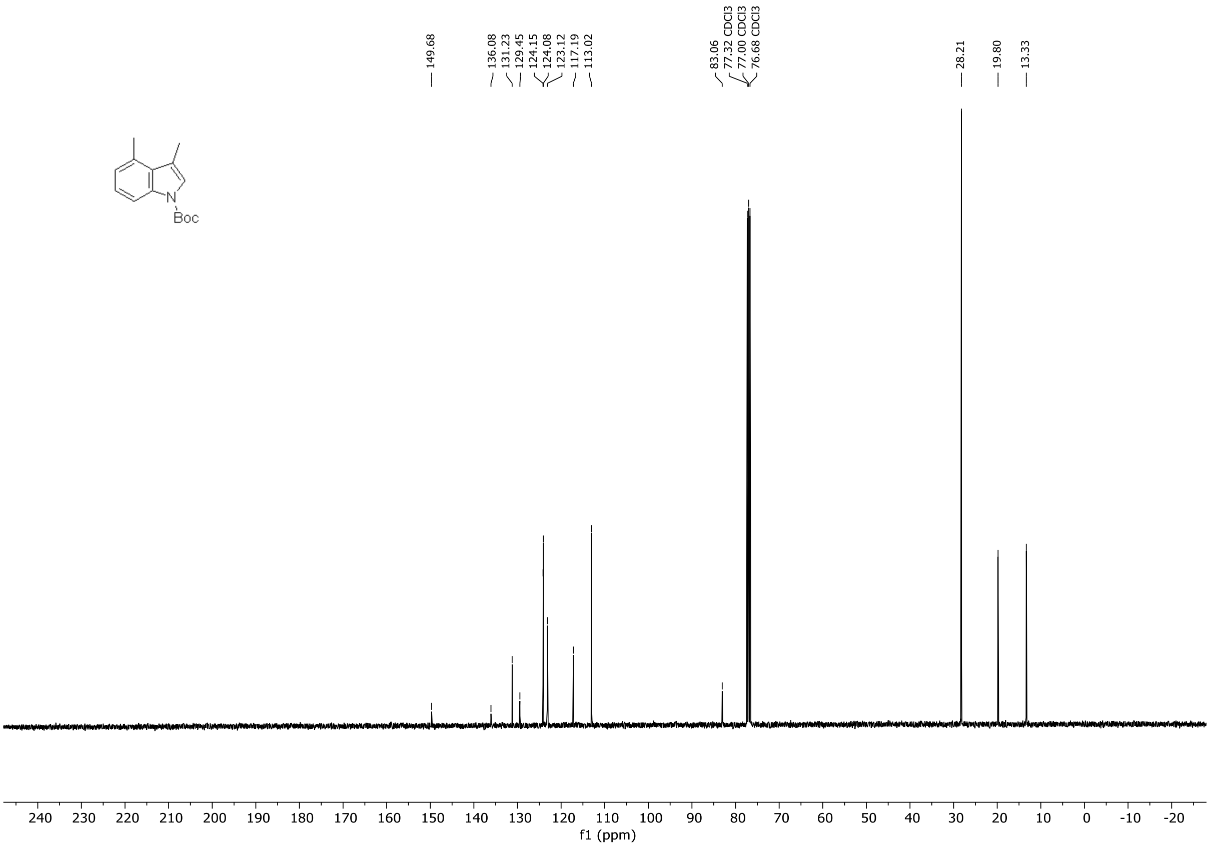

^{1}H NMR (400 MHz, Chloroform-*d*) of compound **1c**

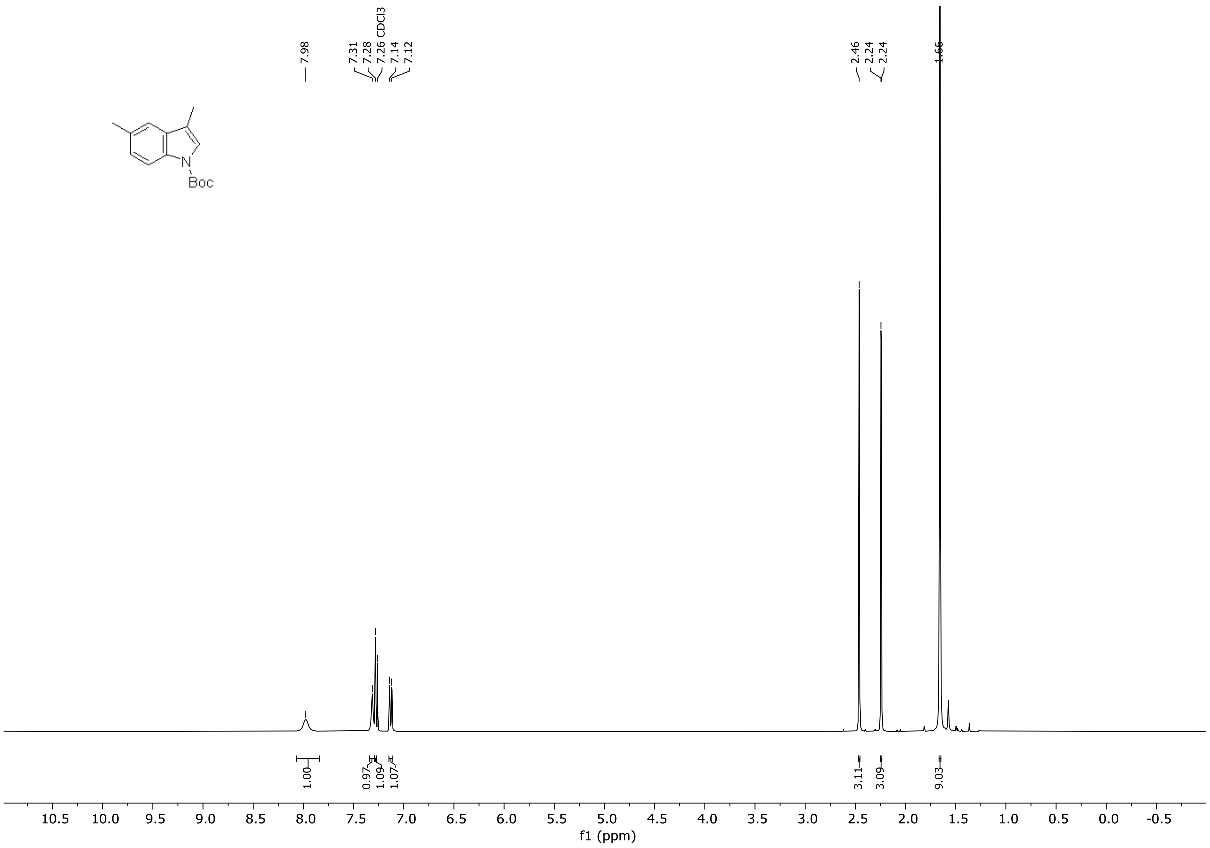

^{13}C NMR (101 MHz, Chloroform-*d*) of compound **1c**

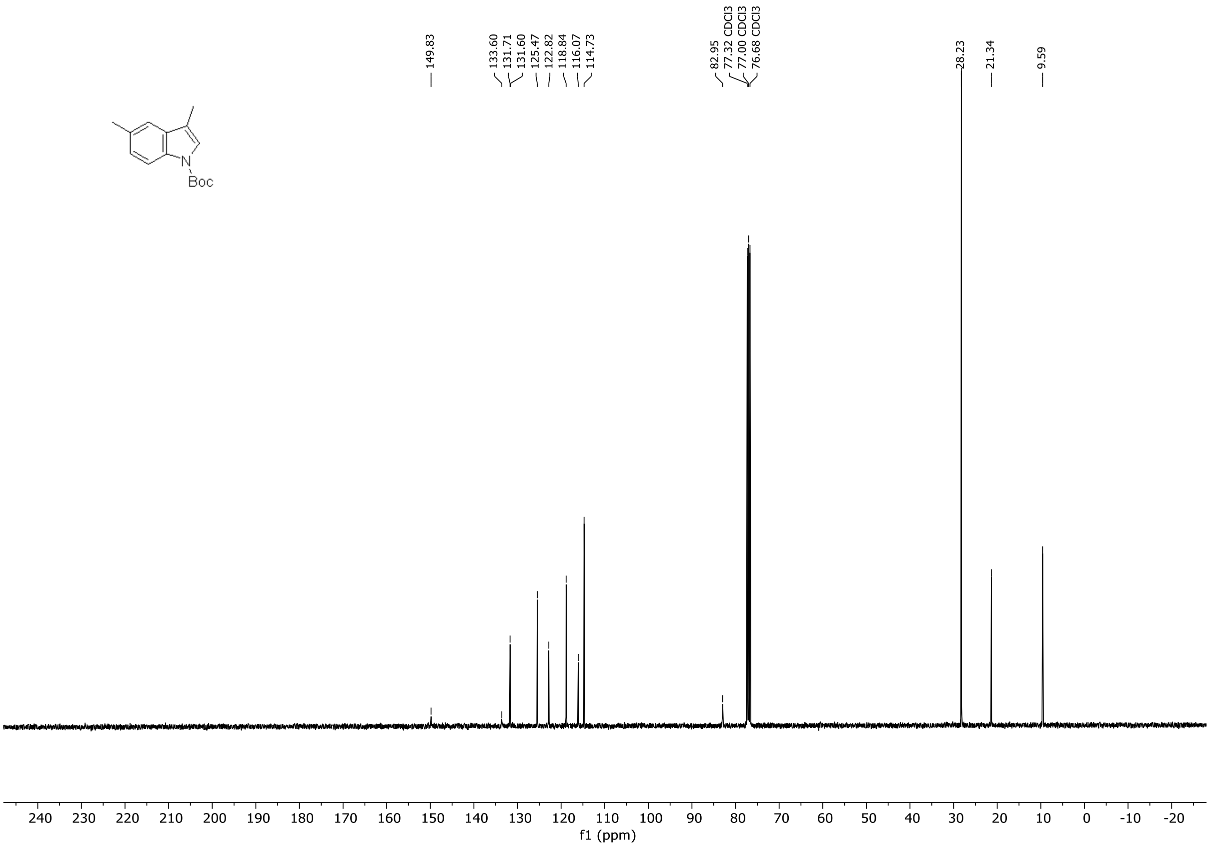

^{1}H NMR (400 MHz, Chloroform-*d*) of compound **1d**

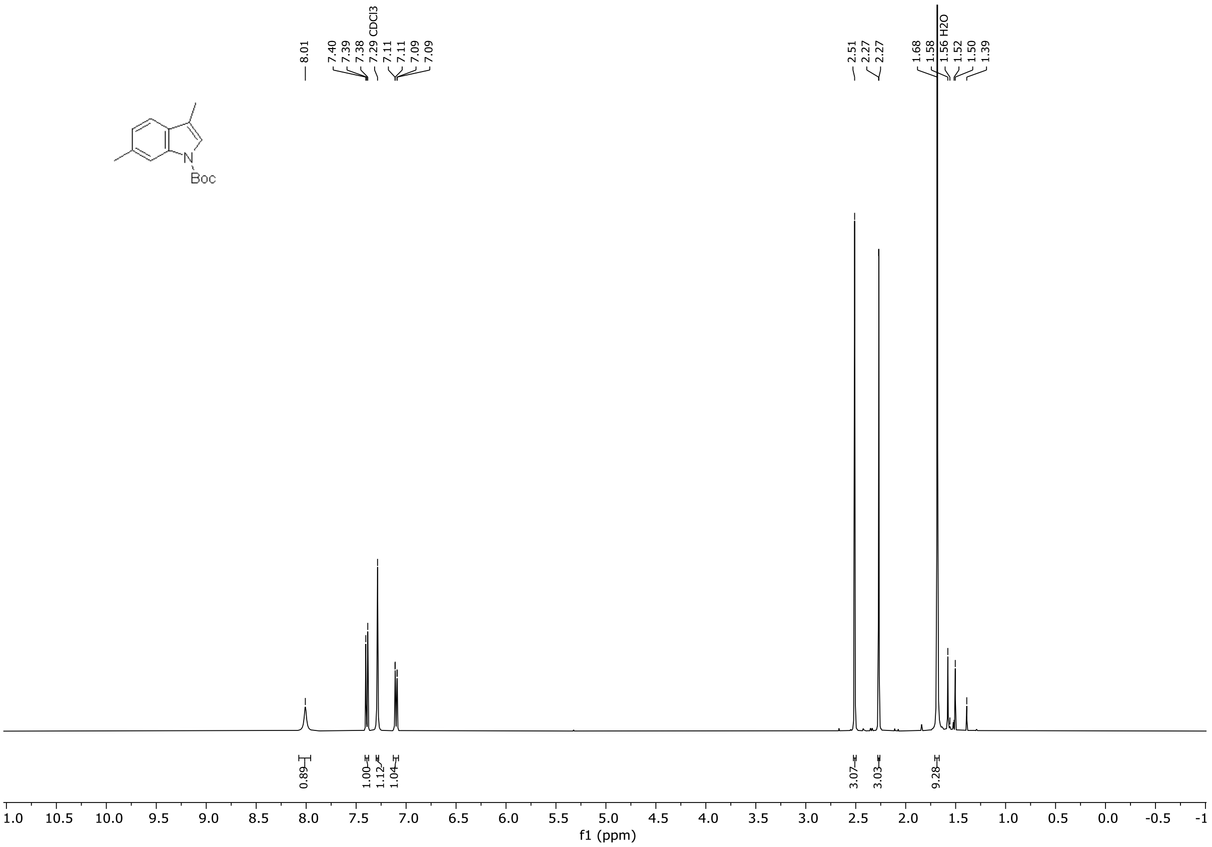

^{13}C NMR (101 MHz, Chloroform-*d*) of compound **1d**

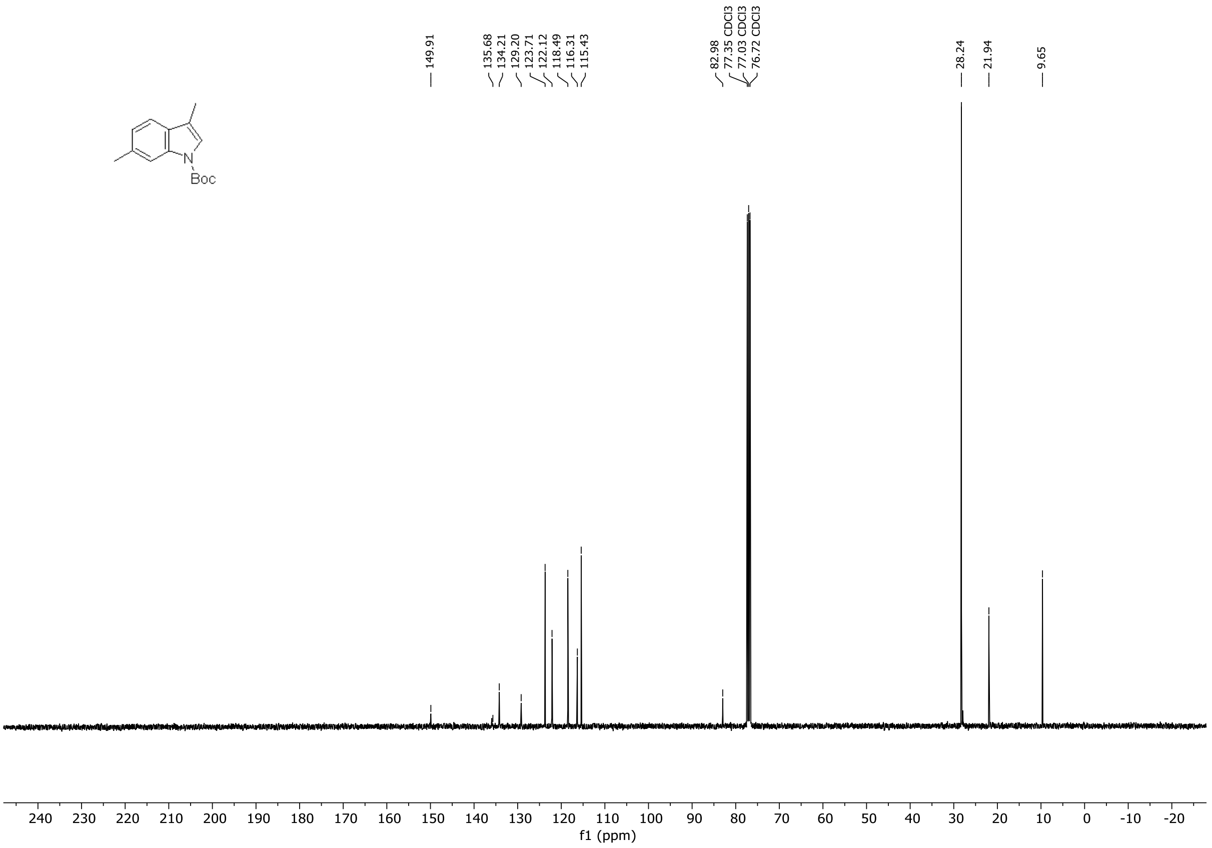

^{1}H NMR (400 MHz, Chloroform-*d*) of compound **1e**

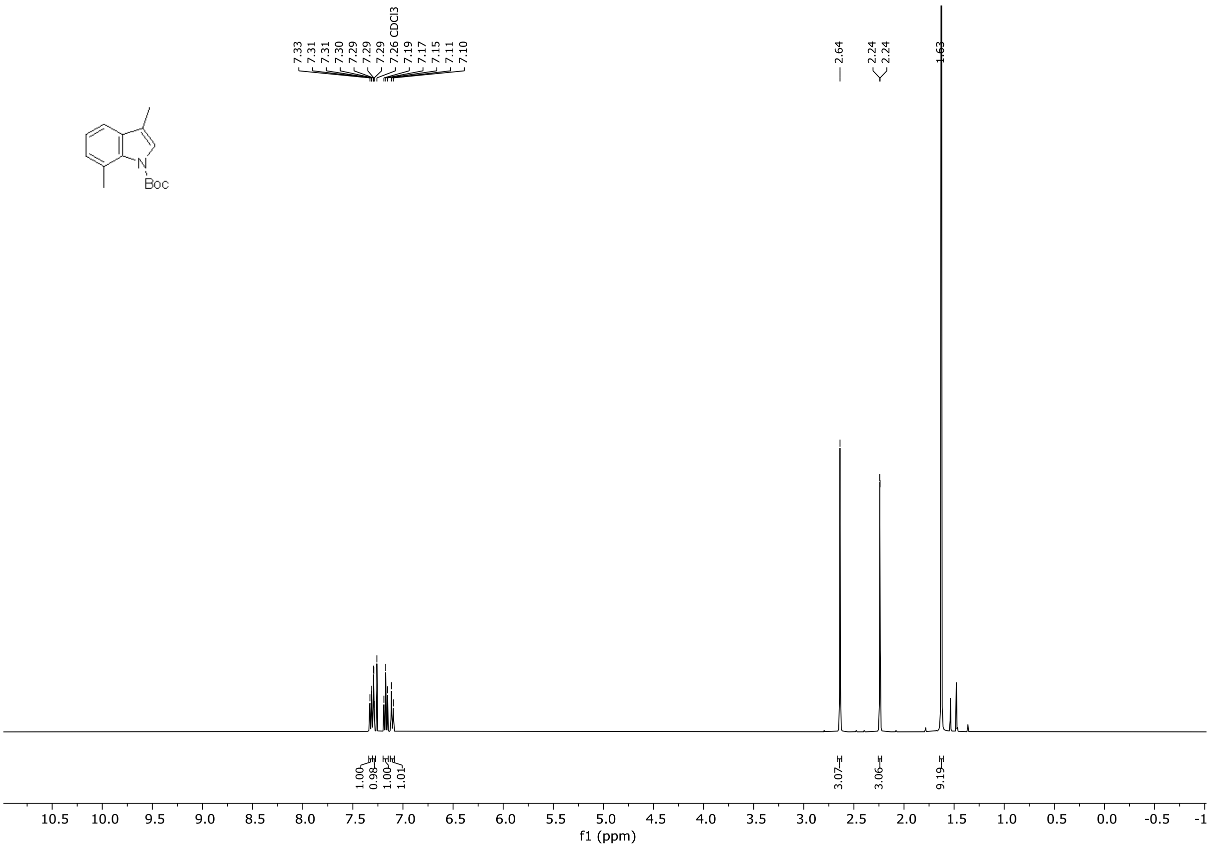

^{13}C NMR (101 MHz, Chloroform-*d*) of compound **1e**

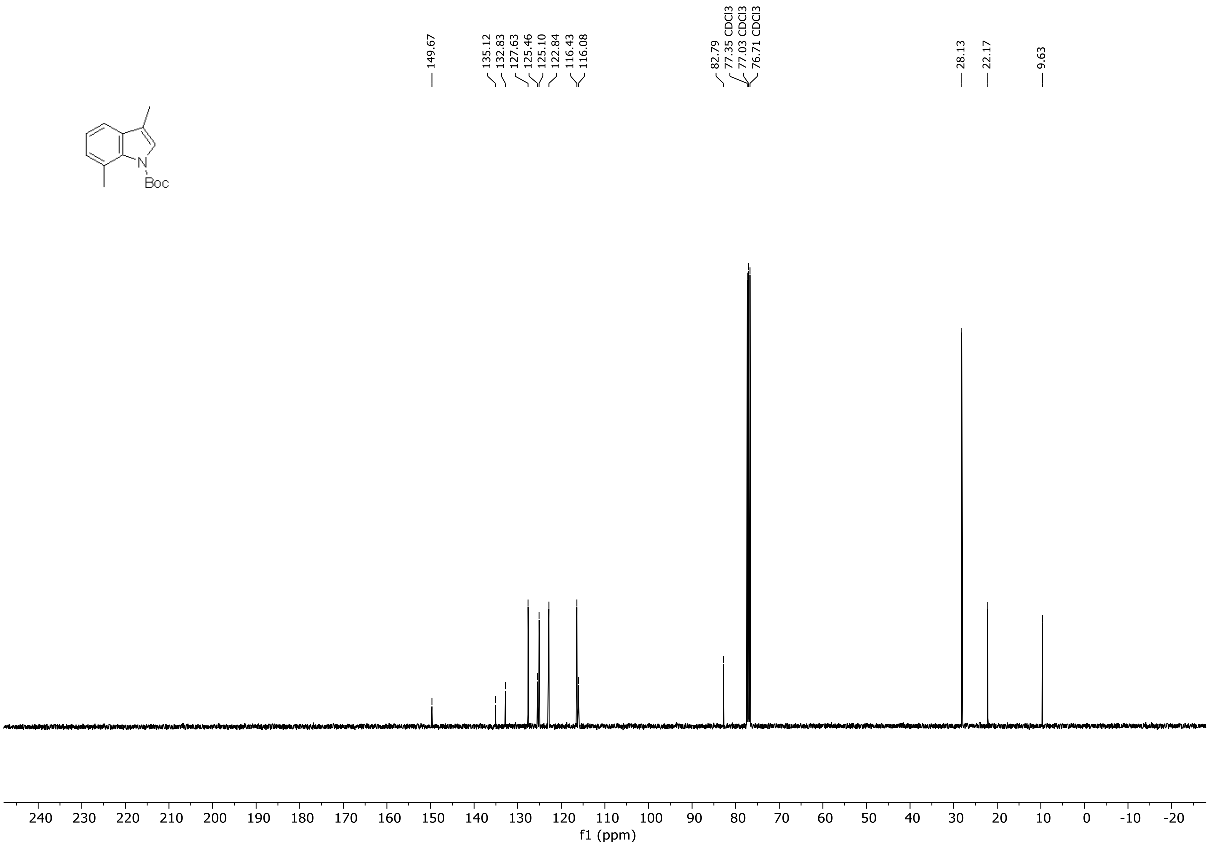

^{1}H NMR (400 MHz, Chloroform-*d*) of compound **1f**

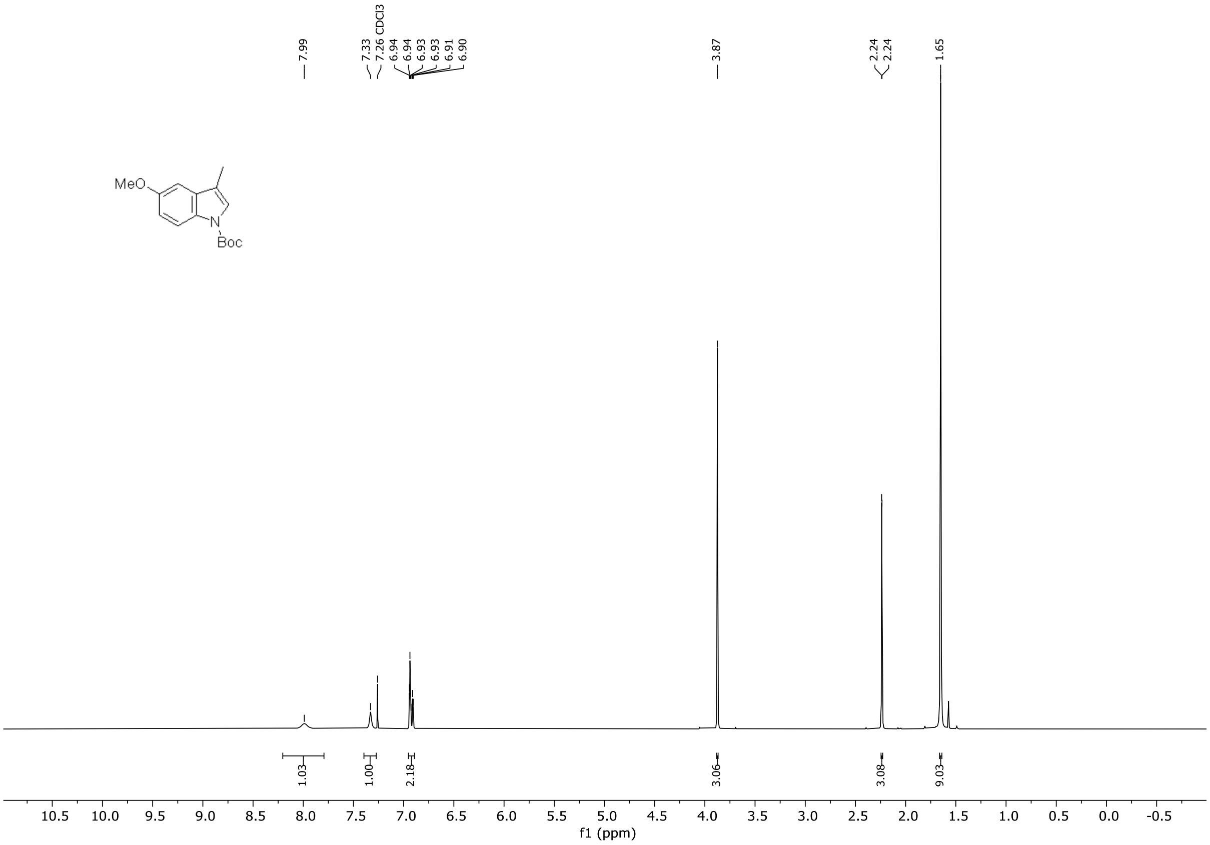

^{13}C NMR (101 MHz, Chloroform-*d*) of compound **1f**

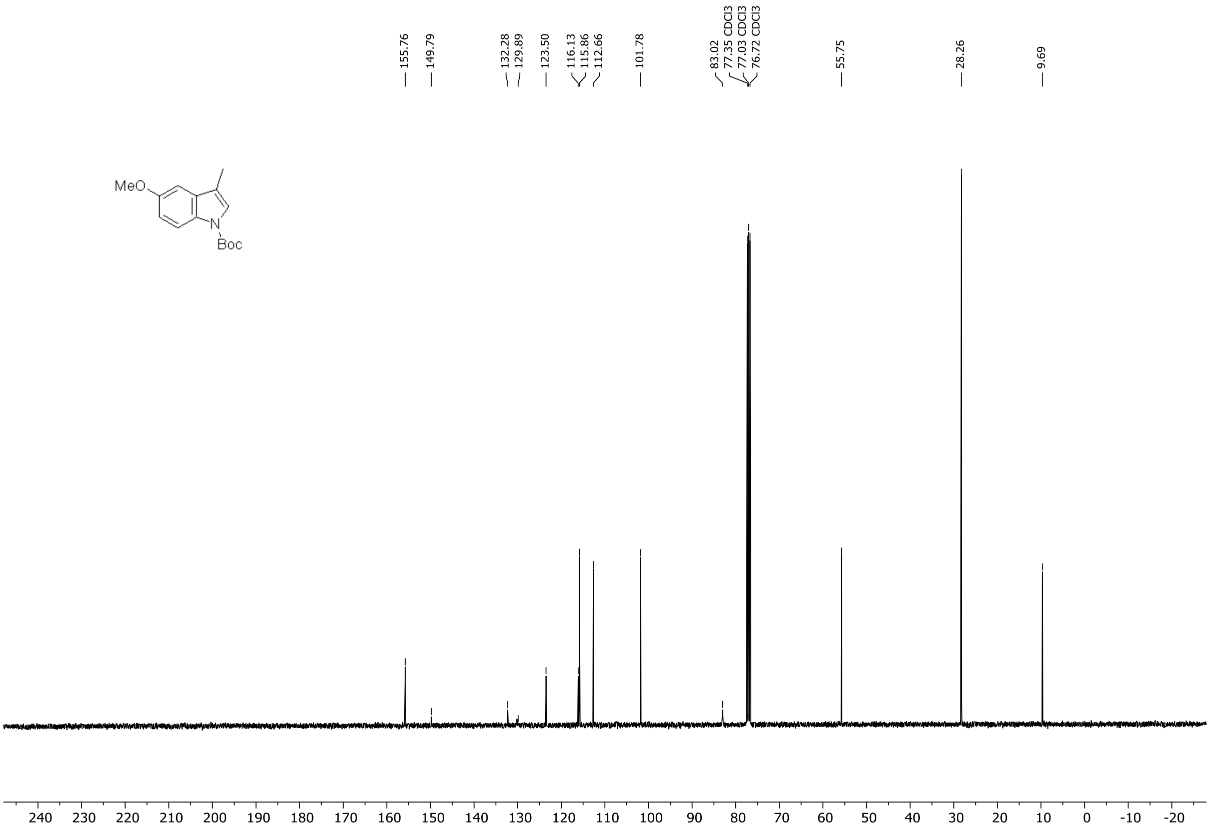

^{1}H NMR (400 MHz, Chloroform-*d*) of compound **S1**

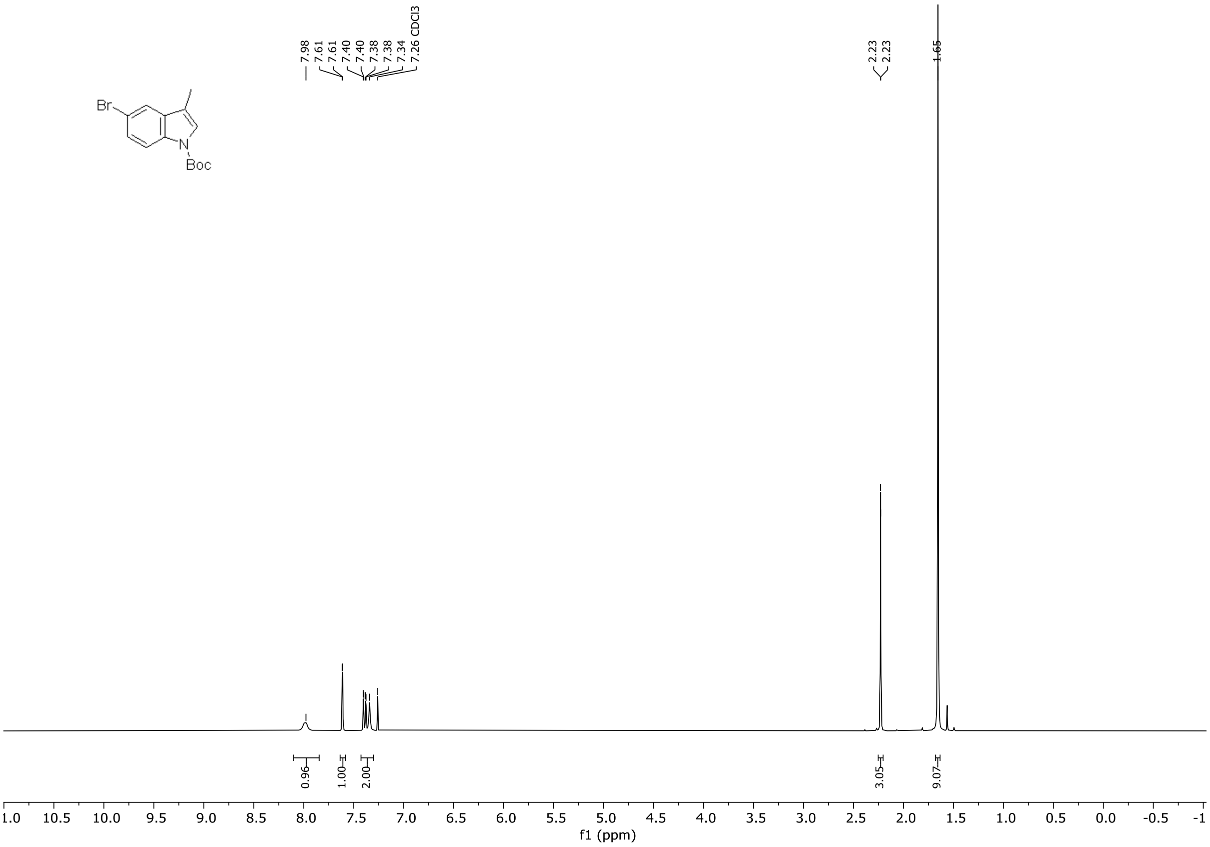

^{13}C NMR (101 MHz, Chloroform-*d*) of compound **S1**

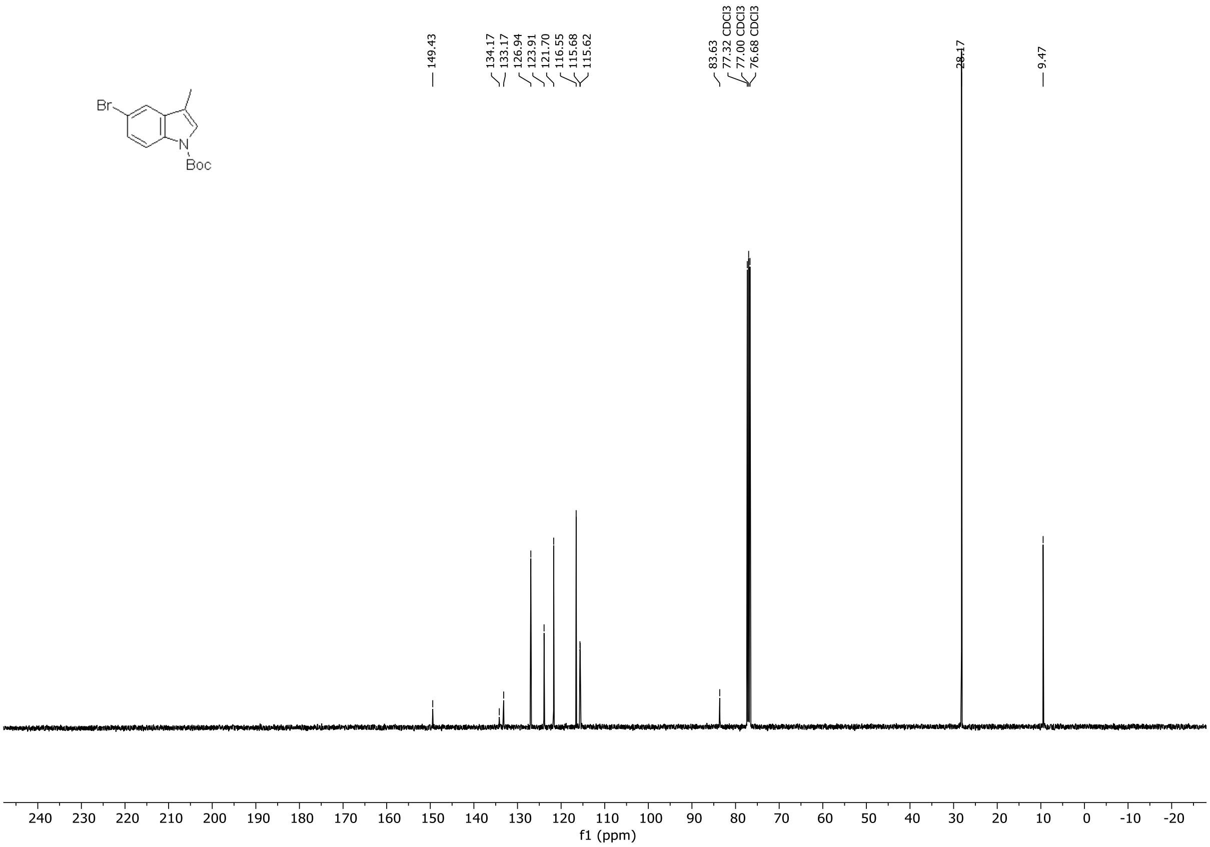

^{1}H NMR (400 MHz, Chloroform-*d*) of compound **1g**

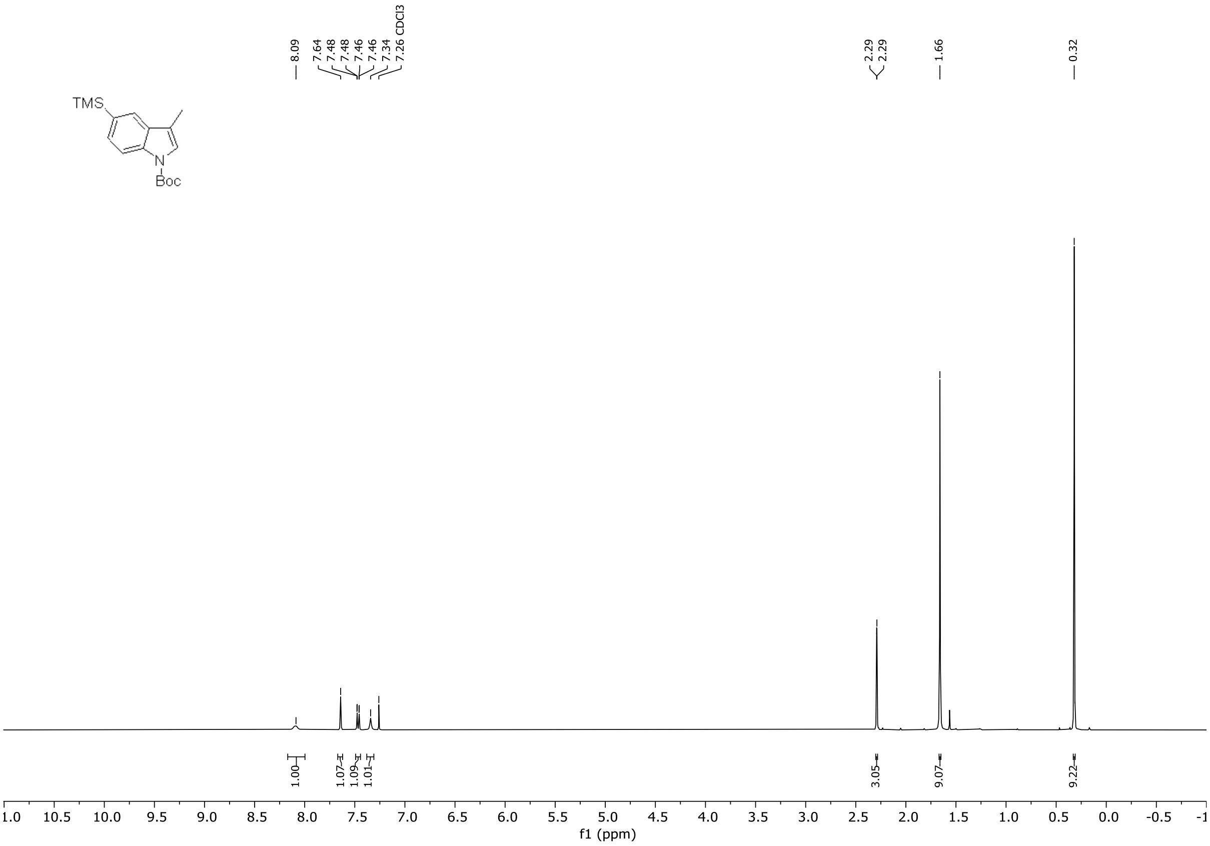

^{13}C NMR (101 MHz, Chloroform-*d*) of compound **1g**

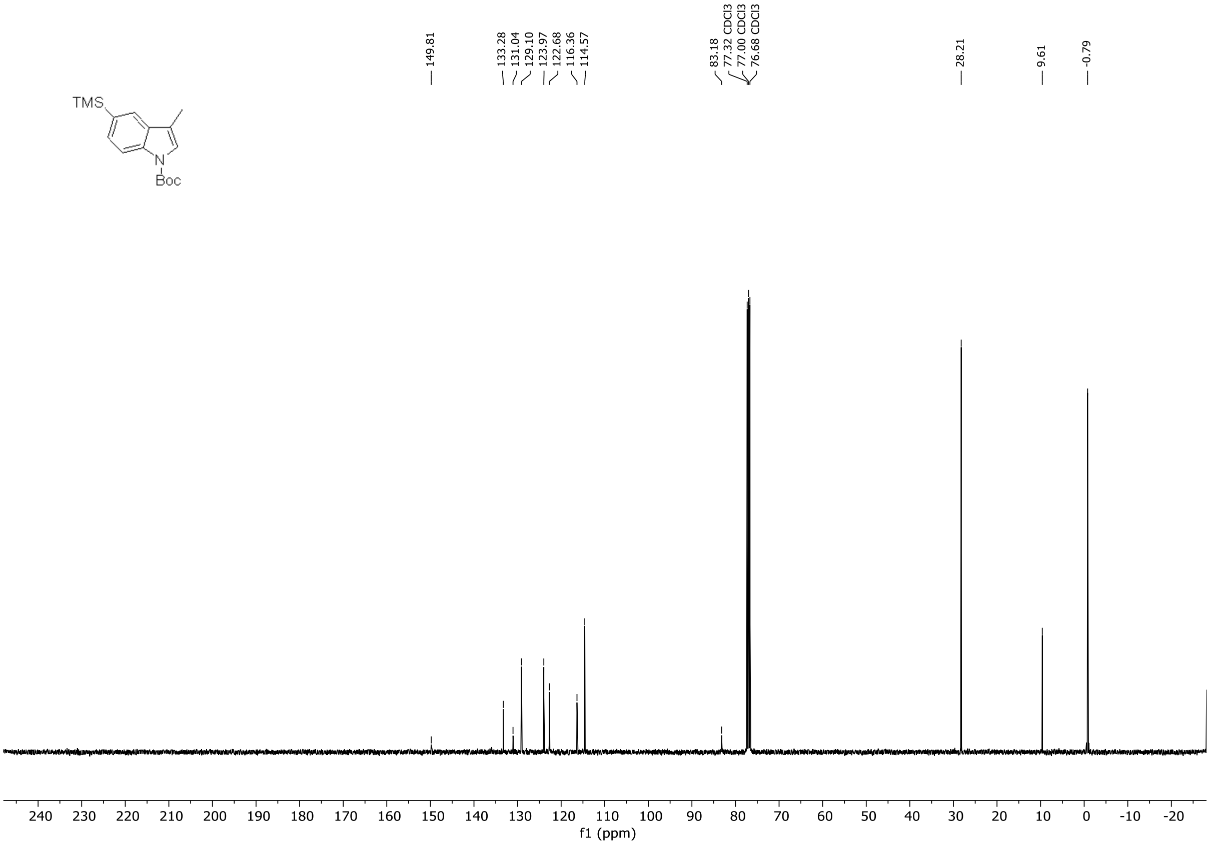

^{1}H NMR (400 MHz, Chloroform-*d*) of compound **1h**

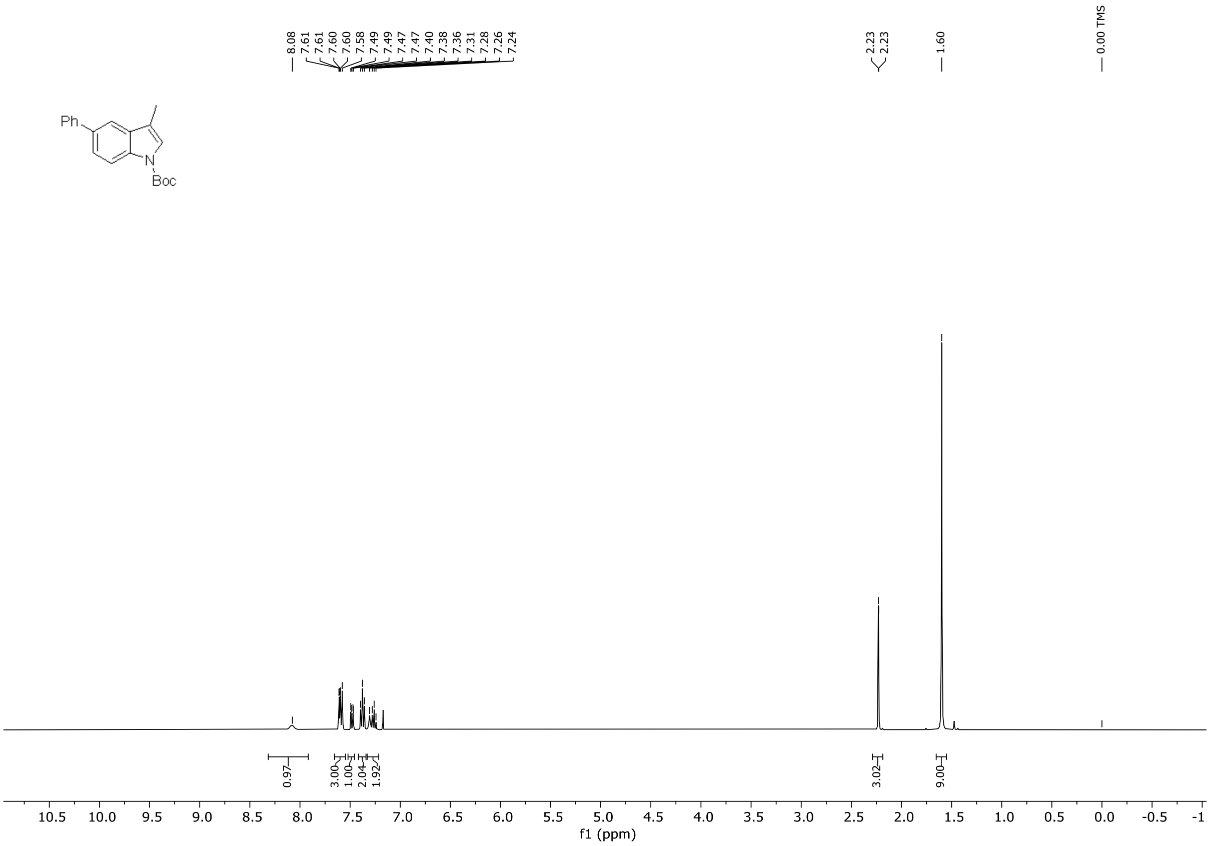

^{13}C NMR (101 MHz, Chloroform-*d*) of compound **1h**

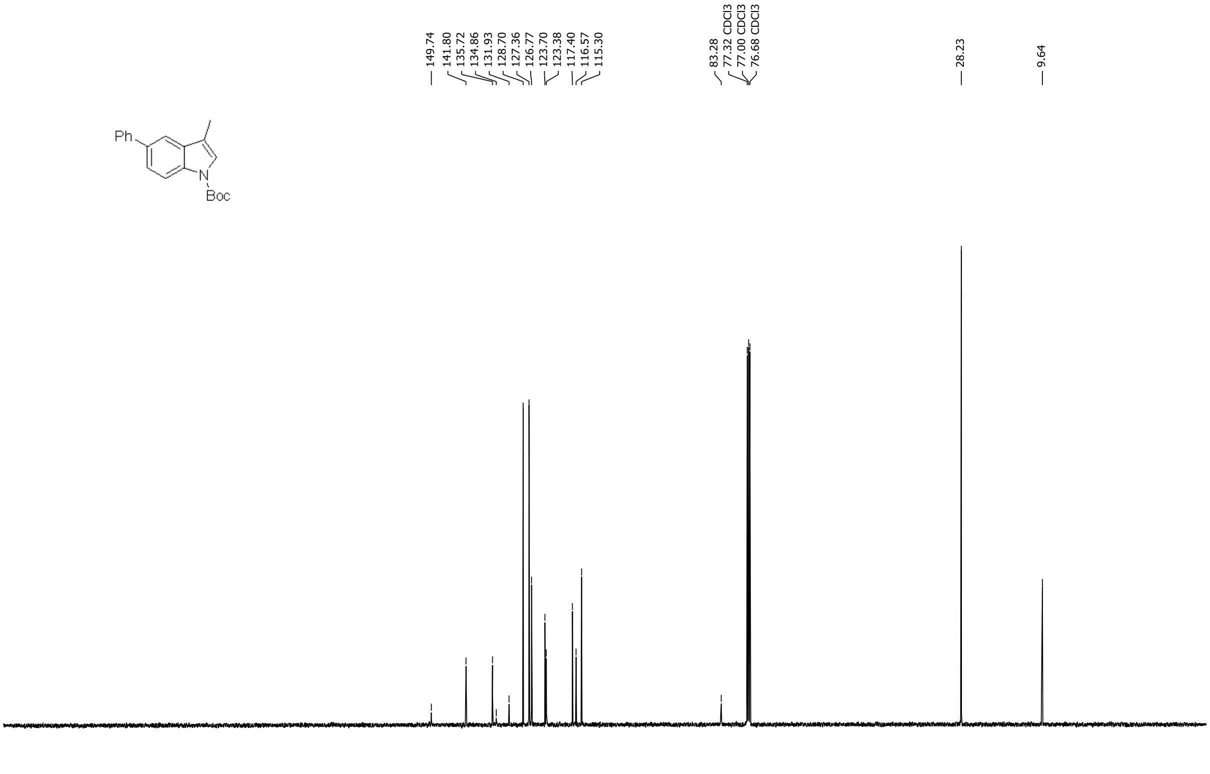

^{1}H NMR (400 MHz, Chloroform-*d*) of compound **1i**

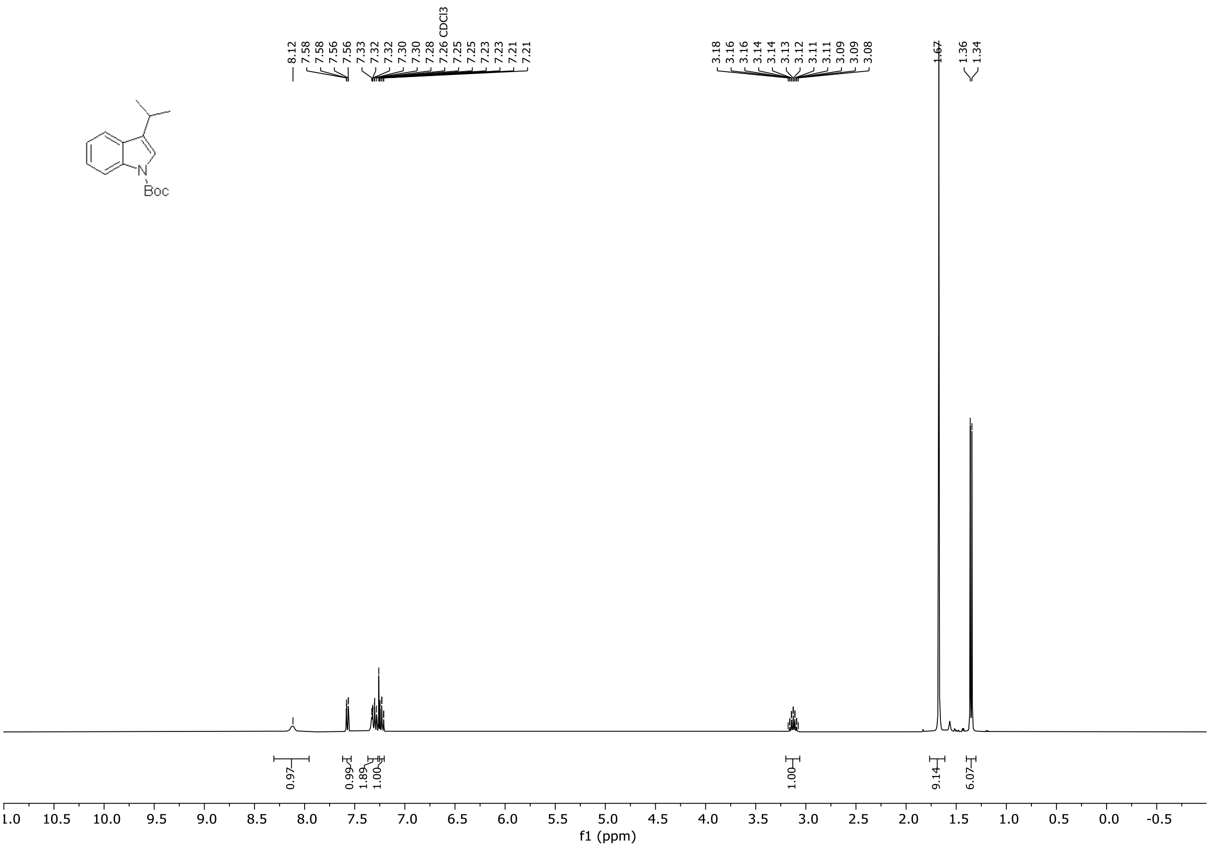

^{13}C NMR (101 MHz, Chloroform-*d*) of compound **1i**

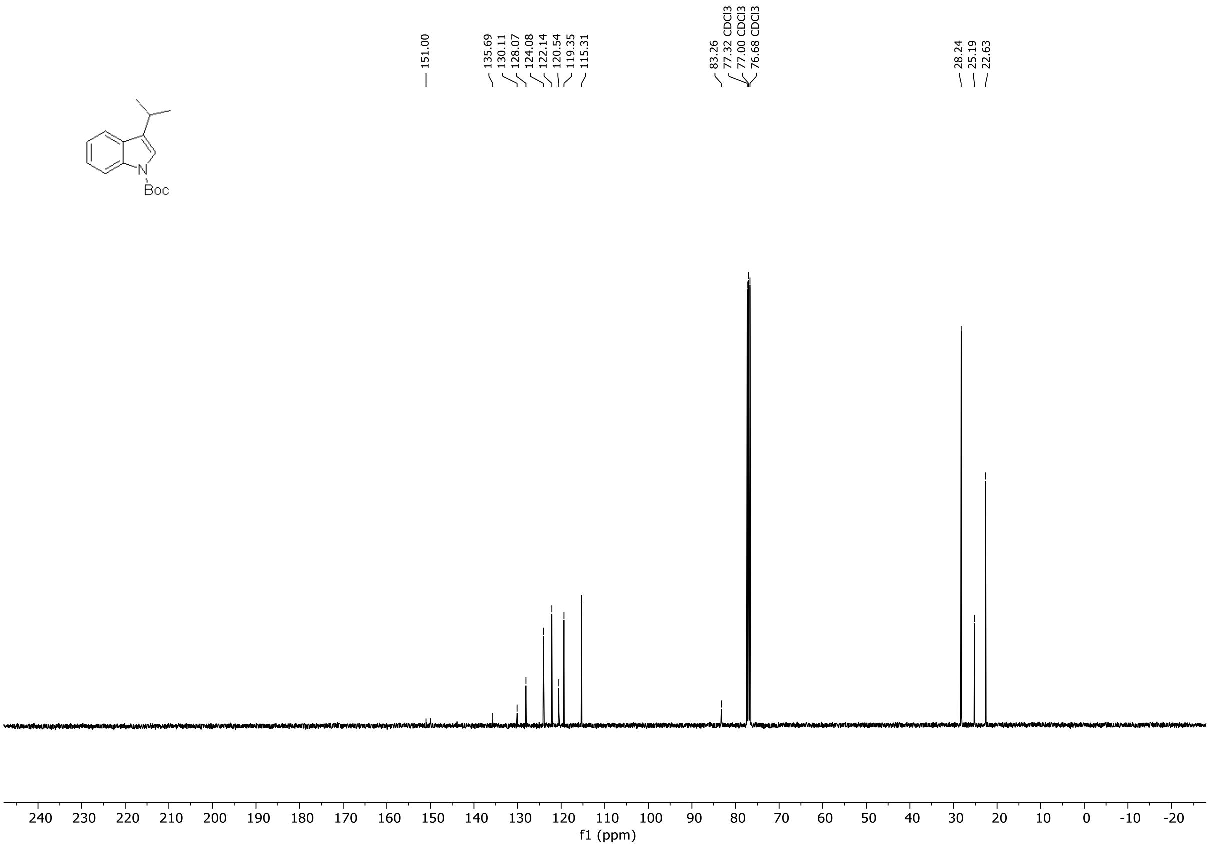

^{1}H NMR (400 MHz, Chloroform-*d*) of compound **1j**

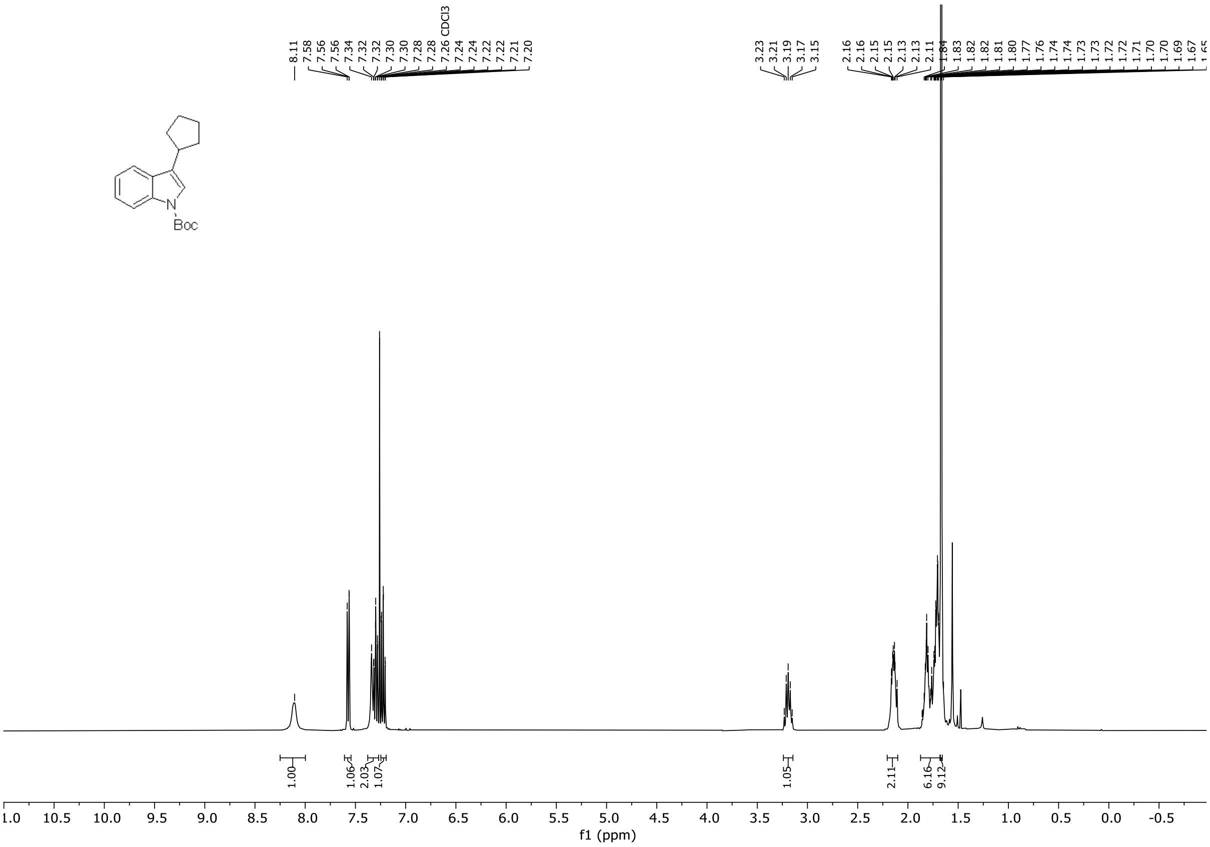

^{13}C NMR (101 MHz, Chloroform-*d*) of compound **1j**

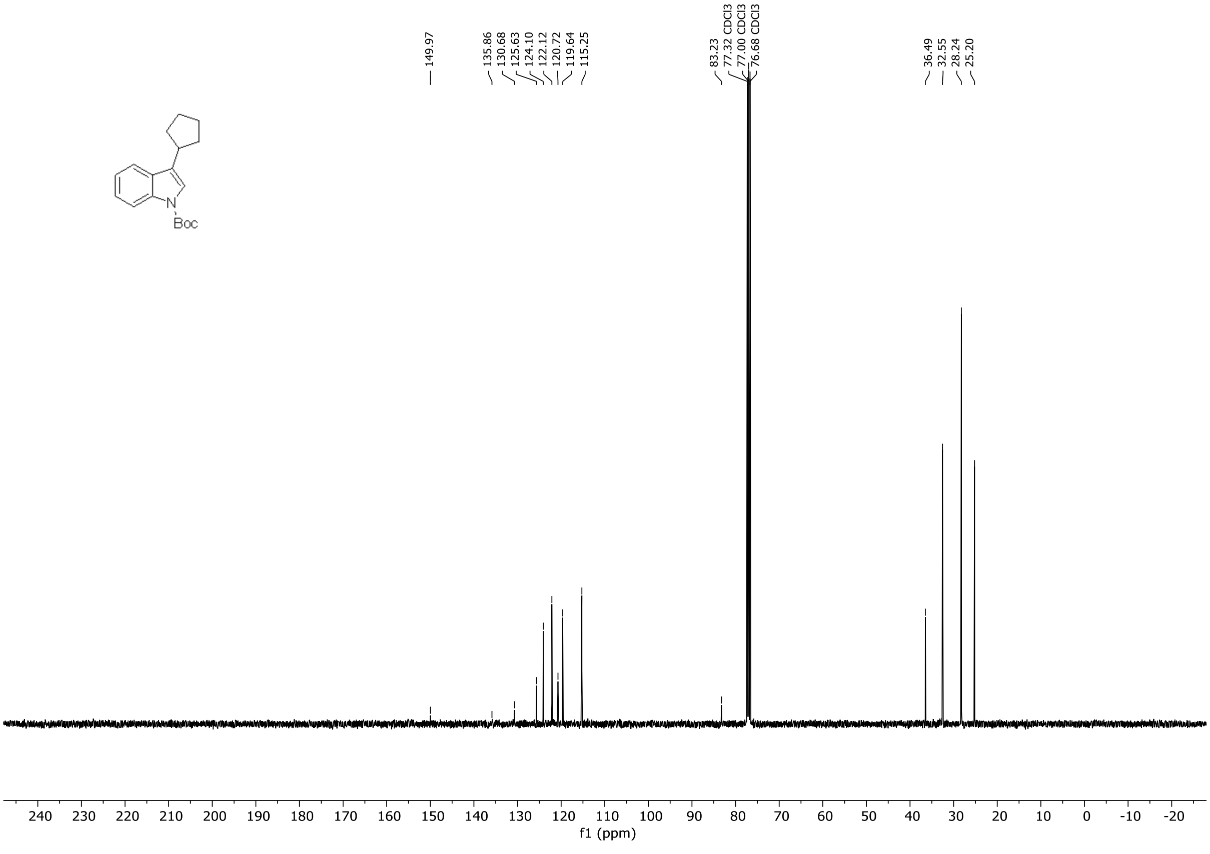

^1H NMR (400 MHz, Chloroform-*d*) of compound **1k**

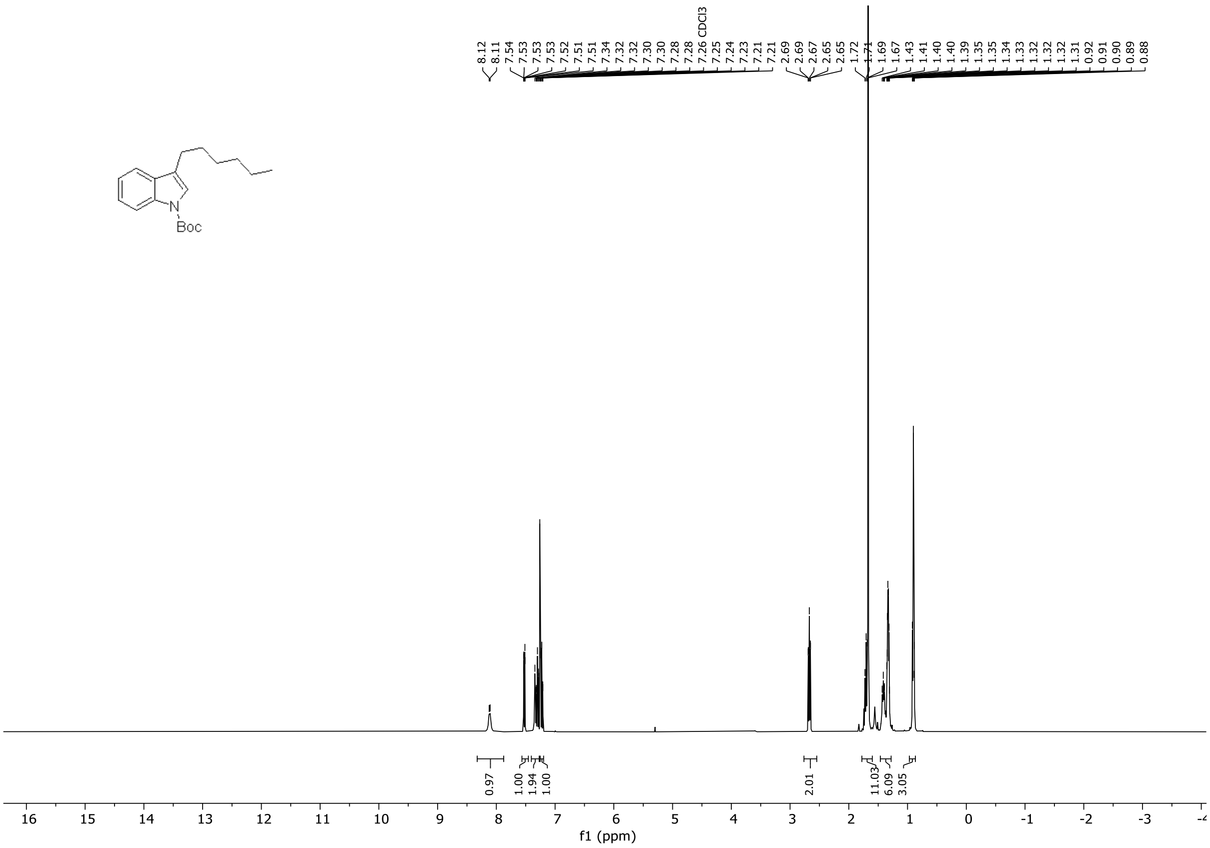

^{13}C NMR (101 MHz, Chloroform-*d*) of compound **1k**

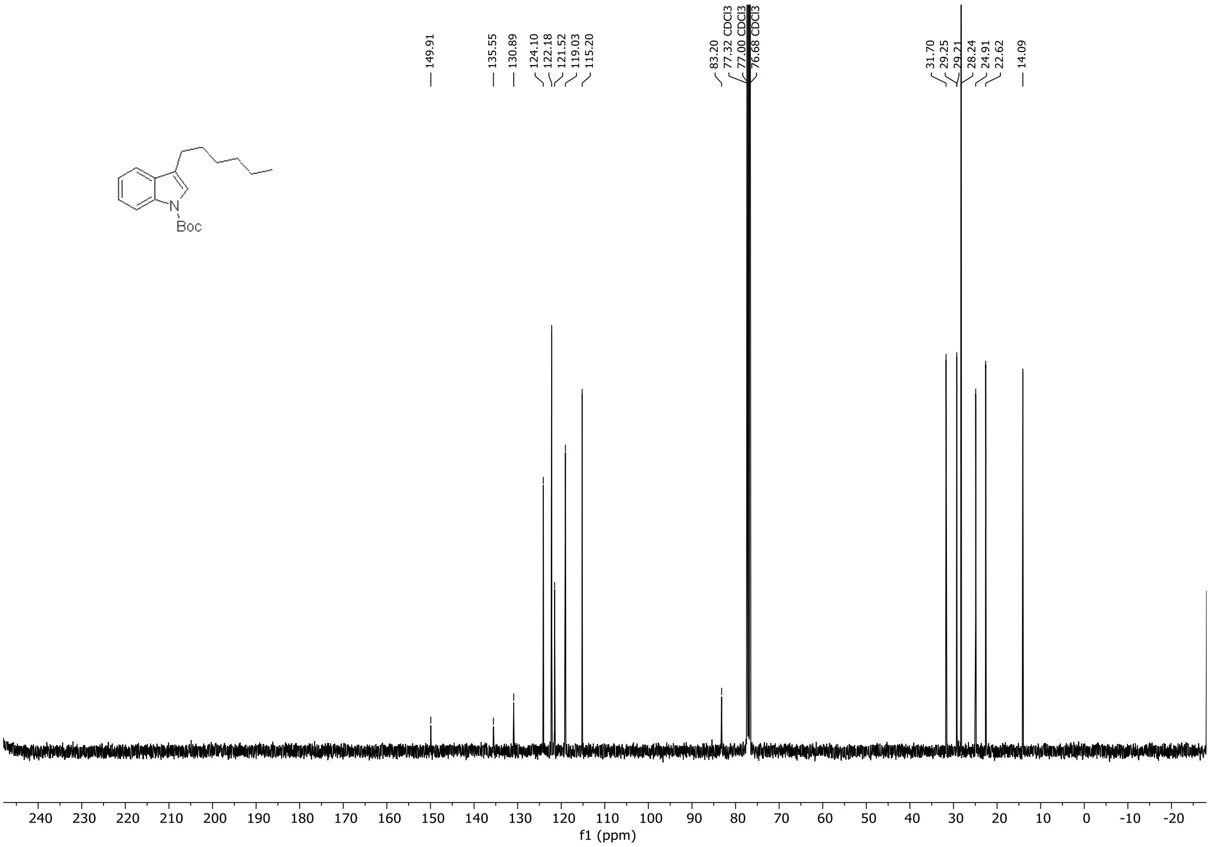

^{1}H NMR (400 MHz, Chloroform-*d*) of compound **1l**

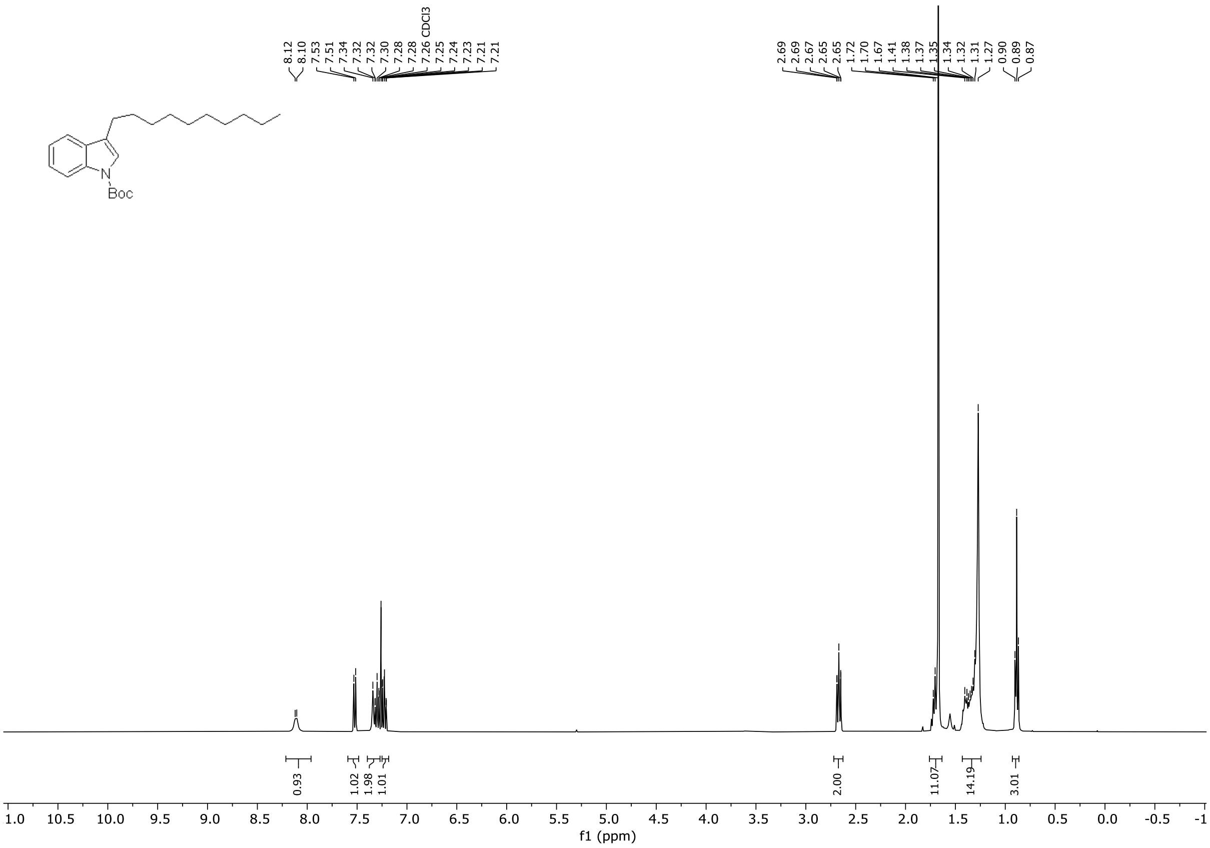

^{13}C NMR (101 MHz, Chloroform-*d*) of compound **1l**

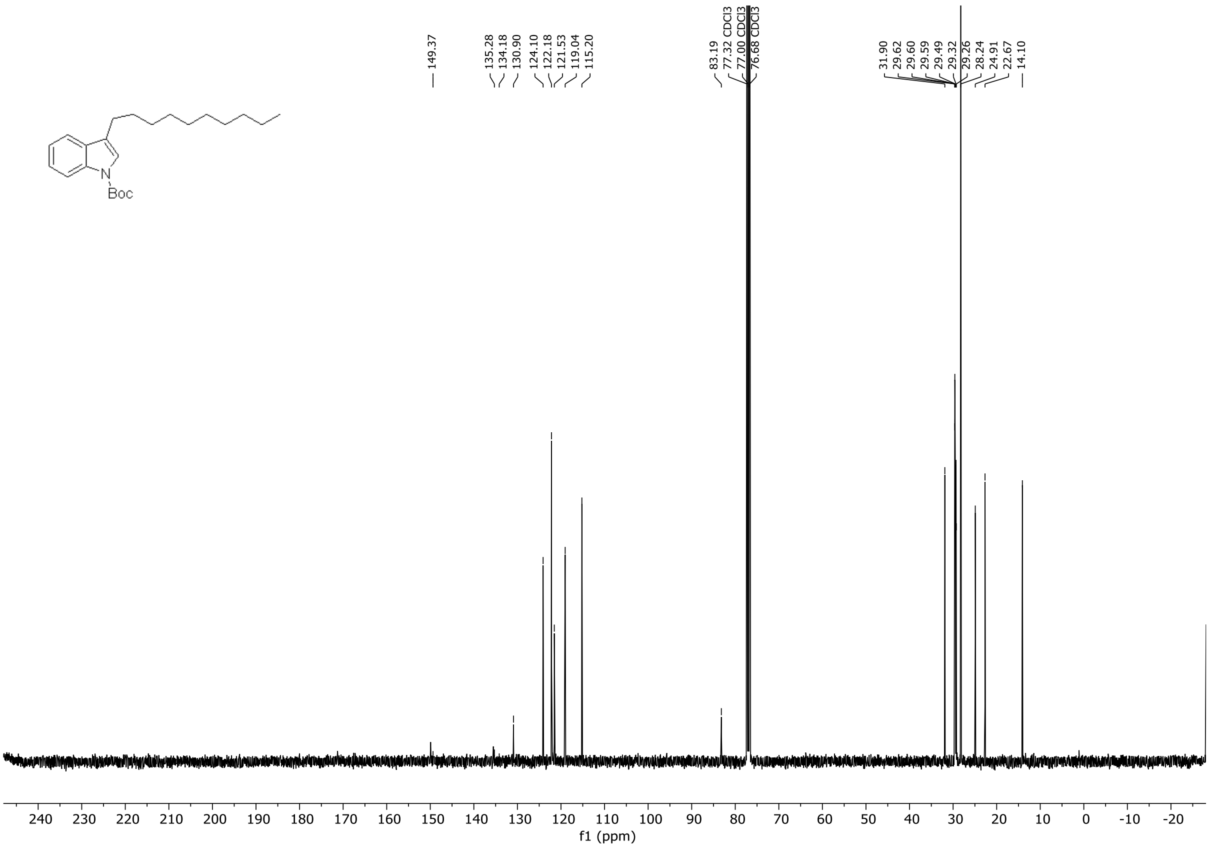

^{1}H NMR (400 MHz, Chloroform-*d*) of compound **1m**

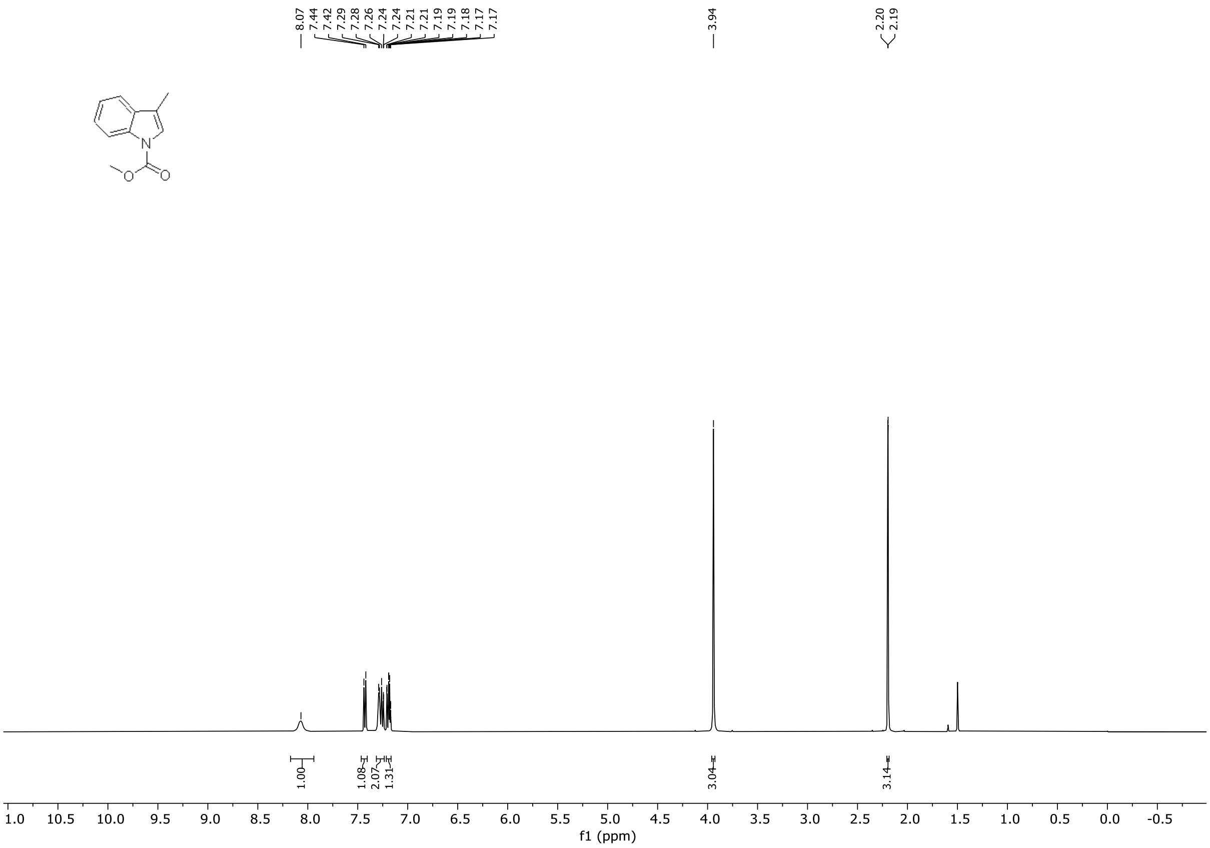

^{13}C NMR (101 MHz, Chloroform-*d*) of compound **1m**

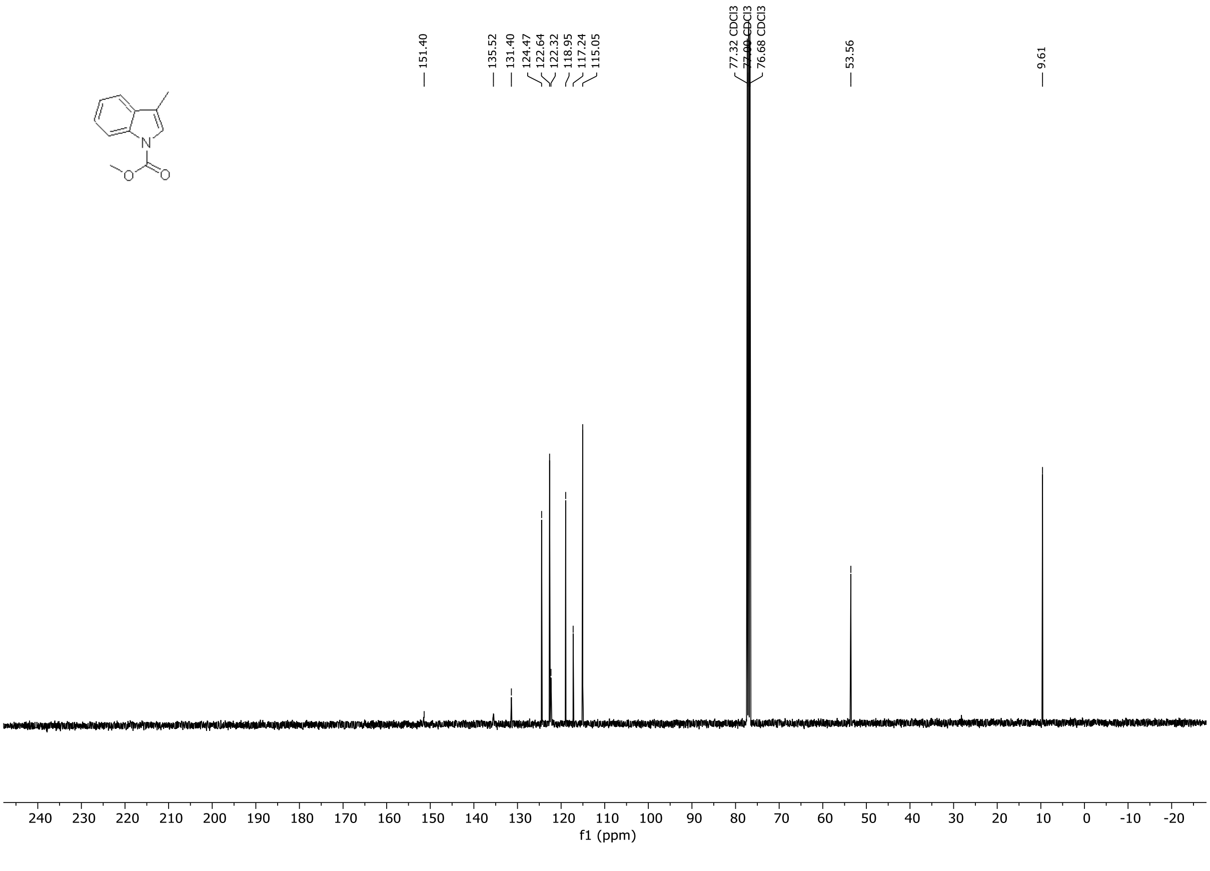

^{1}H NMR (400 MHz, Chloroform-*d*) of compound **4a**

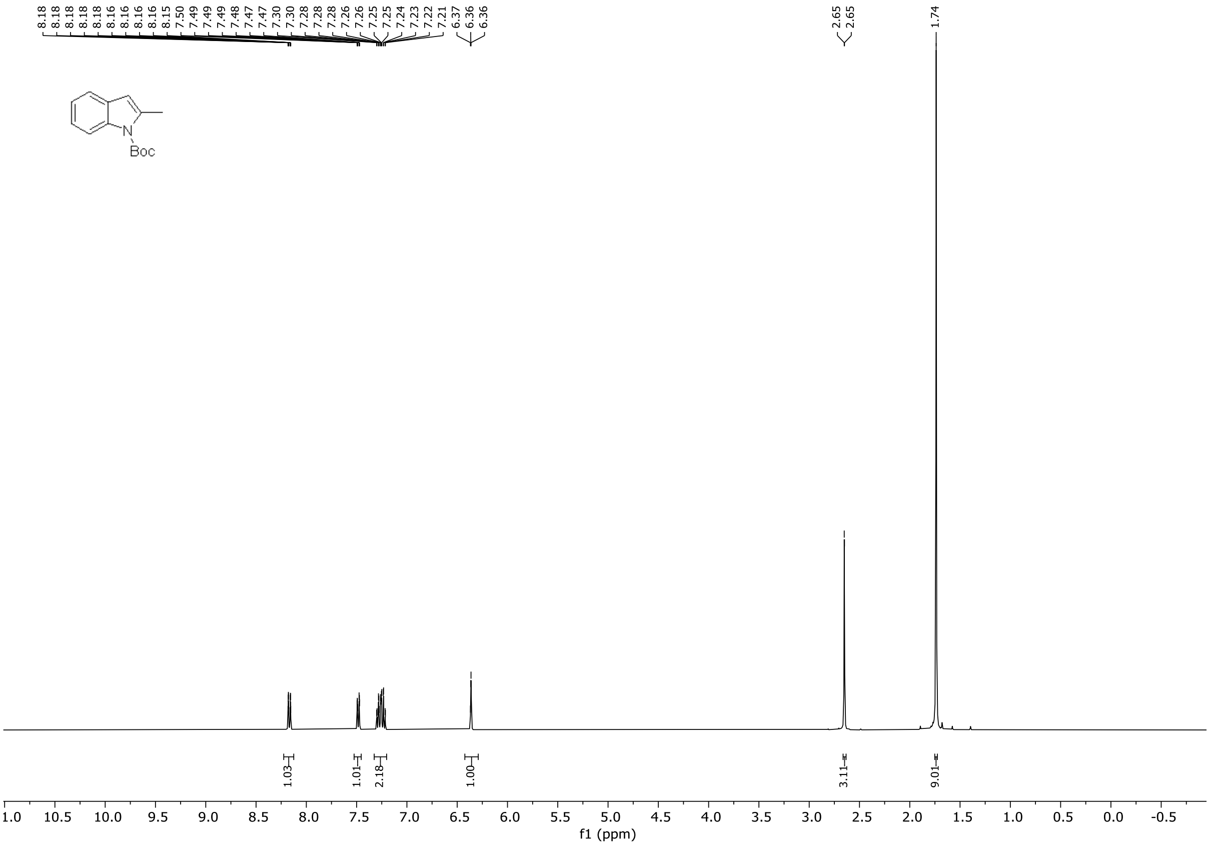

^{13}C NMR (101 MHz, Chloroform-*d*) of compound **4a**

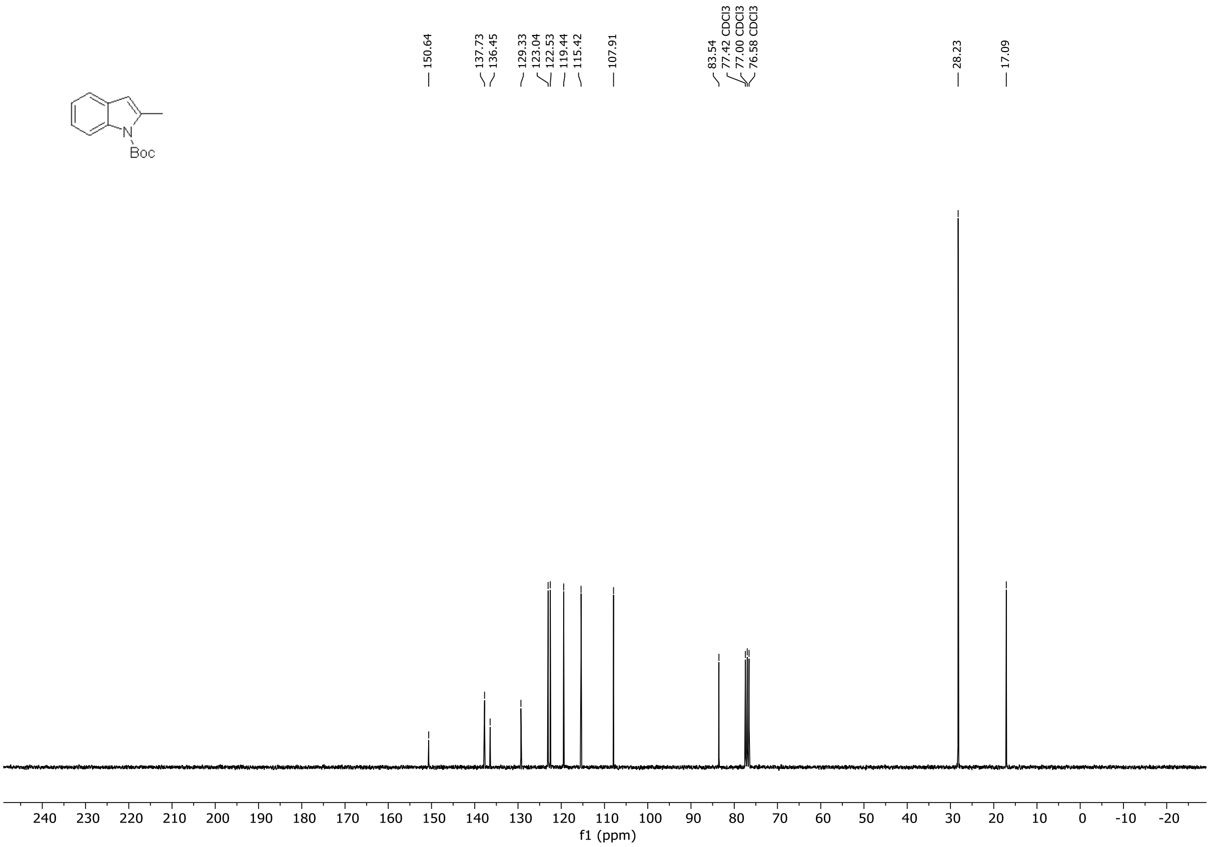

^{1}H NMR (400 MHz, Chloroform-*d*) of compound **4b**

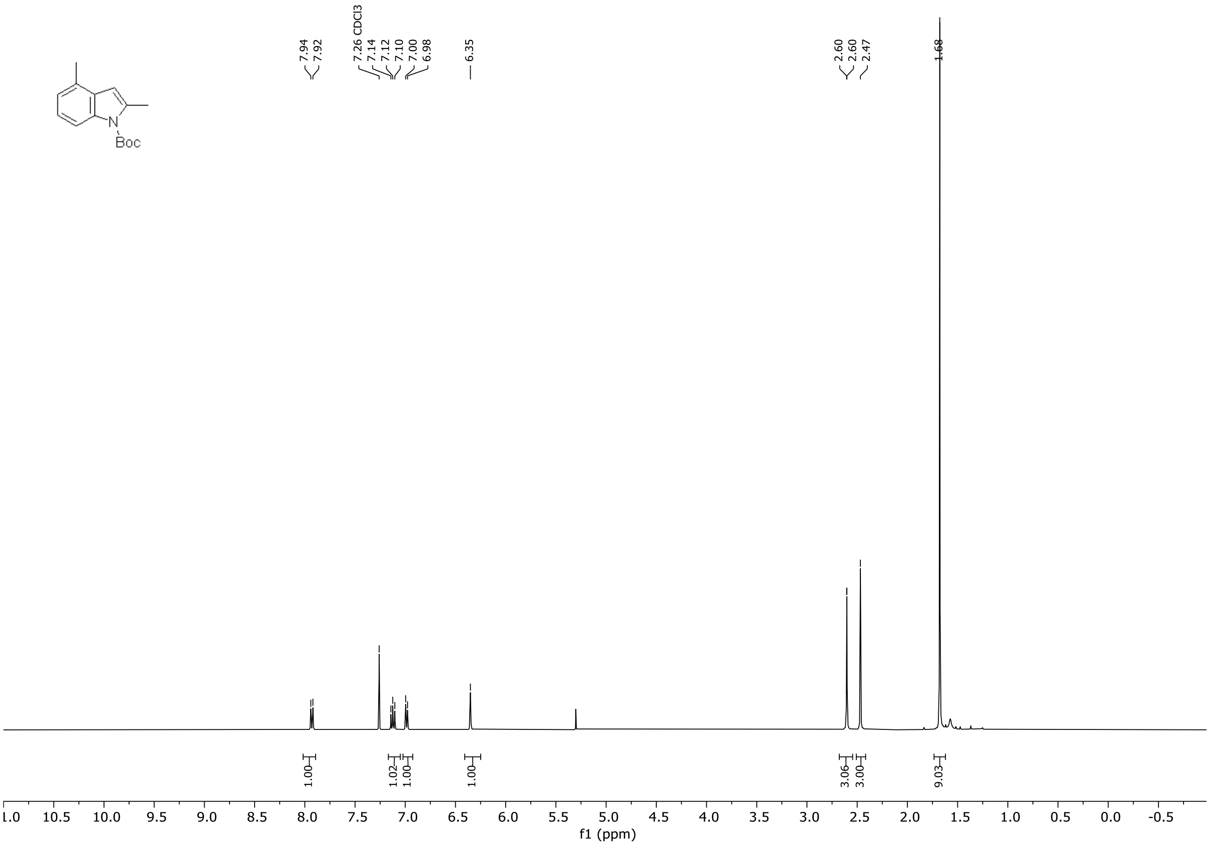

^{13}C NMR (101 MHz, Chloroform-*d*) of compound **4b**

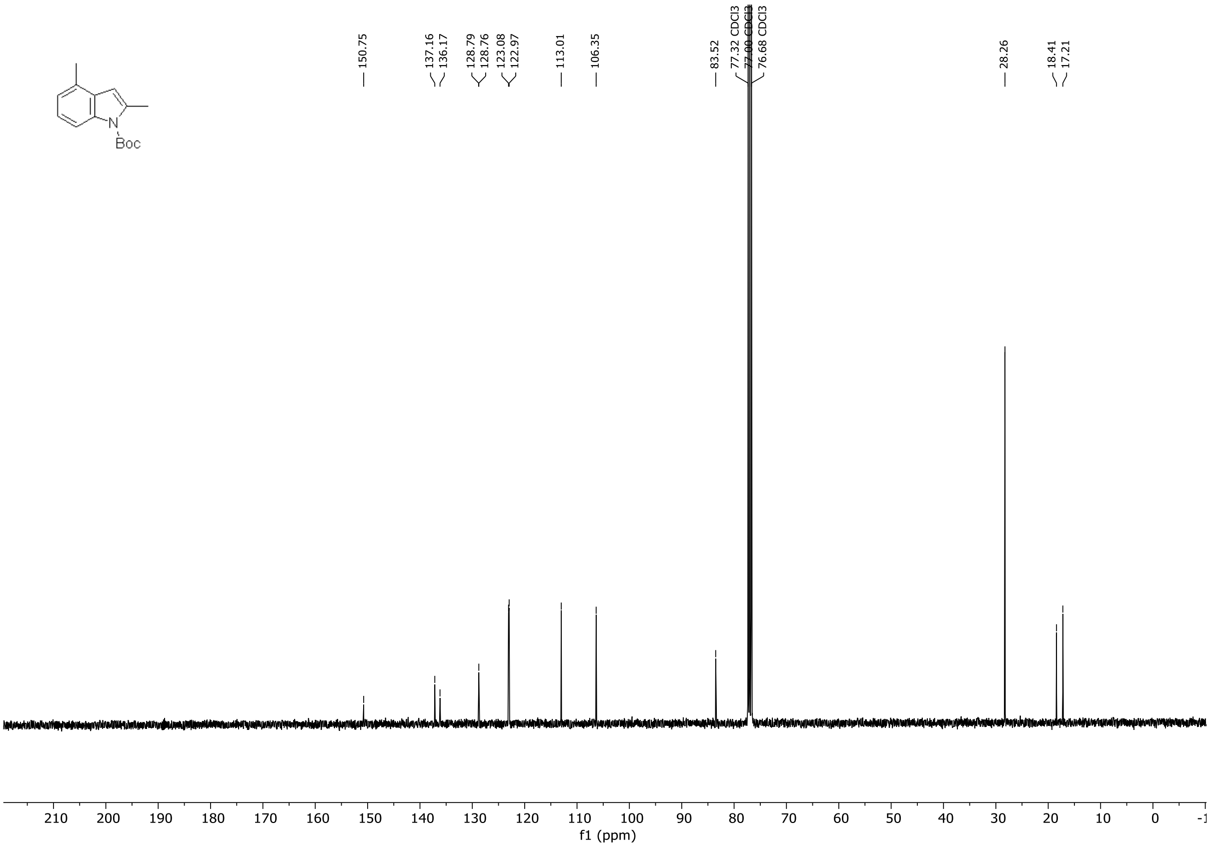

^{1}H NMR (400 MHz, Chloroform-*d*) of compound **4c**

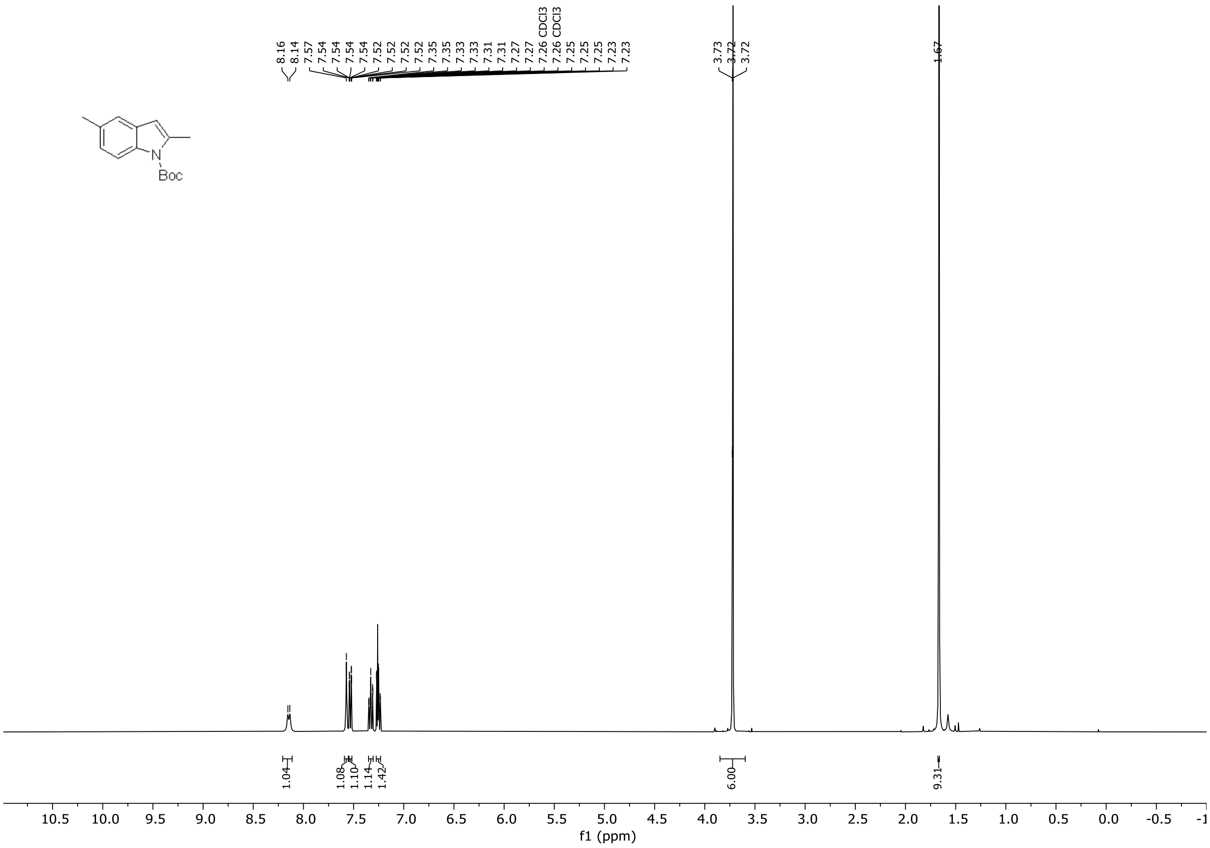

^{13}C NMR (101 MHz, Chloroform-*d*) of compound **4c**

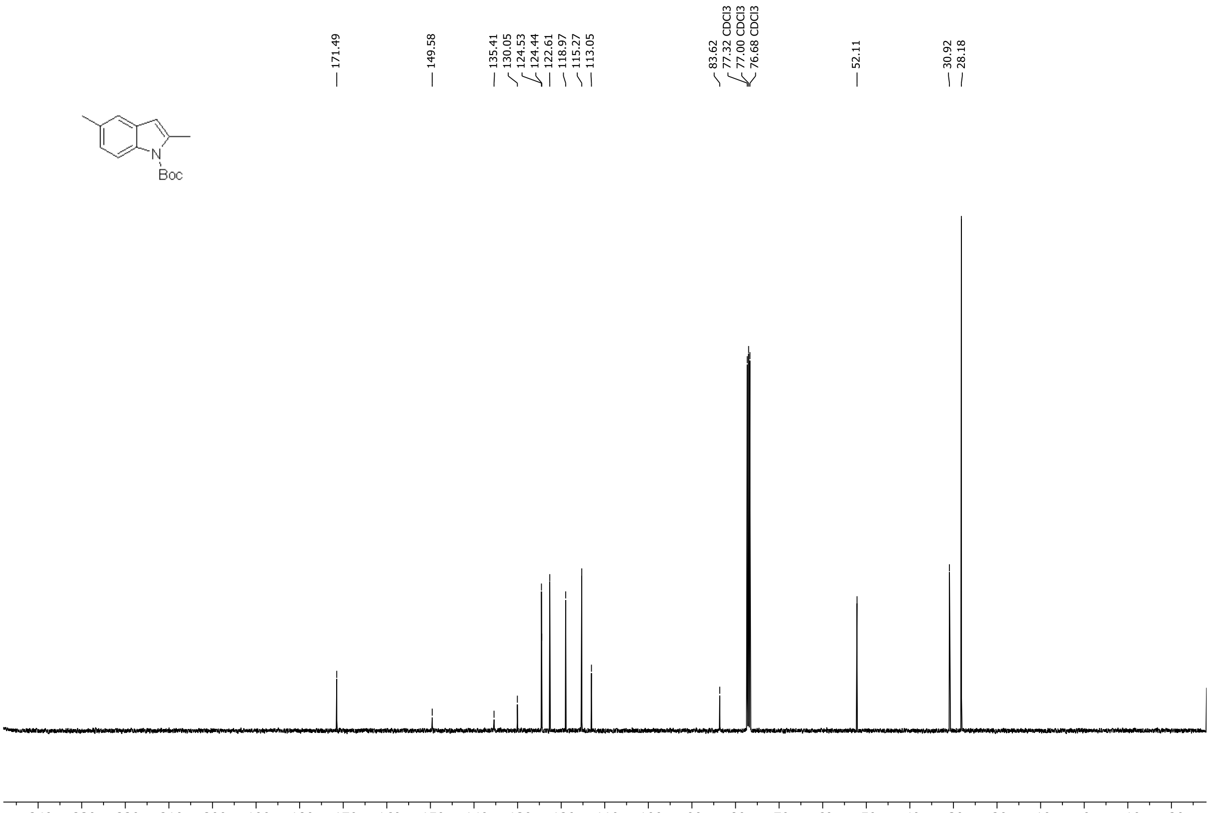

^{1}H NMR (400 MHz, Chloroform-*d*) of compound **4d**

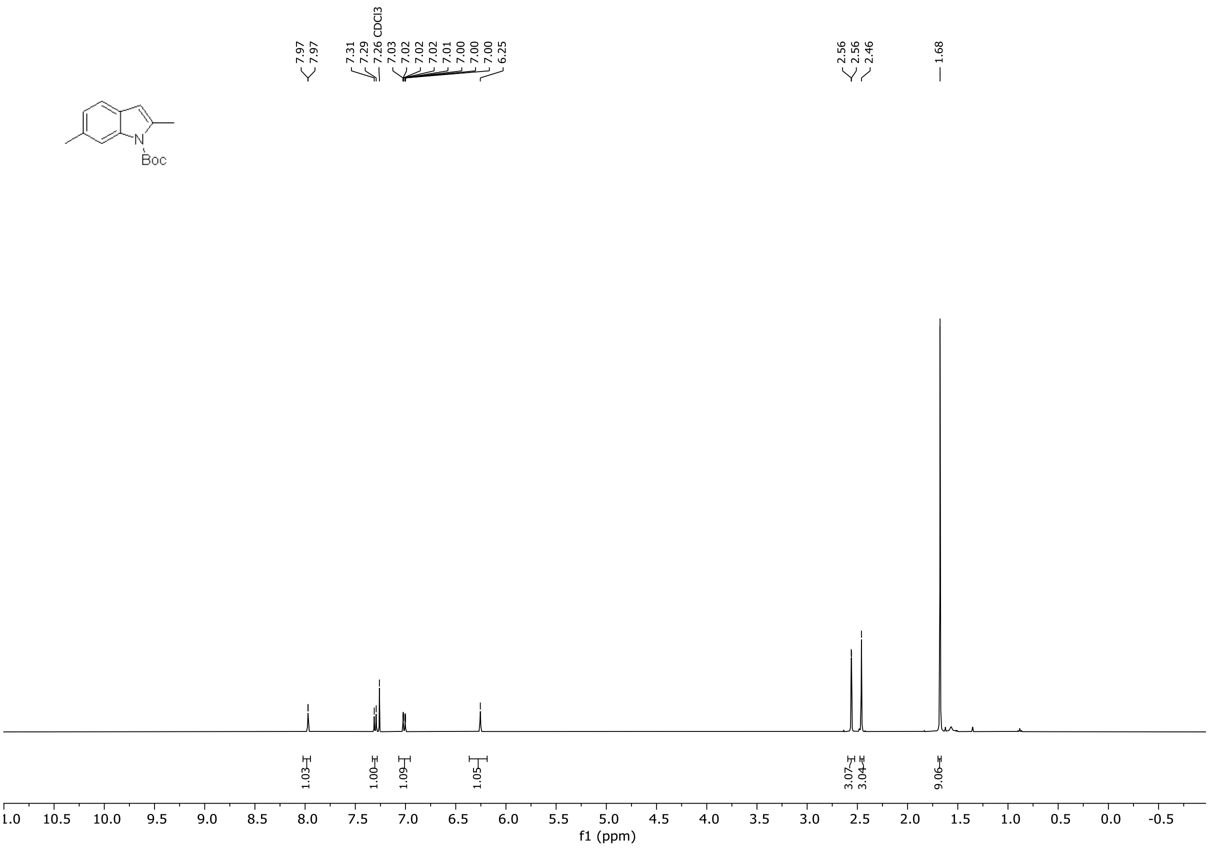

^{13}C NMR (101 MHz, Chloroform-*d*) of compound **4d**

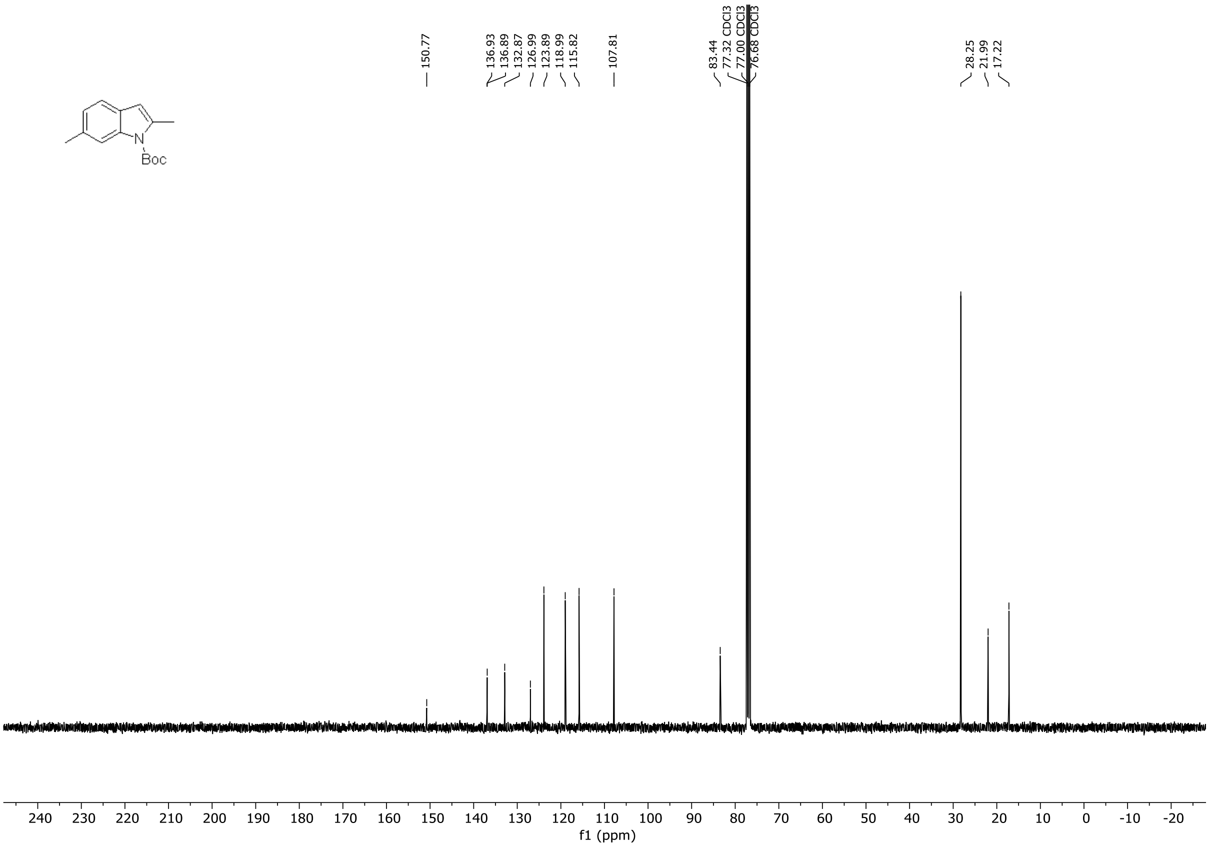

^{1}H NMR (400 MHz, Chloroform-*d*) of compound **4e**

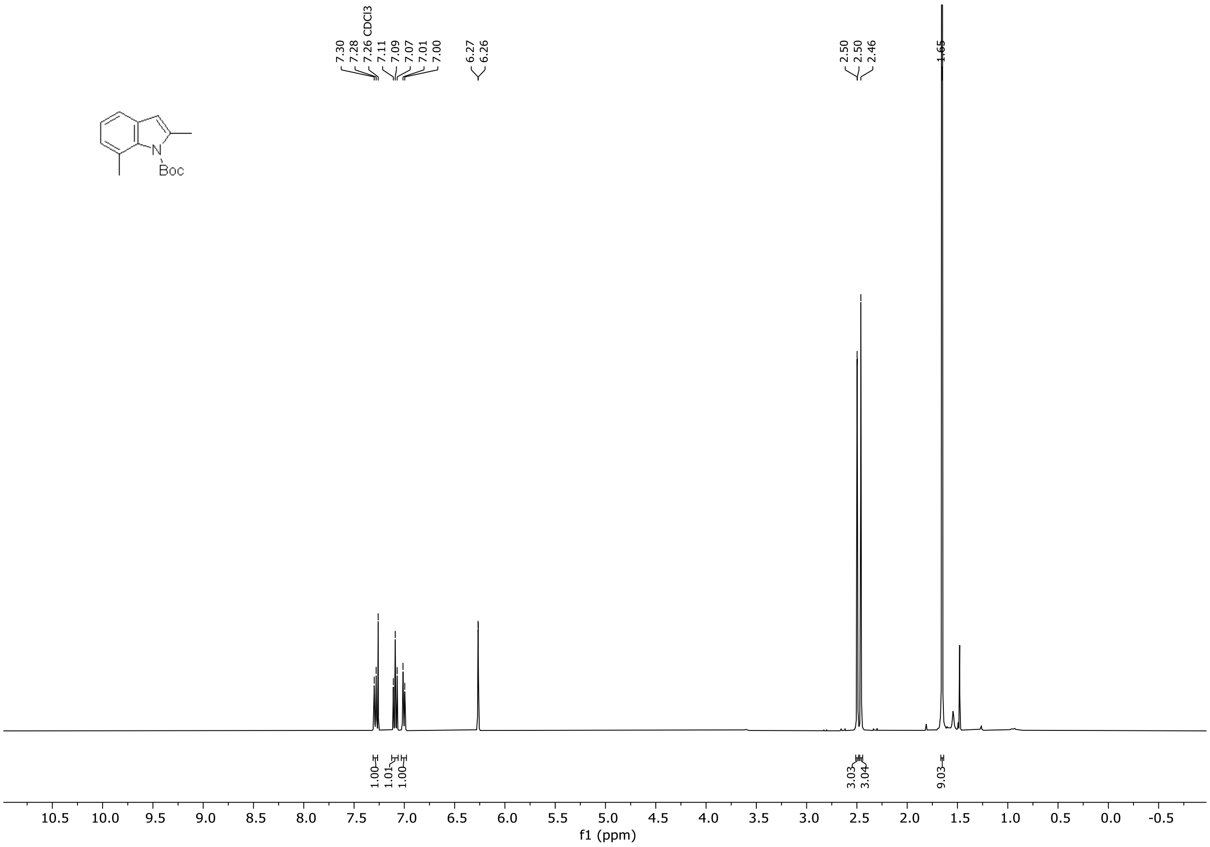

^{13}C NMR (101 MHz, Chloroform-*d*) of compound **4e**

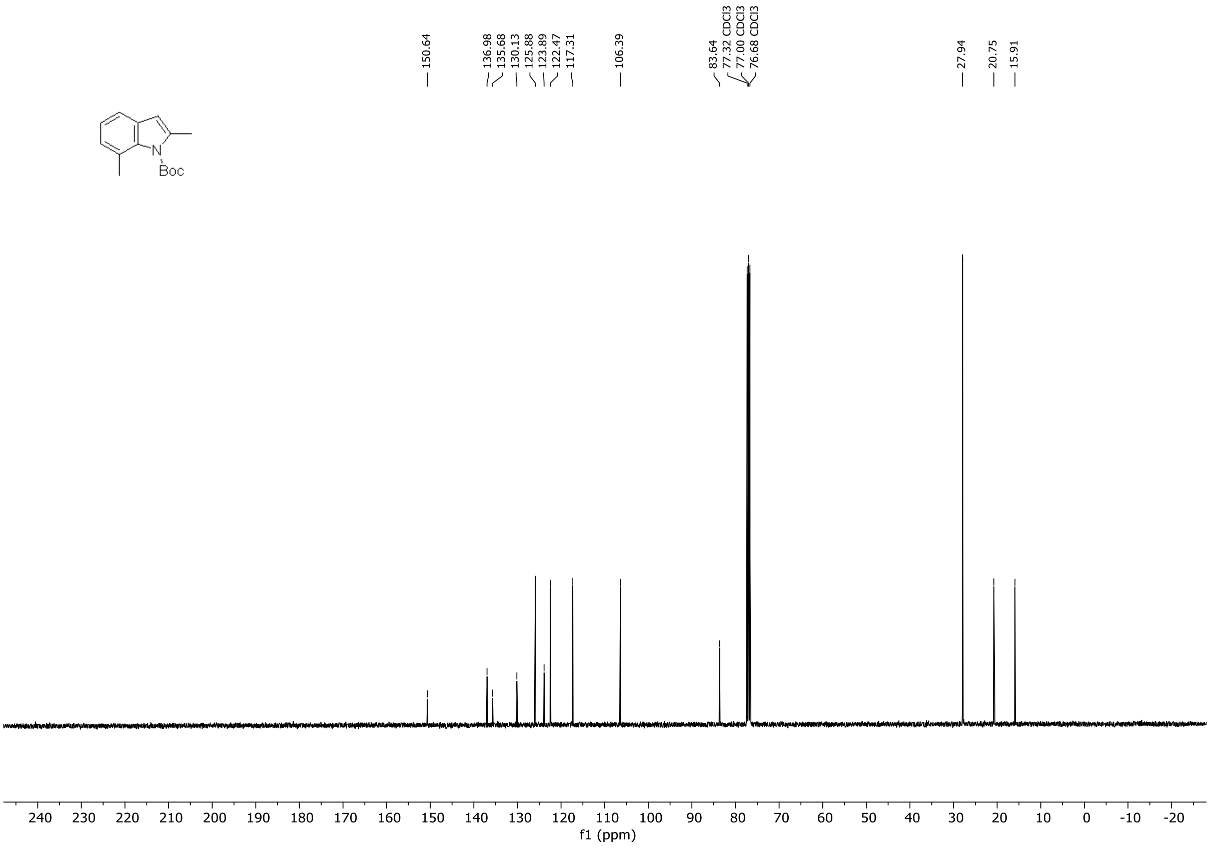

^{1}H NMR (400 MHz, Chloroform-*d*) of compound **4f**

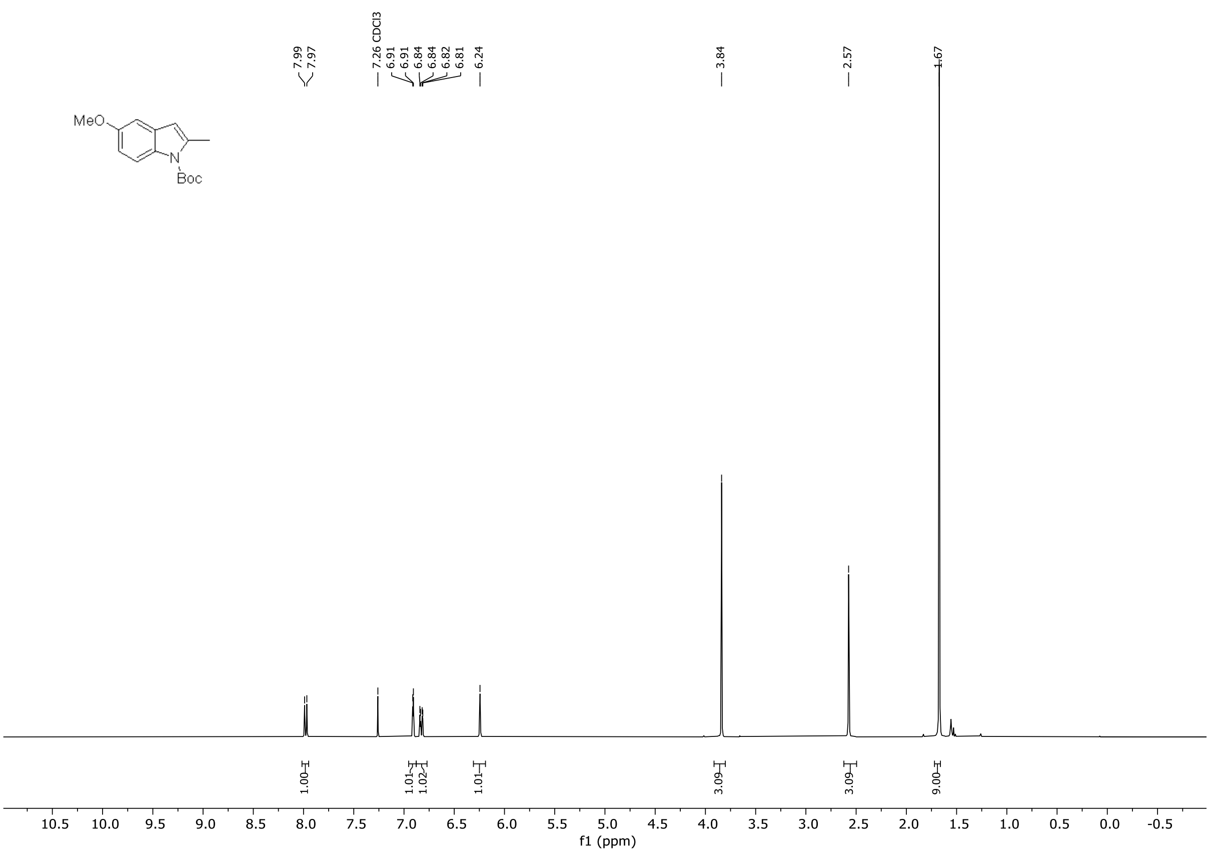

^{13}C NMR (101 MHz, Chloroform-*d*) of compound **4f**

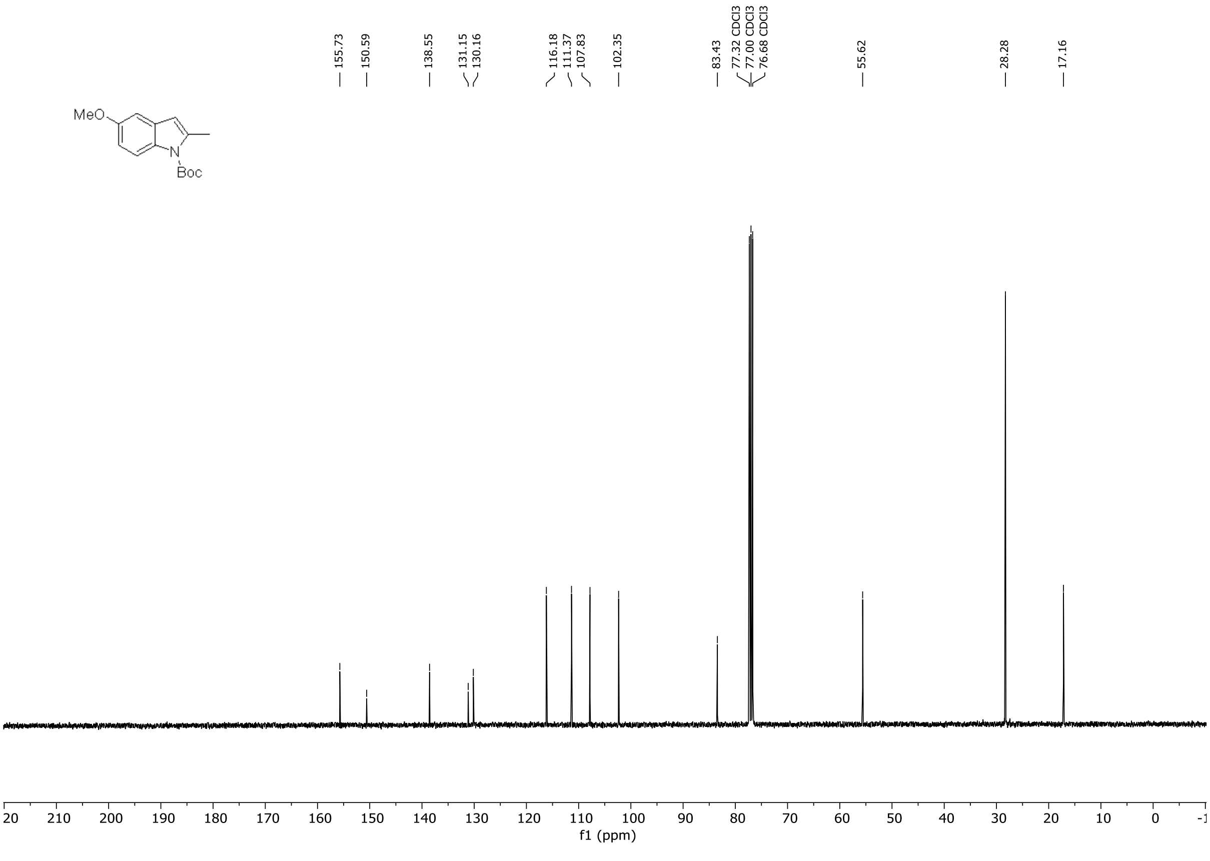

^{1}H NMR (400 MHz, Chloroform-*d*) of compound **4g**

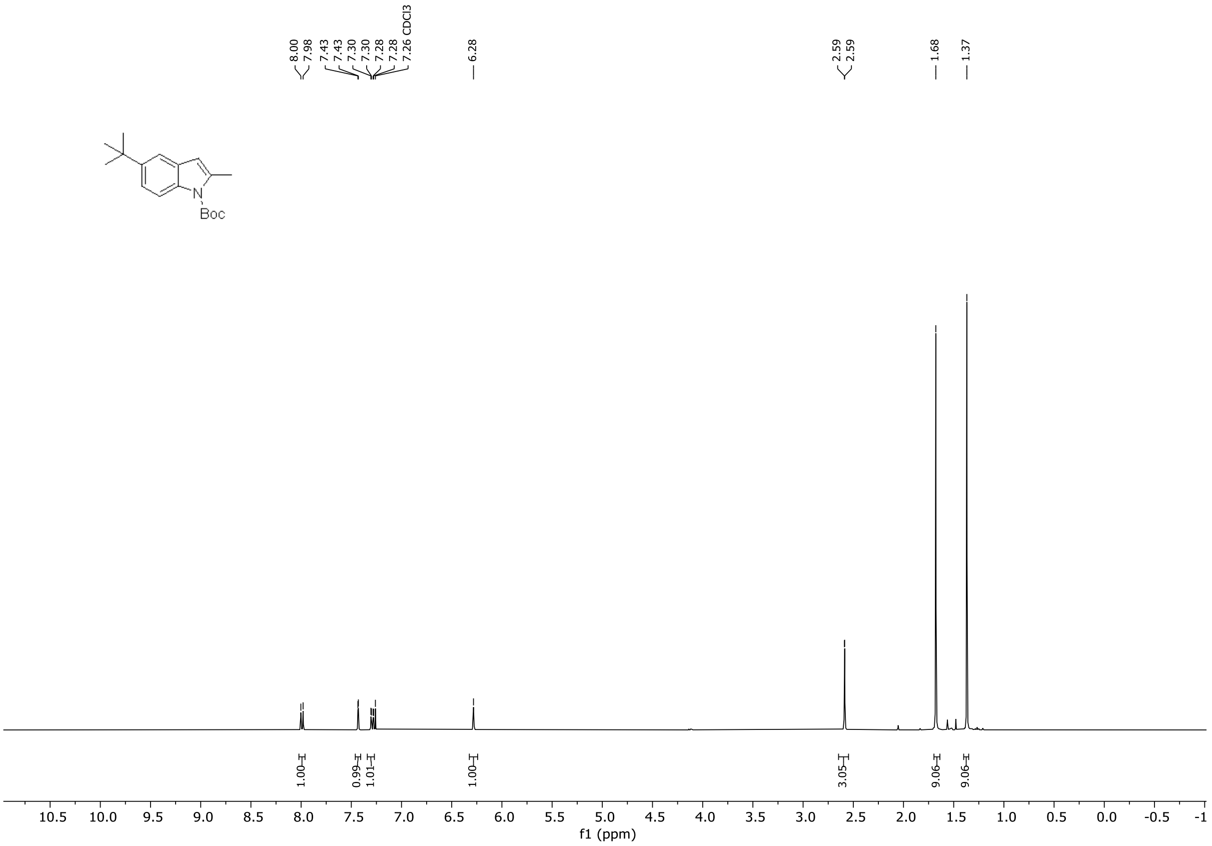

^{13}C NMR (101 MHz, Chloroform-*d*) of compound **4g**

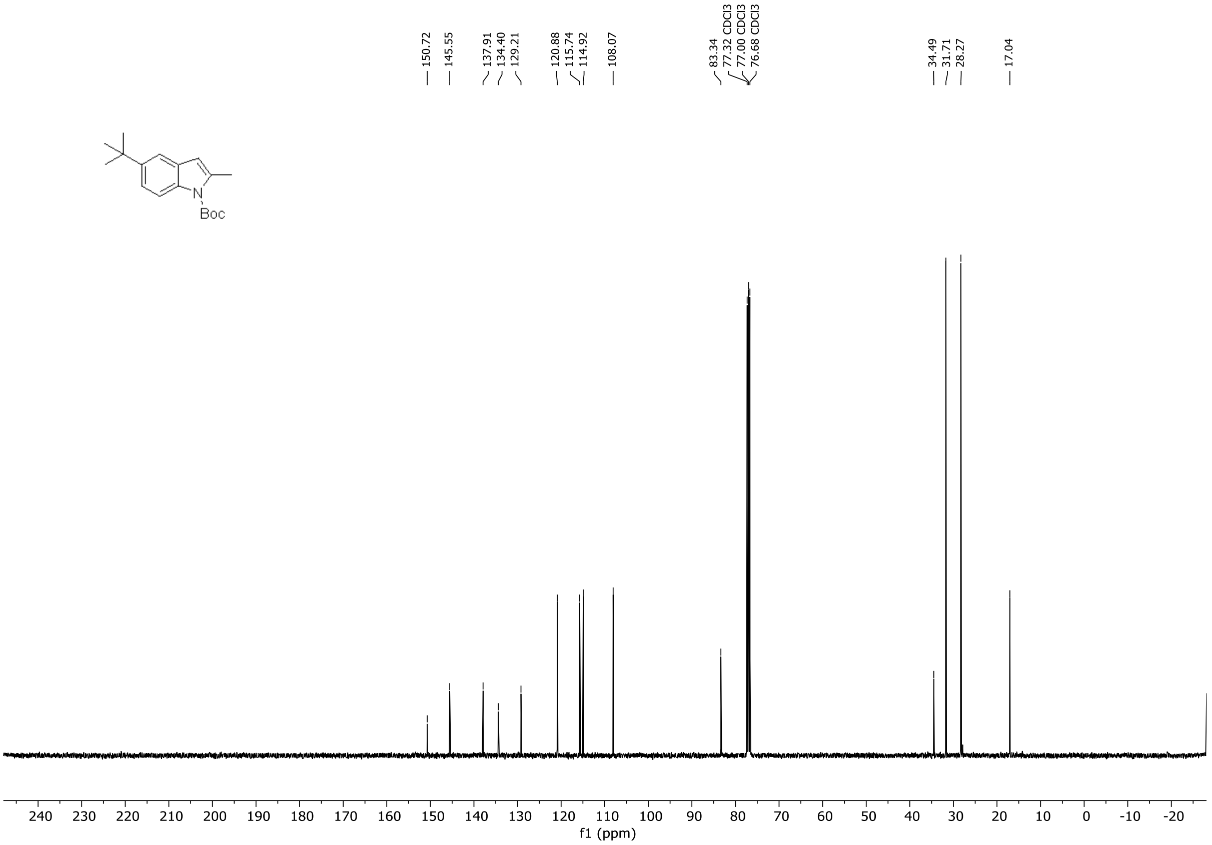

^{1}H NMR (400 MHz, Chloroform-*d*) of compound **4h**

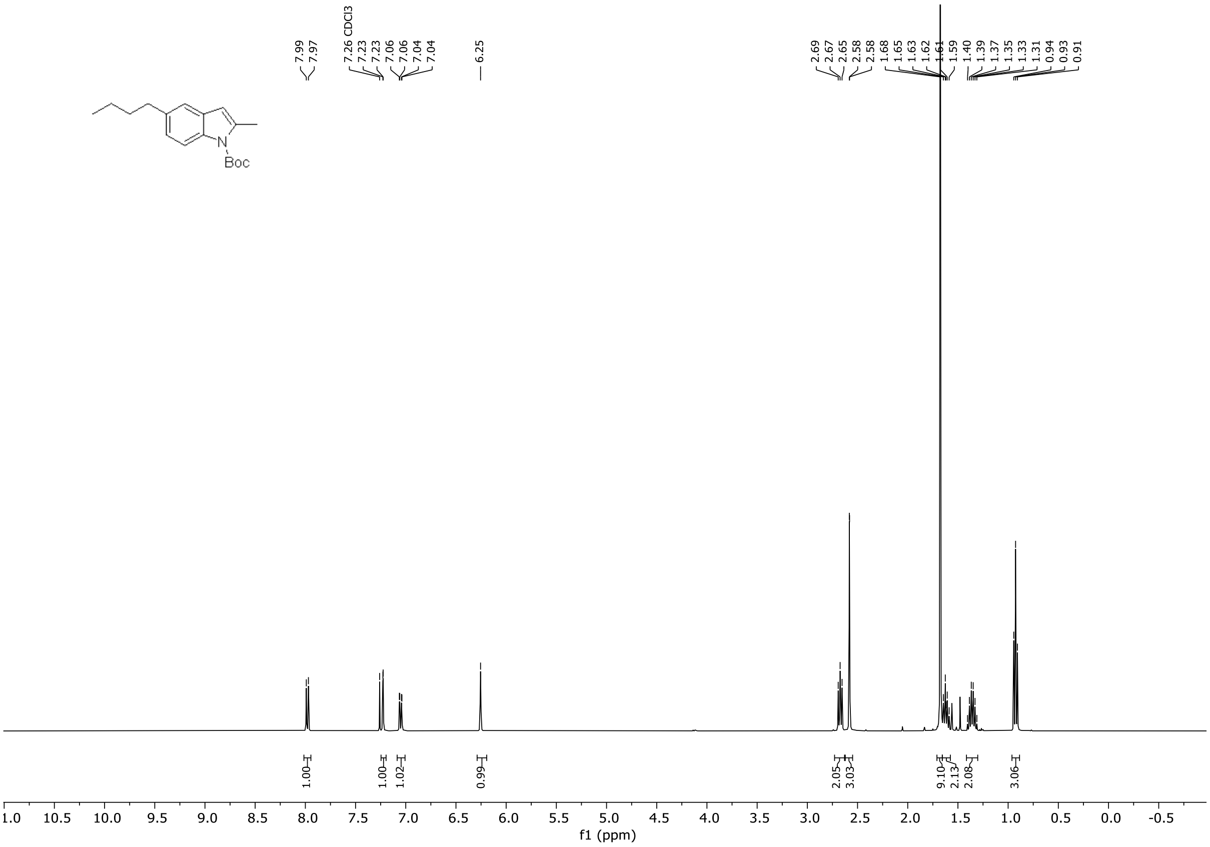

^{13}C NMR (101 MHz, Chloroform-*d*) of compound **4h**

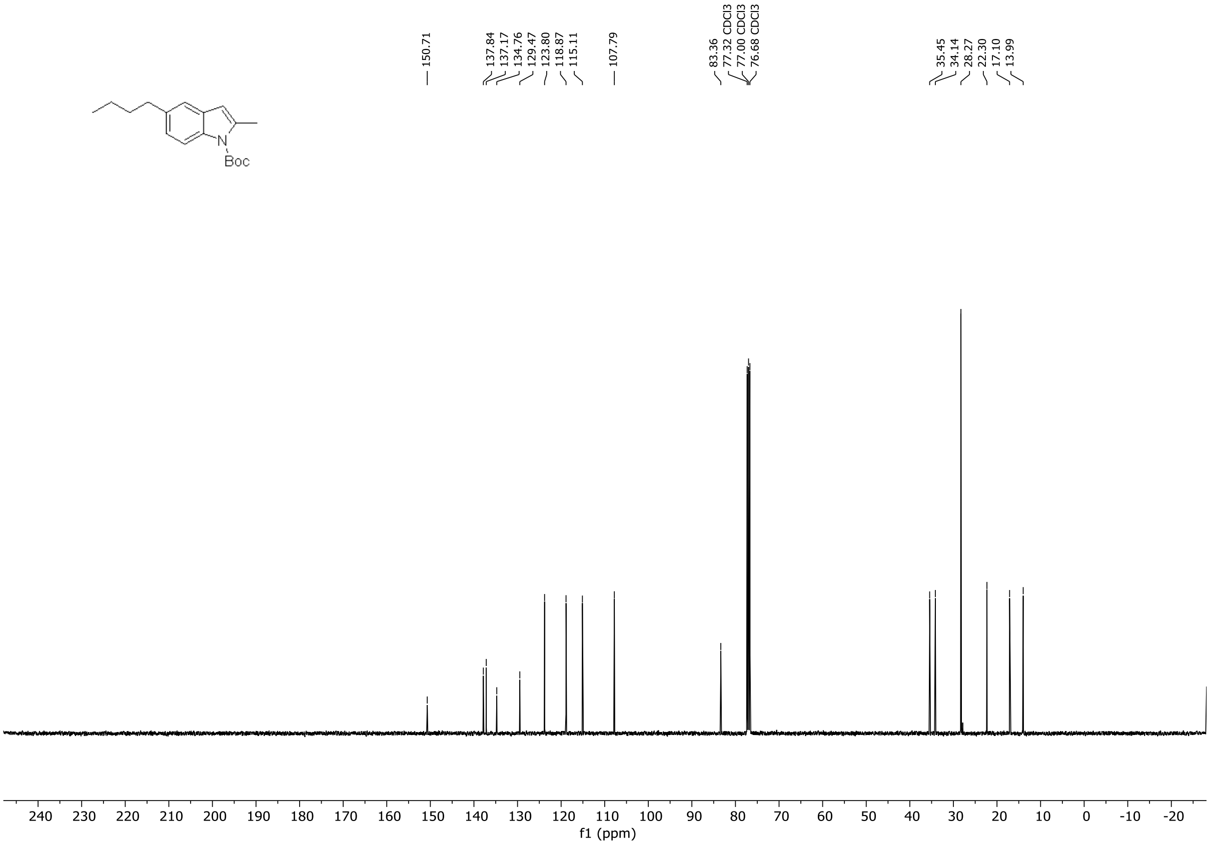

^{1}H NMR (400 MHz, Chloroform-*d*) of compound **4i**

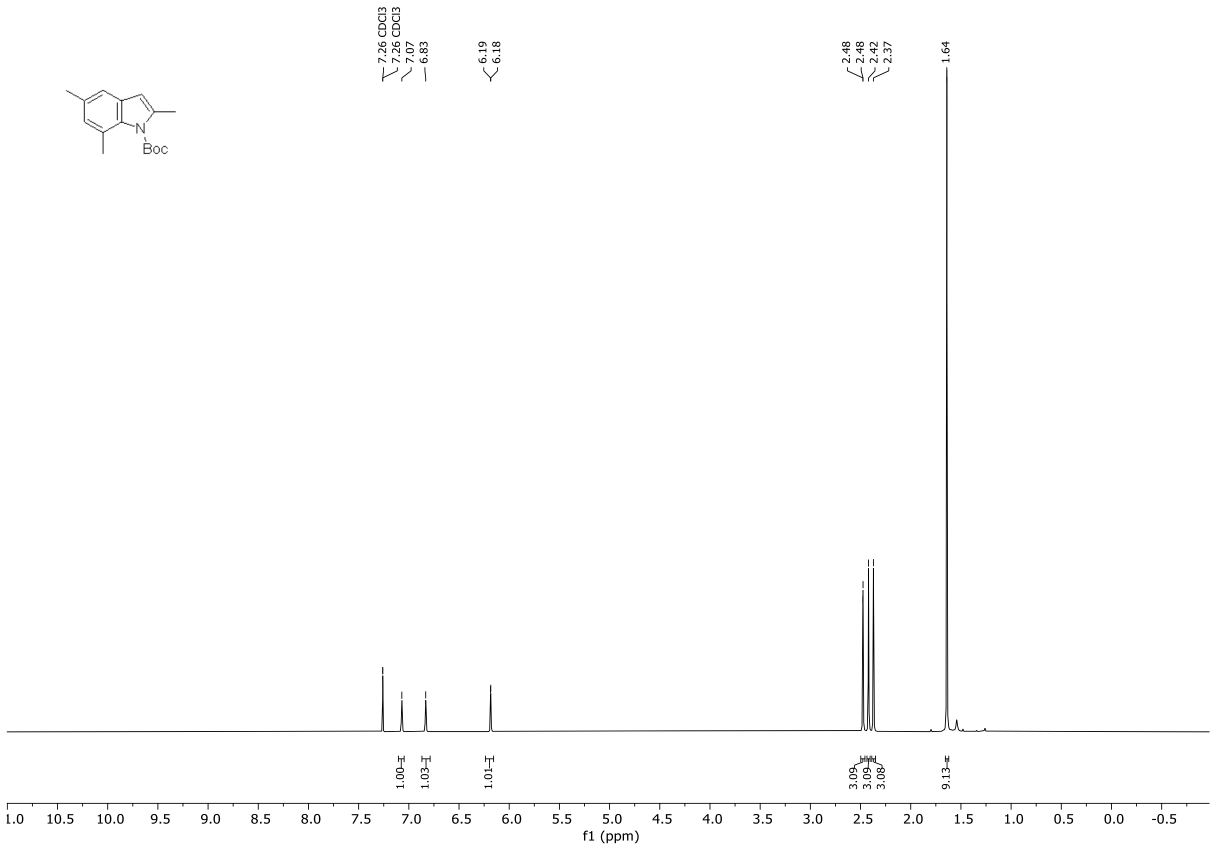

^{13}C NMR (101 MHz, Chloroform-*d*) of compound **4i**

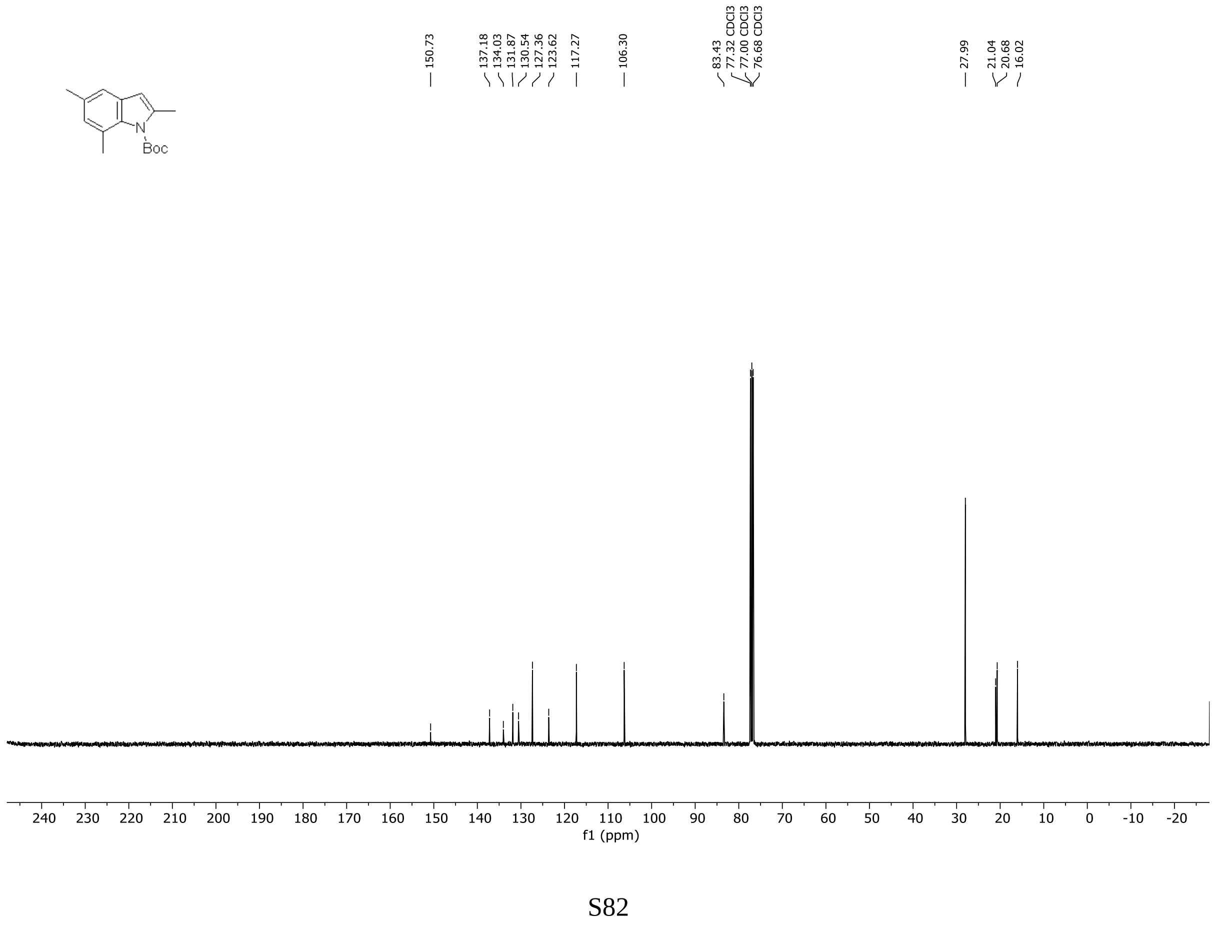

^{1}H NMR (400 MHz, Chloroform-*d*) of compound **4j**

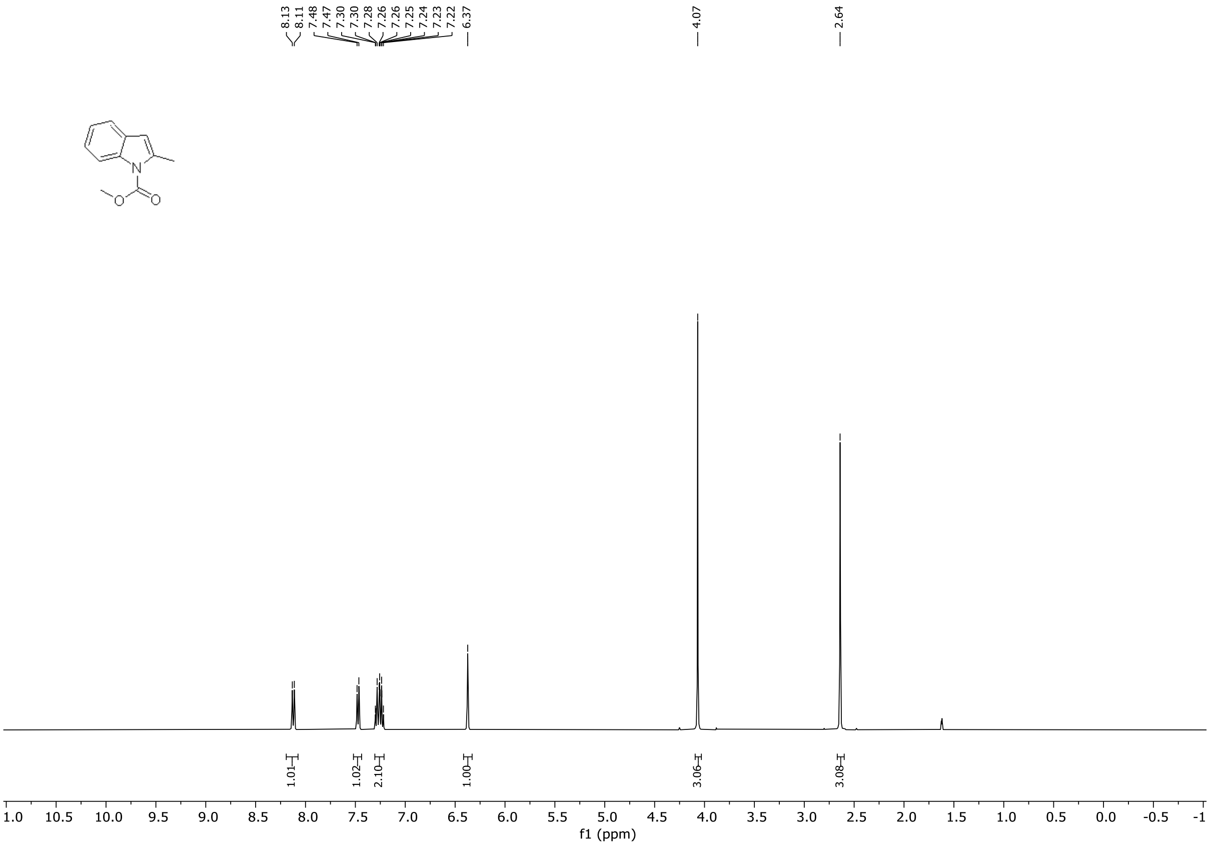

^{13}C NMR (101 MHz, Chloroform-*d*) of compound **4j**

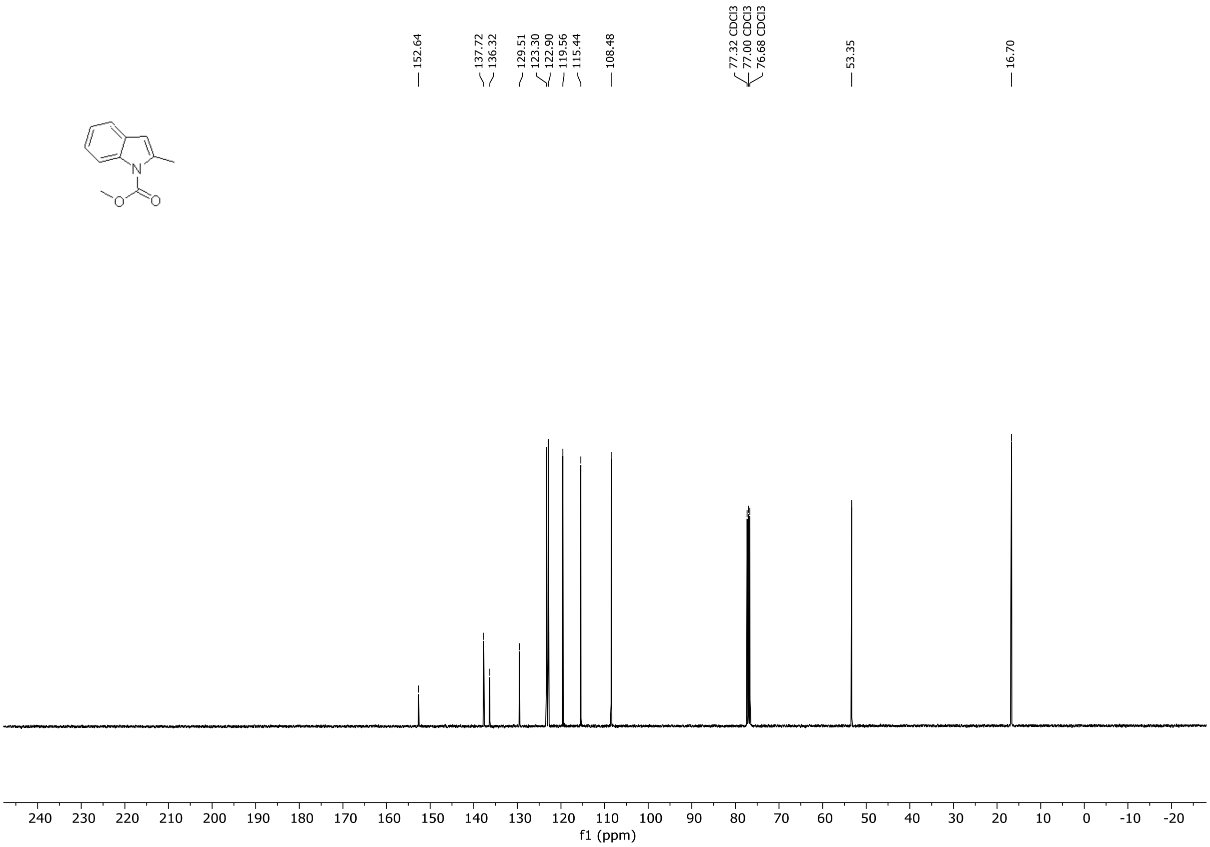

^{1}H NMR (400 MHz, Chloroform-*d*) of compound **4k**

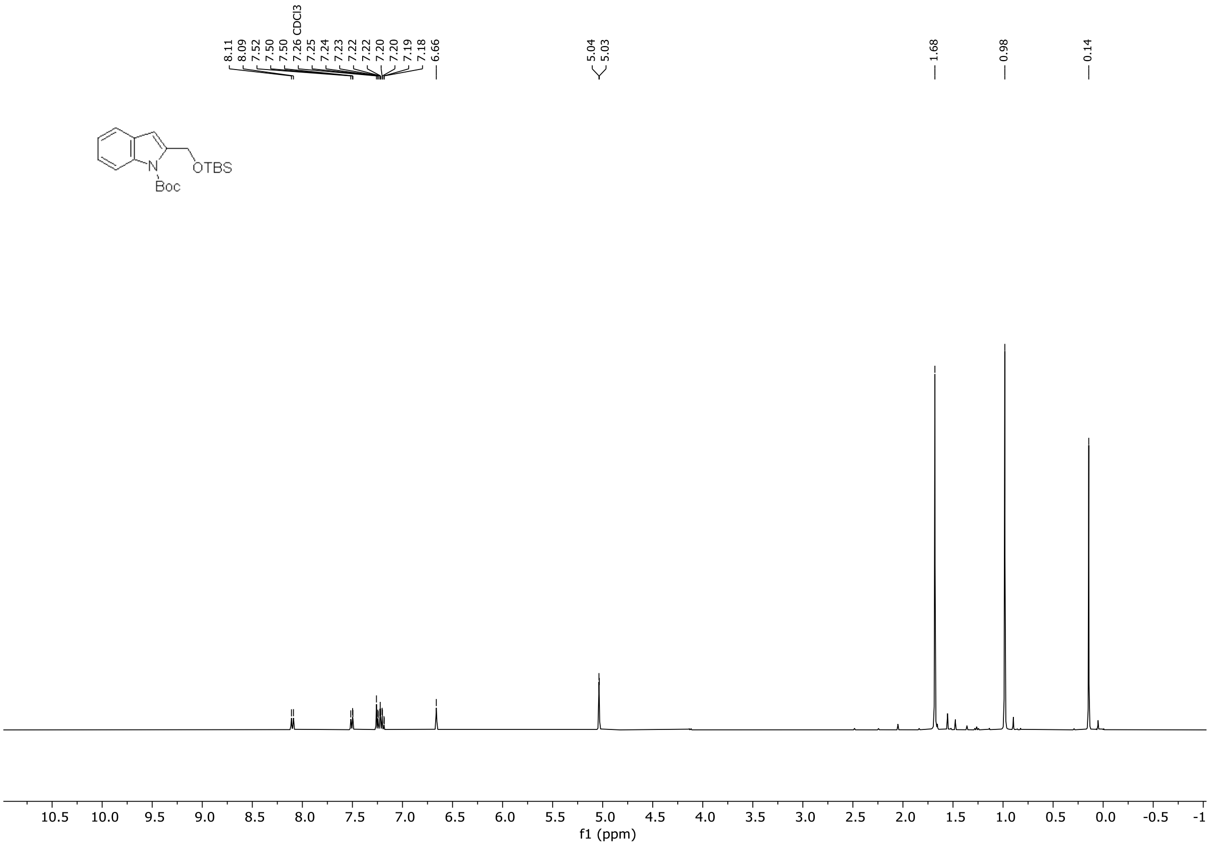

^{13}C NMR (101 MHz, Chloroform-*d*) of compound **4k**

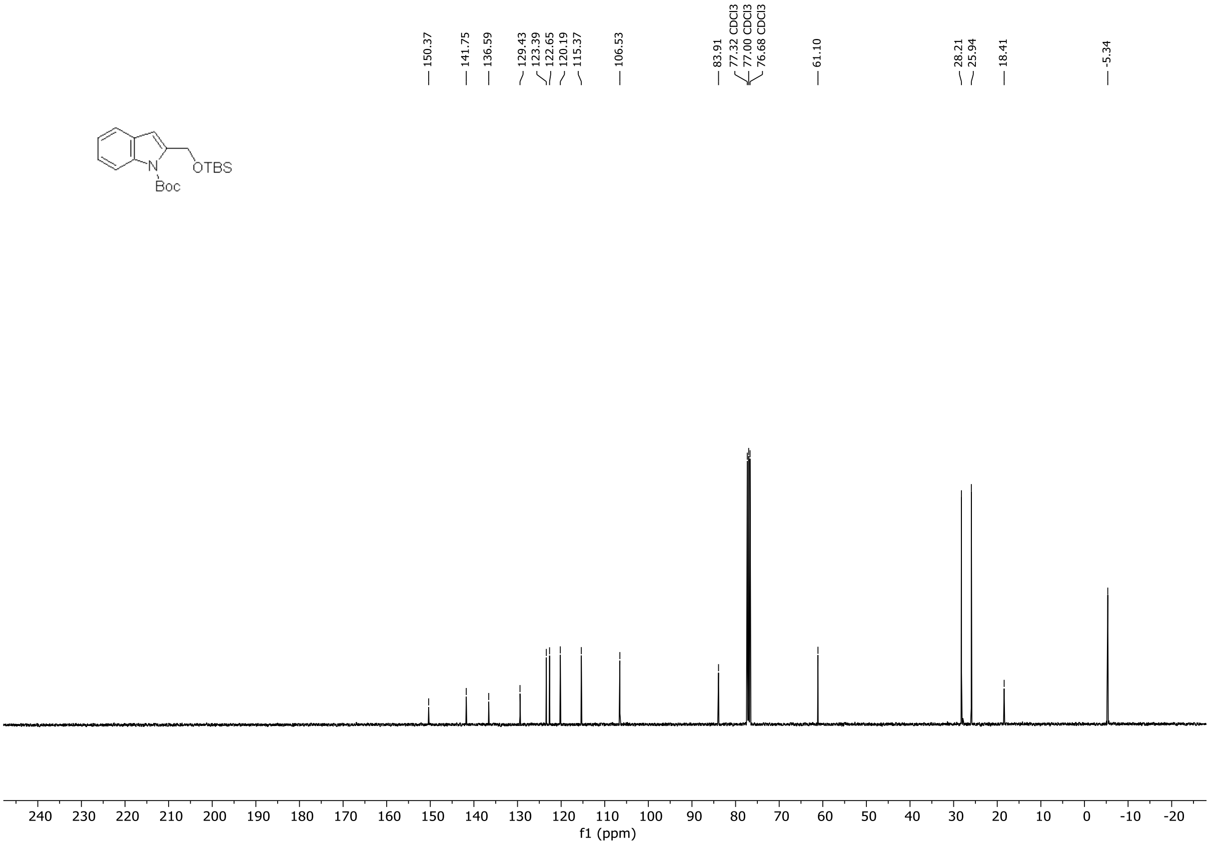

^{1}H NMR (400 MHz, Chloroform-*d*) of compound **2a** ^{13}C NMR (101 MHz, Chloroform-*d*) of compound **2a**

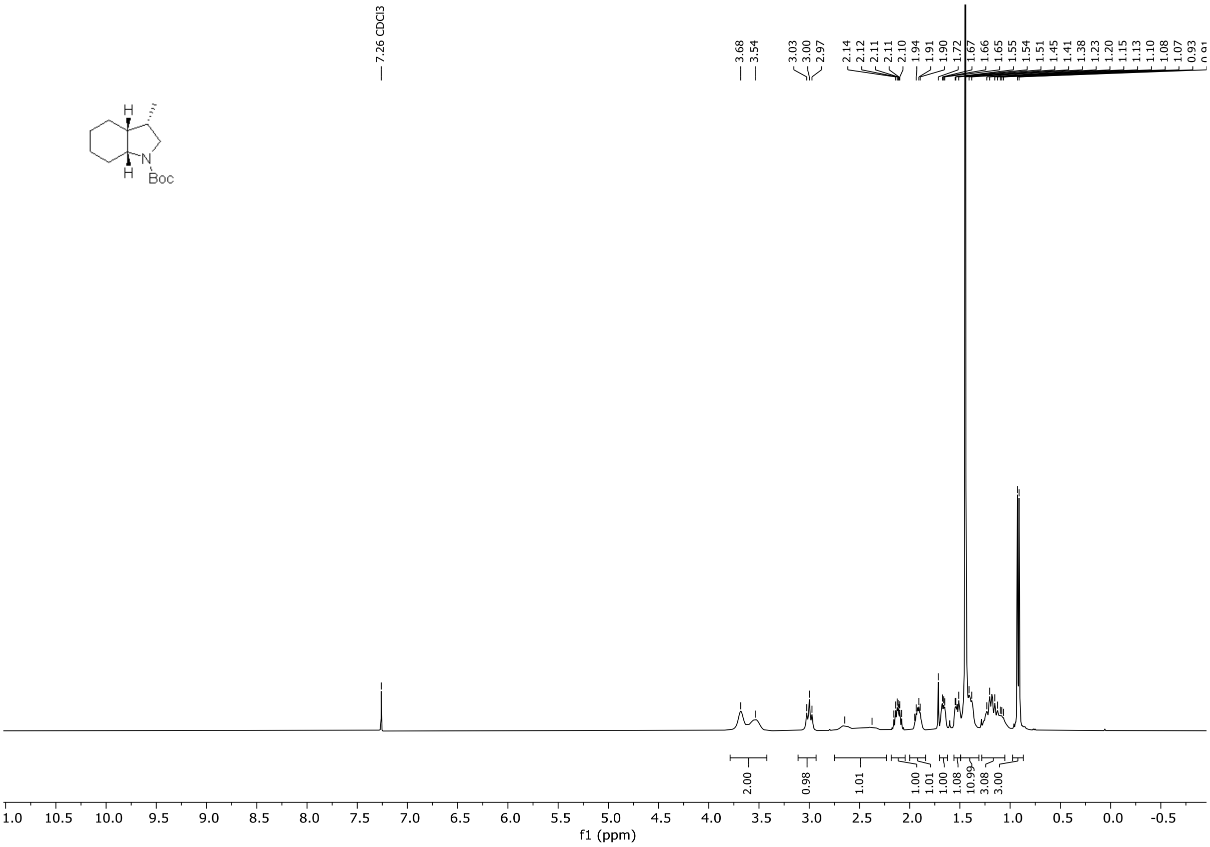

^{13}C NMR (101 MHz, Chloroform-*d*) of compound **2a**

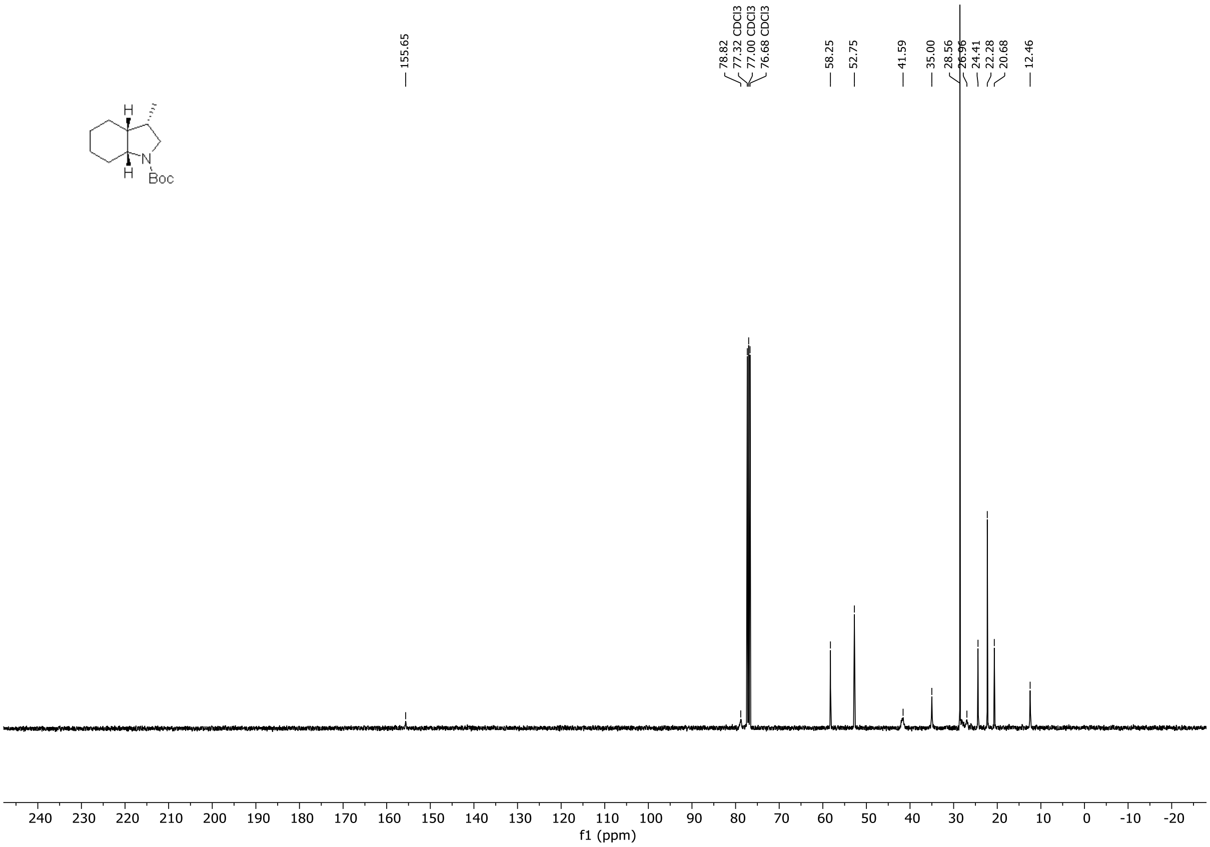

^{1}H NMR (400 MHz, Chloroform-*d*) of compound **2b**

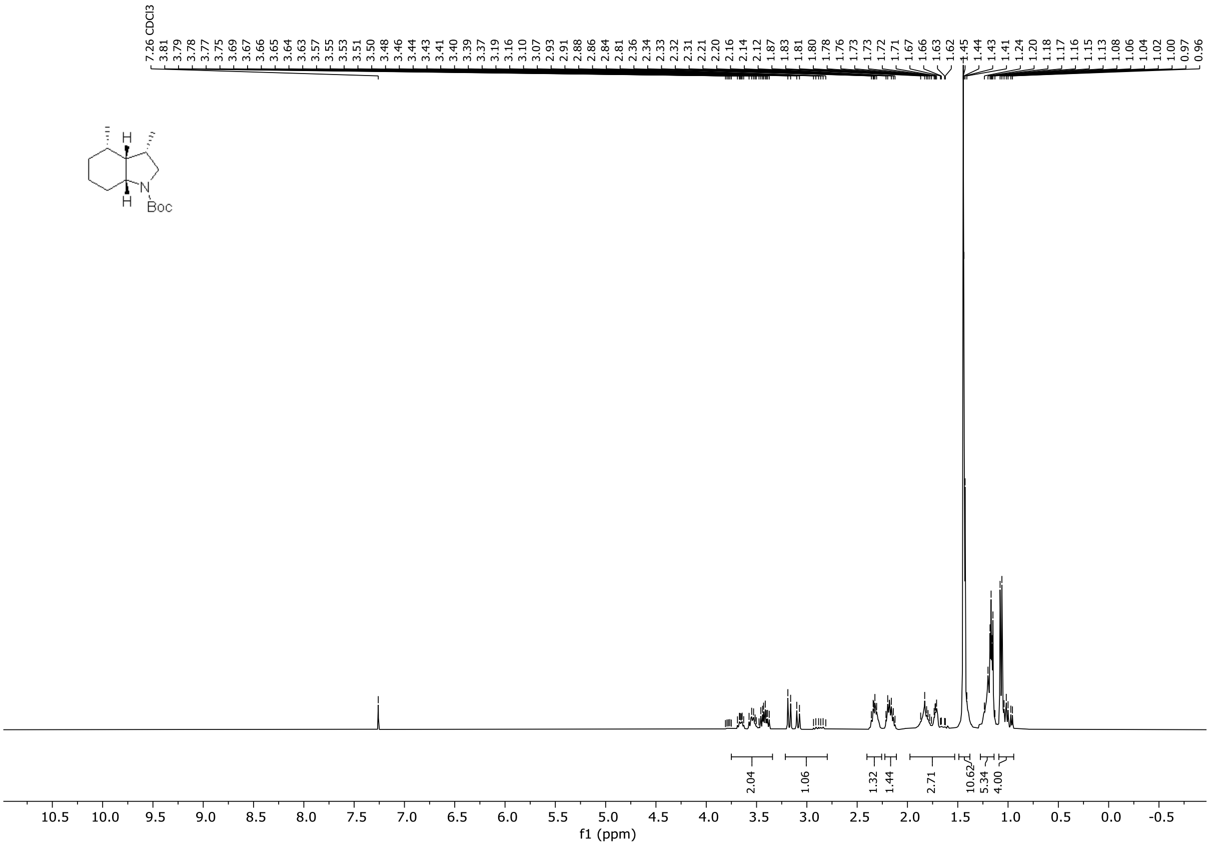

^{13}C NMR (101 MHz, Chloroform-*d*) of compound **2b**

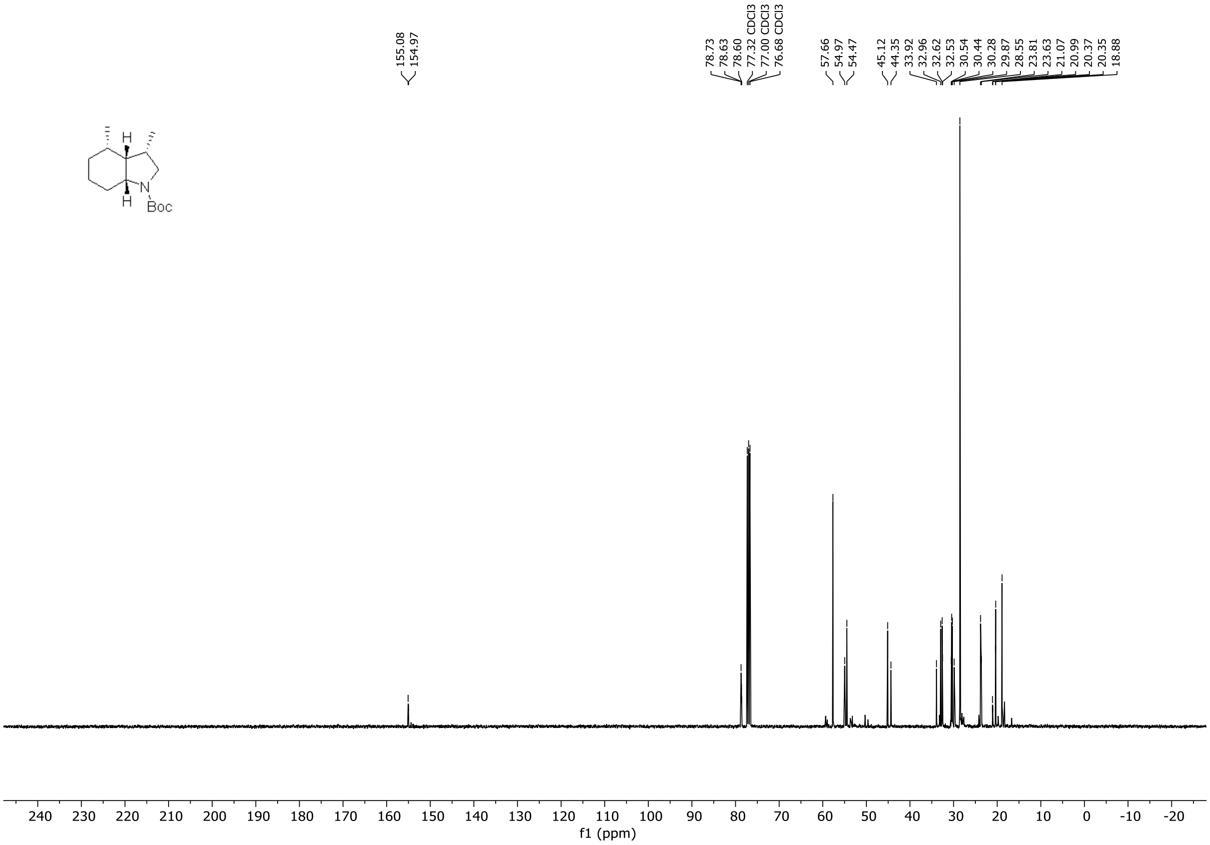

^{1}H NMR (400 MHz, Chloroform-*d*) of compound **2c**

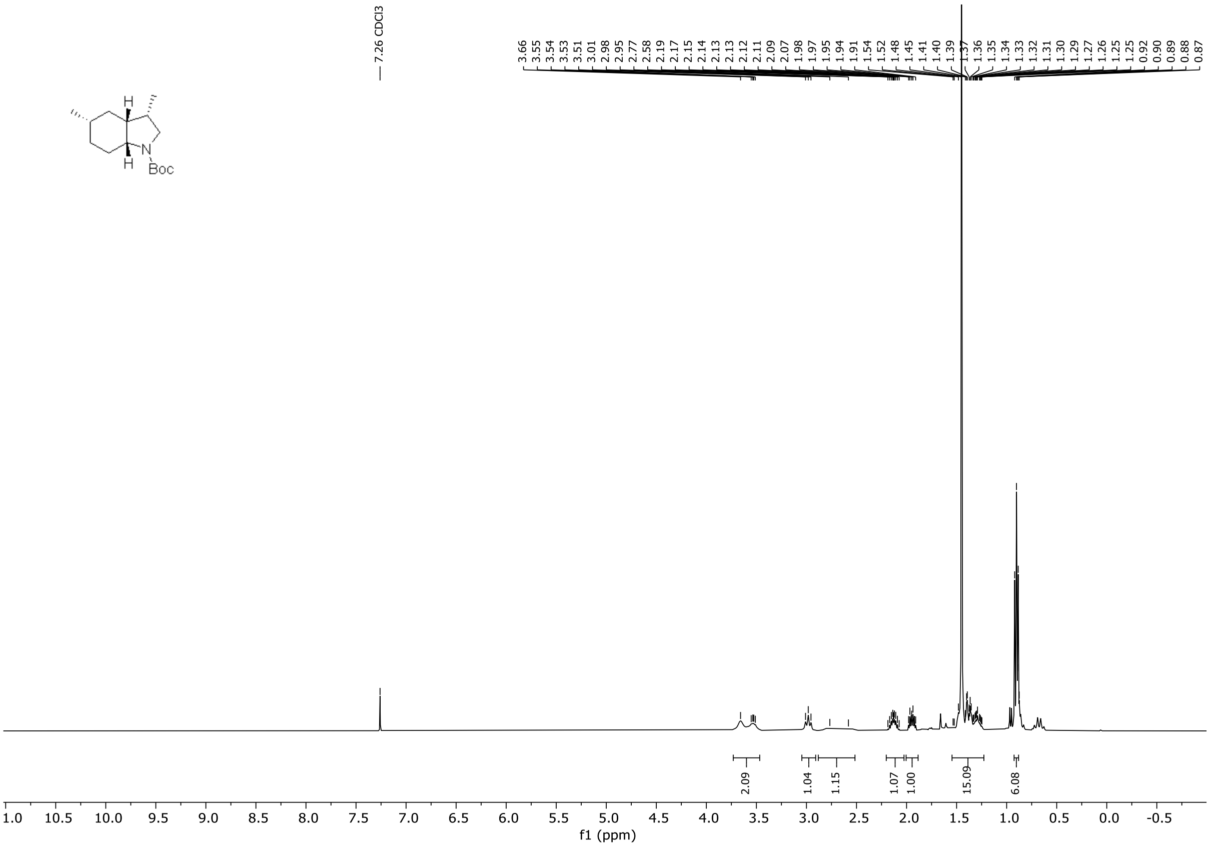

^{13}C NMR (101 MHz, Chloroform-*d*) of compound **2c**

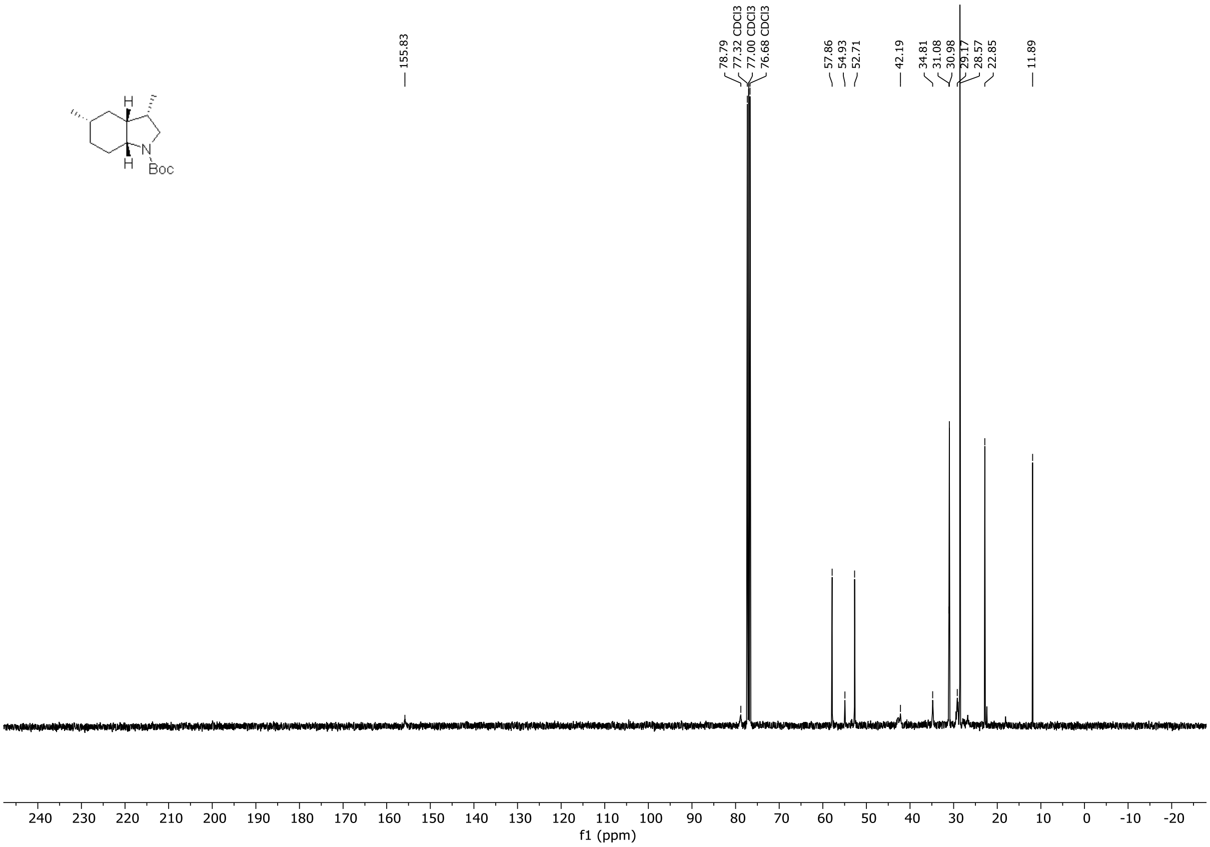

^{1}H NMR (400 MHz, Chloroform-*d*) of compound **2d**

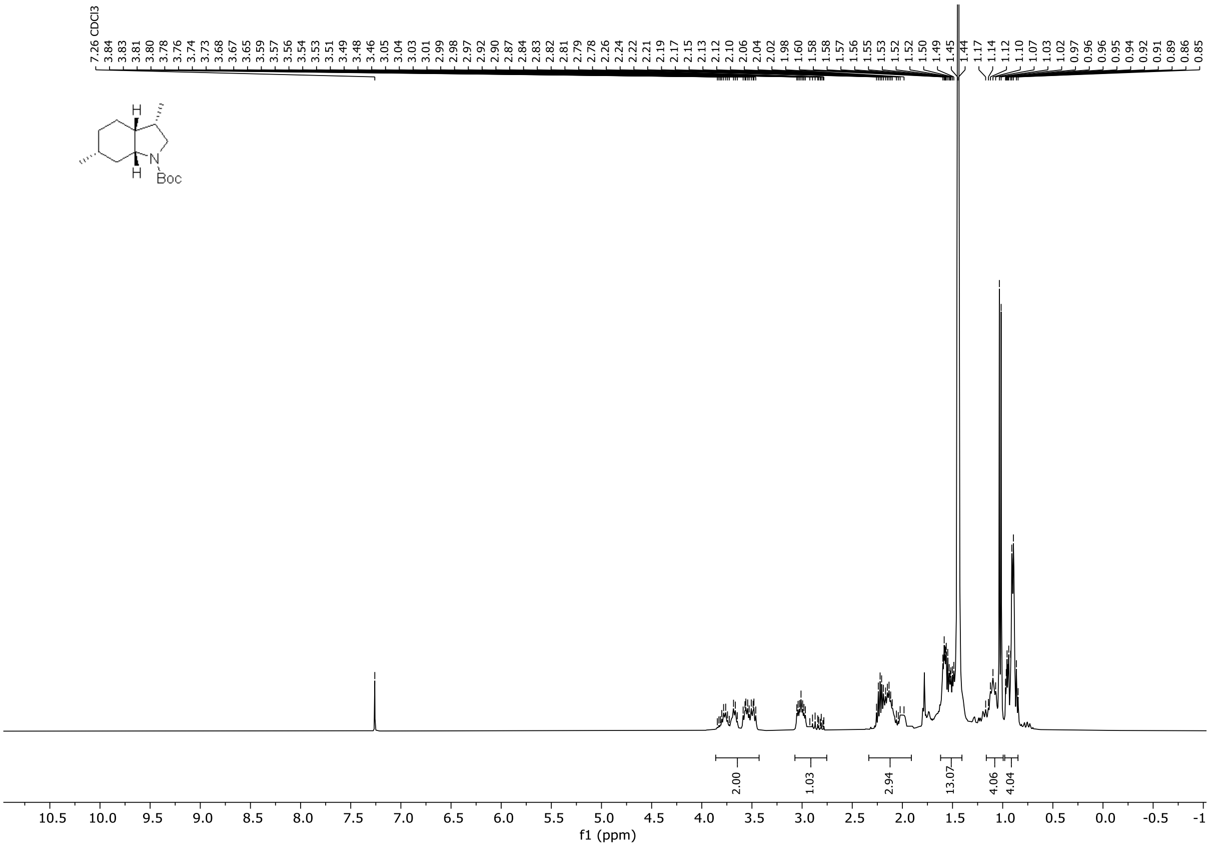

^{13}C NMR (101 MHz, Chloroform-*d*) of compound **2d**

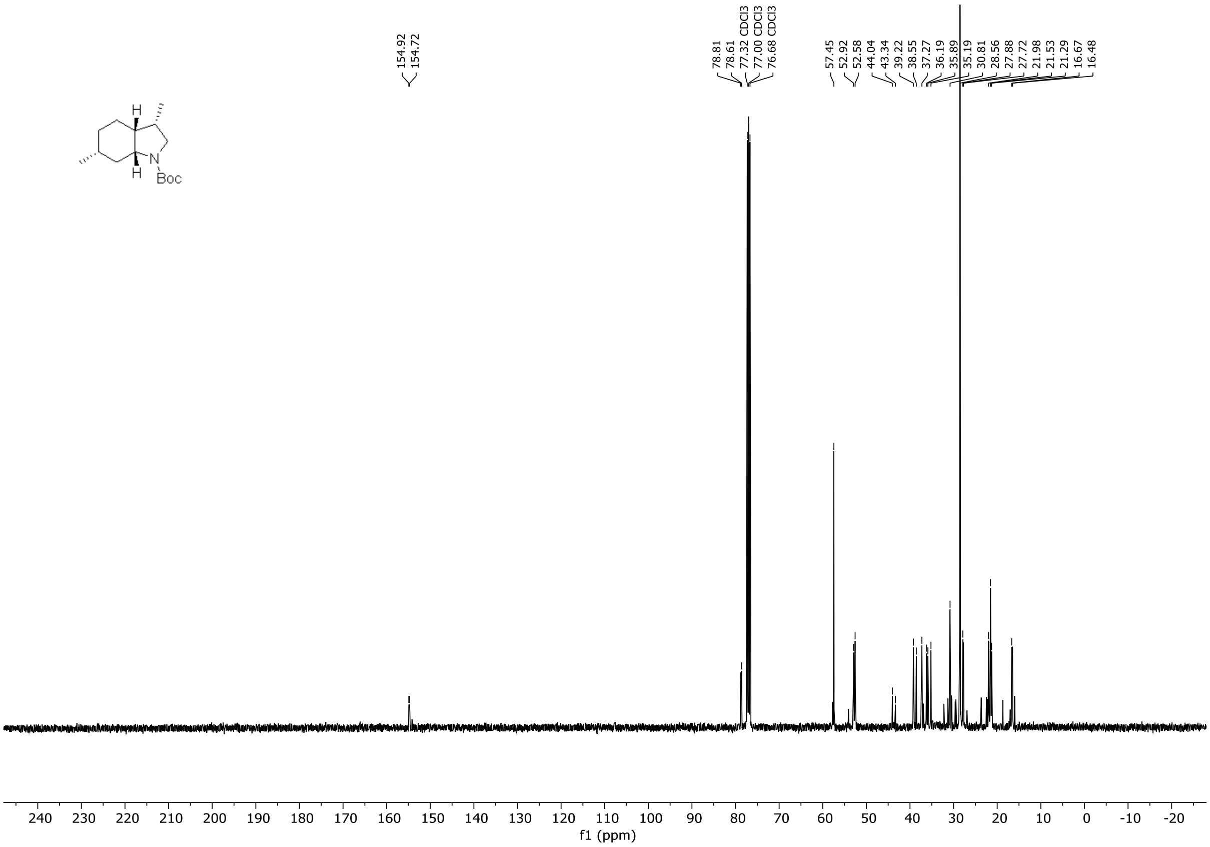

^{1}H NMR (400 MHz, Chloroform-*d*) of compound **2e**

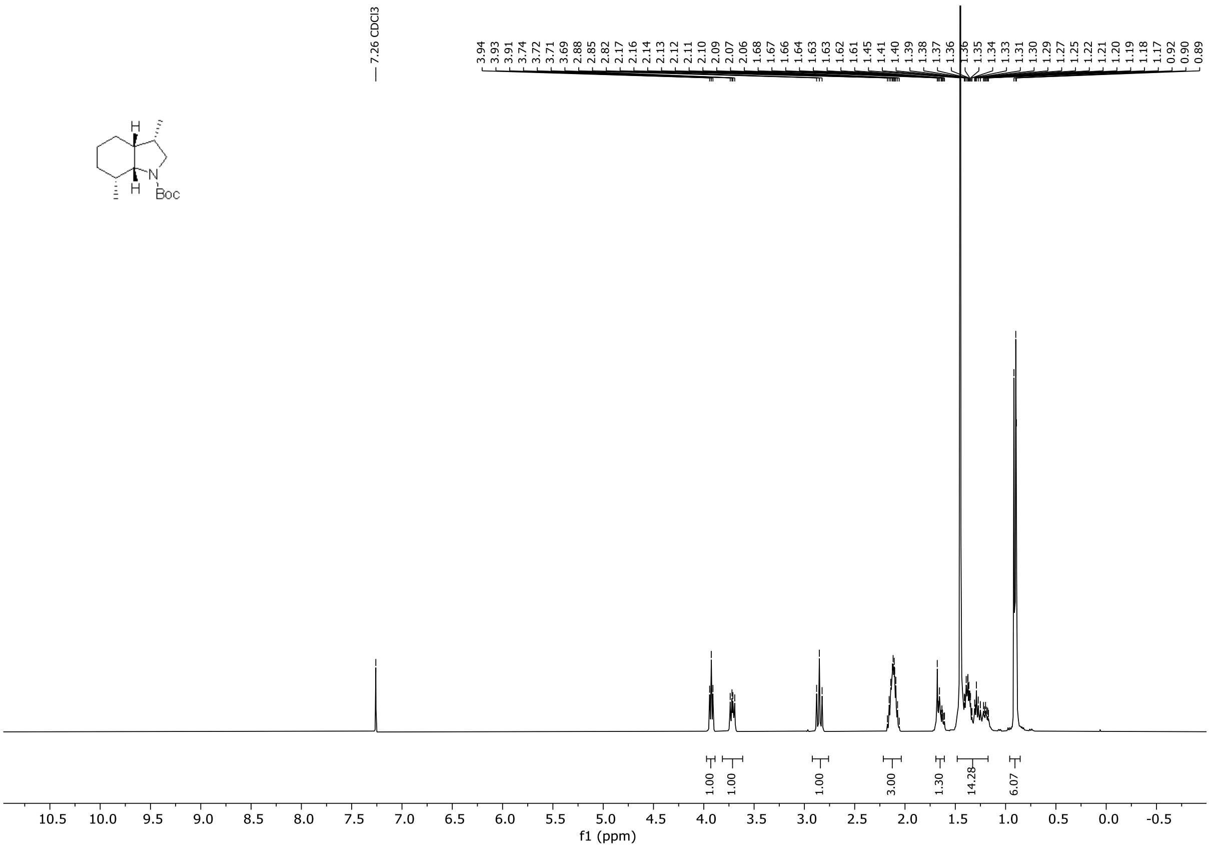

^{13}C NMR (101 MHz, Chloroform-*d*) of compound **2e**

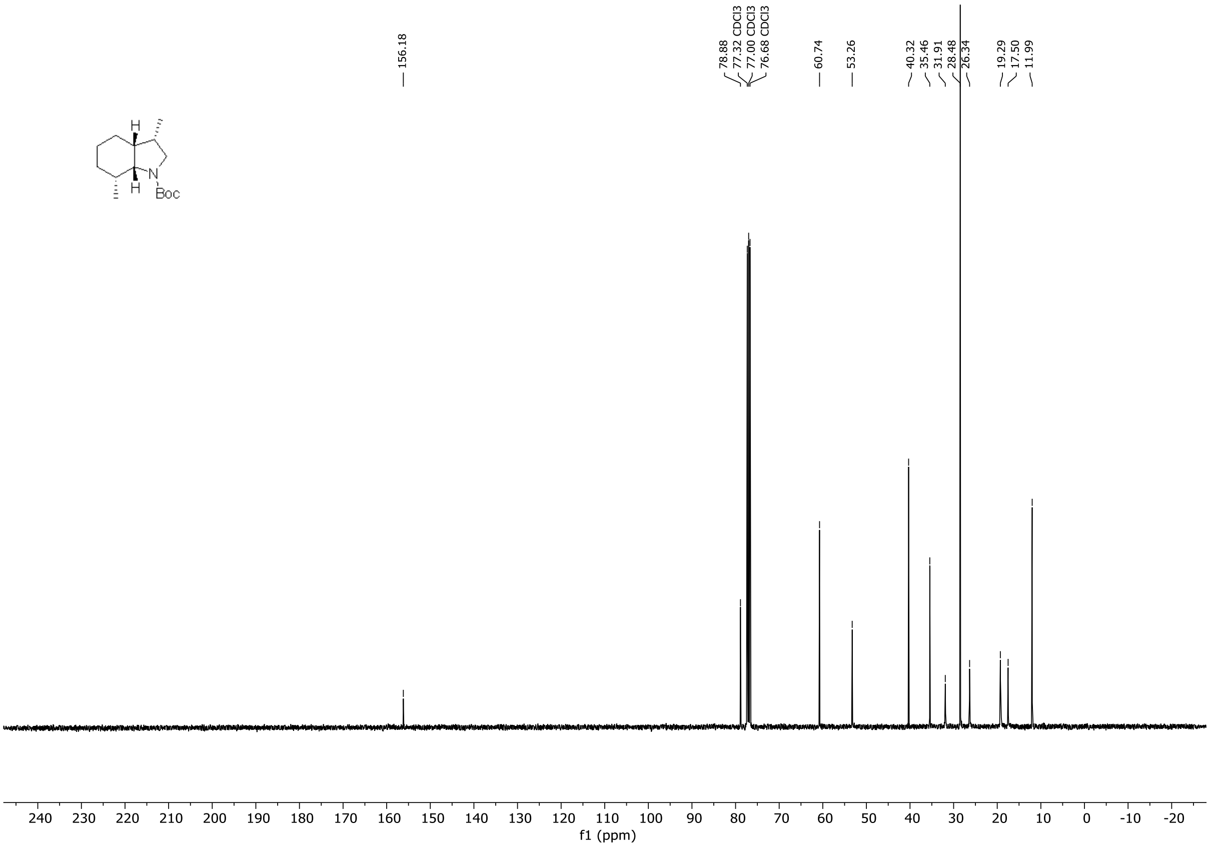

^{1}H NMR (400 MHz, Chloroform-*d*) of compound **2f**

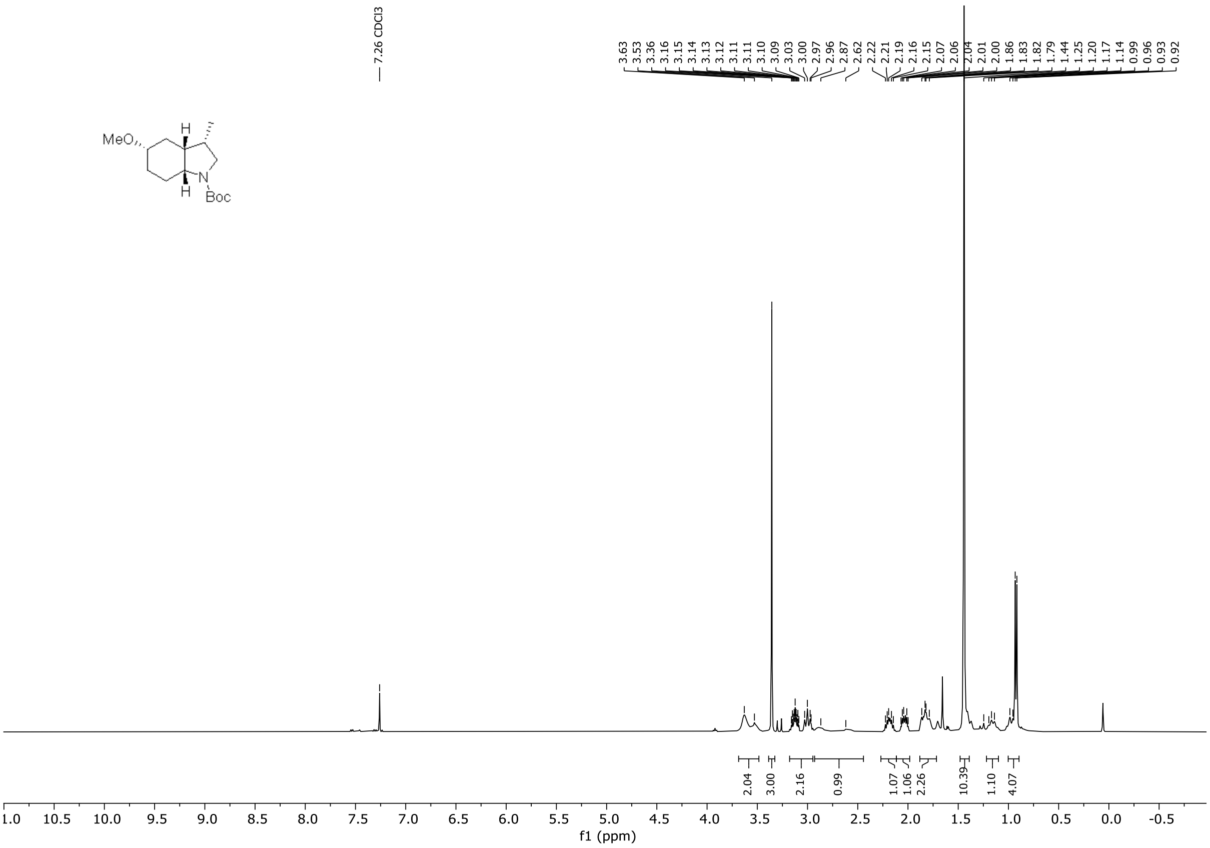

^{13}C NMR (101 MHz, Chloroform-*d*) of compound **2f**

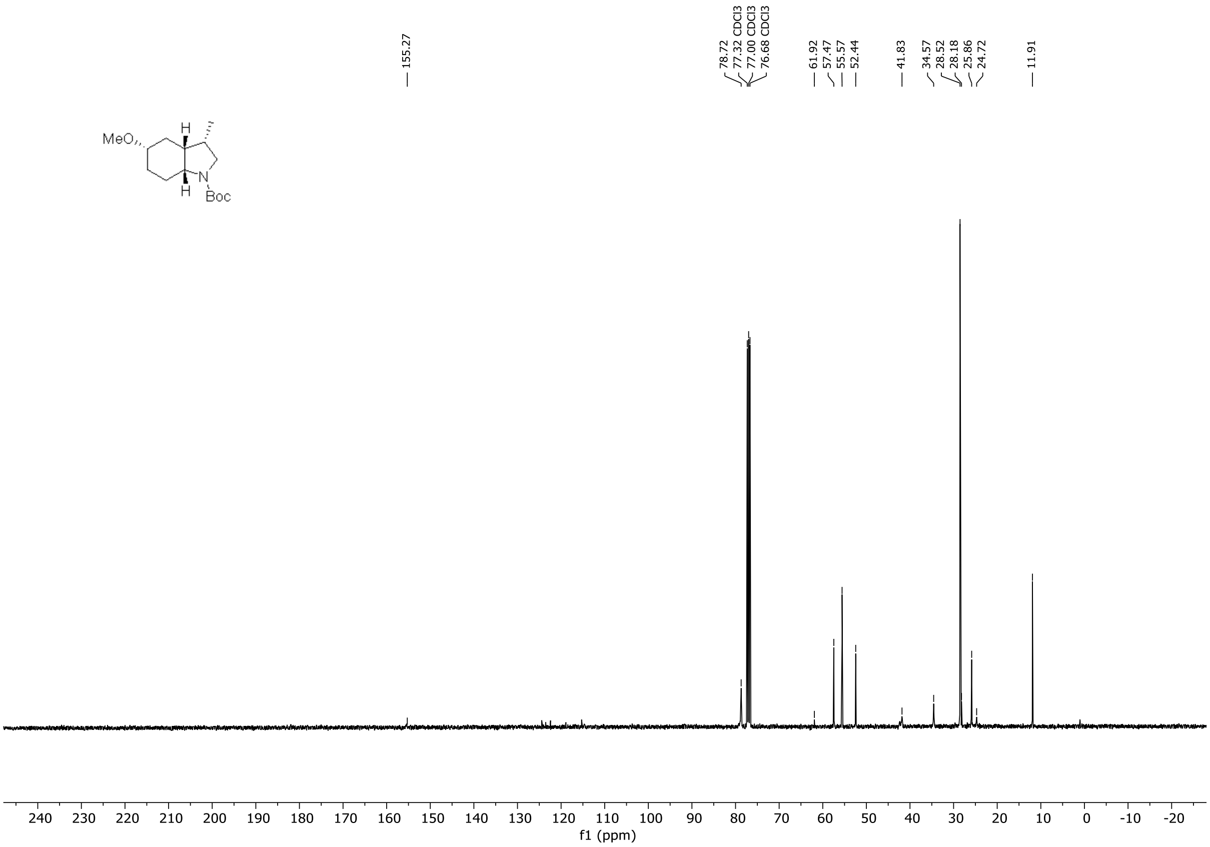

^{1}H NMR (400 MHz, Chloroform-*d*) of compound **2g**

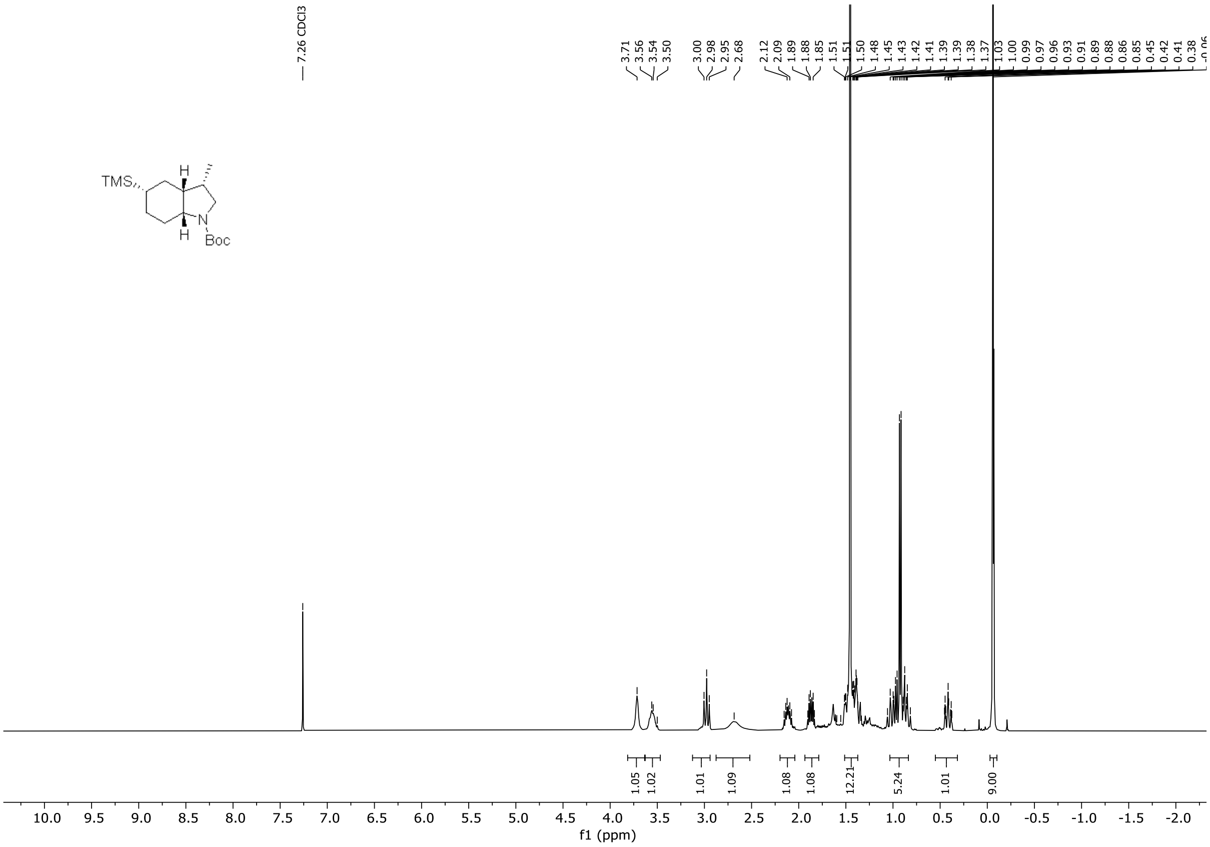

^{13}C NMR (101 MHz, Chloroform-*d*) of compound **2g**

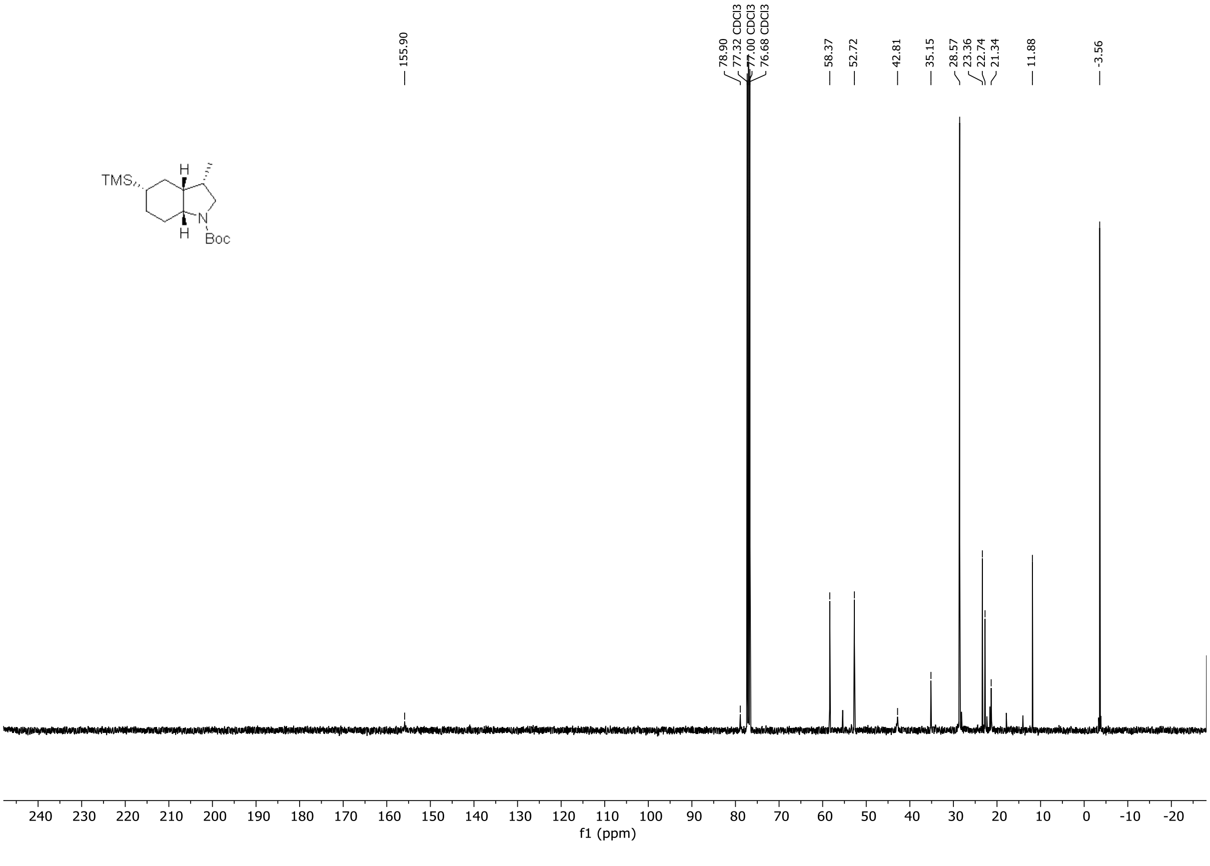

^{1}H NMR (400 MHz, Chloroform-*d*) of compound **2h**

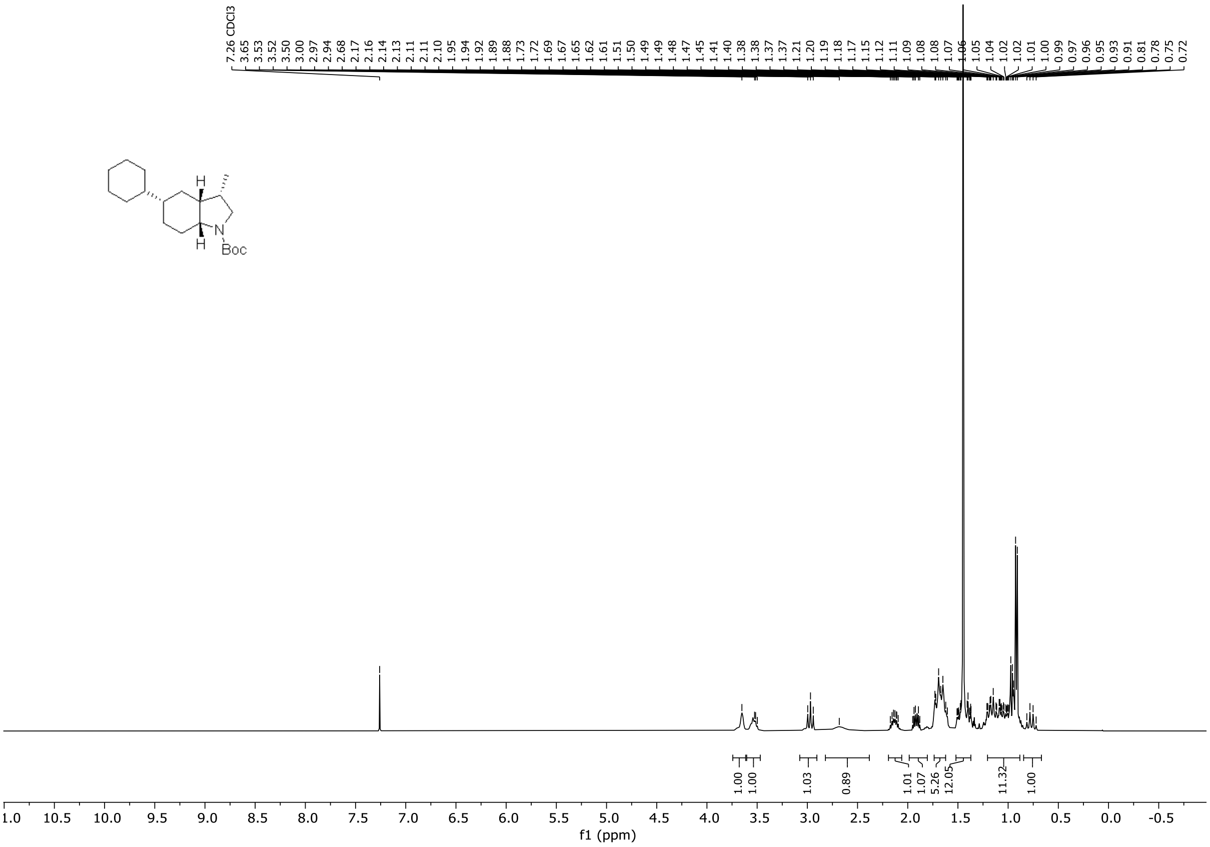

^{13}C NMR (101 MHz, Chloroform-*d*) of compound **2h**

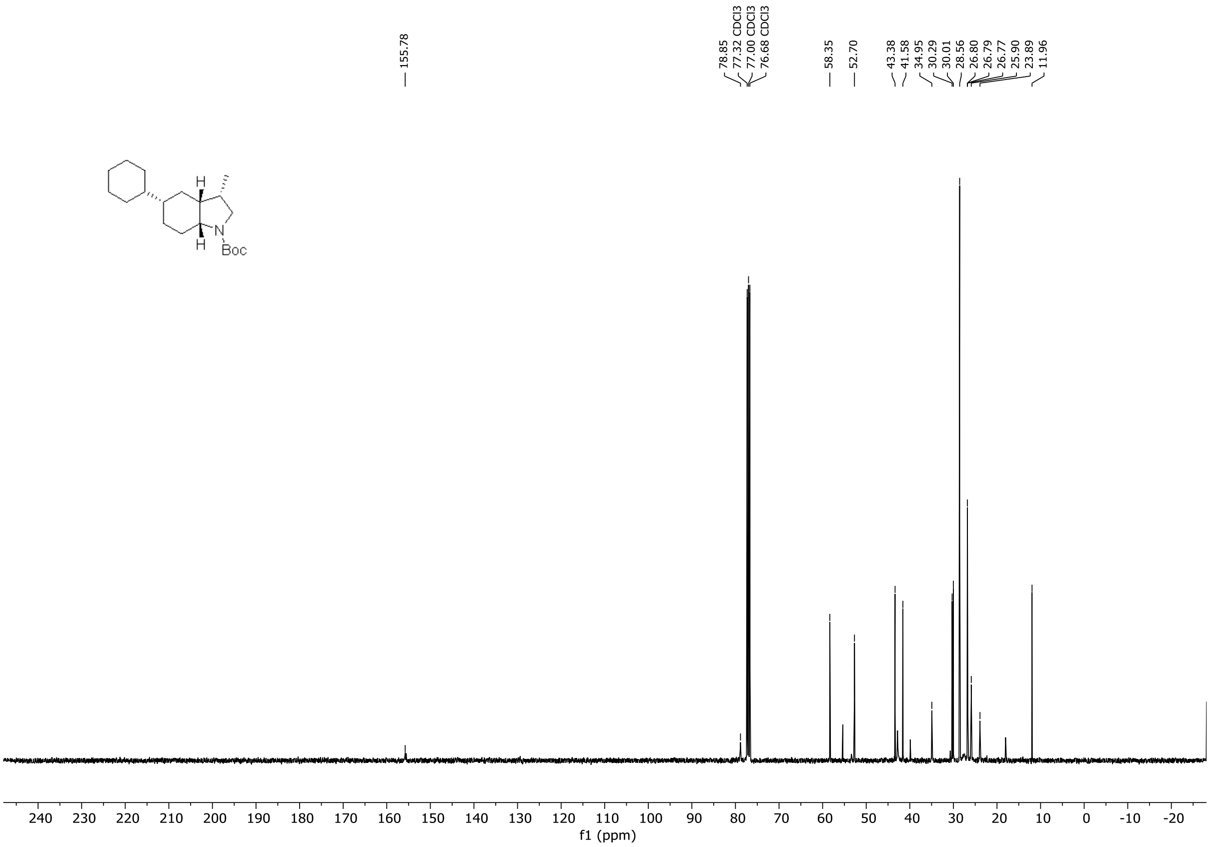

^{1}H NMR (400 MHz, Chloroform-*d*) of compound **2i**

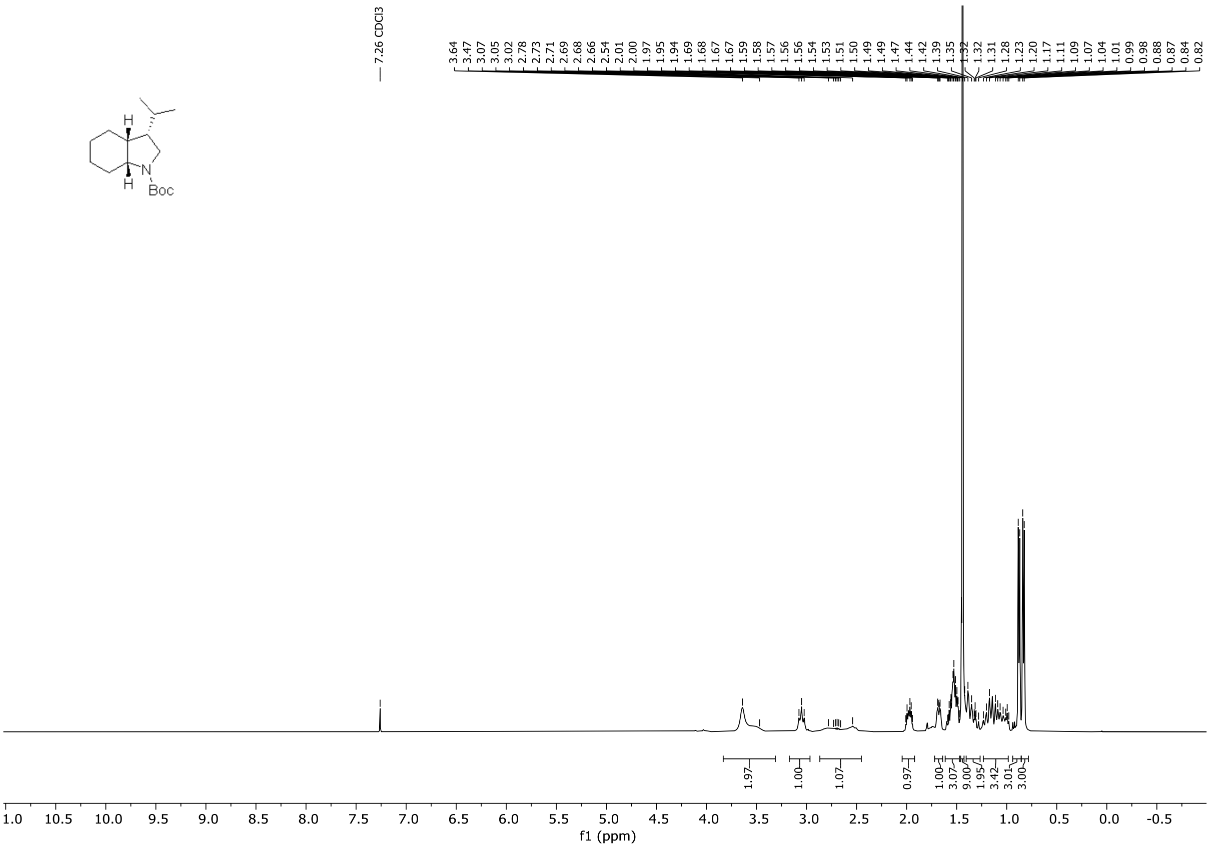

^{13}C NMR (101 MHz, Chloroform-*d*) of compound **2i**

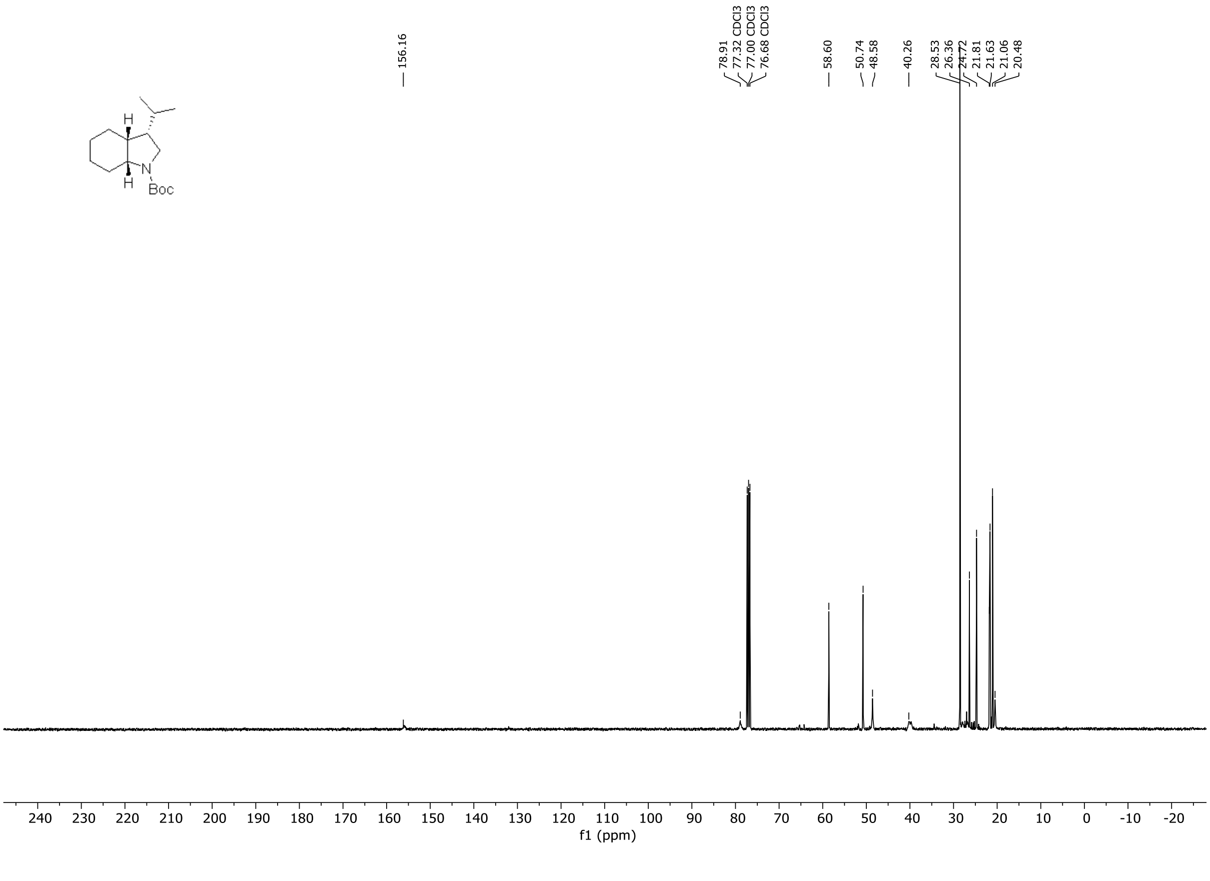

^{1}H NMR (400 MHz, Chloroform-*d*) of compound **2j**

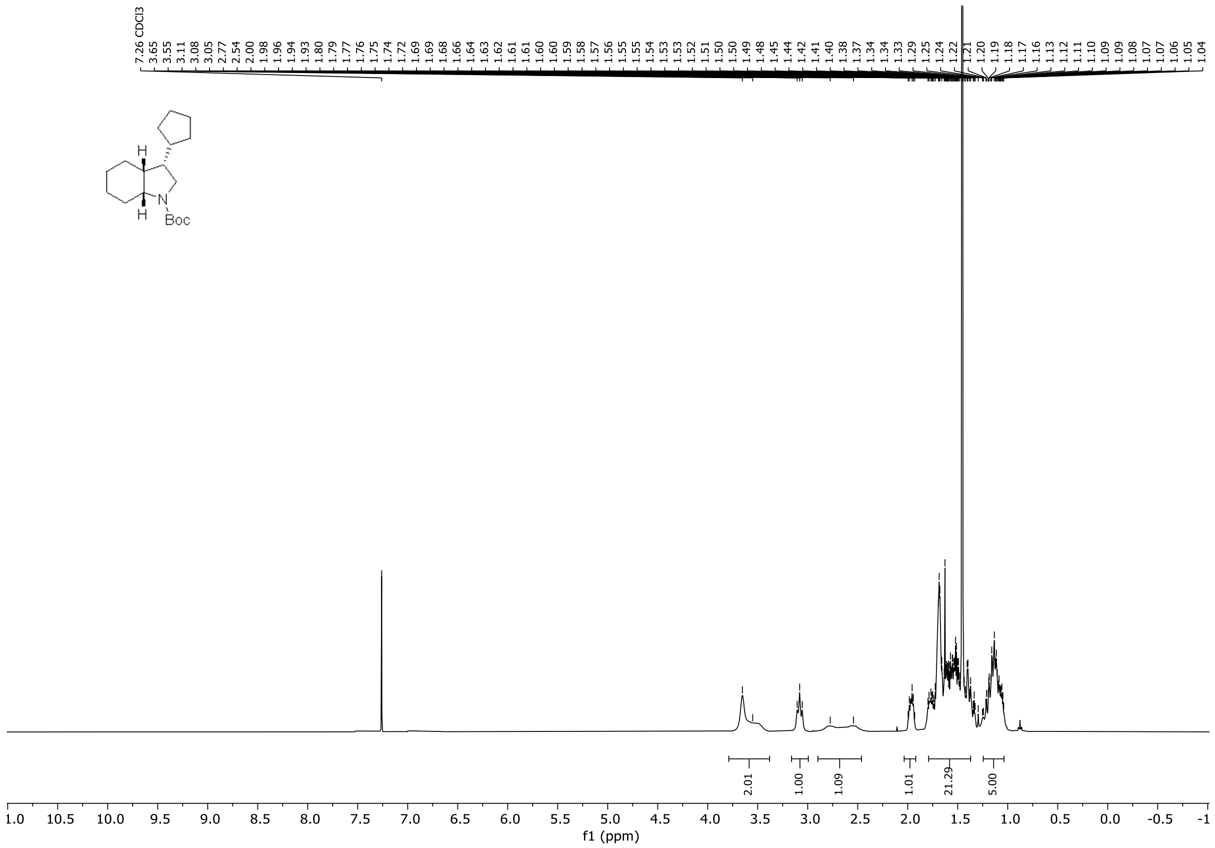

^{13}C NMR (101 MHz, Chloroform-*d*) of compound **2j**

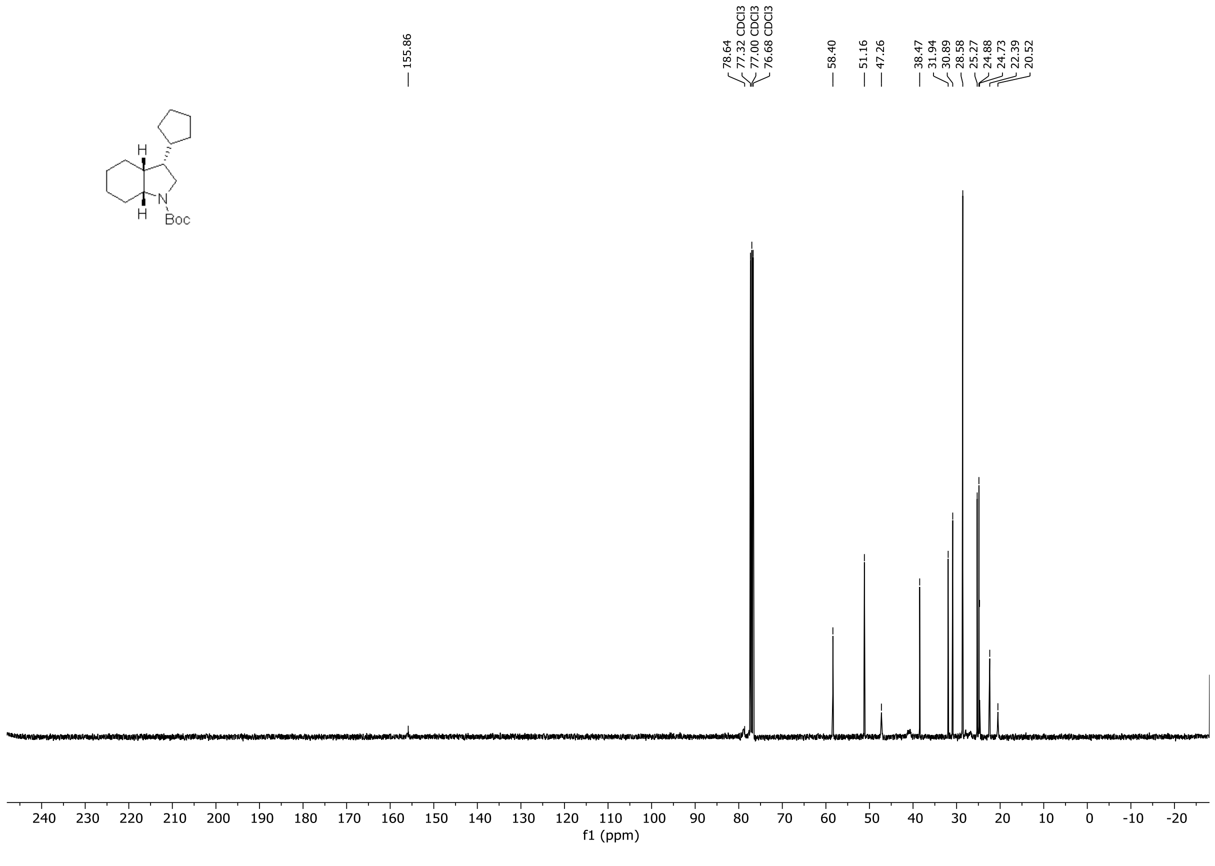

^{1}H NMR (400 MHz, Chloroform-d) of compound **2k**

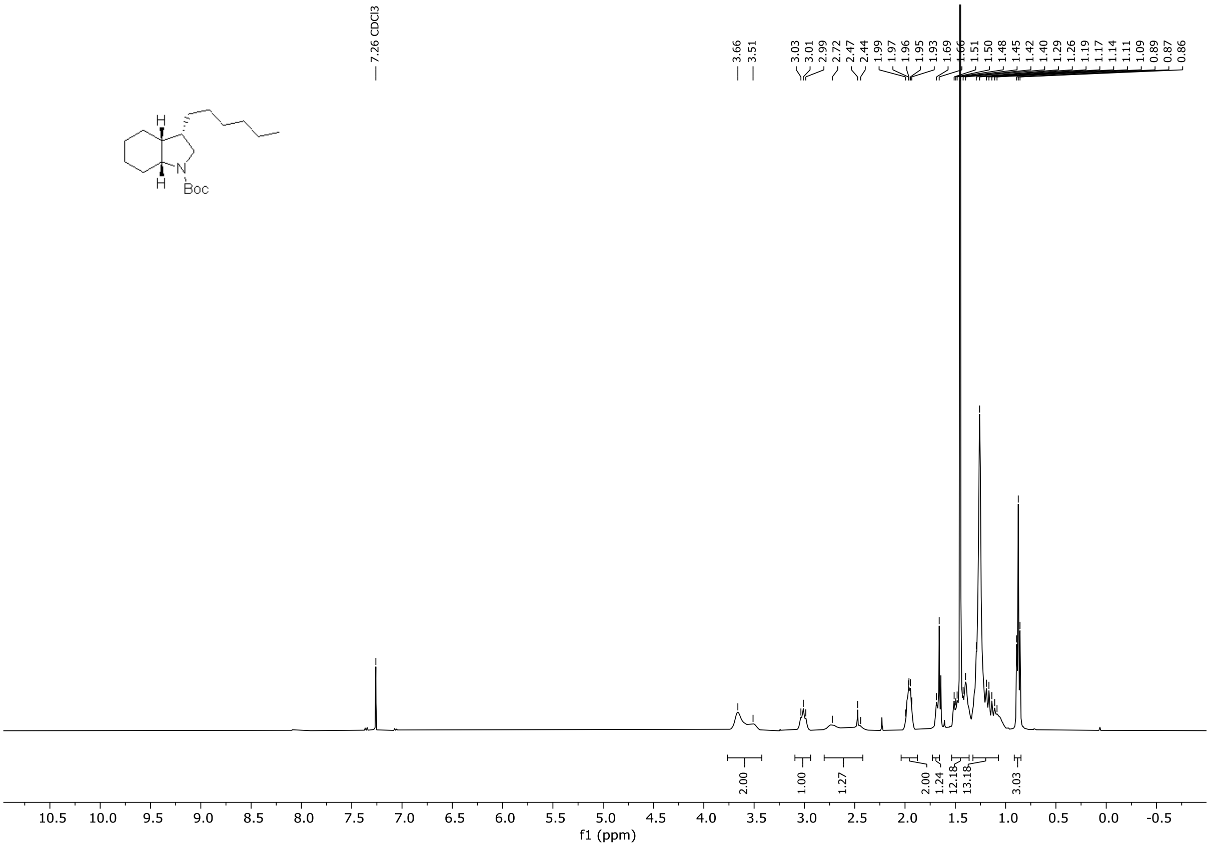

^{13}C NMR (101 MHz, Chloroform-d) of compound **2k**

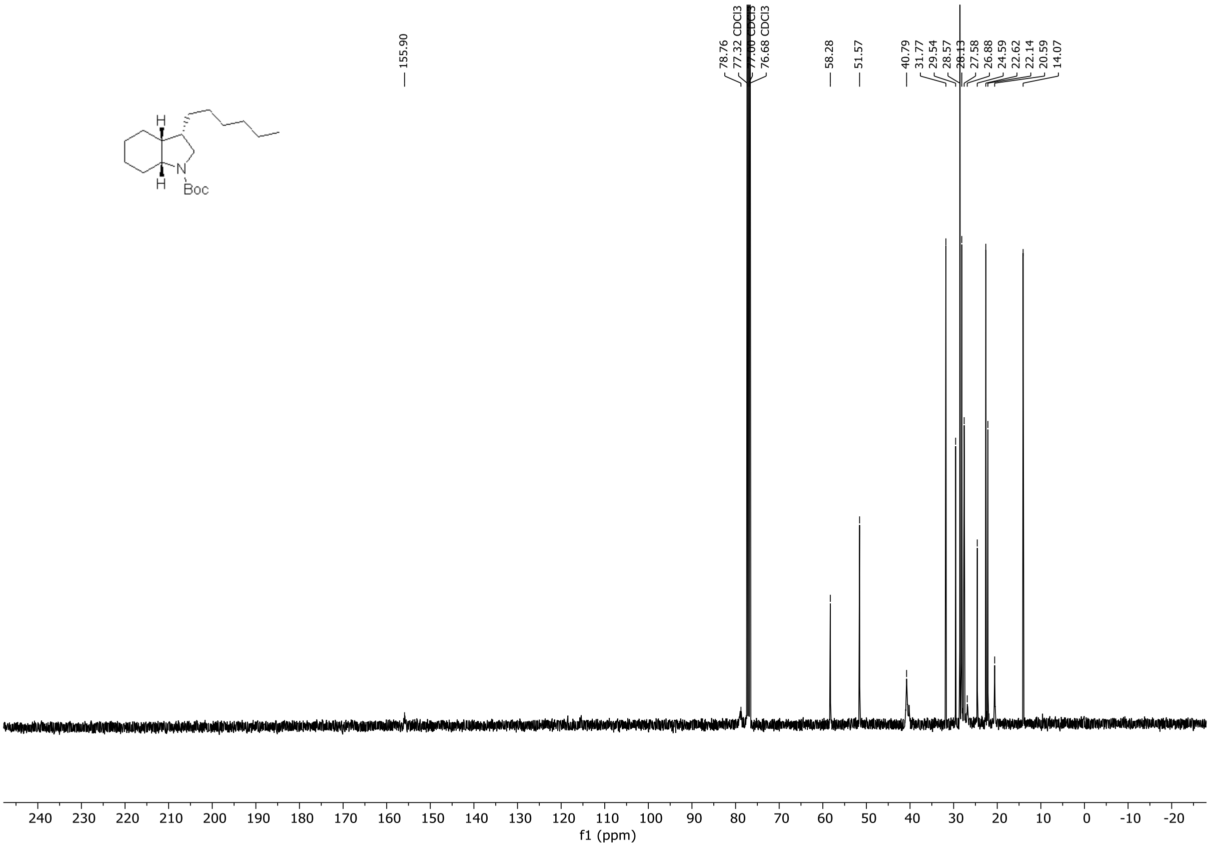

^{1}H NMR (400 MHz, Chloroform-*d*) of compound **2l**

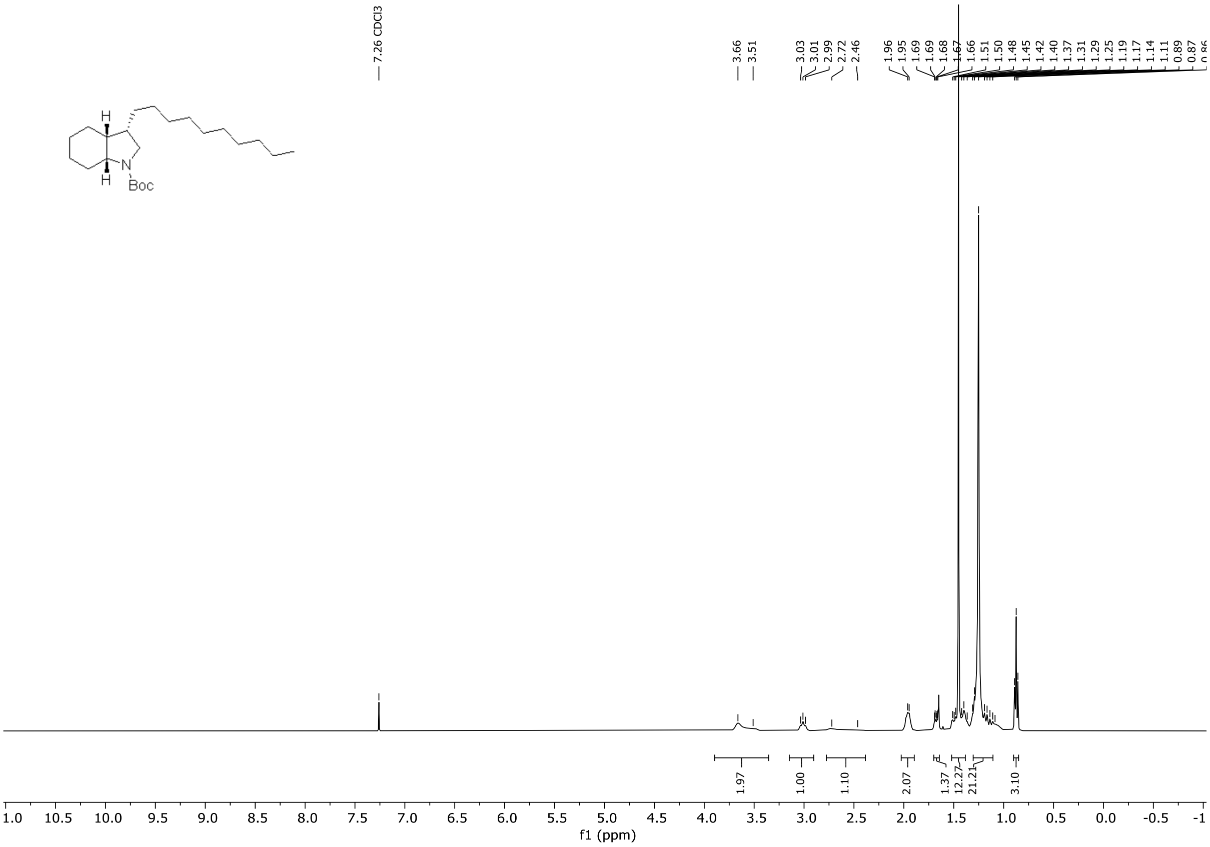

^{13}C NMR (101 MHz, Chloroform-*d*) of compound **2l**

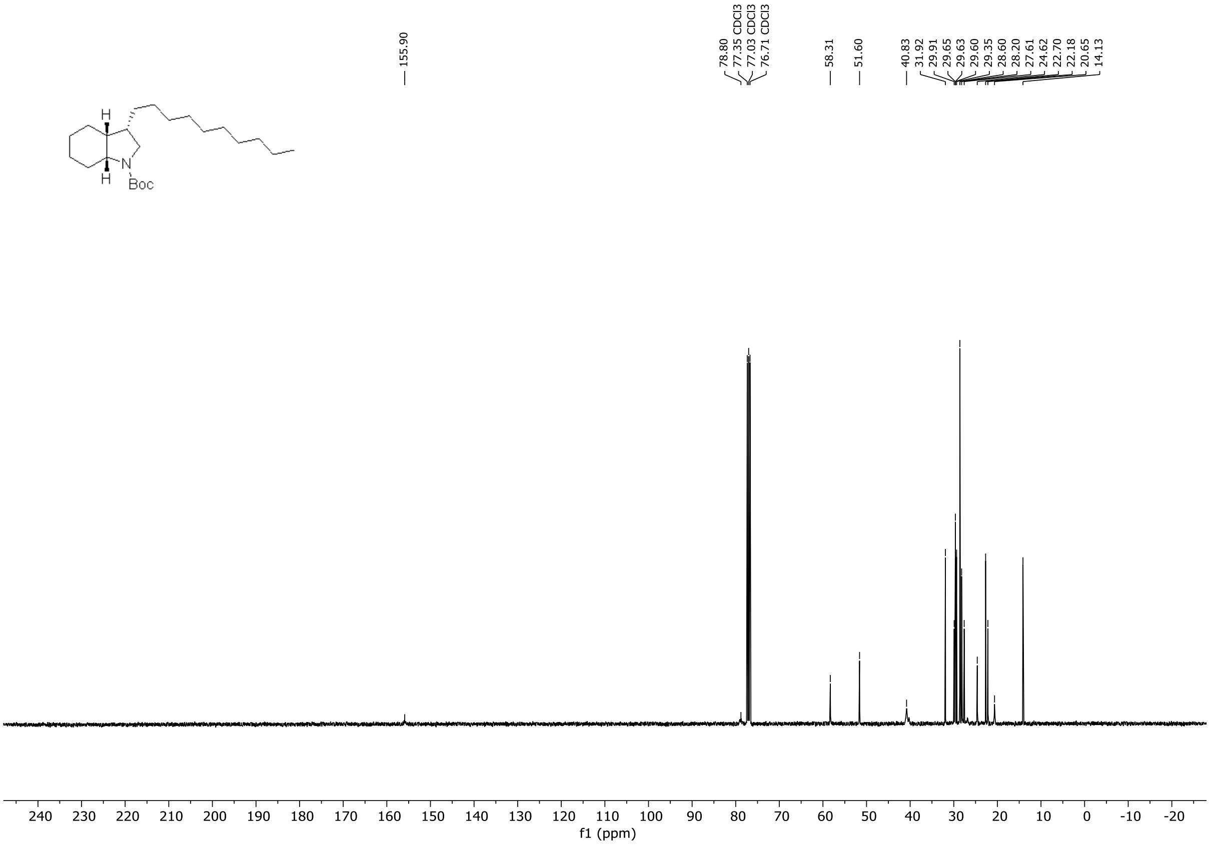

^{1}H NMR (400 MHz, Chloroform-*d*) of compound **2m**

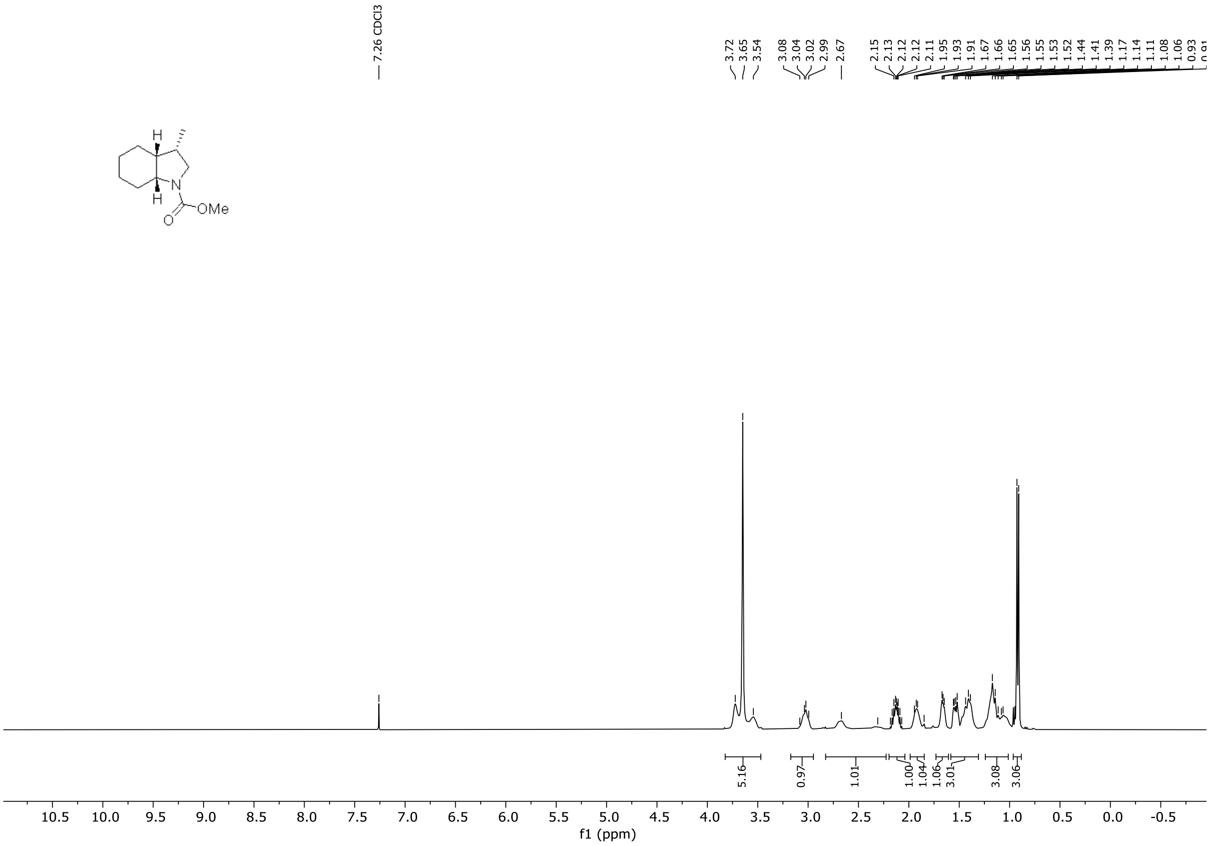

^{13}C NMR (101 MHz, Chloroform-*d*) of compound **2m**

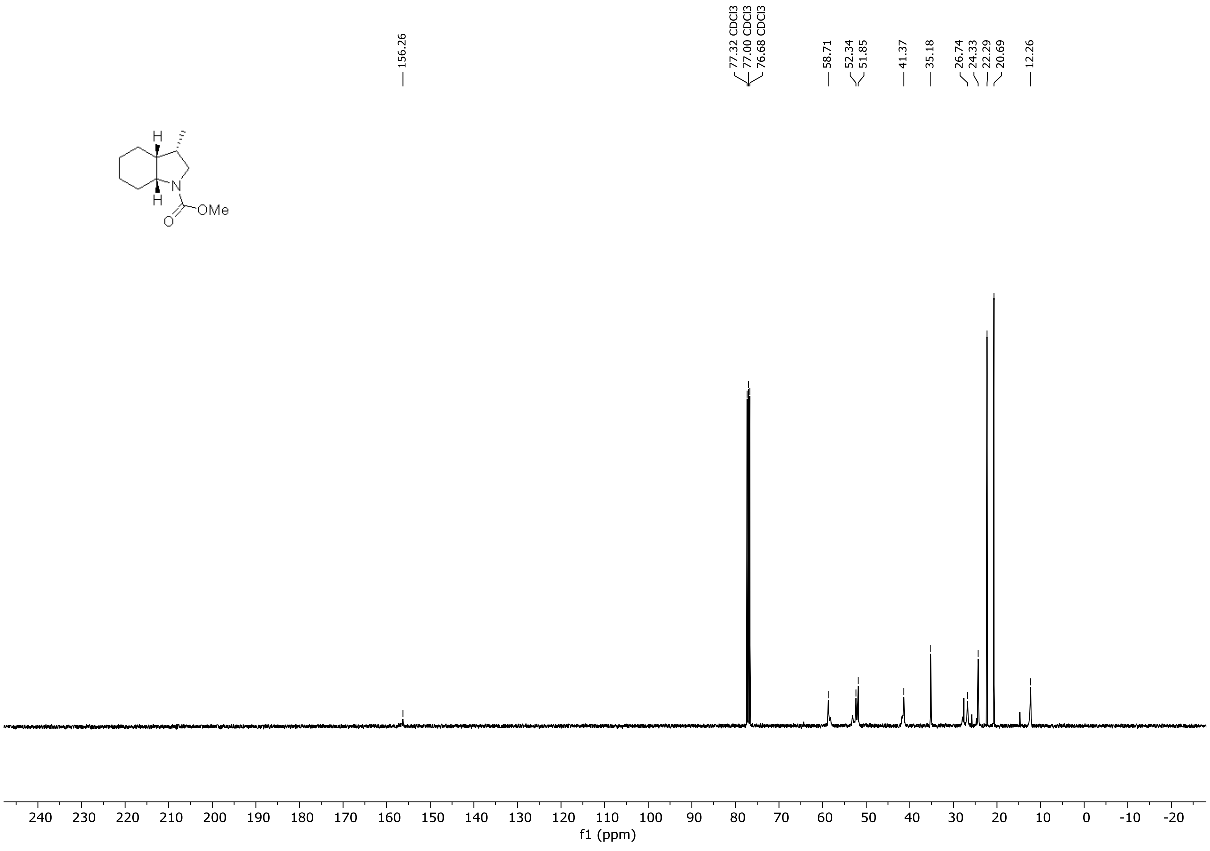

^{1}H NMR (400 MHz, Chloroform-*d*) of compound **5a**

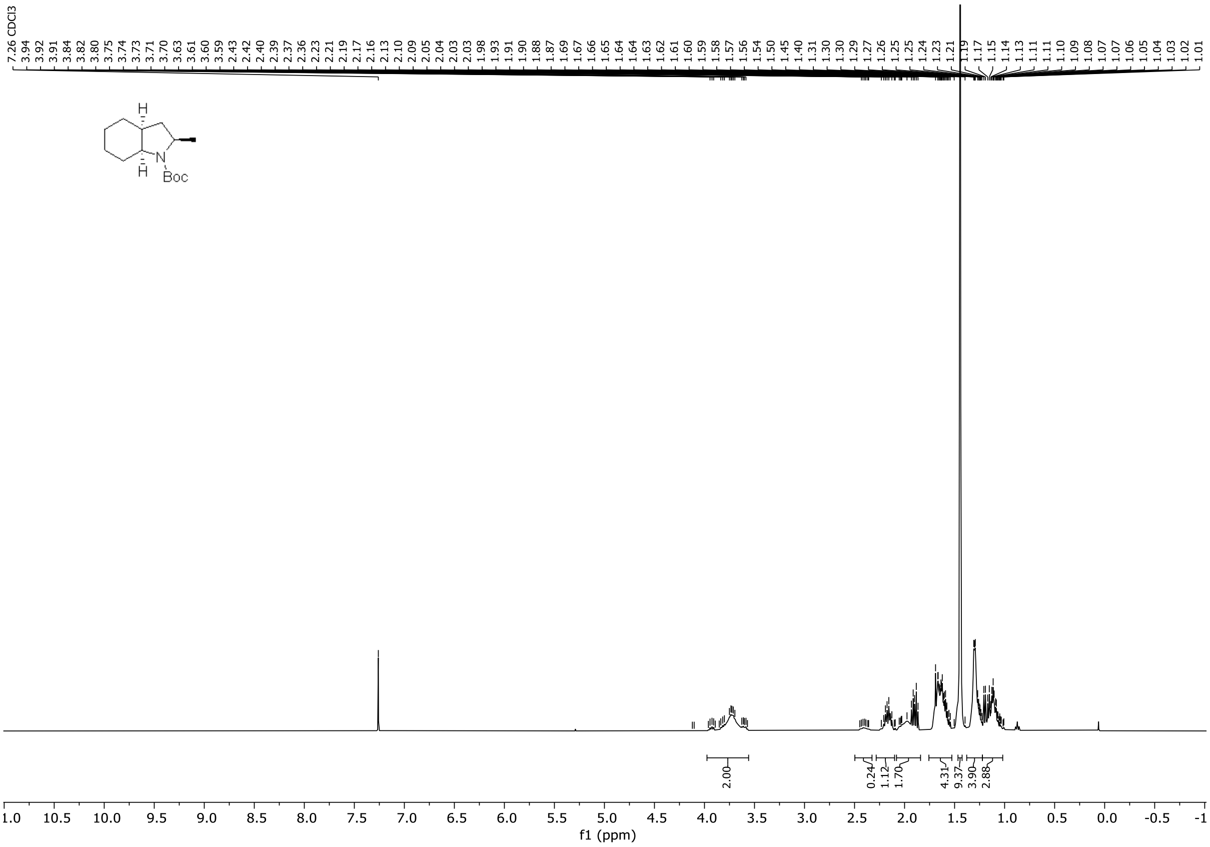

^{13}C NMR (101 MHz, Chloroform-*d*) of compound **5a**

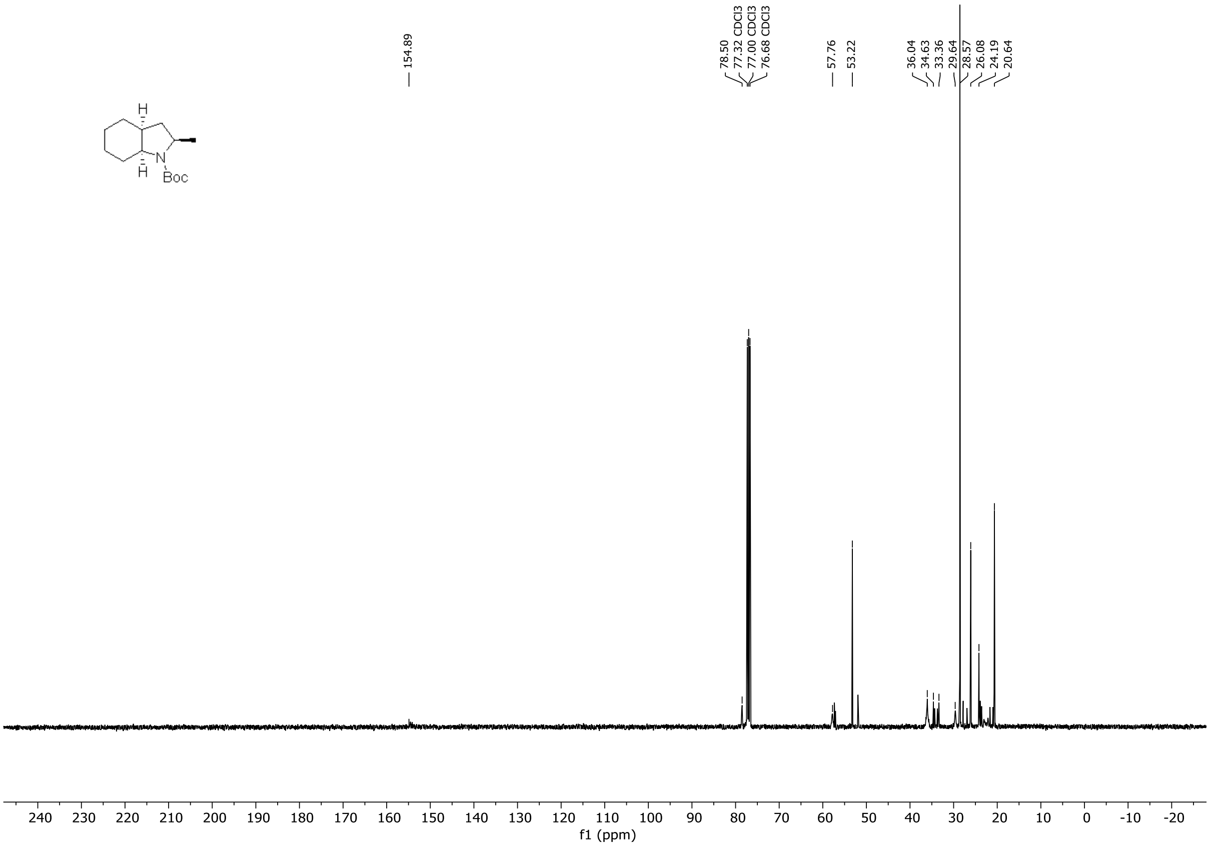

^{1}H NMR (400 MHz, Chloroform-*d*) of compound **5b** ^{13}C NMR (101 MHz, Chloroform-*d*) of compound **5b**

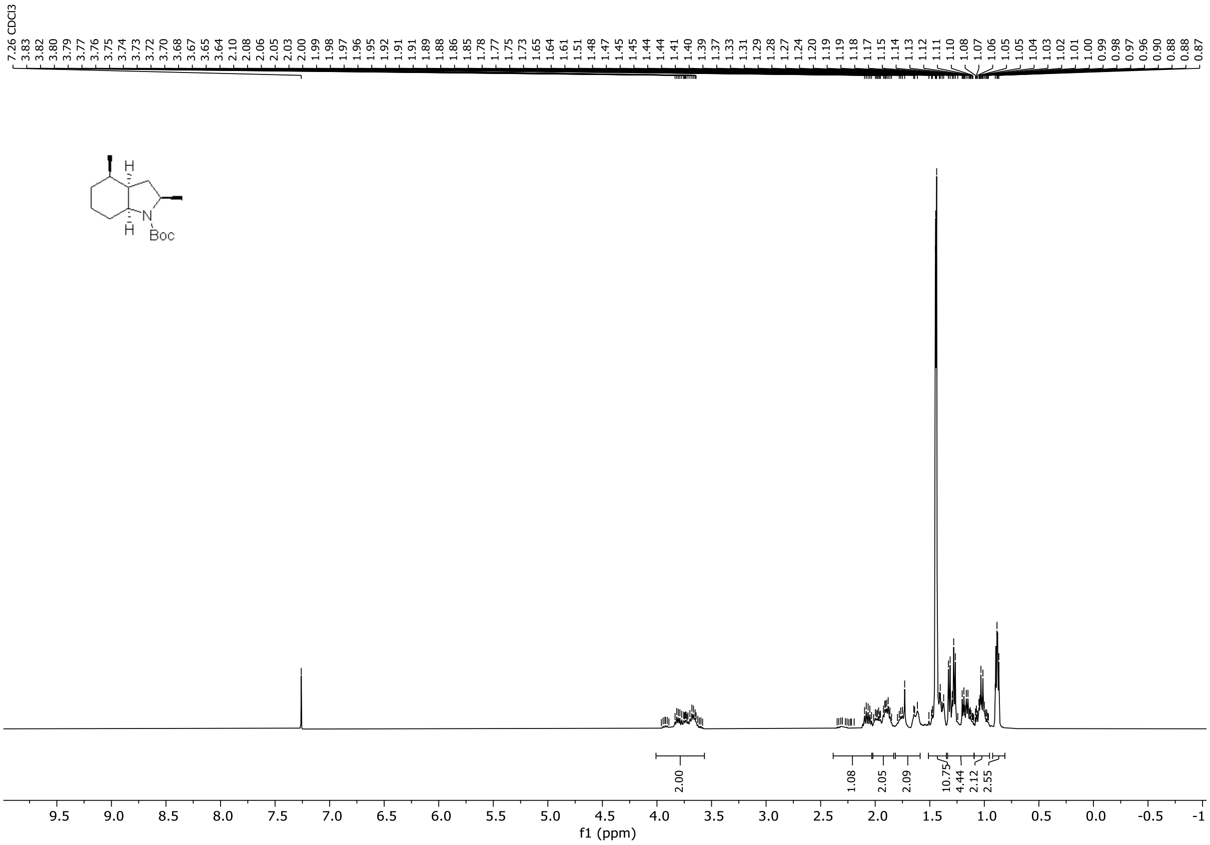

^{13}C NMR (101 MHz, Chloroform-*d*) of compound **5b**

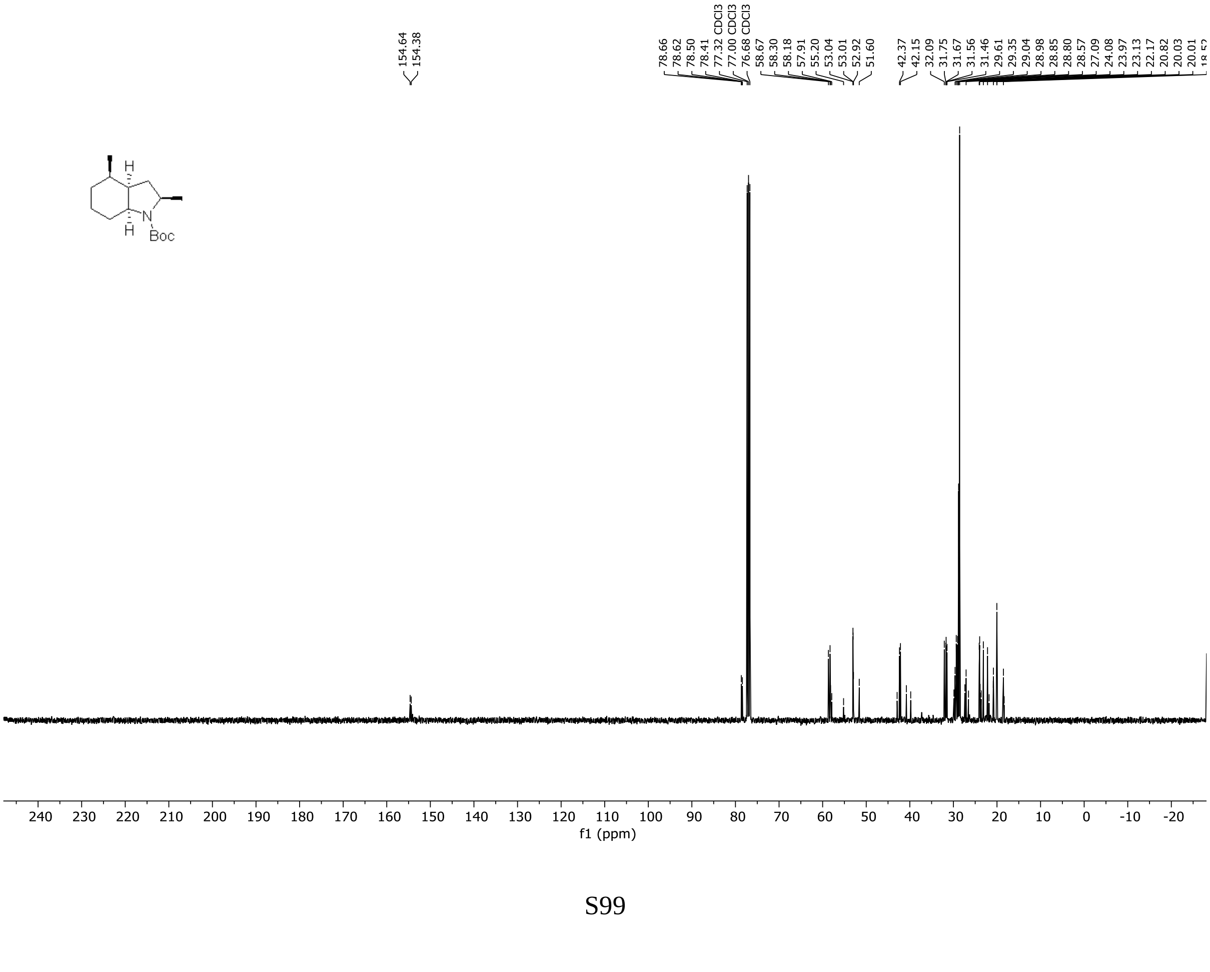

^{1}H NMR (400 MHz, Chloroform-*d*) of compound **5c**

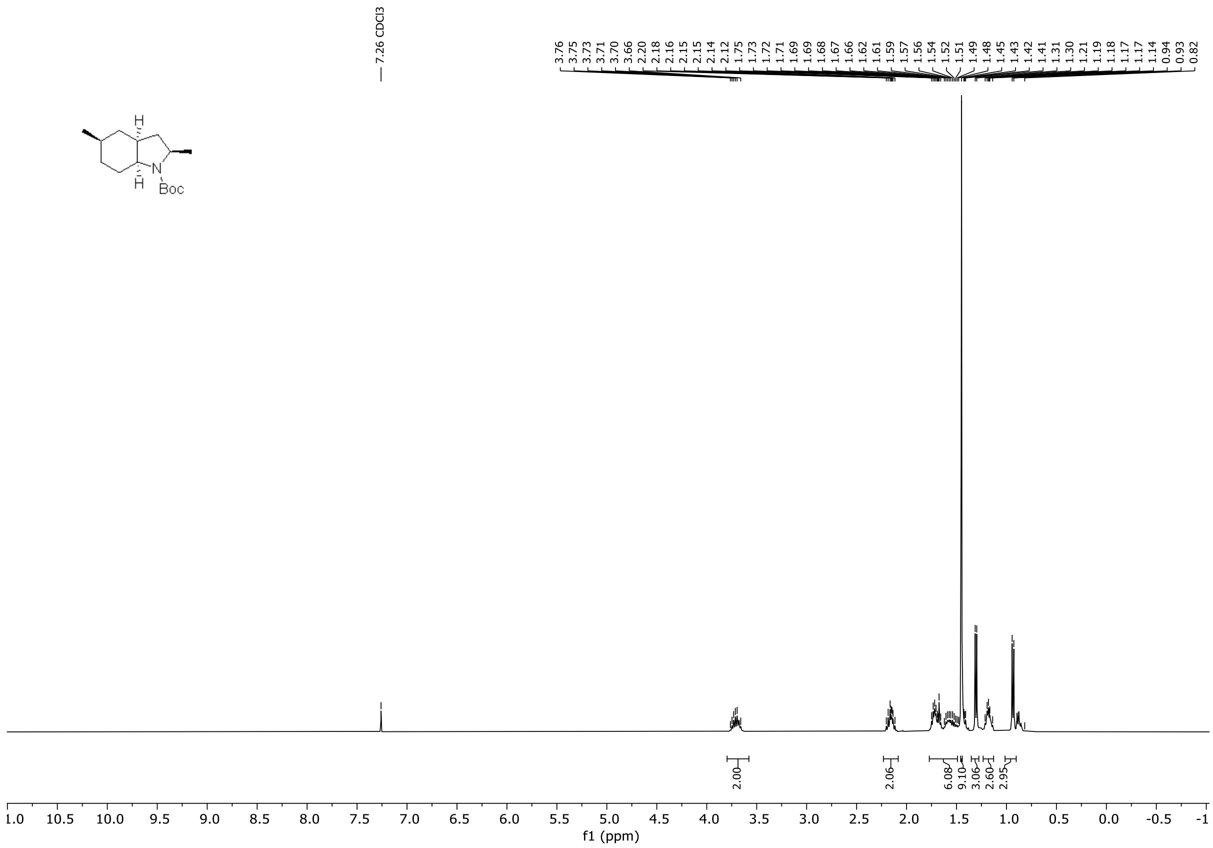

^{13}C NMR (101 MHz, Chloroform-*d*) of compound **5c**

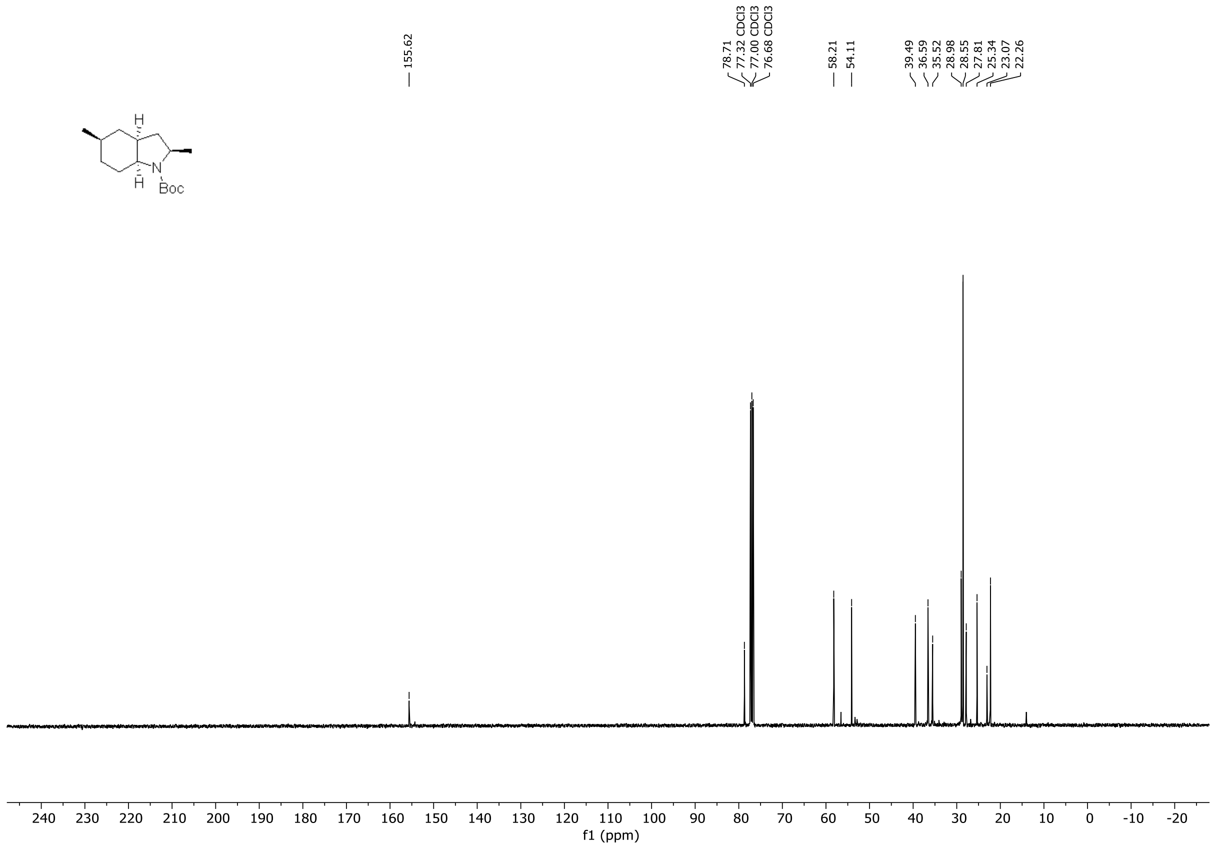

^{1}H NMR (400 MHz, Chloroform-*d*) of compound **5d**

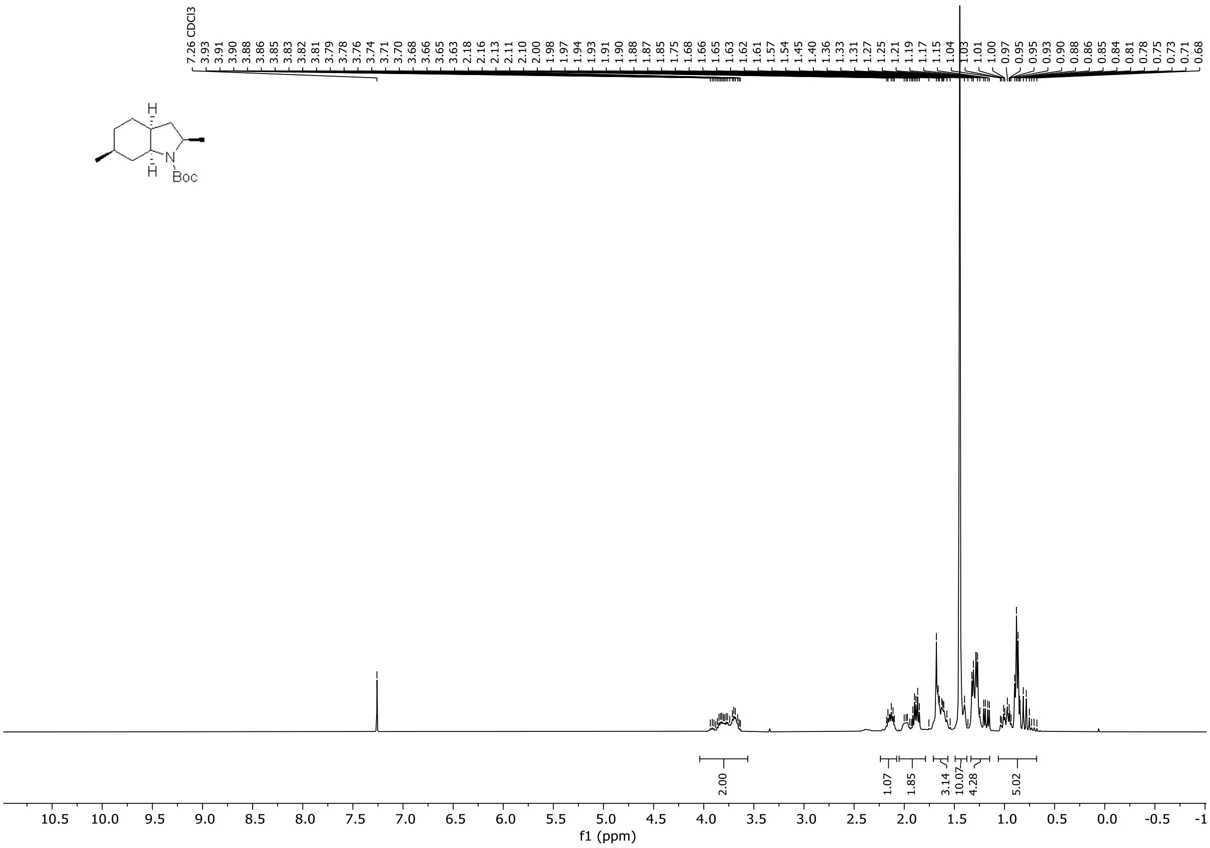

^{13}C NMR (101 MHz, Chloroform-*d*) of compound **5d**

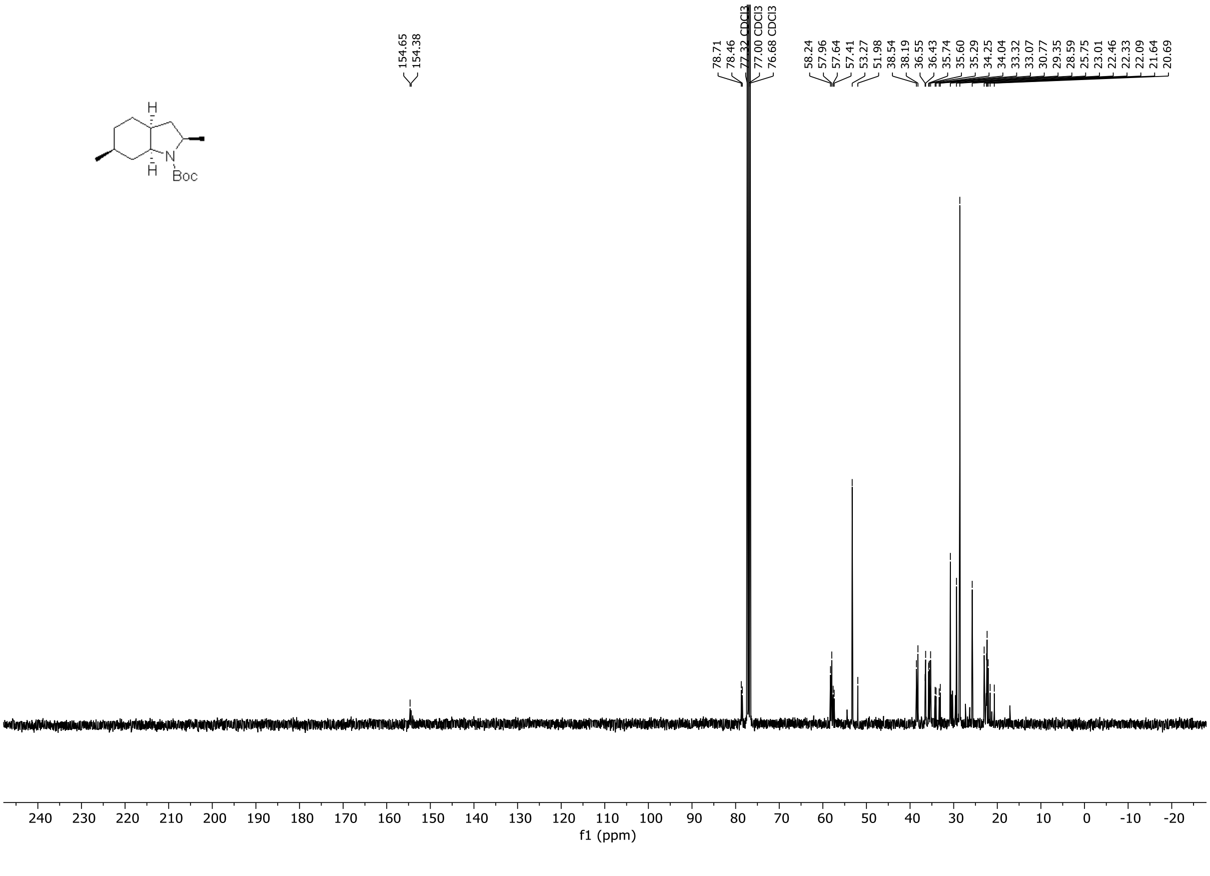

^{1}H NMR (400 MHz, Chloroform-*d*) of compound **5e**

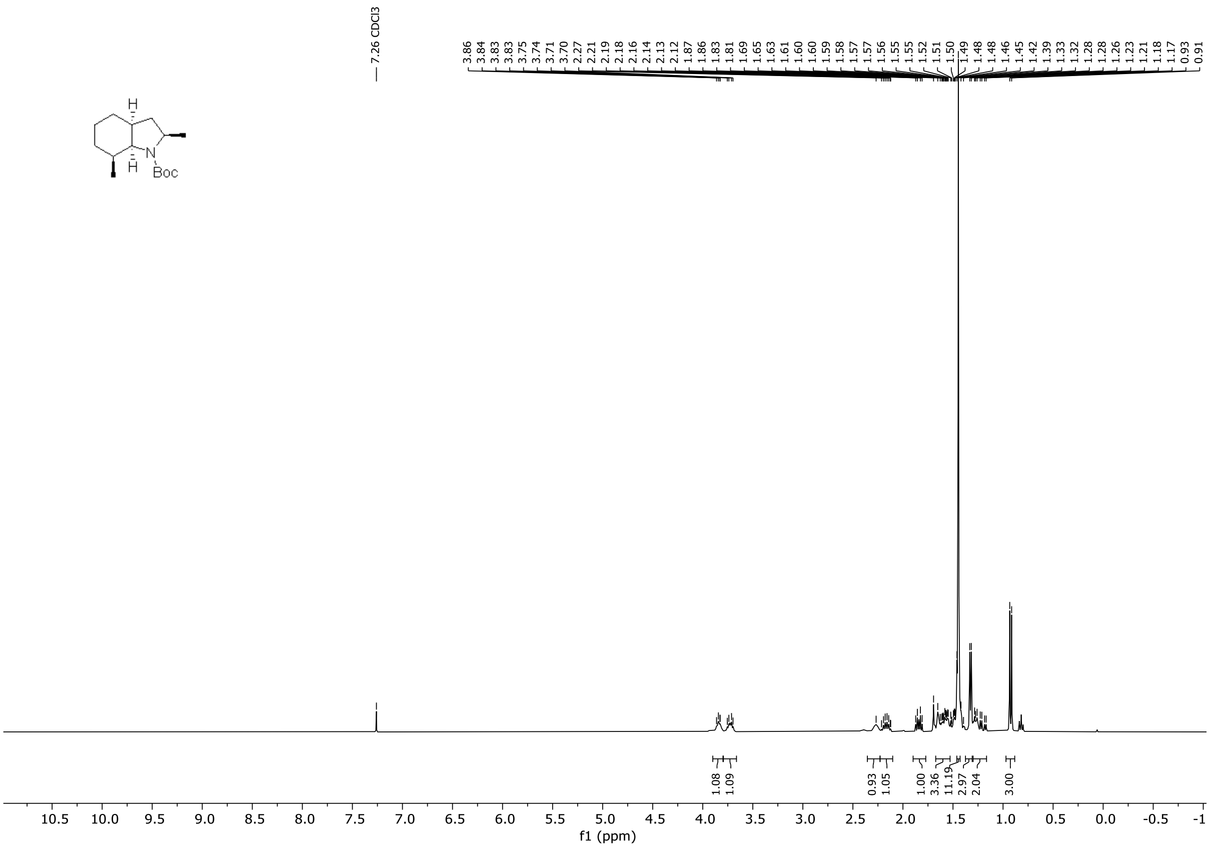

^{13}C NMR (101 MHz, Chloroform-*d*) of compound **5e**

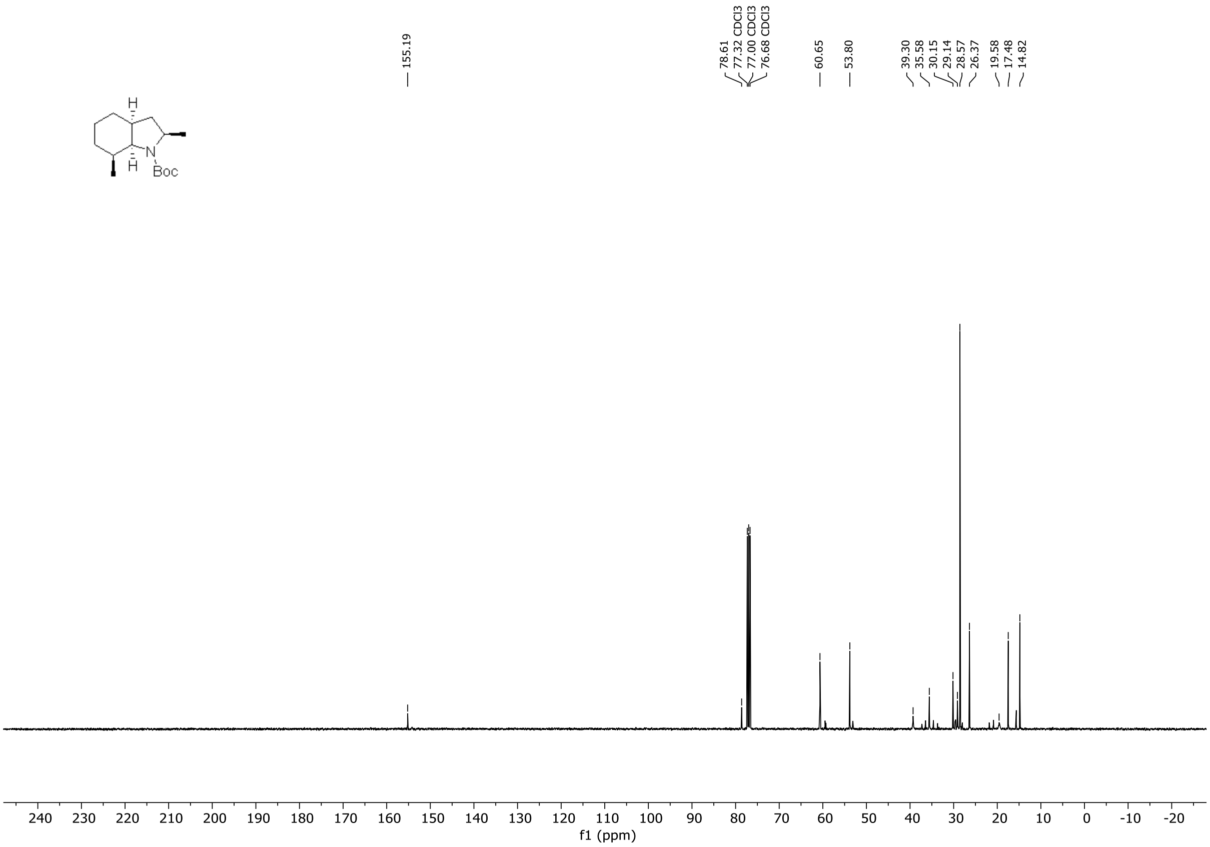

^{1}H NMR (400 MHz, Chloroform-*d*) of compound **5f**

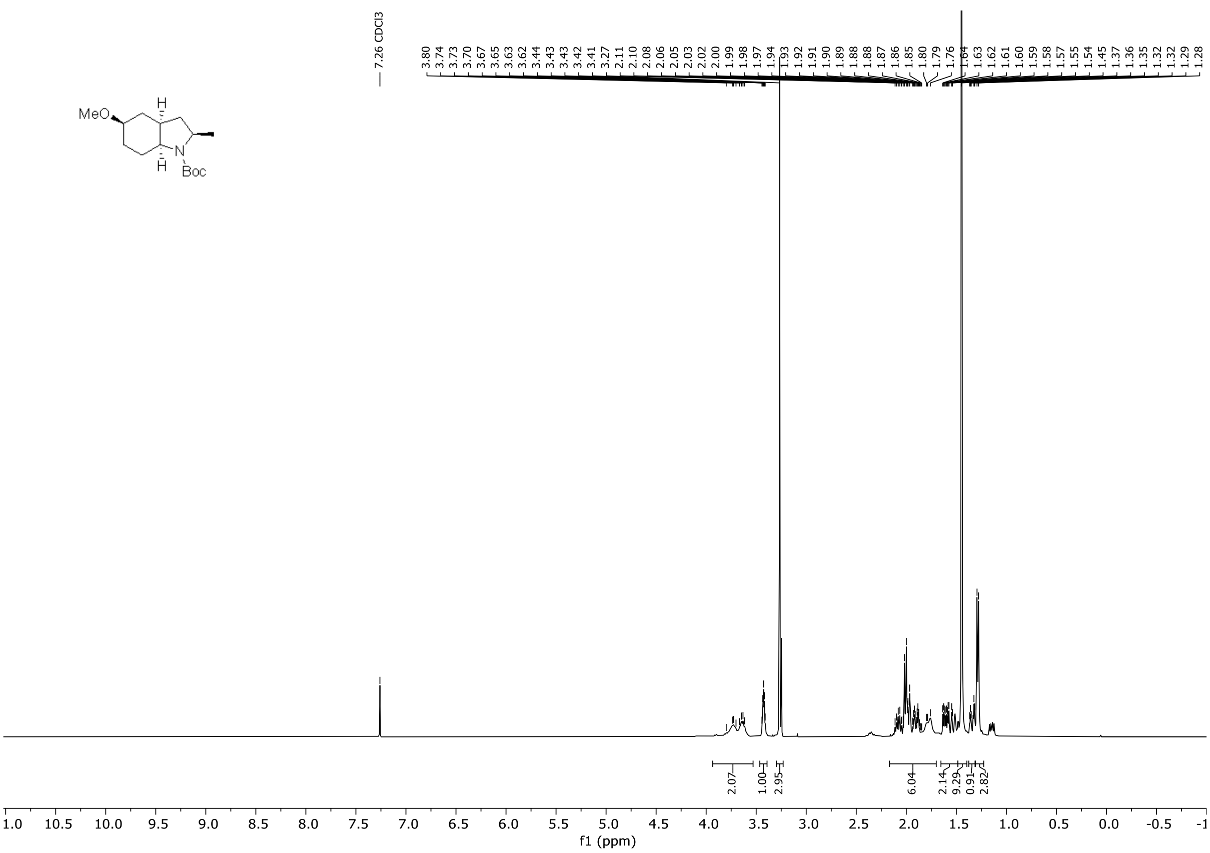

^{13}C NMR (101 MHz, Chloroform-*d*) of compound **5f**

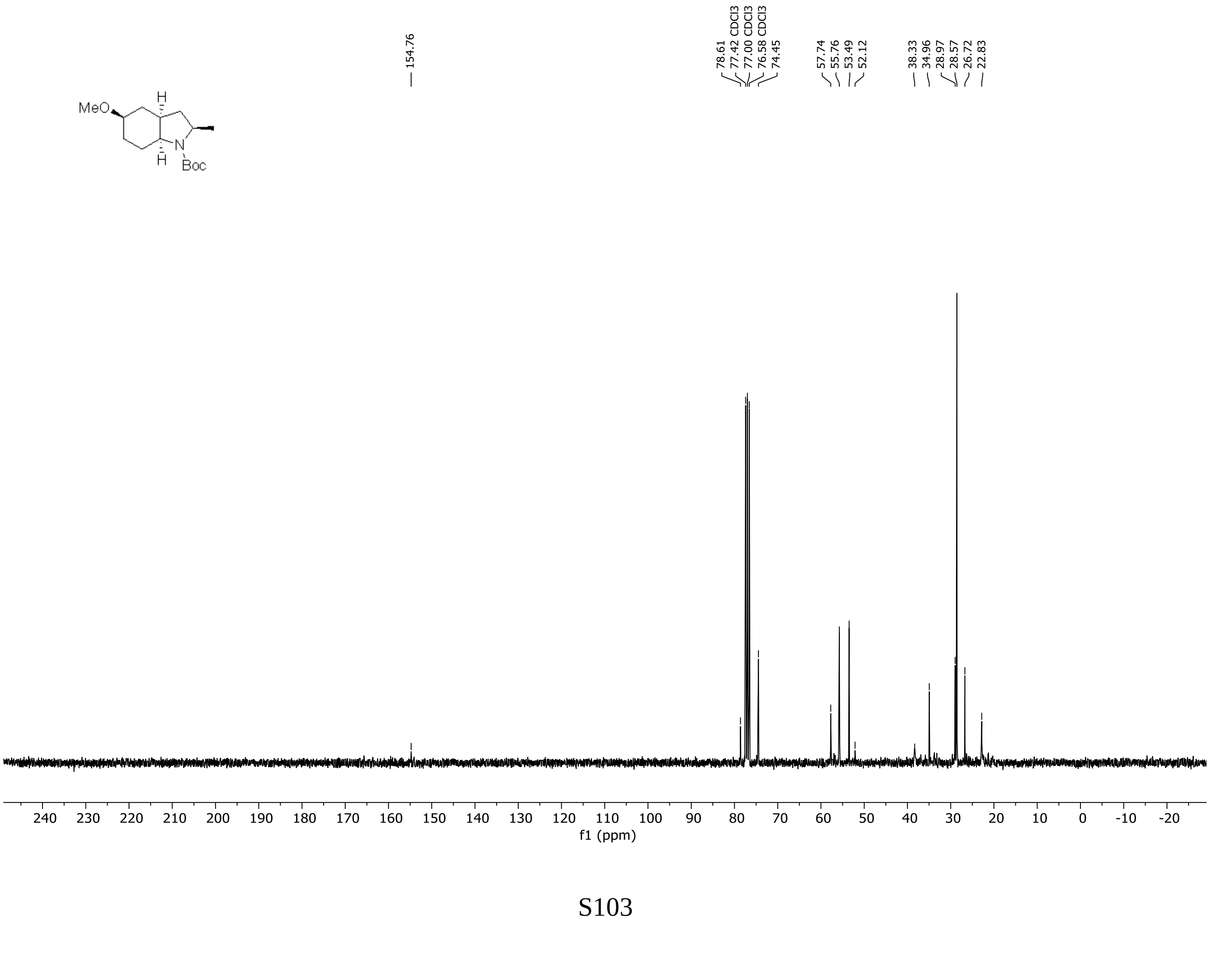

^{1}H NMR (400 MHz, Chloroform-*d*) of compound **5g**

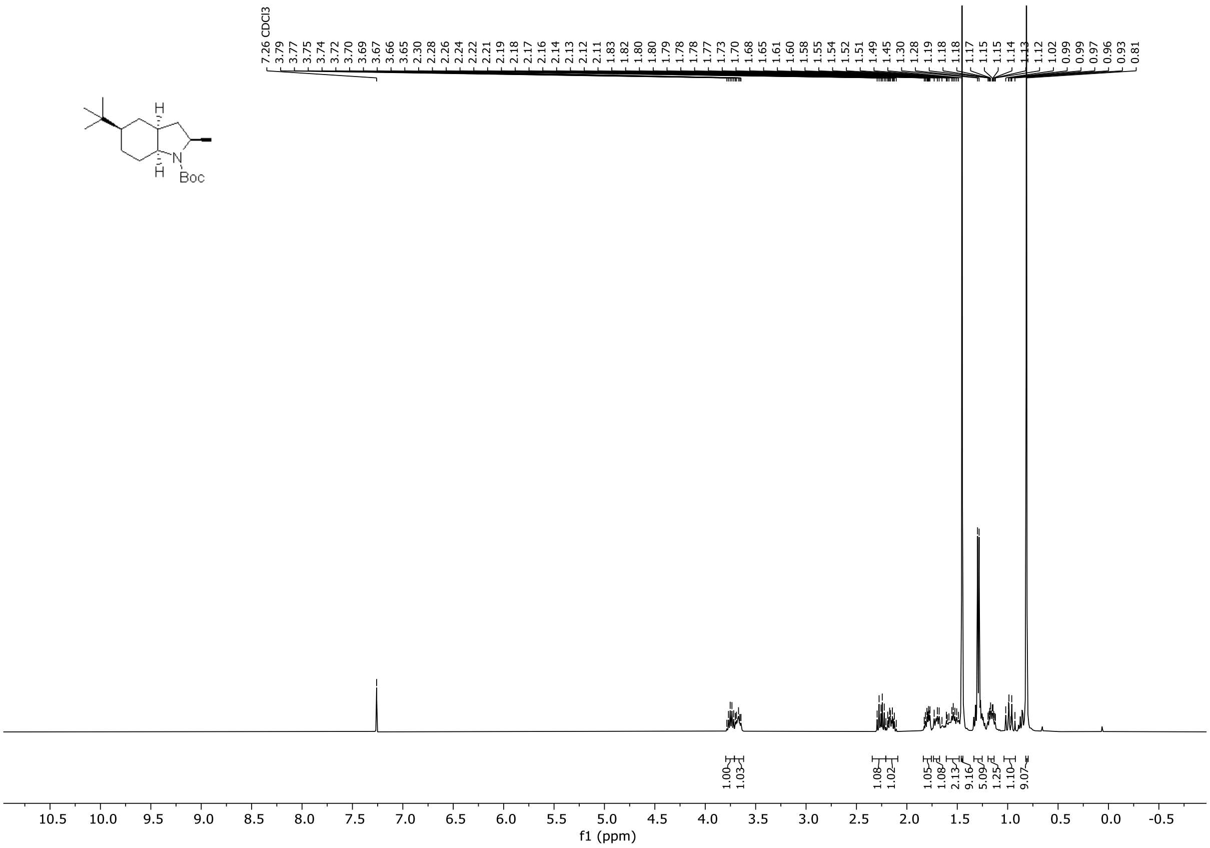

^{13}C NMR (101 MHz, Chloroform-*d*) of compound **5g**

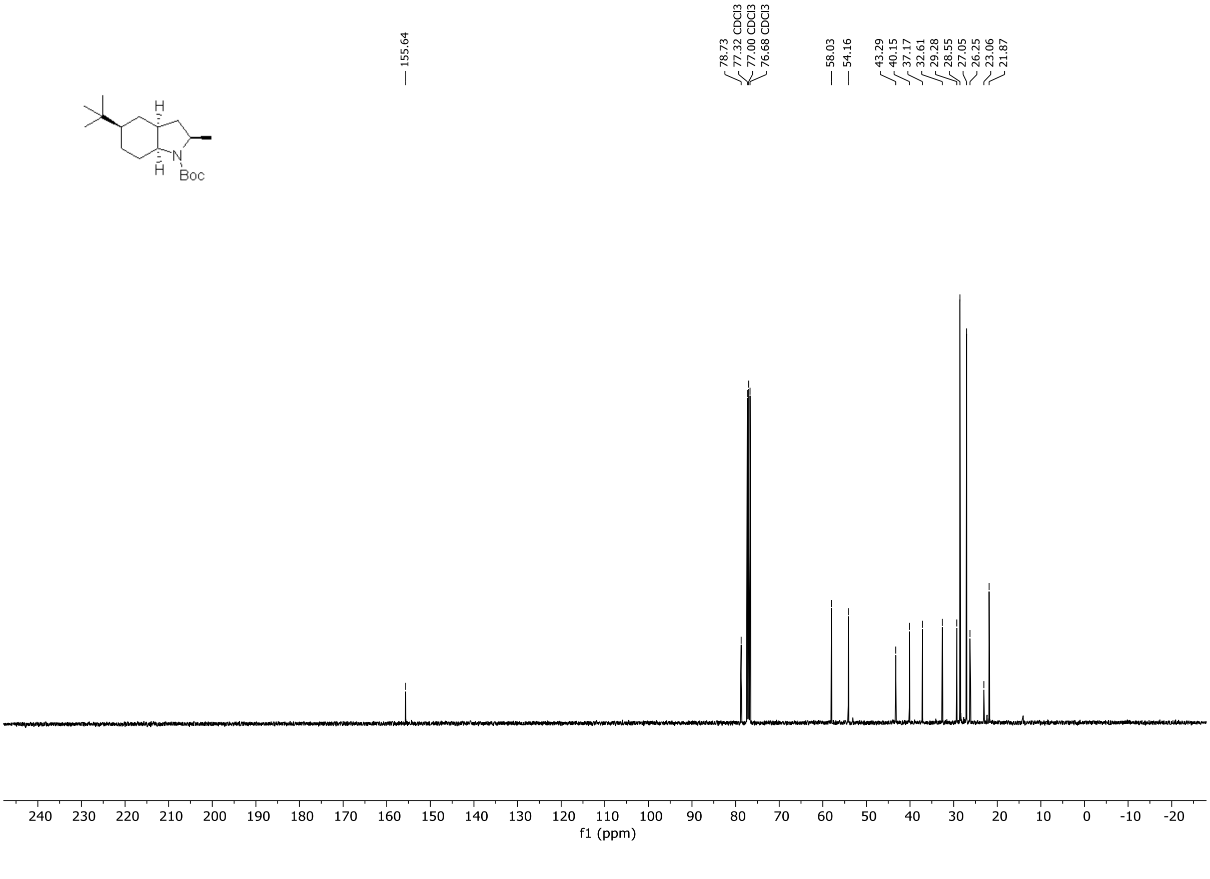

^{1}H NMR (400 MHz, Chloroform-*d*) of compound **5h**

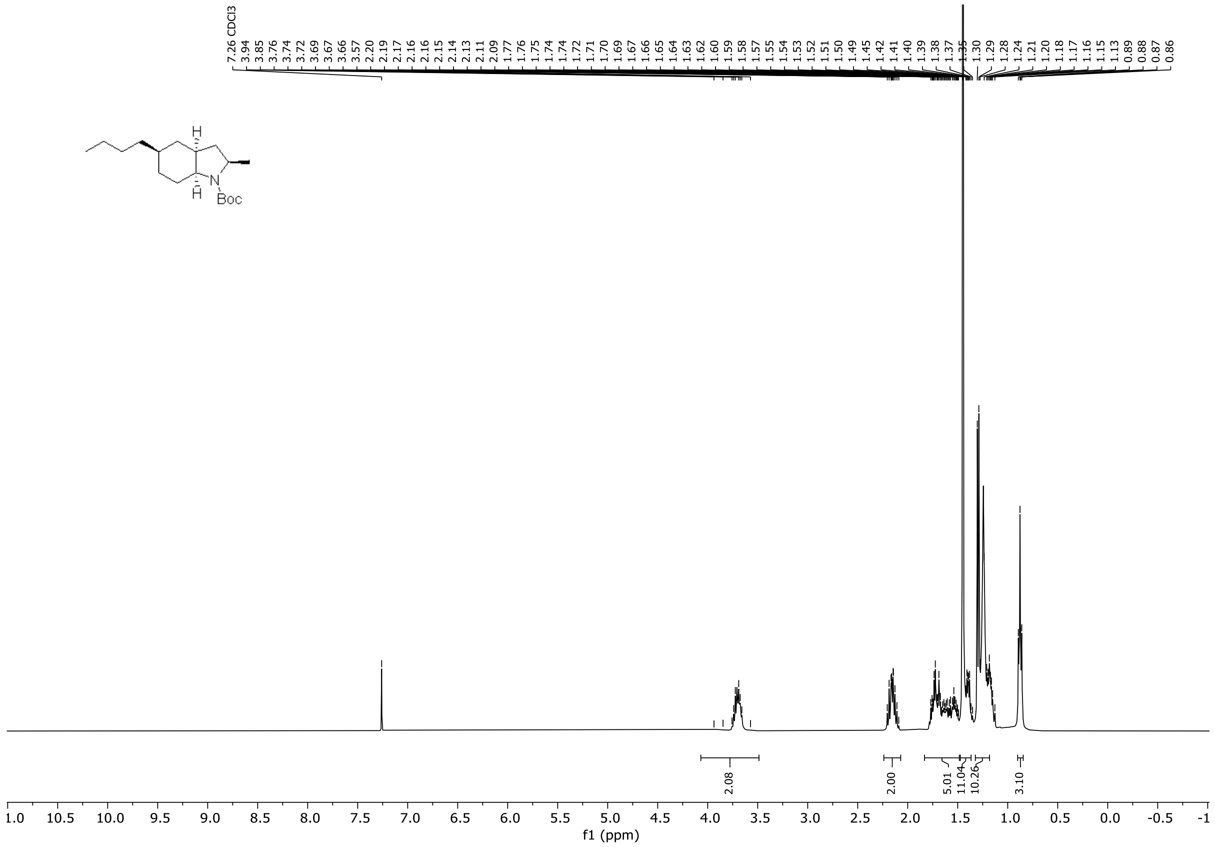

^{13}C NMR (101 MHz, Chloroform-*d*) of compound **5h**

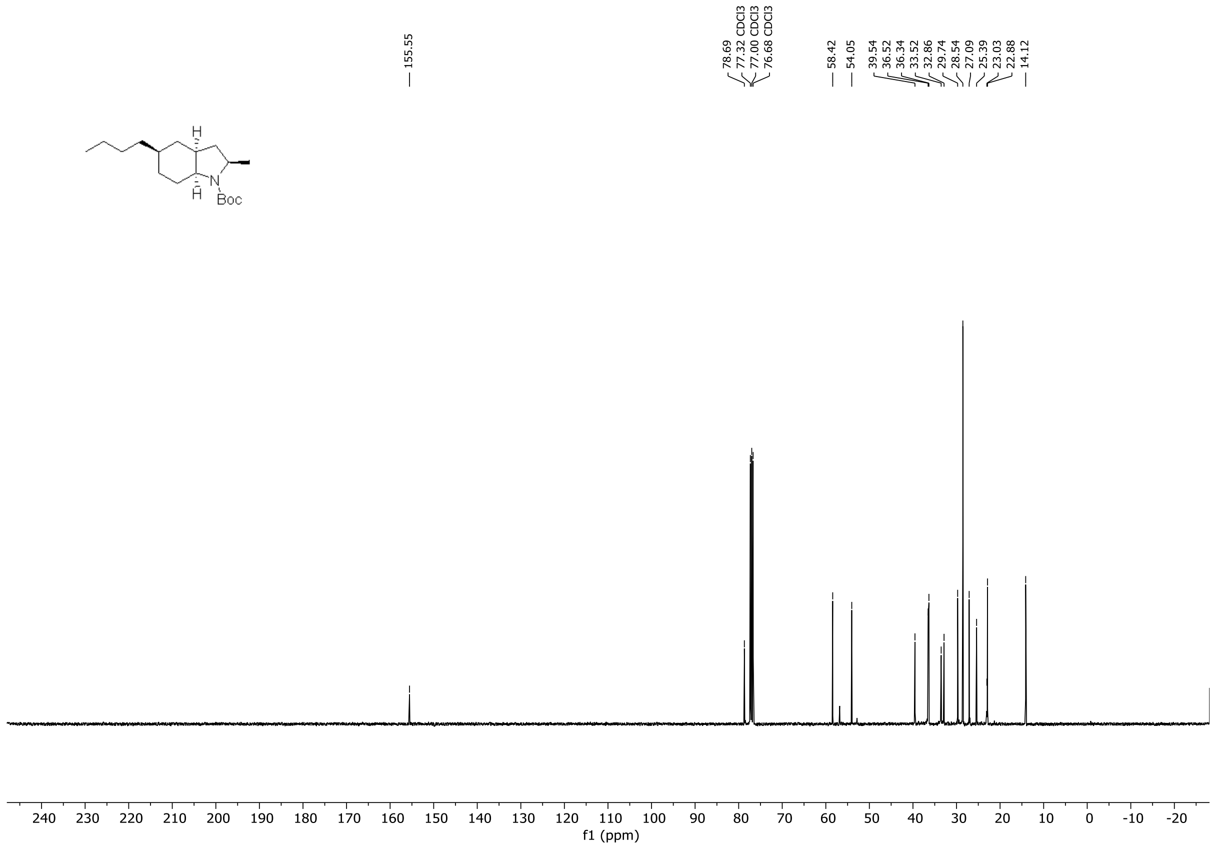

^{1}H NMR (400 MHz, Chloroform-*d*) of compound **5i**

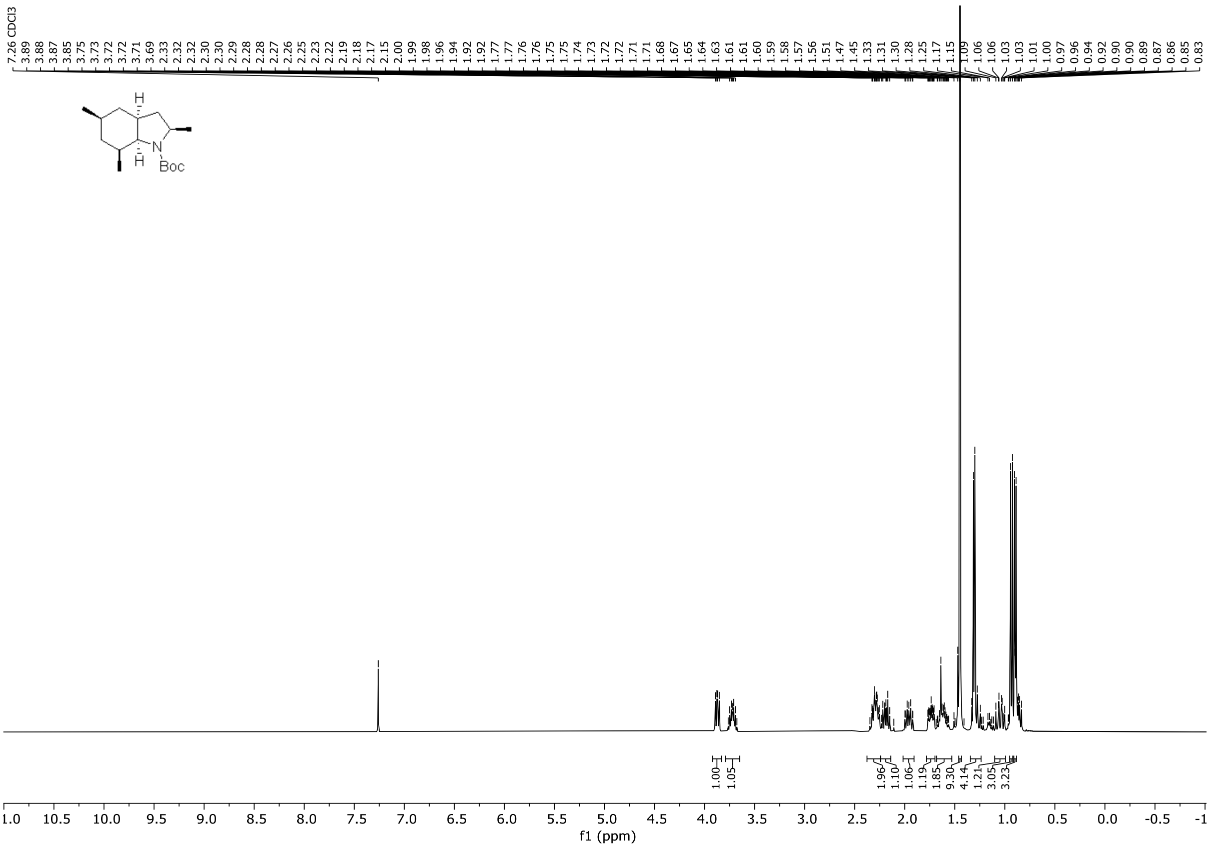

^{13}C NMR (101 MHz, Chloroform-*d*) of compound **5i**

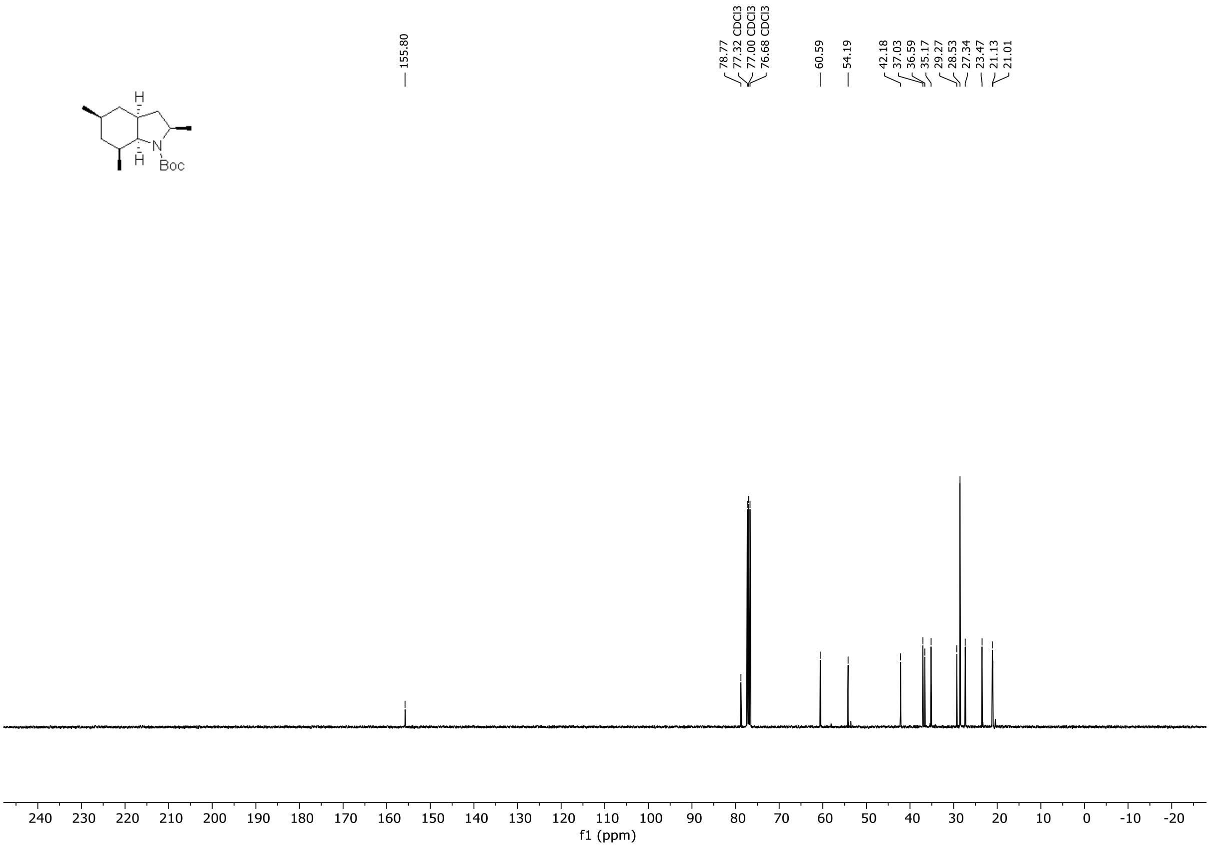

^{1}H NMR (400 MHz, Chloroform-*d*) of compound **5j**

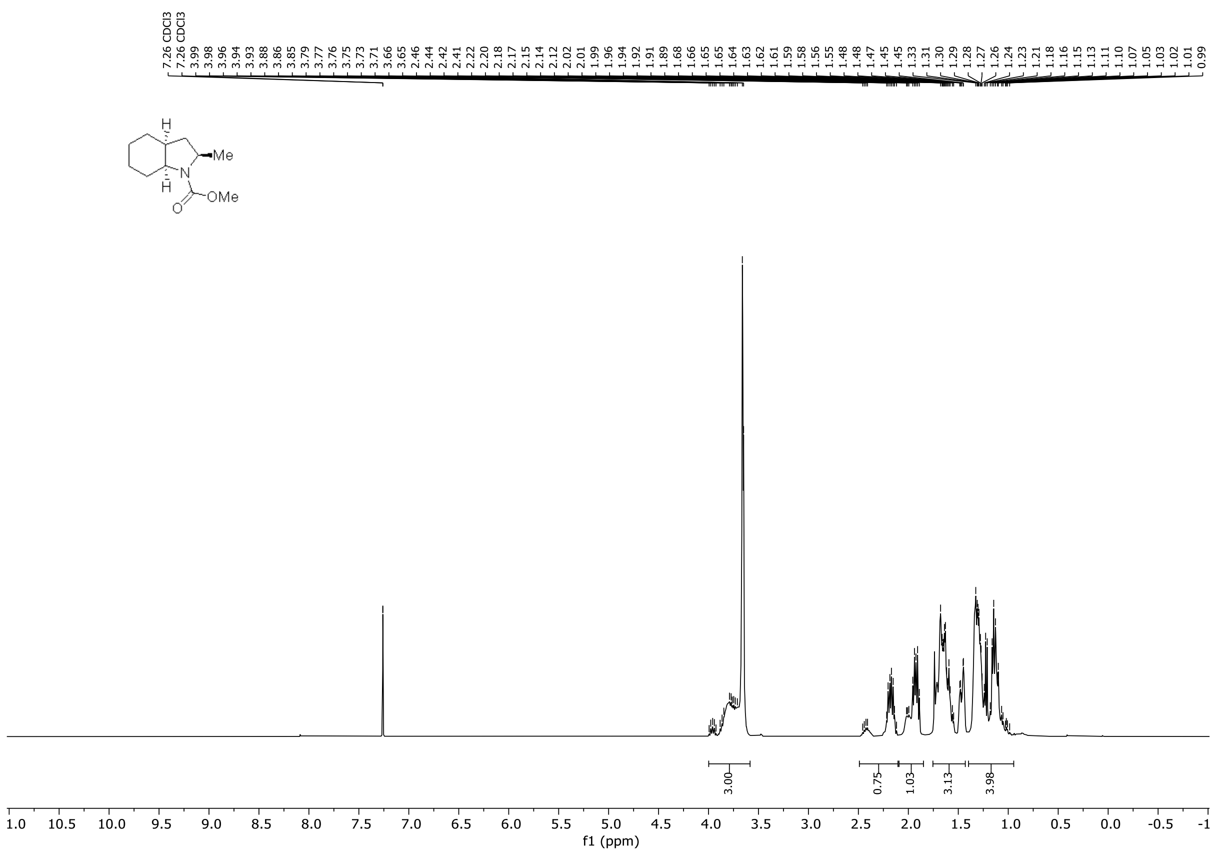

^{13}C NMR (101 MHz, Chloroform-*d*) of compound **5j**

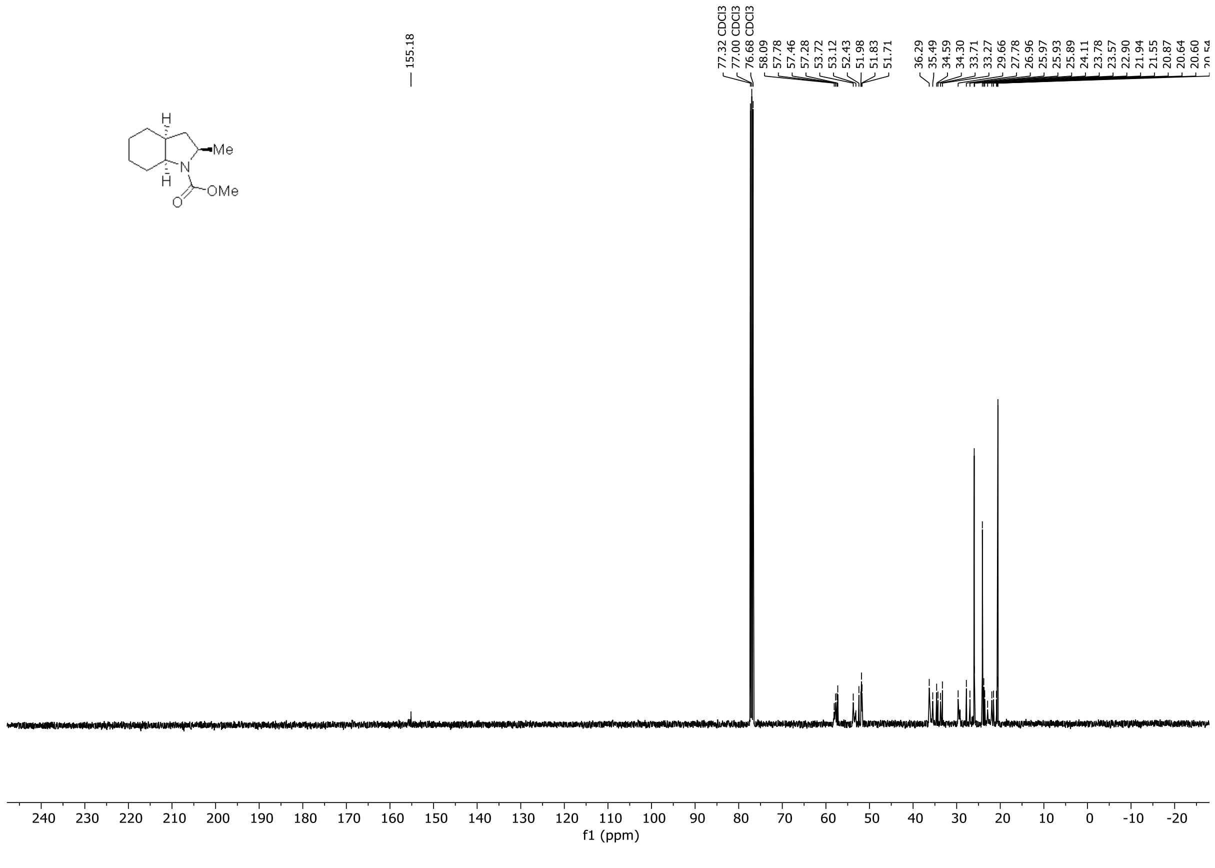

^{1}H NMR (400 MHz, Chloroform-*d*) of compound **5k**

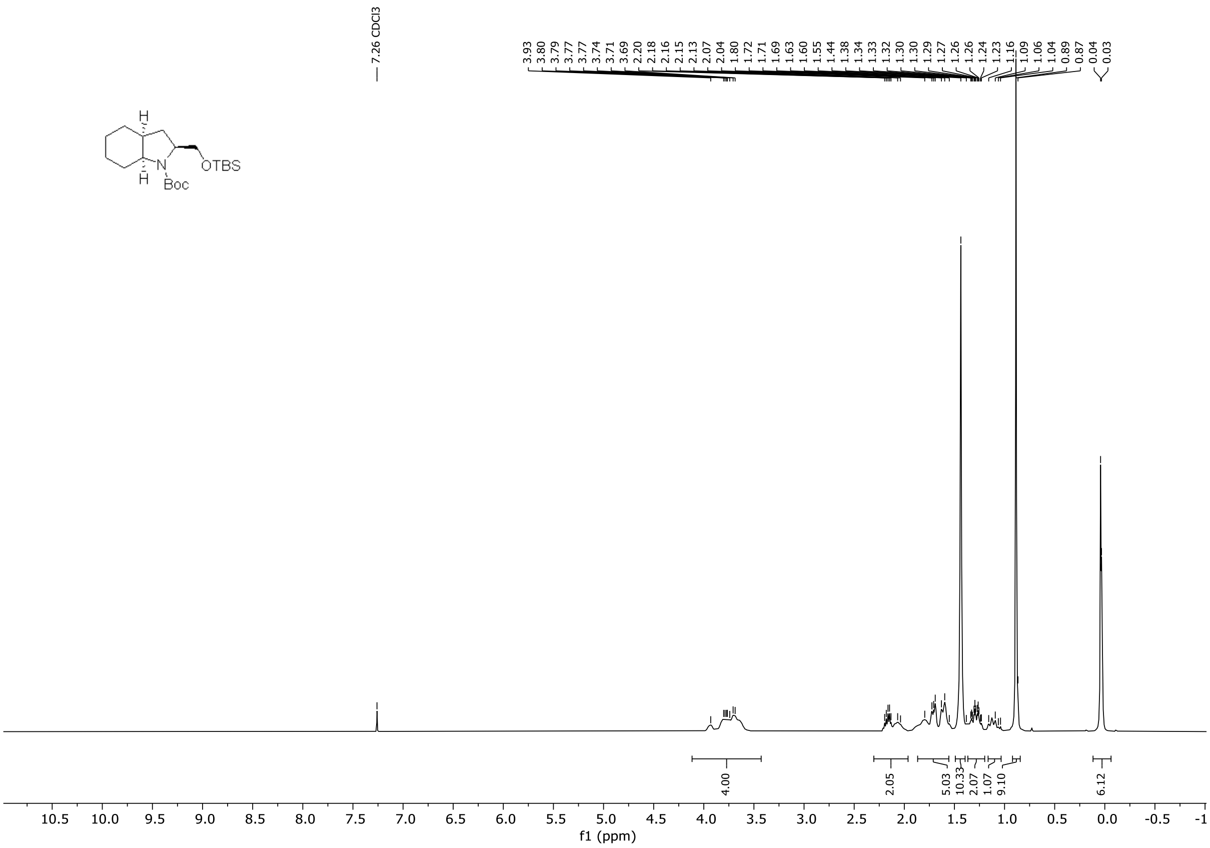

^{13}C NMR (101 MHz, Chloroform-*d*) of compound **5k**

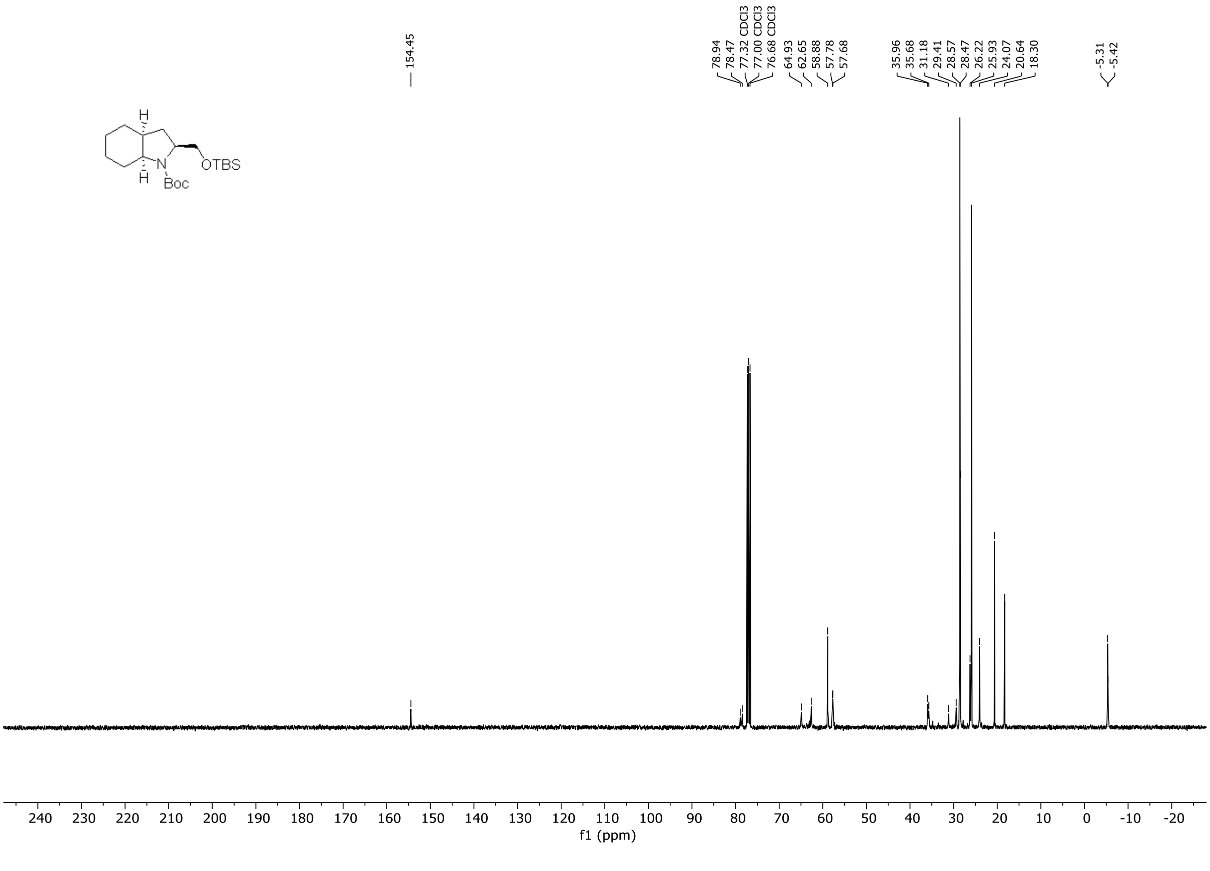

^{1}H NMR (400 MHz, Chloroform-*d*) of compound **7a**

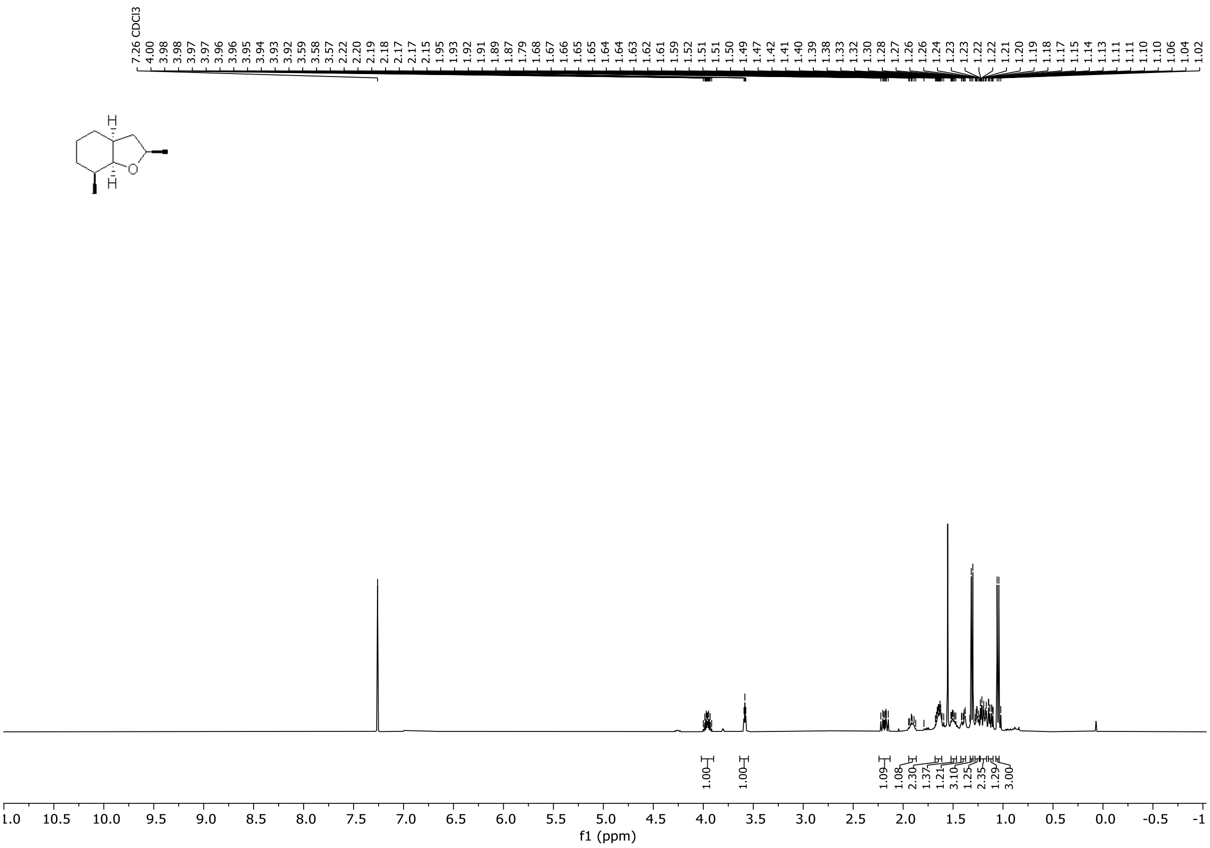

^{13}C NMR (101 MHz, Chloroform-*d*) of compound **7a**

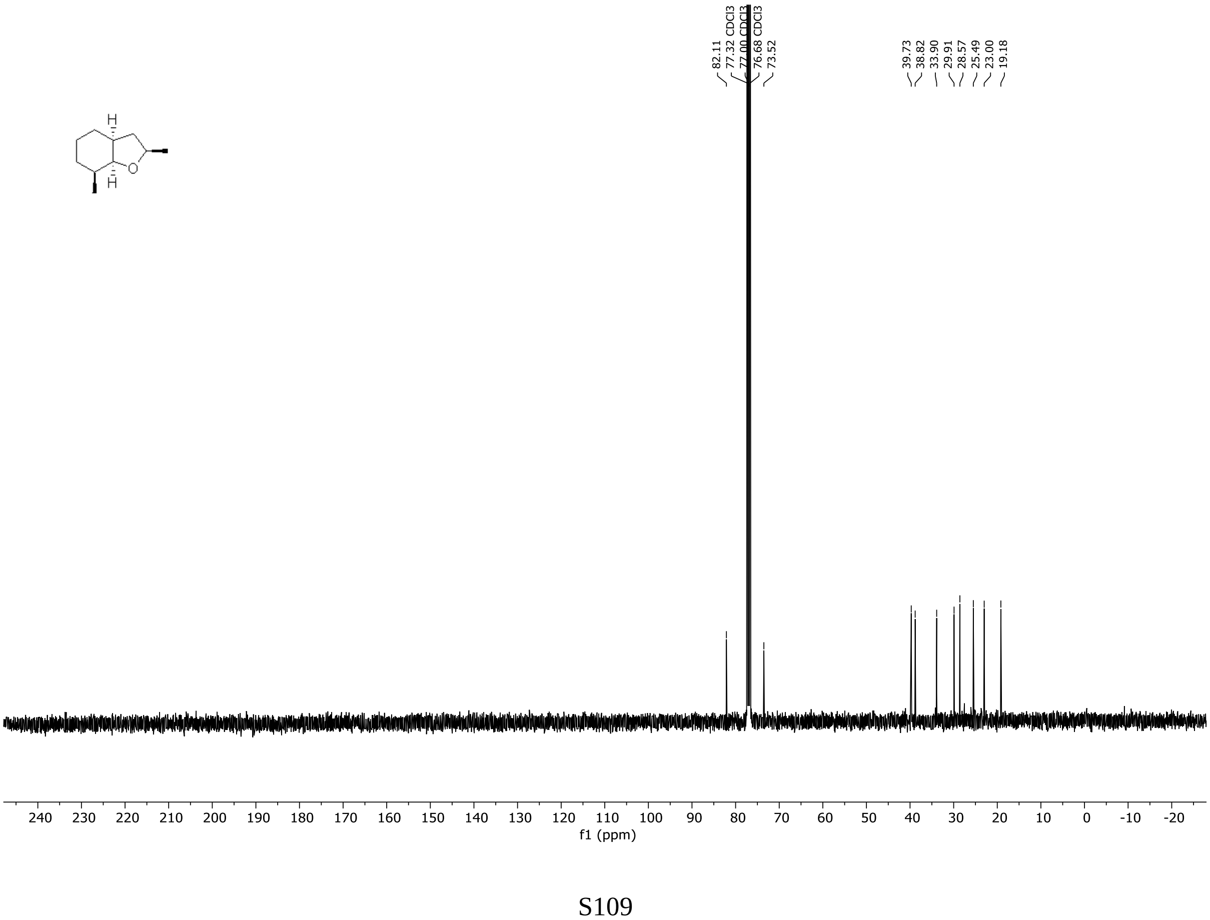

^{1}H NMR (400 MHz, Chloroform-*d*) of compound **7b**

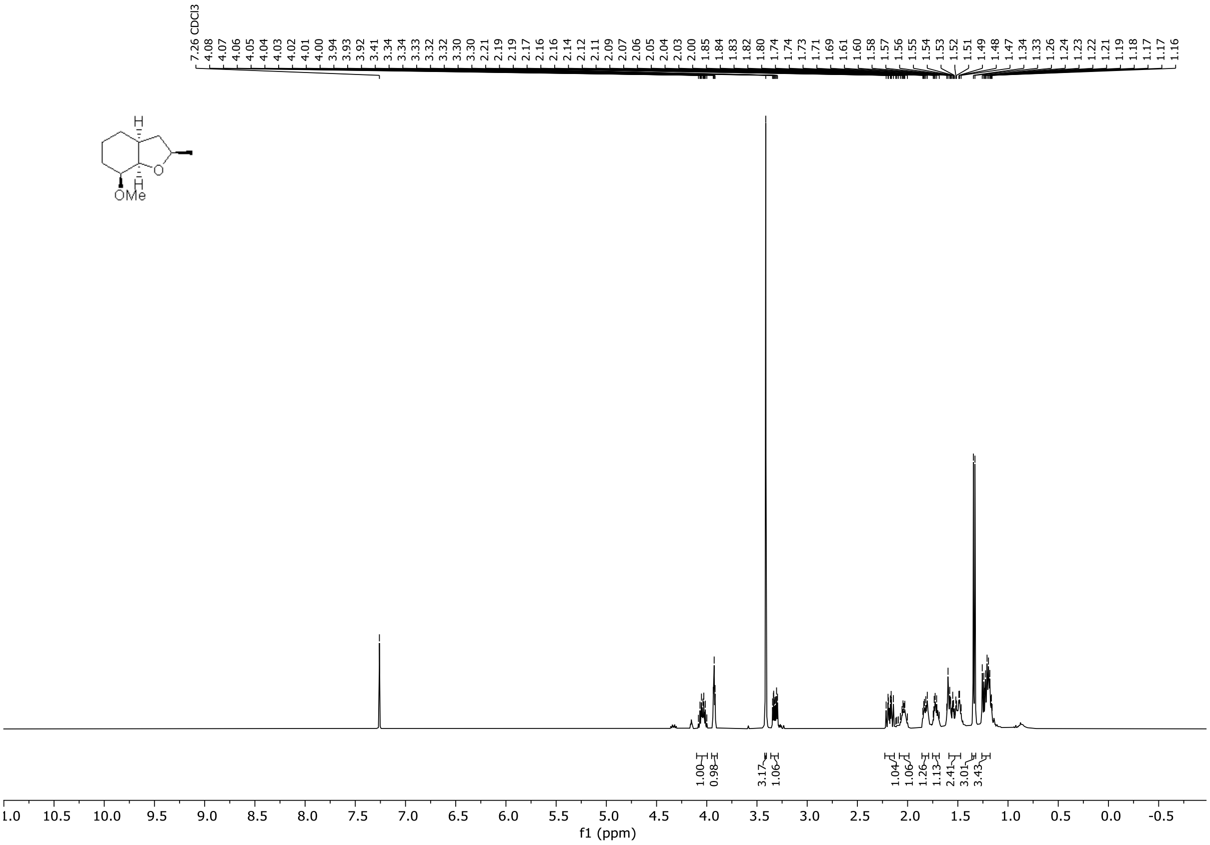

^{13}C NMR (101 MHz, Chloroform-*d*) of compound **7b**

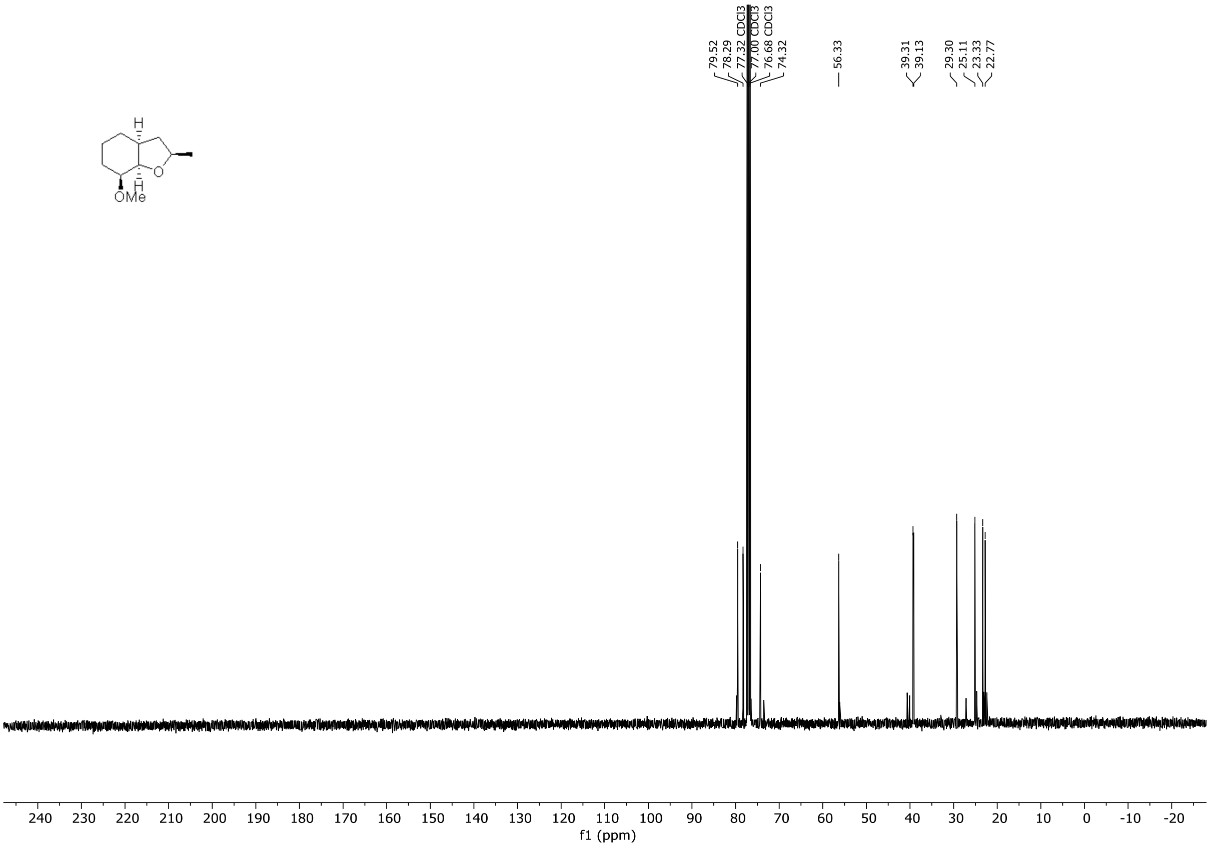

^{1}H NMR (400 MHz, Chloroform-*d*) of compound **7c**

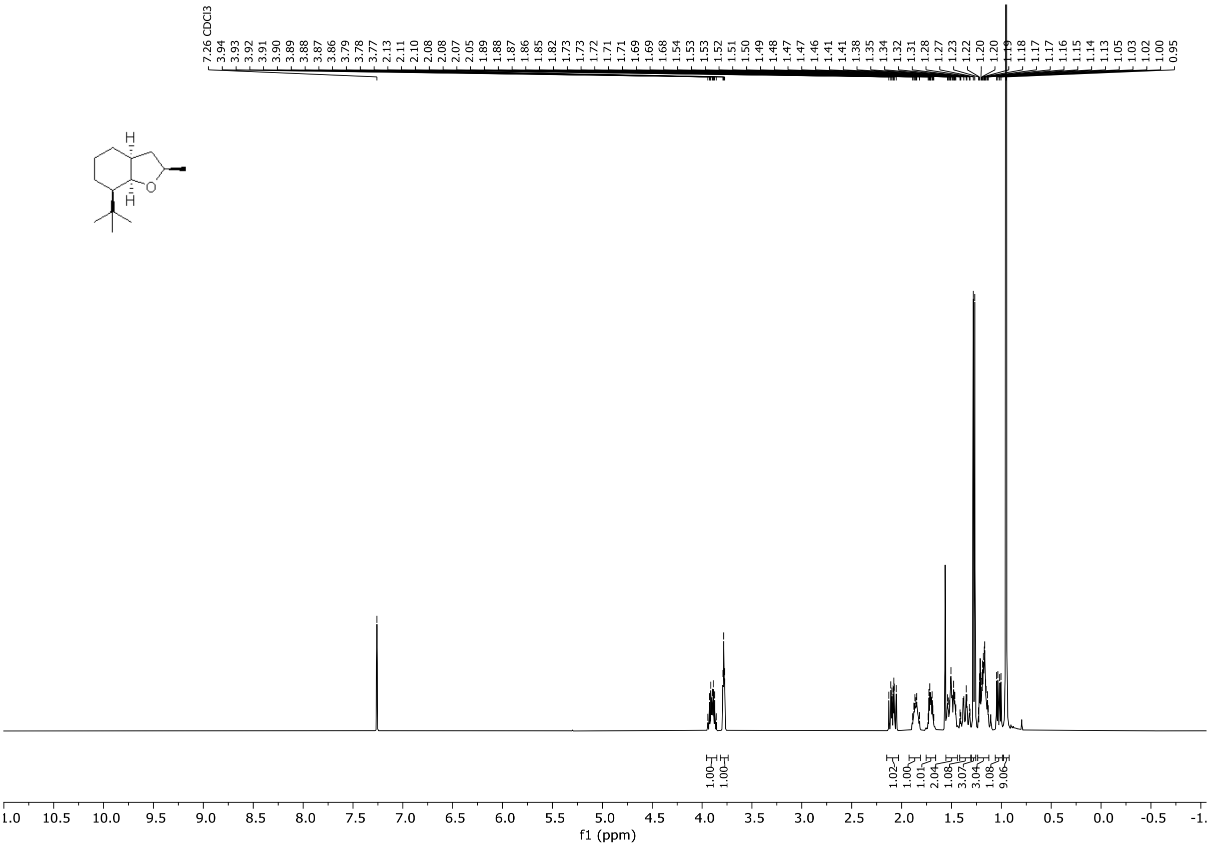

^{13}C NMR (101 MHz, Chloroform-*d*) of compound **7c**

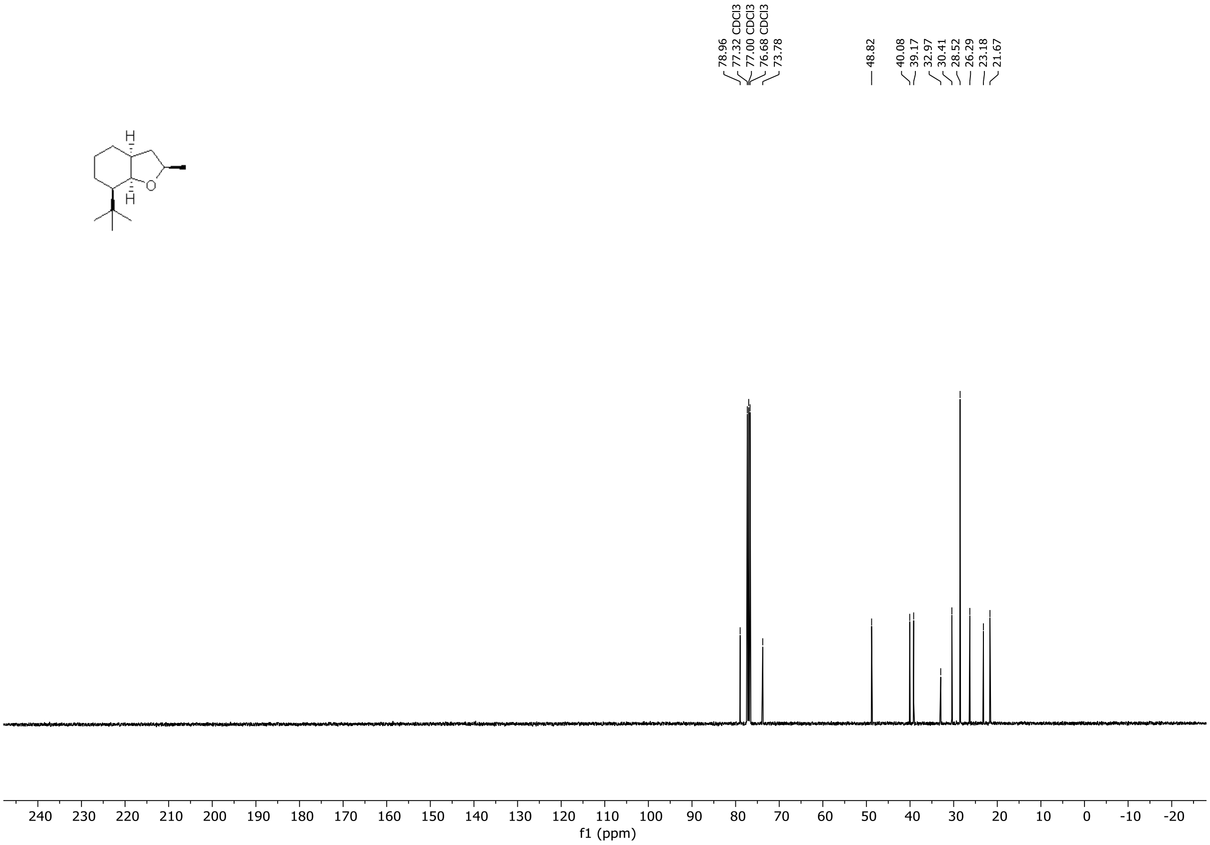

^{1}H NMR (400 MHz, Chloroform-*d*) of compound **7d**

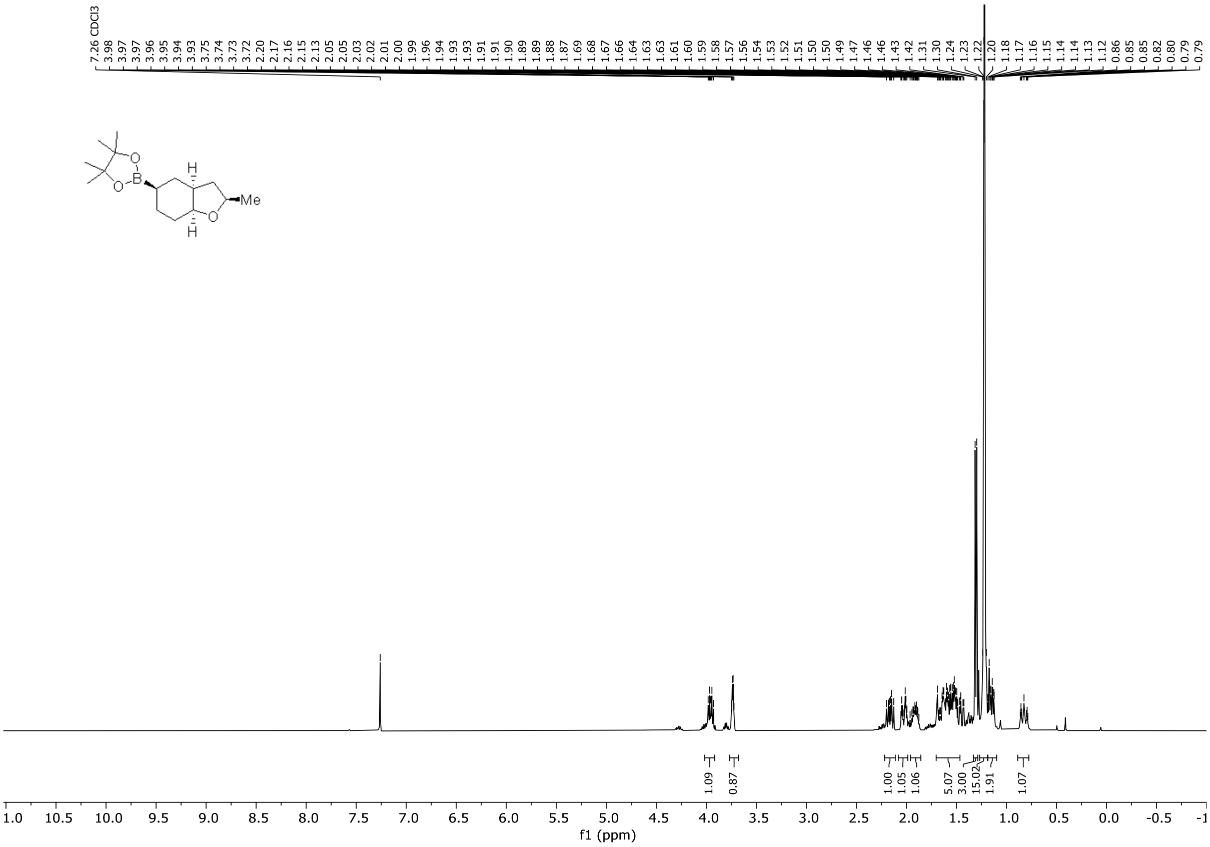

^{13}C NMR (101 MHz, Chloroform-*d*) of compound **7d**

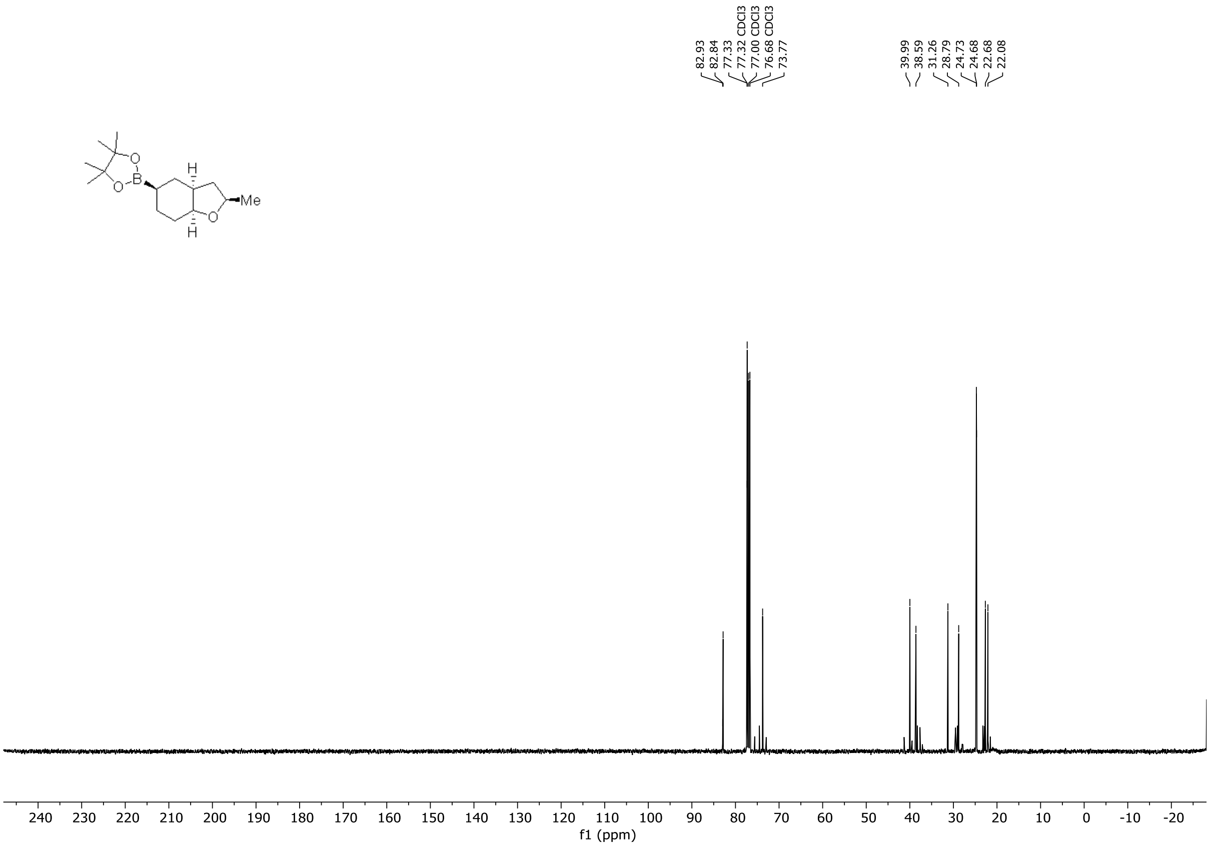

^{11}B NMR (128 MHz, Chloroform-*d*) of compound **7d**

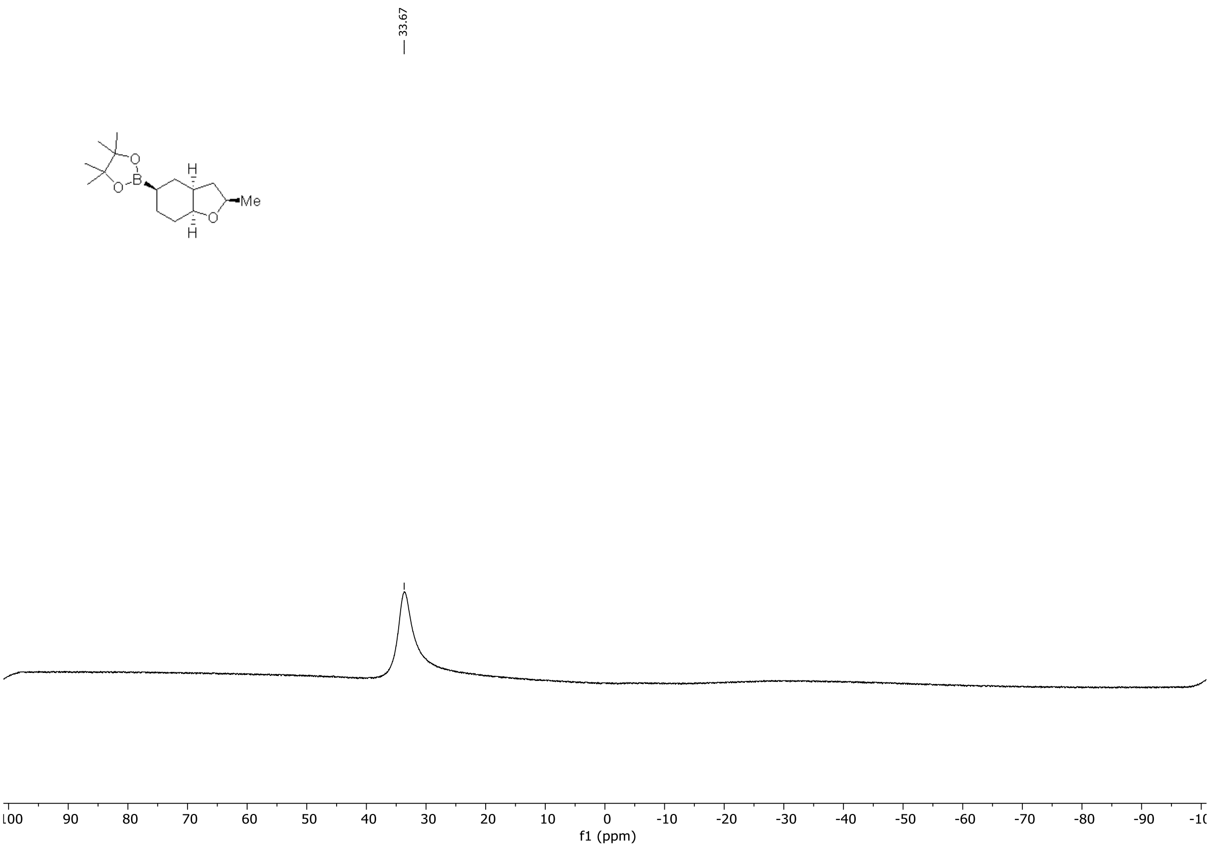

^{1}H NMR (400 MHz, Chloroform-*d*) of compound **7e**

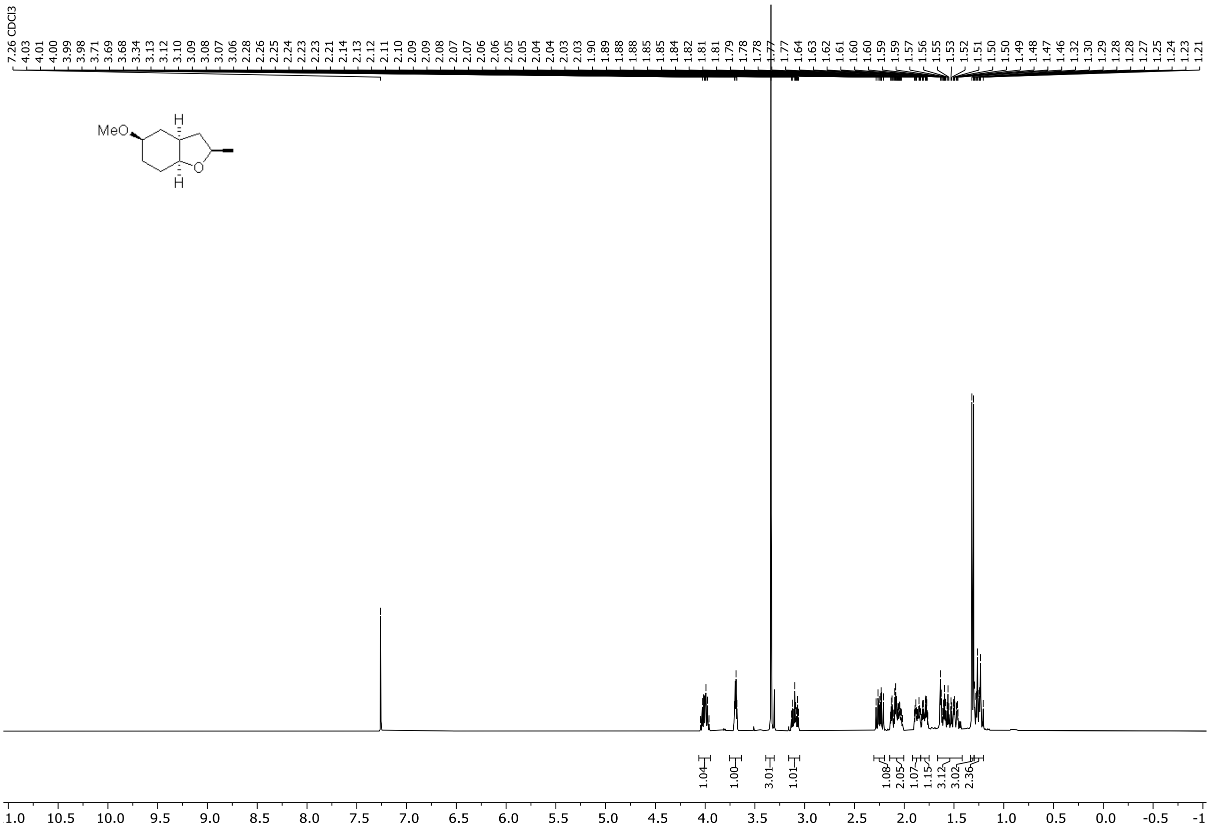

^{13}C NMR (101 MHz, Chloroform-*d*) of compound **7e**

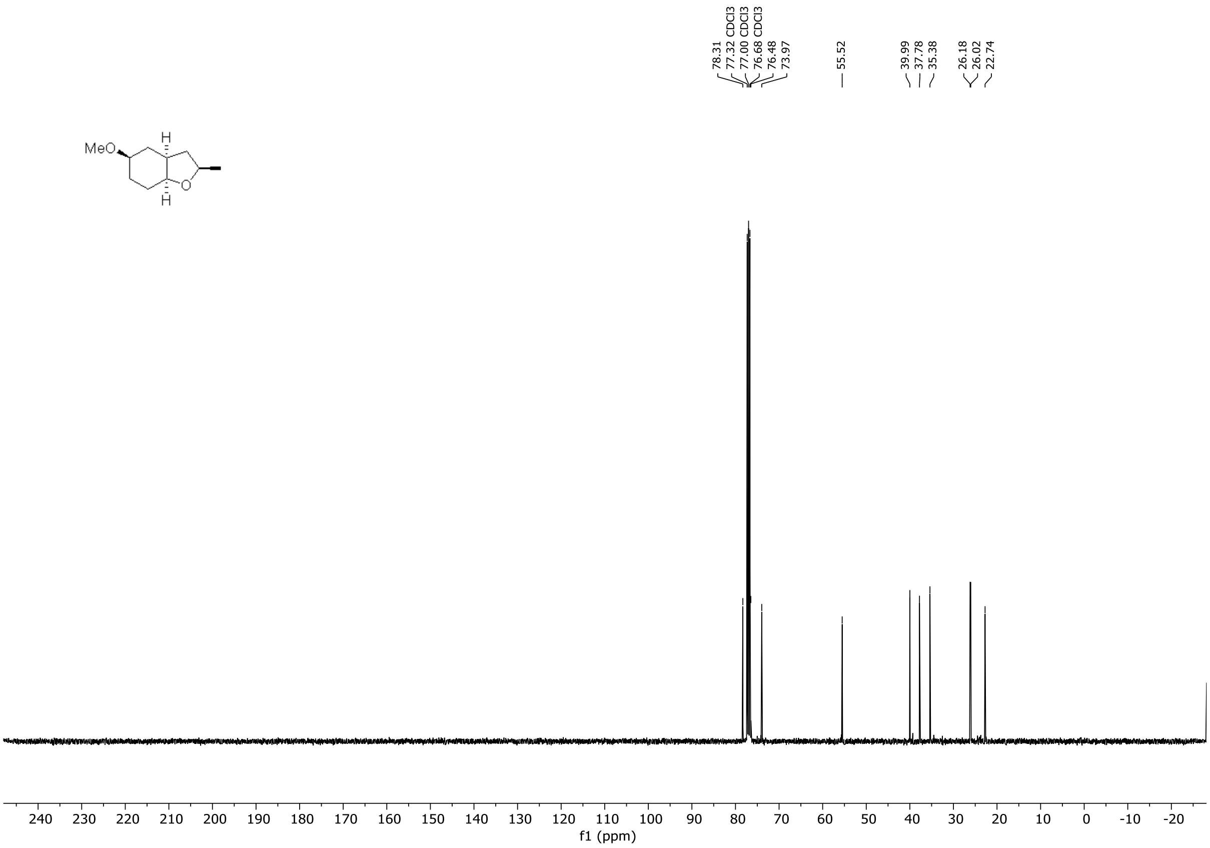

^{1}H NMR (400 MHz, Chloroform-*d*) of compound **7f**

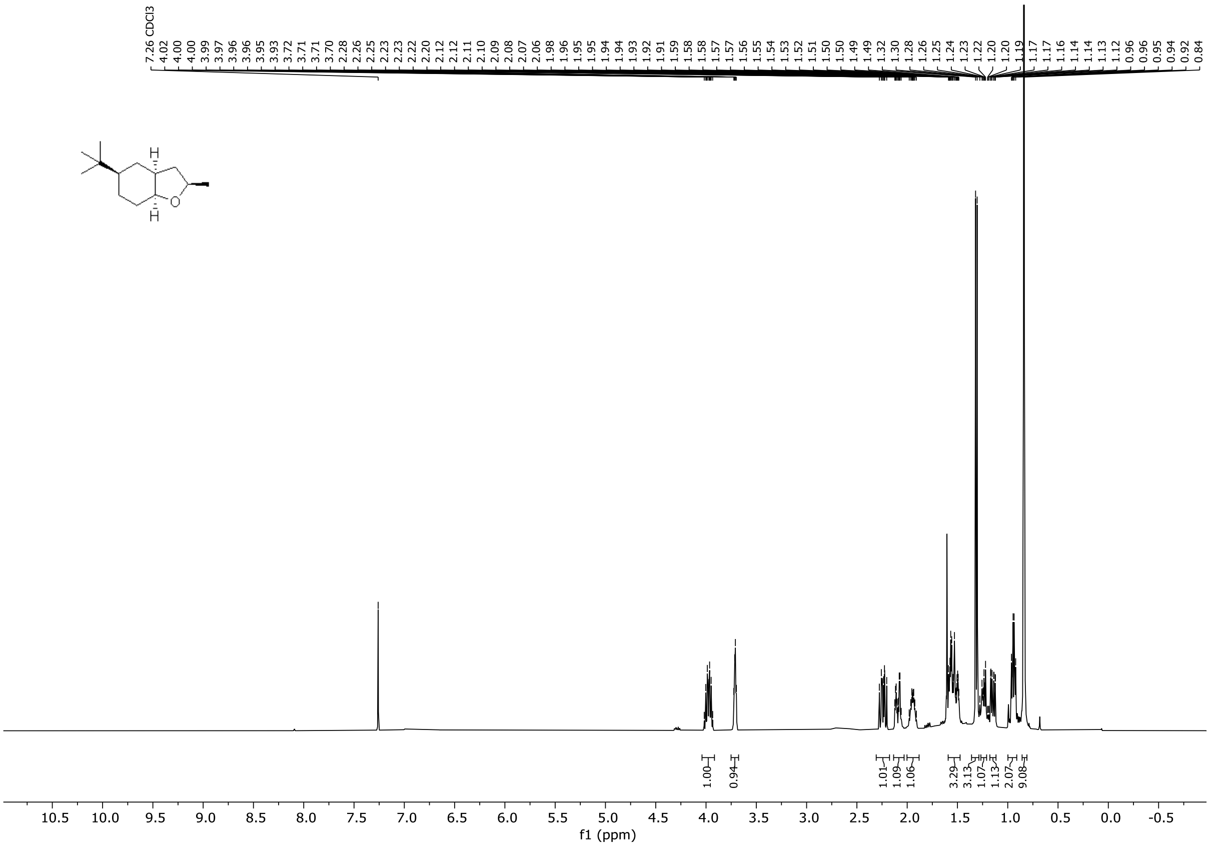

^{13}C NMR (101 MHz, Chloroform-*d*) of compound **7f**

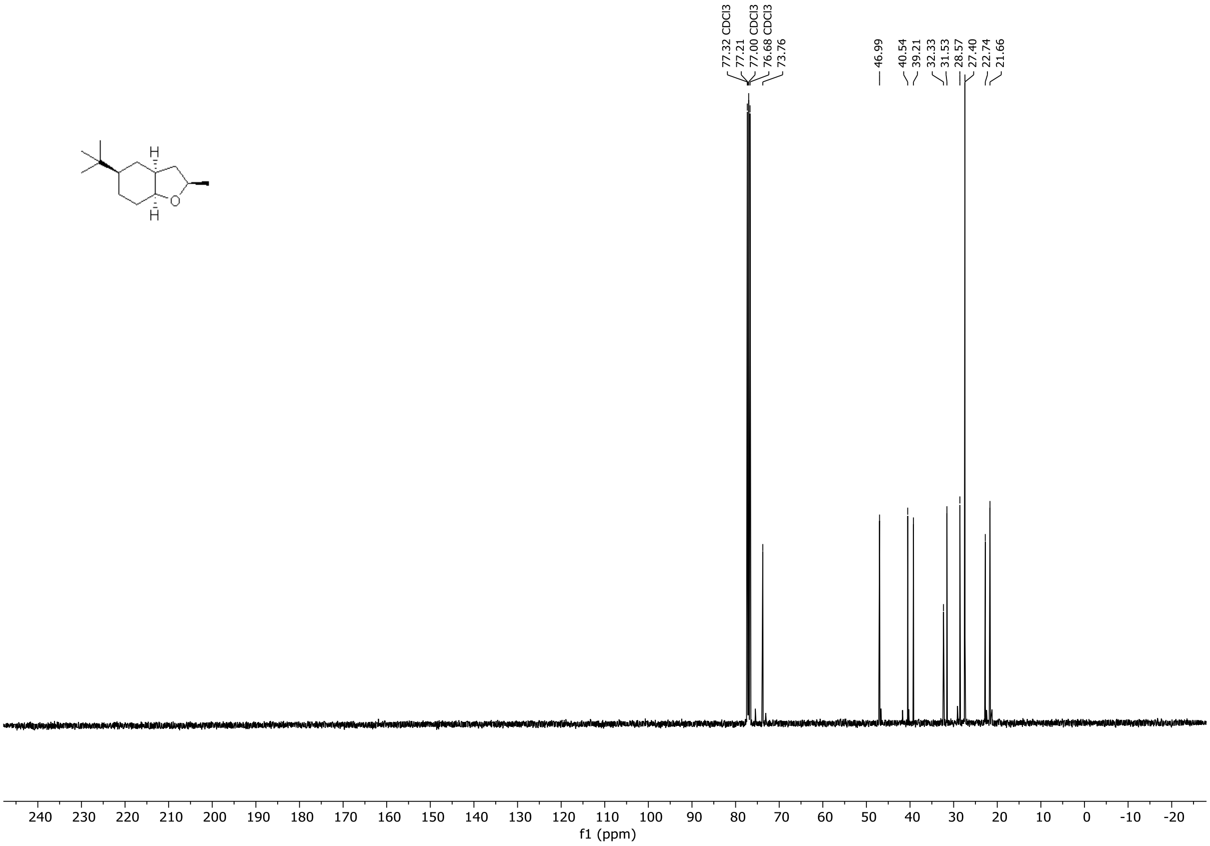

^{1}H NMR (400 MHz, Chloroform-*d*) of compound **7g**

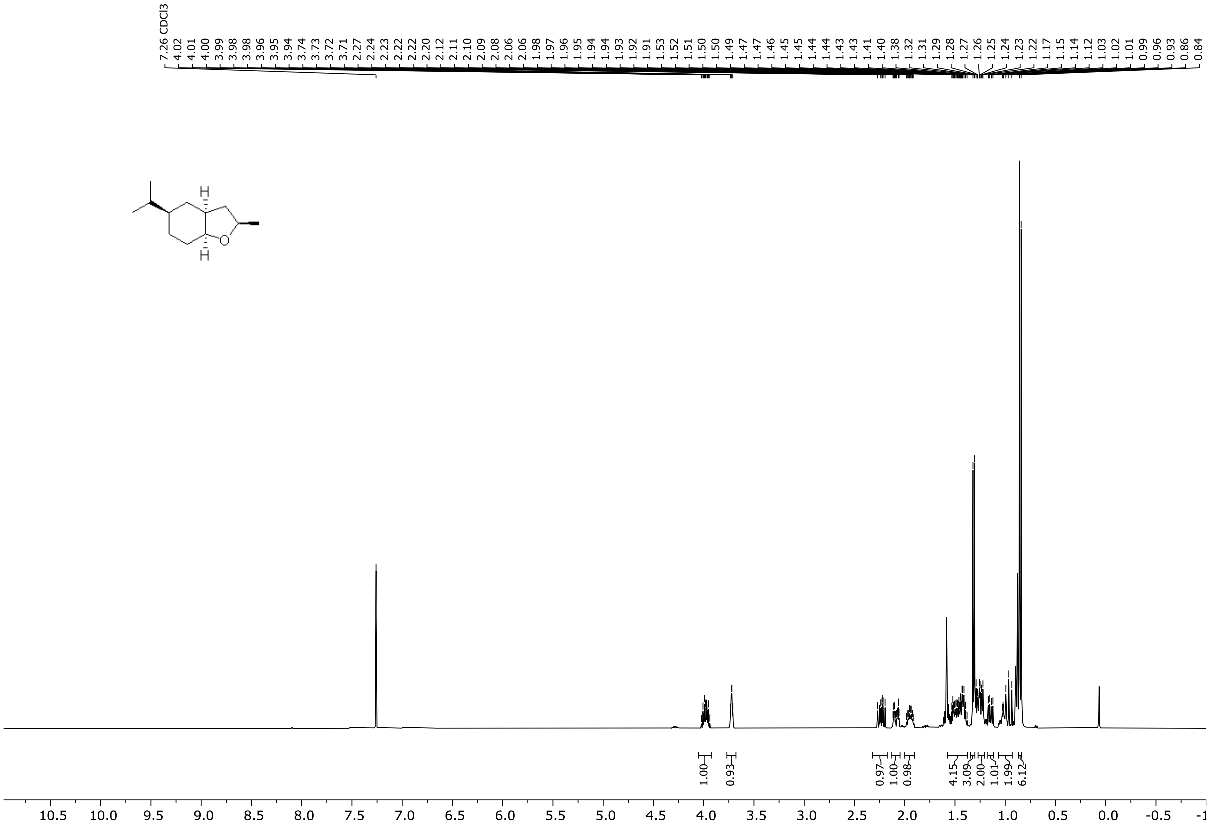

^{13}C NMR (101 MHz, Chloroform-*d*) of compound **7g**

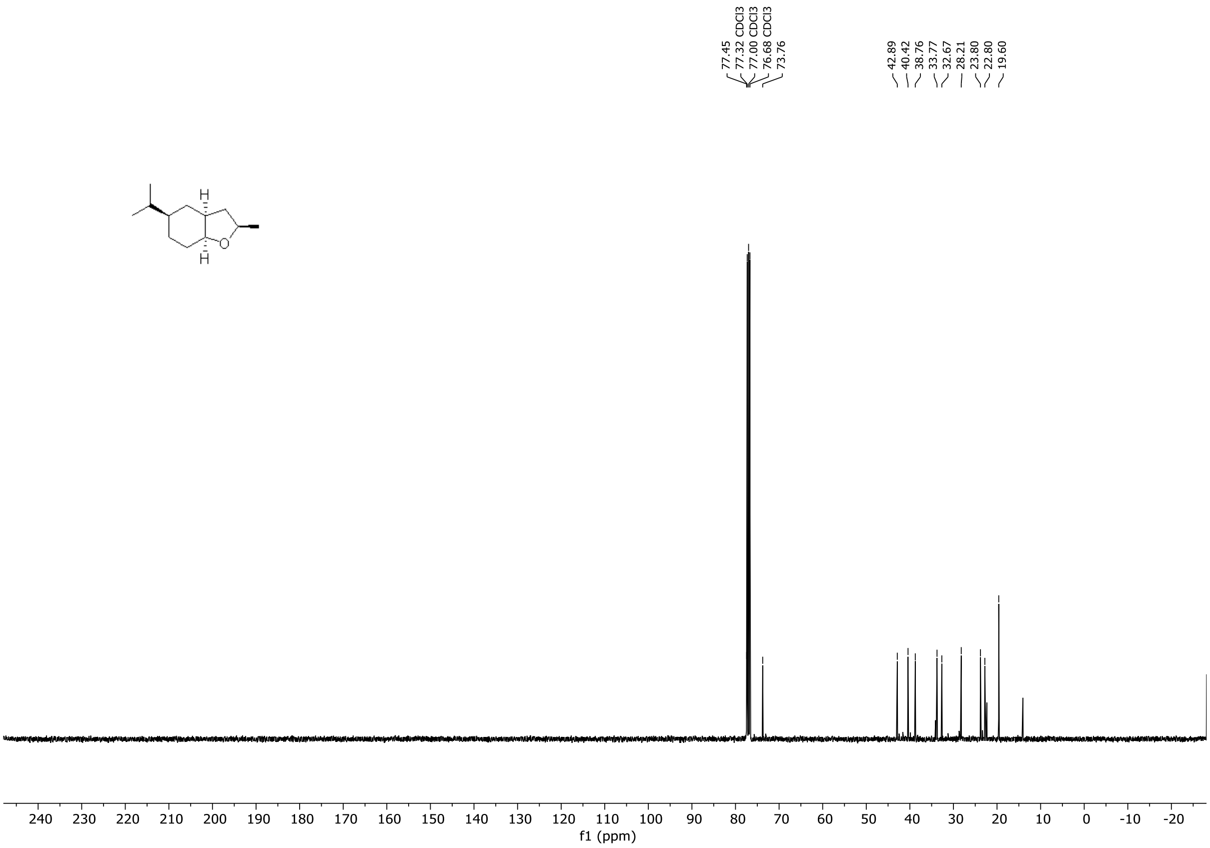

^{1}H NMR (400 MHz, Chloroform-*d*) of compound **7h**

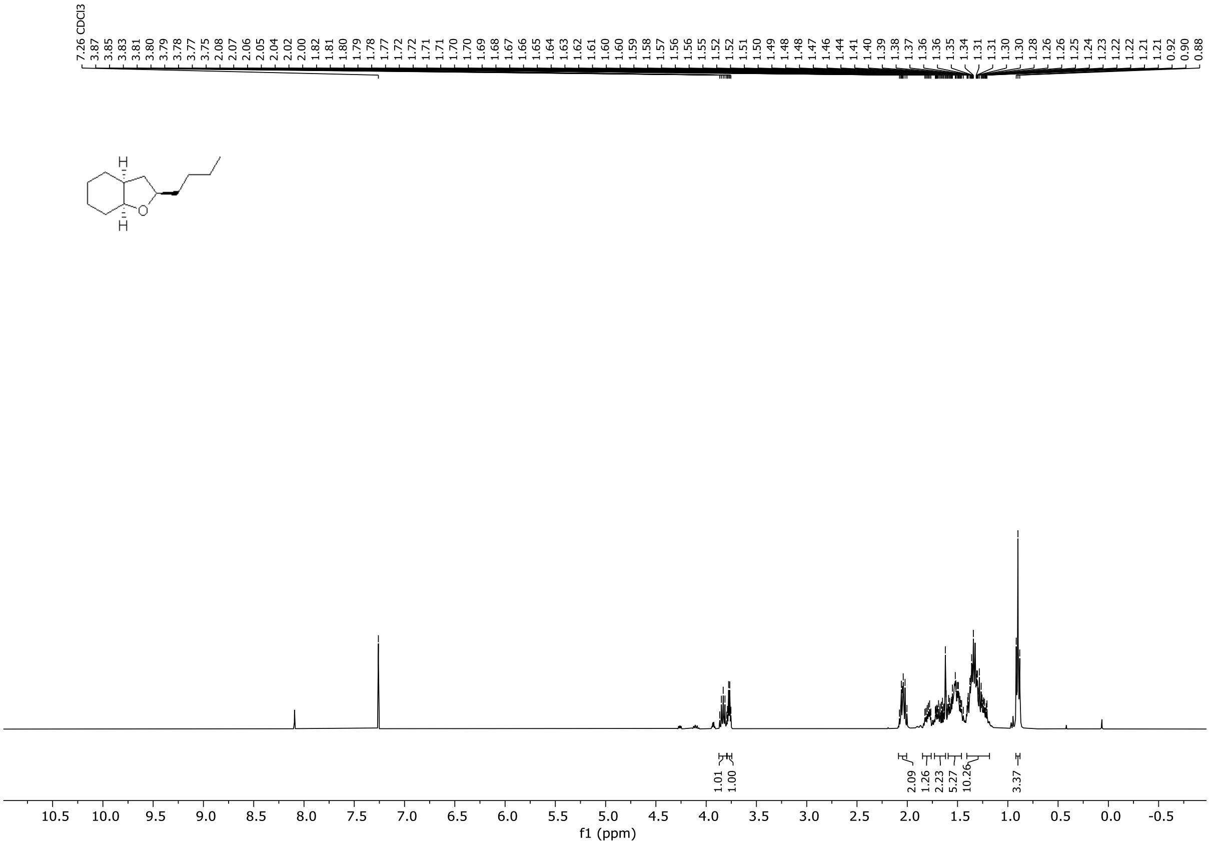

^{13}C NMR (101 MHz, Chloroform-*d*) of compound **7h**

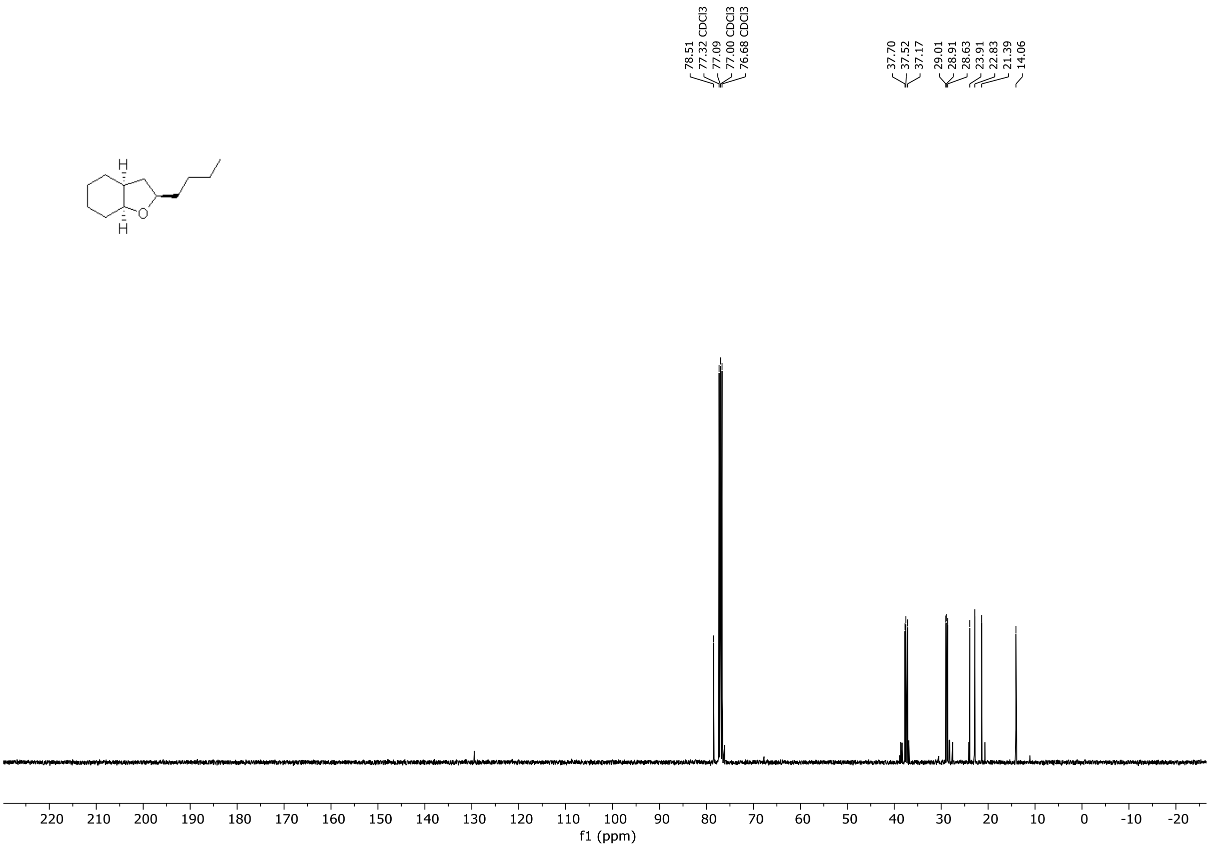

^{1}H NMR (400 MHz, Chloroform-*d*) of compound **2aa**

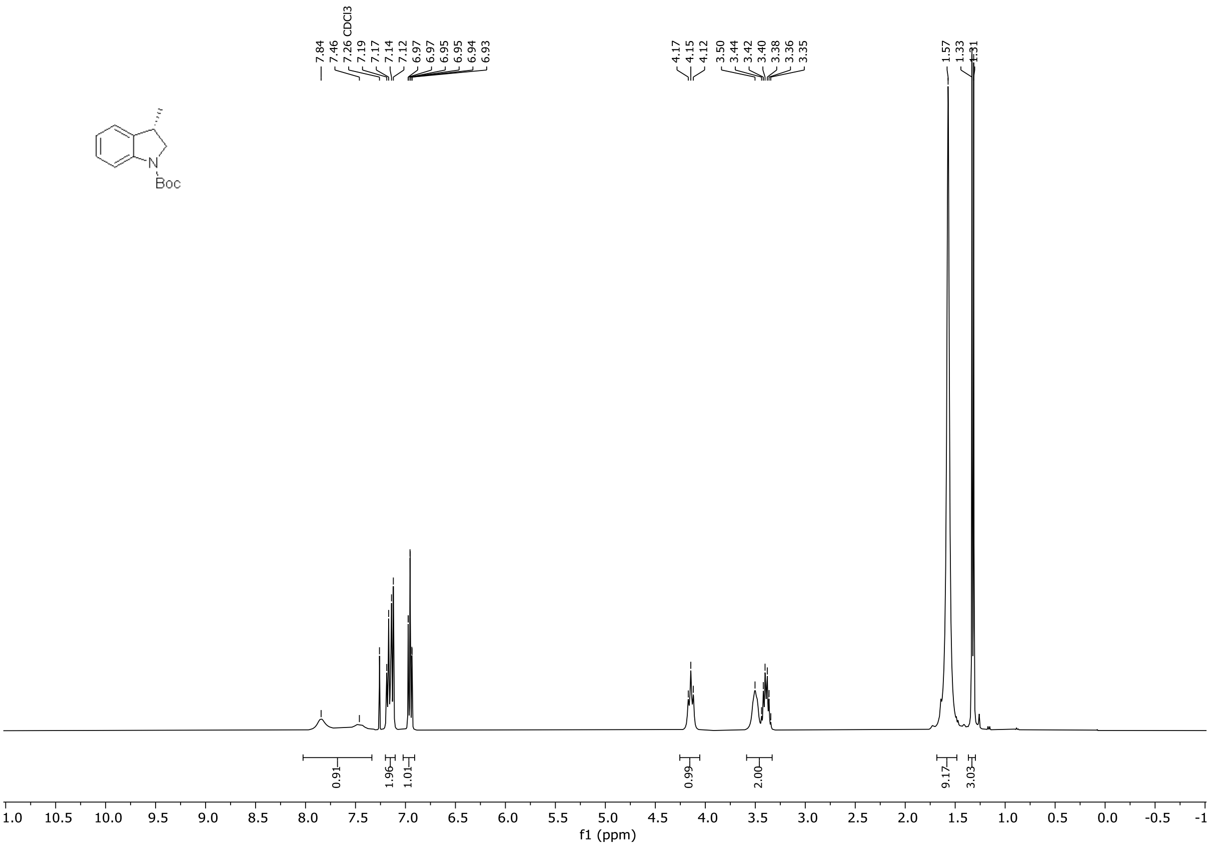

^{13}C NMR (101 MHz, Chloroform-*d*) of compound **2aa**

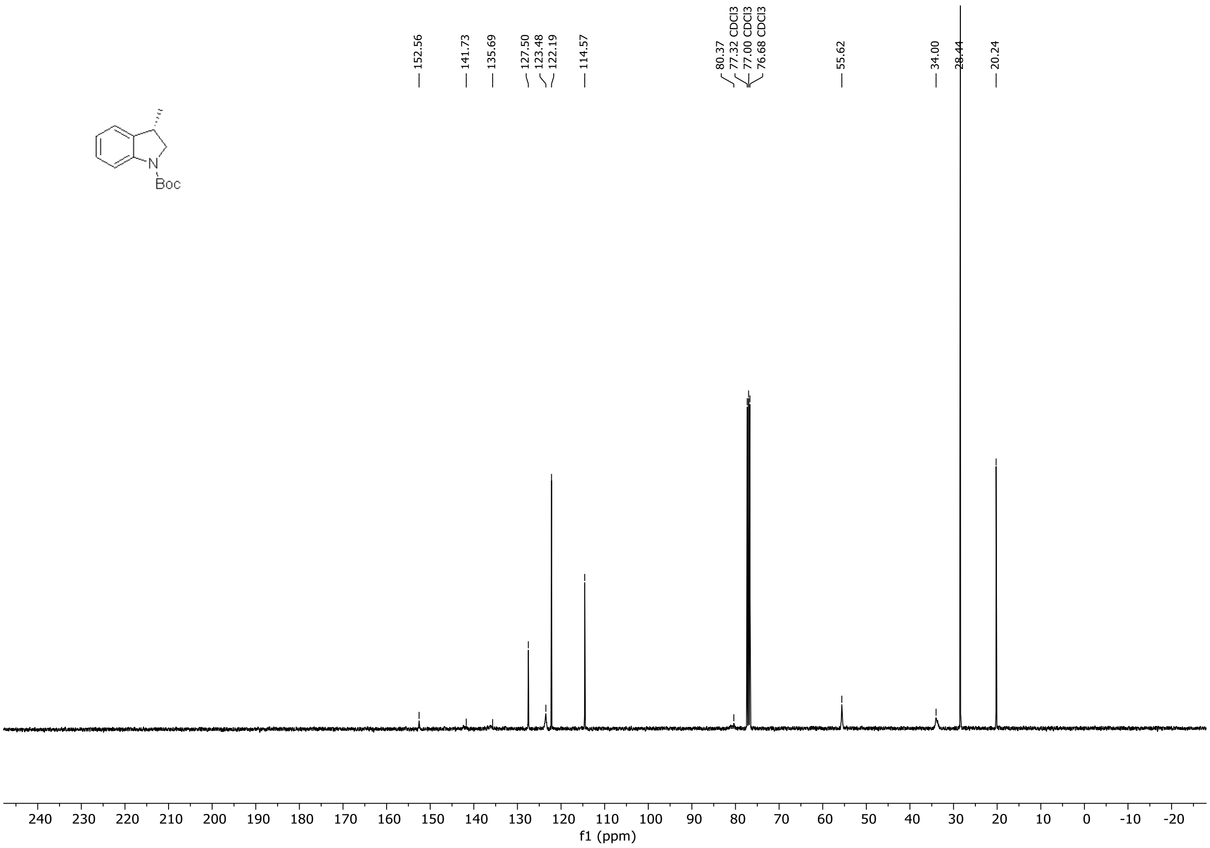

^{1}H NMR (400 MHz, Chloroform-*d*) of compound **5aa**

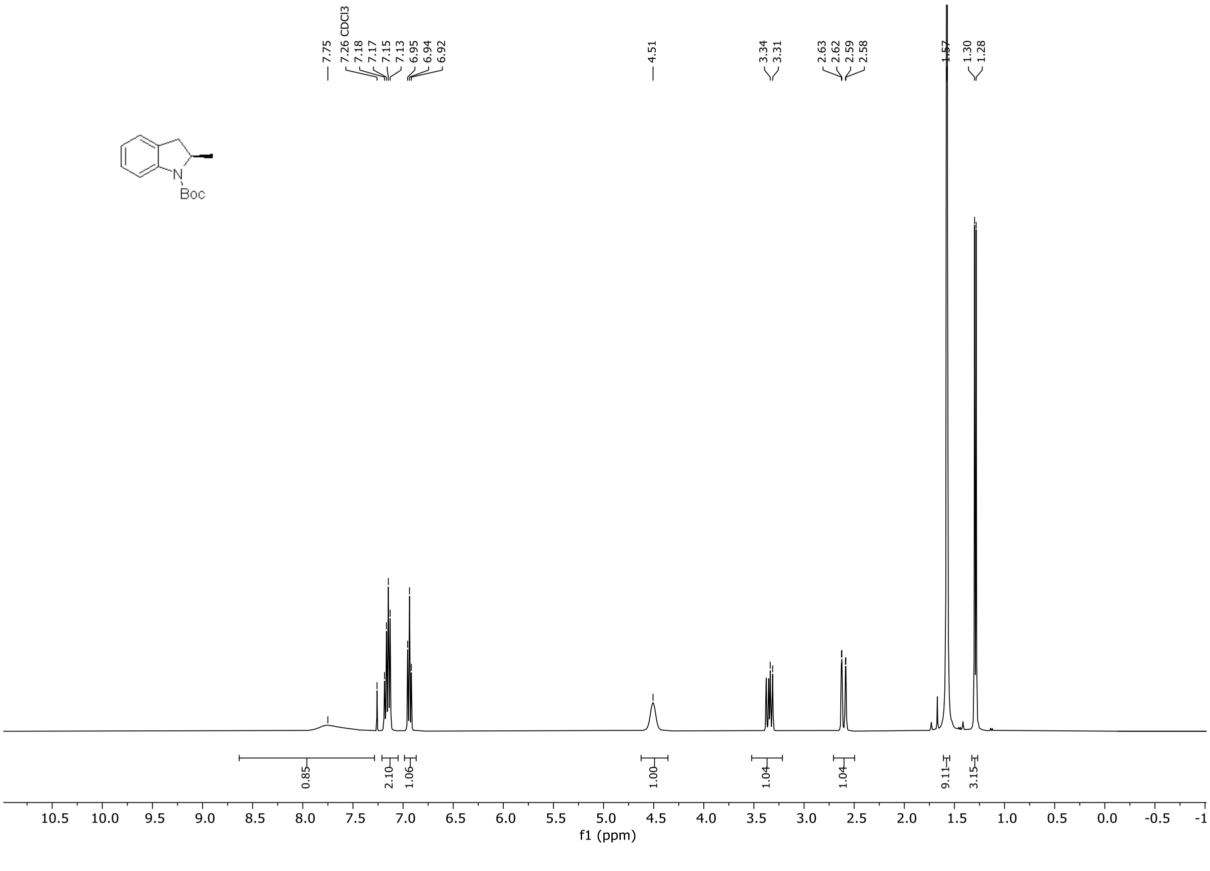

^{13}C NMR (101 MHz, Chloroform-*d*) of compound **5aa**

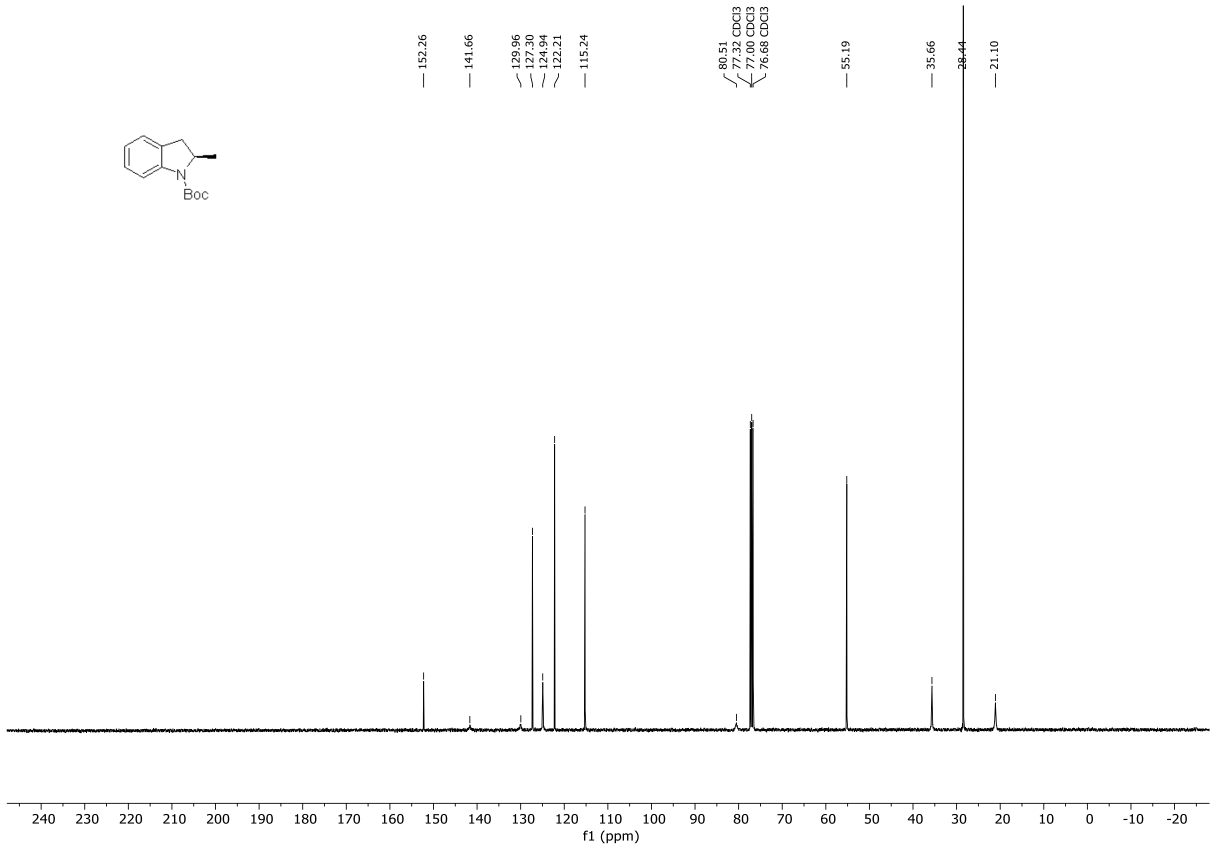

^{1}H NMR (400 MHz, Chloroform-*d*) of compound **8c**

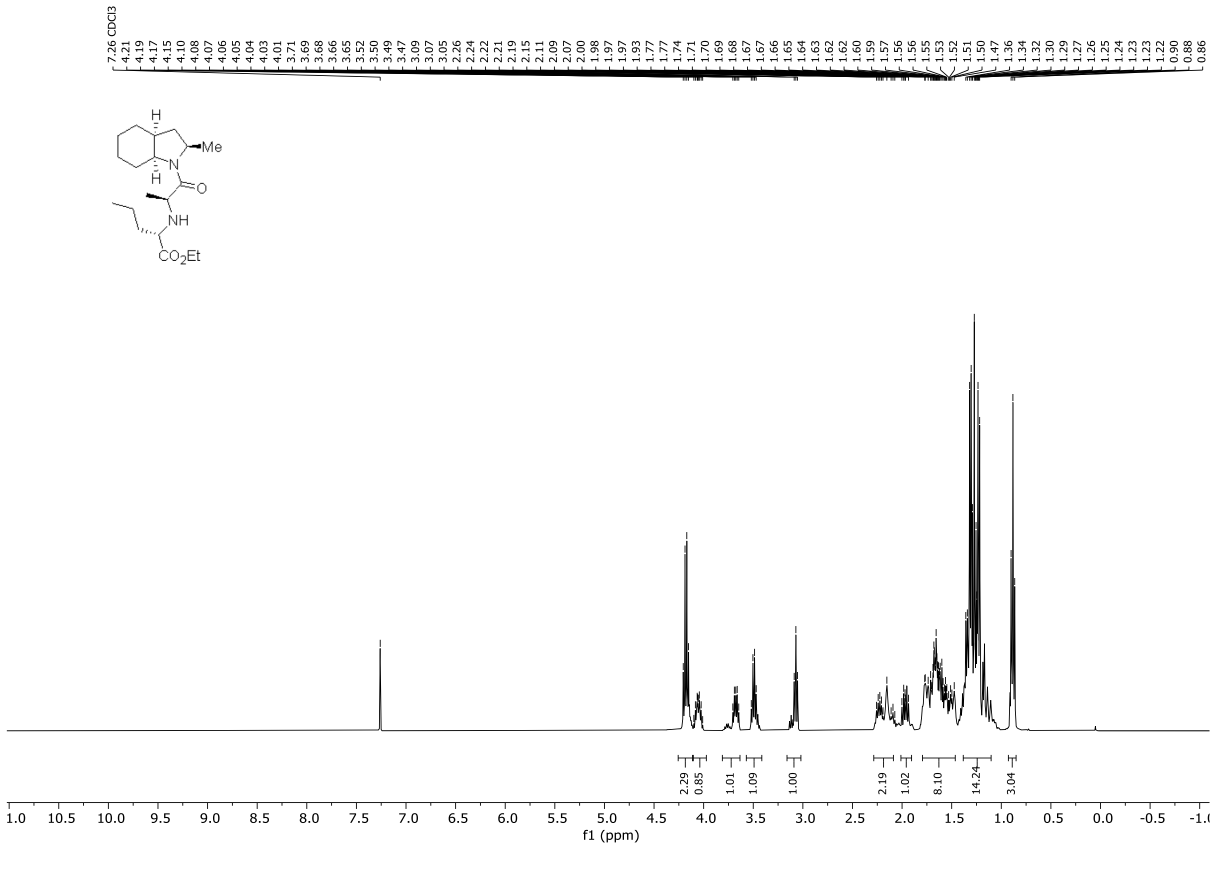

^{13}C NMR (101 MHz, Chloroform-*d*) of compound **8c**

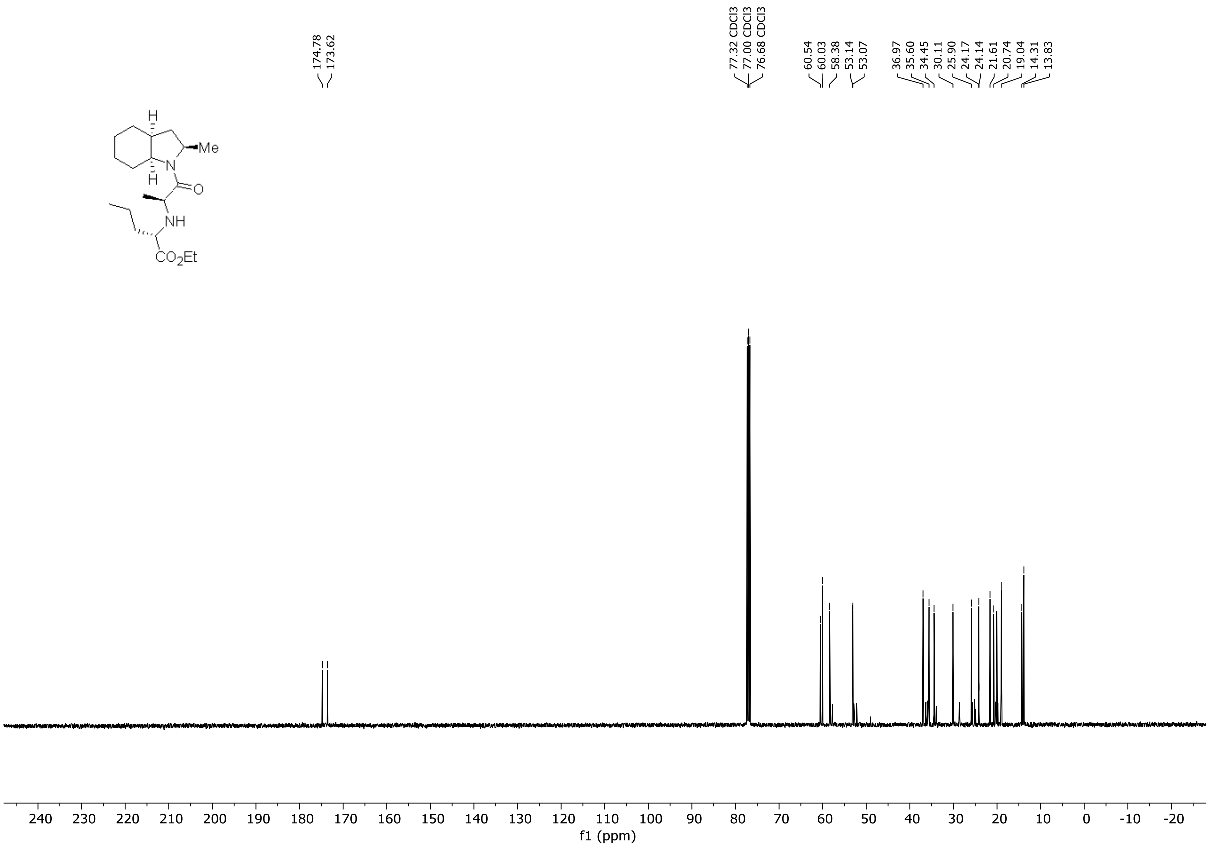

^{1}H NMR (400 MHz, Chloroform-*d*) of compound **9a**

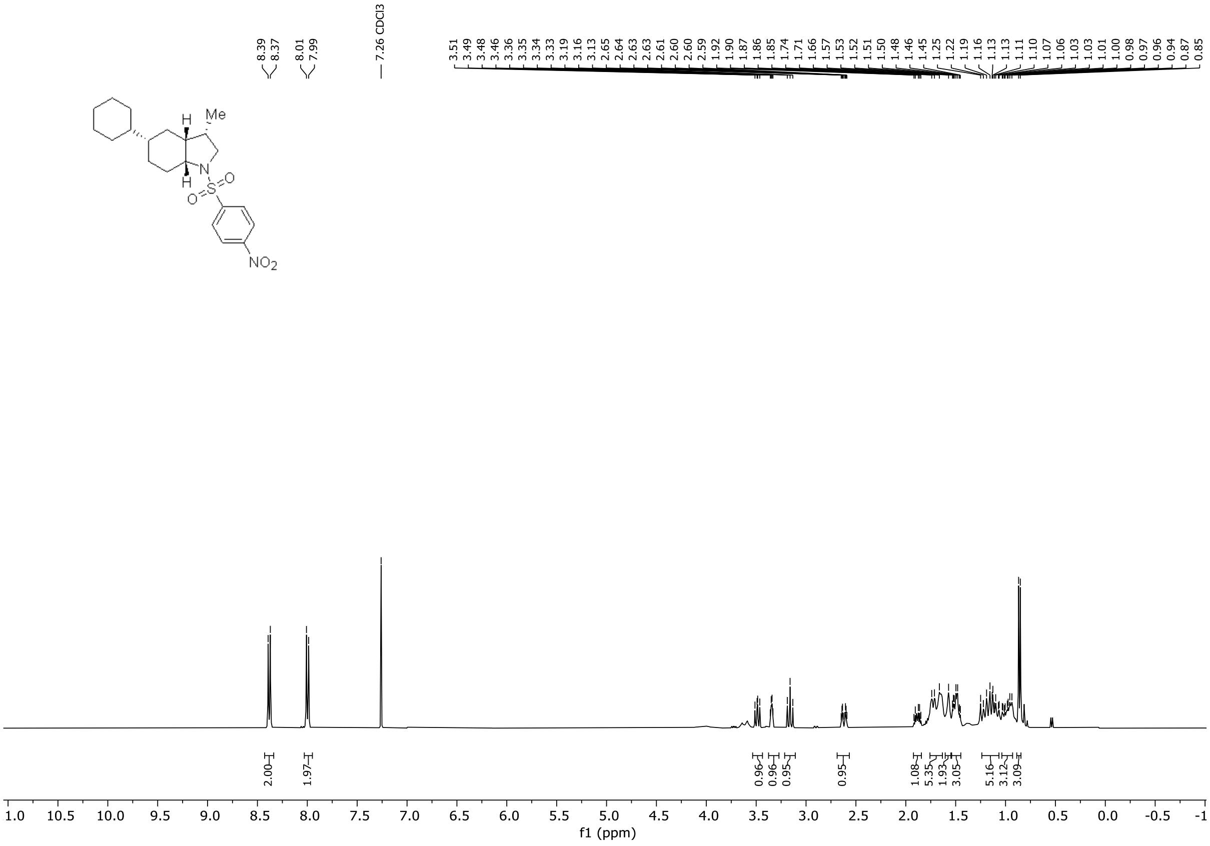

^{13}C NMR (101 MHz, Chloroform-*d*) of compound **9a**

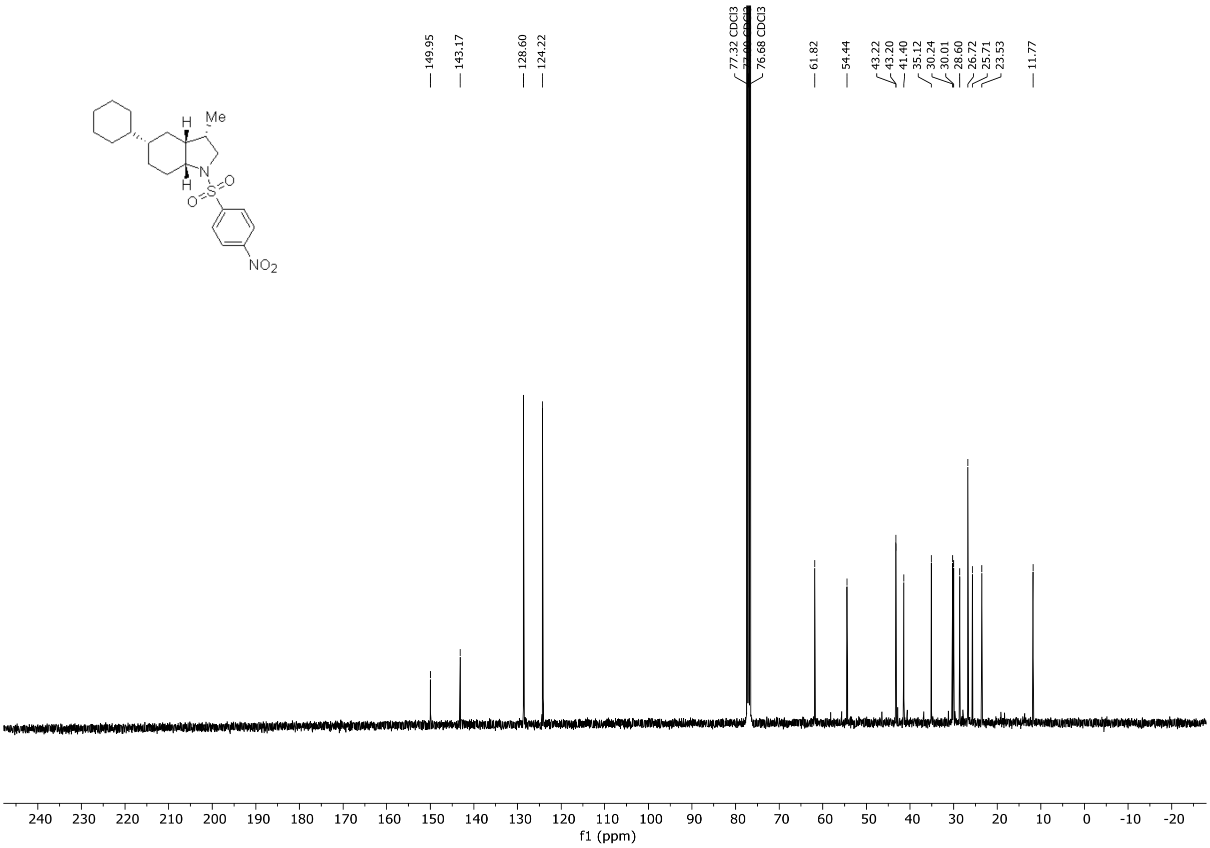

^{1}H NMR (400 MHz, Chloroform-*d*) of compound **10d**

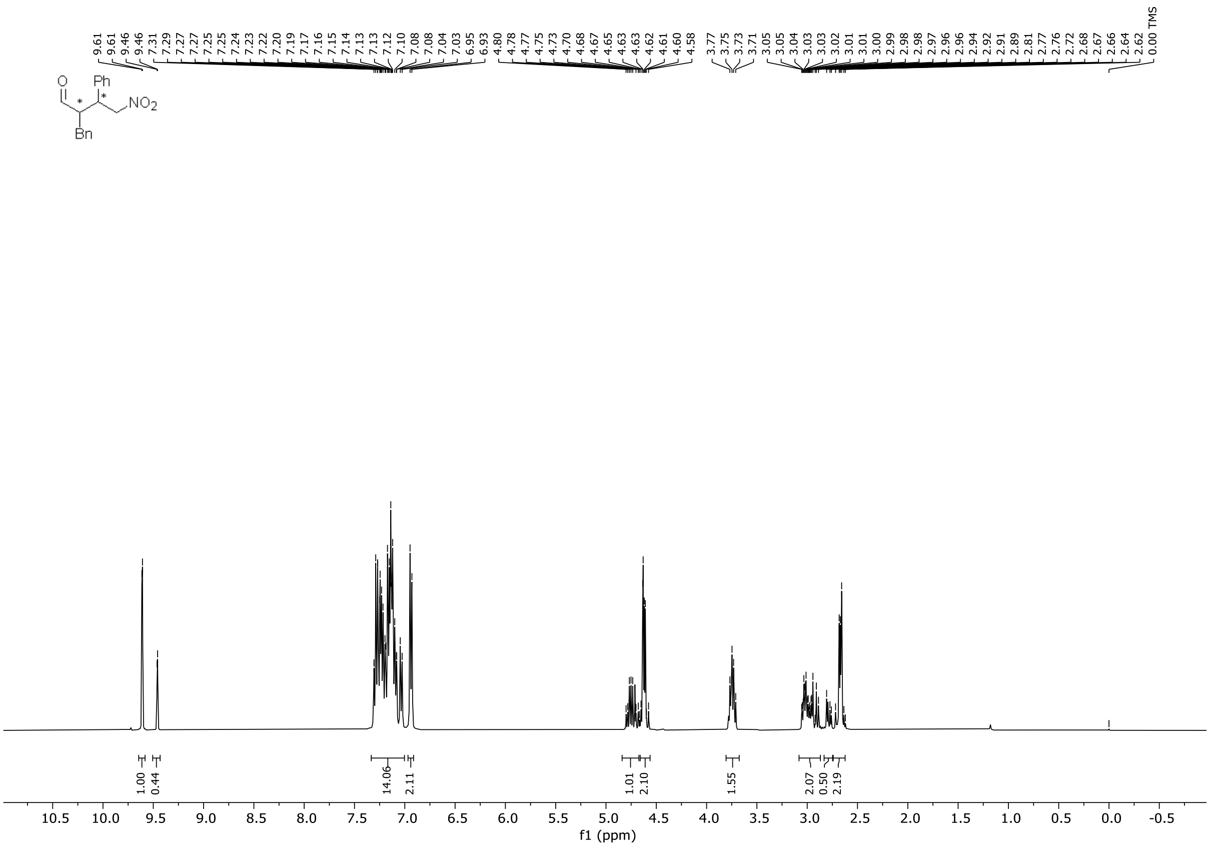

^{13}C NMR (101 MHz, Chloroform-*d*) of compound **10d**

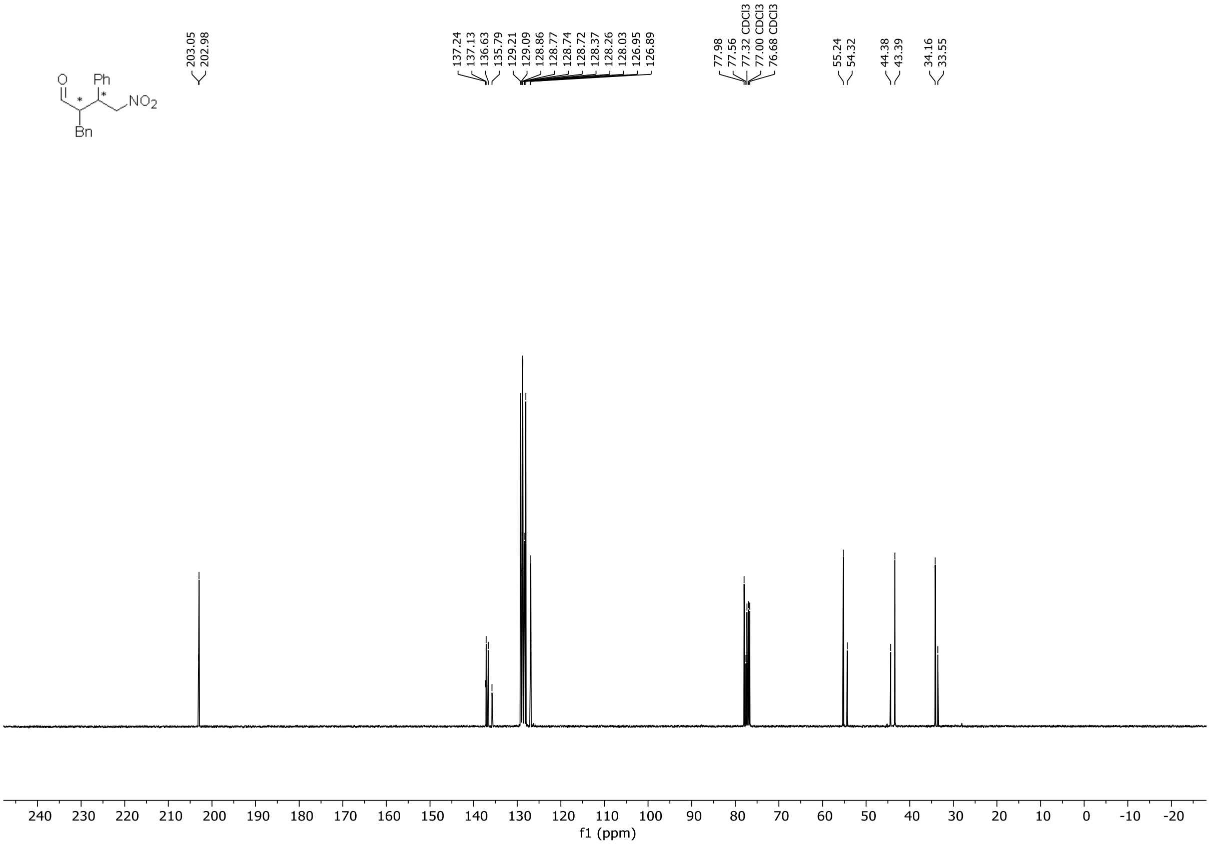

15. References

[1] M. P. Wiesenfeldt, D. Moock, D. Paul, F. Glorius, *Chem. Sci.* **2021**, *12*, 5611-5615.

[2] a) T. Krüger, S. Weiland, G. Falck, M. Gerlach, M. Boschanski, S. Alam, K. M. Müller, T. Dierks, N. Sewald, *Angew. Chem., Int. Ed.* **2018**, *57*, 7245-7249; b) A. J. Smith, D. Dimitrova, J. N. Arokianathar, K. Kolodziejczak, A. Young, M. Allison, D. L. Poole, S. G. Leach, J. A. Parkinson, T. Tuttle, J. A. Murphy, *Chem. Sci.* **2020**, *11*, 3719-3726; c) T. K. Allred, M. B. Shaghafi, P.-P. Chen, Q. Tran, K. N. Houk, L. E. Overman, *Org. Lett.* **2021**, *23*, 7618-7623; d) K. Maeda, R. Matsubara, M. Hayashi, *Org. Lett.* **2021**, *23*, 1530-1534; e) L. Ren, G. Nan, Y. Wang, Z. Xiao, *J. Org. Chem.* **2018**, *83*, 14472-14488; f) J. Zhang, S. Torabi Kohlbouni, B. Borhan, *Org. Lett.* **2019**, *21*, 14-17; g) D. Moock, T. Wagener, T. Hu, T. Gallagher, F. Glorius, *Angew. Chem., Int. Ed.* **2021**, *60*, 13677-13681.

[3] R. Kuwano, M. Kashiwabara, *Org. Lett.* **2006**, *8*, 2653-2655.

[4] a) J. A. Widegren, R. G. Finke, *J. Mol. Catal. A: Chem.* **2003**, *198*, 317-341; b) M. Zhang, M. Wang, B. Xu, D. Ma, *Joule* **2019**, *3*, 2876-2883; c) D. Moock, M. P. Wiesenfeldt, M. Freitag, S. Muratsugu, S. Ikemoto, R. Knitsch, J. Schneidewind, W. Baumann, A. H. Schafer, A. Timmer, M. Tada, M. R. Hansen, F. Glorius, *ACS Catal.* **2020**, *10*, 6309-6317.

[5] D. Bae, J. W. Lee, D. H. Ryu, *J. Org. Chem.* **2022**, *87*, 16532-16541.

[6] L. Pitzer, F. Schäfers, F. Glorius, *Angew. Chem., Int. Ed.* **2019**, *58*, 8572-8576.

[7] Bruker AXS (**2021**) *APEX4 Version 2021.4-0*, *SAINT Version 8.40B* and *SADABS Bruker AXS area detector scaling and absorption correction Version 2016/2*, Bruker AXS Inc., Madison, Wisconsin, USA.

[8] Sheldrick, G. M., *SHELXT – Integrated space-group and crystal-structure determination*, *Acta Cryst.,* **2015**, *A71*, 3-8.

[9] Sheldrick, G.M., *Crystal structure refinement with SHELXL*, *Acta Cryst.*, **2015**, *C71 (1)*, 3-8.

[10] Bruker AXS (**1998**) *XP – Interactive molecular graphics, Version 5.1,* Bruker AXS Inc., Madison, Wisconsin, USA.
